# Supplementary material for: Association of guideline and policy changes with incidence of lifestyle advice and treatment for uncomplicated mild hypertension in primary care: a longitudinal cohort study in the Clinical Practice Research Datalink
Source: BMJ Open. 2018 Sep 5;8(9):e021827. doi: 10.1136/bmjopen-2018-021827 (PMC6129091; doi:10.1136/bmjopen-2018-021827)
Supplement: Author's manuscript [file bmjopen-2018-021827.draft_revisions.pdf]

BMJ Open is committed to open peer review. As part of this commitment we make the peer review history of every article we publish publicly available.

When an article is published we post the peer reviewers' comments and the authors' responses online. We also post the versions of the paper that were used during peer review. These are the versions that the peer review comments apply to.

The versions of the paper that follow are the versions that were submitted during the peer review process. They are not the versions of record or the final published versions. They should not be cited or distributed as the published version of this manuscript.

BMJ Open is an open access journal and the full, final, typeset and author-corrected version of record of the manuscript is available on our site with no access controls, subscription charges or pay-per-view fees (http://bmjopen.bmj.com).

If you have any questions on BMJ Open's open peer review process please email info.bmjopen@bmj.com

BMJ Open

Association of guideline and policy changes with incidence of lifestyle advice and treatment for uncomplicated mild hypertension in Primary Care: a longitudinal cohort study in the Clinical Practice Research Datalink

Journal:	*BMJ Open*
Manuscript ID	bmjopen-2018-021827
Article Type:	Research
Date Submitted by the Author:	23-Jan-2018
Complete List of Authors:	Sheppard, James; University of Oxford, Nuffield Department of Primary Care Health Sciences<br>Stevens, Sarah; University of Oxford, Nuffield Department of Primary Care Health Sciences<br>Stevens, Richard; University of Oxford, Nuffield Dept Primary Care Health Sciences<br>Mant, Jonathan; University of Cambridge, General Practice and Primary Care Research Unit<br>Martin, Una; University of Birmingham, Institute of Clinical Sciences<br>Hobbs, Richard; University of Oxford, Dept of Primary Health Care<br>McManus, Richard; University of Oxford, Dept of Primary Care Health Sciences
Keywords:	Blood pressure, Interrupted time series, Electronic health records, Cardiovascular disease prevention, general practice

SCHOLARONE™
Manuscripts

For peer review only

Association of guideline and policy changes with incidence of lifestyle advice and treatment for uncomplicated mild hypertension in Primary Care: a longitudinal cohort study in the Clinical Practice Research Datalink

James P Sheppard, Ph.D,[1] Sarah Stevens, M.Sc,[1] Richard Stevens, Ph.D,[1] Jonathan Mant, MD,[2] Una Martin FRCP,[3] FD Richard Hobbs, FRCGP,[1] and Richard J McManus, FRCGP[1]

[1]University of Oxford, Oxford, UK
[2]University of Cambridge, Cambridge, UK
[3]University of Birmingham, Birmingham, UK

Corresponding author: James P Sheppard
Email: james.sheppard@phc.ox.ac.uk
Telephone: +44 1865 617192
Address: Nuffield Department of Primary Care Health Sciences, Radcliffe Observatory Quarter, University of Oxford, Oxford, OX2 6GG, UK

Word count: 3,235 (excluding title page, abstract, references, tables and figures)
Number of tables: 3
Number of figures: 3
Number of appendices: 3

For peer review only - http://bmjopen.bmj.com/site/about/guidelines.xhtml

Abstract

Objectives: Evidence to support initiation of pharmacological treatment in patients with uncomplicated (low risk) mild hypertension is inconclusive. As such, clinical guidelines are contradictory and healthcare policy has changed regularly. The aim of this study was to determine the incidence of lifestyle advice and drug therapy in this population and whether secular trends were associated with policy changes.

Design: Longitudinal cohort study.

Setting: Primary Care practices contributing to the Clinical Practice Research Datalink in England.

Participants: Data were extracted from the linked electronic health records of patients aged 18-74 years, with stage 1 hypertension (blood pressure between 140/90-159/99mmHg), no cardiovascular disease (CVD) risk factors and no treatment, from 1998-2015. Patients exited if follow-up records became unavailable, they progressed to stage 2 hypertension, developed a CVD risk factor or received lifestyle advice/treatment.

Primary outcome measures: The association between policy changes and incidence of lifestyle advice or treatment, examined using an interrupted time series analysis.

Results: A total of 108,935 patients were defined as having uncomplicated mild hypertension (mean age 51.9±12.9 years, 60.0% female). Patients were in the study spent for a median 2.6 years (IQR 0.9, 5.5), after which 12.2% (95%CI 12.0%-12.4%) were given lifestyle advice, 29.9% (95%CI 29.7%-30.2%) were prescribed medication and 19.4% (95%CI 19.2%-19.6%) were given both. The introduction of the Quality Outcomes Framework (QOF) and subsequent changes to QOF indicators were followed by significant increases in the incidence of lifestyle advice. Treatment prescriptions decreased slightly over time, but were not associated with policy changes.

Conclusions: Despite secular trends that accord with UK guidance, many patients are still prescribed treatment for mild hypertension. Adequately powered studies are needed to determine if this is appropriate.

Word count: 275

Keywords: Blood pressure, Interrupted time series, electronic health records, cardiovascular disease prevention, general practice

For peer review only - http://bmjopen.bmj.com/site/about/guidelines.xhtml

Strengths and weaknesses of this study

- This study examined electronic health records from a large, nationally representative sample of the UK population.
- Analyses were undertaken according to a pre-specified protocol which was reviewed and approved by an independent scientific advisory committee.
- Mild hypertension was defined consistently according to blood pressure readings, meaning that those with a diagnostic code for hypertension but no associated blood pressure values may have been excluded.
- Low risk patients were defined according to individual risk factors, rather than an established risk calculator due to large amounts of missing data (e.g. for cholesterol, BMI), potentially underestimating the number of truly low risk patients available for analysis.
- The incidence of lifestyle advice was defined according to clinical codes but it was not possible to identify patients given lifestyle advice where it was either not coded or where it was only recorded in the free text consultation notes.

For peer review only

For peer review only - http://bmjopen.bmj.com/site/about/guidelines.xhtml

Introduction

High blood pressure (hypertension) is a key risk factor for the development of cardiovascular disease,[1] a major cause of mortality worldwide.[2] The threshold for hypertension is a sustained blood pressure above 140/90mmHg, and many guidelines recommend pharmacological treatment at this level, regardless of the underlying risk of cardiovascular disease.[3-8] These recommendations are considered controversial, particularly with regard to treatment of uncomplicated (*i.e.* low cardiovascular disease risk) people with 'mild' hypertension (*i.e.* sustained blood pressure between 140/90-159/99mmHg [stage 1]).[9-13]

There is little evidence to support initiation of pharmacological treatment in patients with uncomplicated mild hypertension. A Cochrane review[14] of trials in this population found no reduction in mortality or cardiovascular events with treatment, but was underpowered to detect significant differences.[10 14] More recent studies[15 16] have shown benefit with treatment in patients with mild hypertension. However, these included a significant proportion of patients with diabetes or at least one other cardiovascular risk factor and therefore participants would not be considered 'uncomplicated' under current clinical guidelines.[17]

Clinical guidelines often make recommendations based on 'expert opinion' where evidence is lacking or inconsistent. Such guidelines are predictably contradictory, with those in the UK promoting lifestyle modification in low risk patients[17] whilst guidelines from Europe[6] and the US[4 7 8] encourage prescription of drug therapy. Against this background, a number of guideline and policy changes have occurred in the UK over the past 10 years which may have affected general practitioner's treatment decisions. In particular, the introduction of a nationwide cardiovascular risk screening programme (the NHS health check) in 2009,[18] and pay-for-performance indicators (the Quality and Outcomes Framework [QOF]), which promoted both more lifestyle advice and stricter treatment targets in this population.[19]

The aim of this study was to use routine patient records from the Clinical Practice Research Datalink (CPRD)[20] to establish the proportion of patients given lifestyle advice and/or pharmacological treatment for uncomplicated mild hypertension in Primary Care and the association of policy with changes to these proportions over time.

Methods

Design

This was a longitudinal cohort study in conducted in Primary Care from 1998 to 2015 using data from the Clinical Practice Research Datalink (CPRD), a database of electronic health records from England.[20] The incidence of lifestyle advice and treatment prescriptions was estimated by month, adjusted for age and sex. The association of guideline and policy changes was examined using an interrupted time series analysis with introduction of the NHS health check[18] and changes in QOF indicators[19] defined as pre-specified break points. The CPRD population has been shown previously to approximate to the UK population.[20]

Study population

Individual patient data were extracted from the medical records of all patients registered at general practices contributing to the CPRD in England. Patients were entered into the study on the date of

For peer review only - http://bmjopen.bmj.com/site/about/guidelines.xhtml

the third consecutive blood pressure reading between 140/90-159/99mmHg occurring after the study start date 01/01/1998. The start date was chosen because it represents the date from which all relevant data linkages were first available. Patients exited the study if they had three consecutive blood pressure readings above 160/100mmHg (i.e. progressed to stage 2 hypertension), developed a risk factor for cardiovascular disease, received lifestyle advice or treatment or follow-up records became unavailable (patient or practice left the CPRD) (table 1). The last day of follow-up for those remaining in the study was 30th September 2015 (last day of follow-up in linked data).

Outcomes

The primary outcome of this study was evidence that lifestyle advice and/or pharmacological treatment had been given during follow-up. Lifestyle advice was defined as Read code for any lifestyle advice relating to smoking, alcohol, diet, hypertension, physical activity, weight, oral health or lipid disorders (see online appendix, eTable 1). Pharmacological treatment was defined as a coded prescription of an angiotensin-converting-enzyme inhibitor, angiotensin-II-receptor blocker, calcium channel blocker, thiazide or thiazide-like diuretic, beta-blocker, alpha-blocker or any other antihypertensive listed in the British National Formulary (see online appendix, eTable 2).

Covariates

Data relating to baseline patient characteristics (age, sex, ethnicity, [patient level] deprivation), lifestyle factors (smoking status [never, current, ex-smoker], alcohol consumption [units per week], and BMI), pre-treatment blood pressure readings (in the preceding *12 months*), non-cardiovascular related co-morbidities (rheumatoid arthritis, hypercholesterolemia [Read code or most recent total cholesterol value $\geq$7.5mmol/l]) and all prescribed statin/antiplatelet medications were extracted from the medical records of eligible patients and used to define the study population.

Quantitative data

Ethnicity was classified into white, black, South Asian, mixed race, other and unknown groups. The Index of Multiple Deprivation (IMD) was provided as quintiles of deprivation in England, with the highest quintile indicating the highest level of deprivation. Smoking status was defined as never, previous, or current smoker. Those with unknown smoking status were assumed to have never smoked. Where there was no record of blood pressure lowering, statin or antiplatelet treatment, it was assumed that patients were not prescribed treatment.

Only patients with records deemed 'acceptable for research purposes' by CPRD were included in the analysis, and hence age and sex information was complete. Where there was no record of alcohol consumption, rheumatoid arthritis or hypercholesterolemia, patients were assumed to be non-drinkers and no history of these conditions. A total of 91 patients (0.001%) were missing IMD data and were excluded. To enable calculation of a cardiovascular risk score in sensitivity analysis, missing values for cholesterol and BMI were simulated once, assuming a normal distribution, and using means and standard deviations in each age-sex strata from the 2011 Health Survey for England.[21]

Sample size

As a descriptive study, no formal sample size calculation was required. All patients from the CPRD fulfilling the eligibility criteria were included.

For peer review only - http://bmjopen.bmj.com/site/about/guidelines.xhtml

Statistical analysis

Descriptive statistics were used to describe the study population and estimate the proportion of patients being offered lifestyle advice or therapy (or both) following a diagnosis of uncomplicated mild hypertension.

The impact of the introduction of the NHS health check programme in April 2009[18] (month 111) and changes to QOF indicators (April 2009 and 2013 [month 159])[19] (see table 2) on lifestyle advice and treatment prescription rates were examined using an interrupted time series analysis. A two year run-in phase (1998-1999) was used, where patients could enter and leave the cohort, but incidence rates were not estimated. This was to ensure the cohort accumulated sufficient numbers of patients to enable accurate estimates of monthly incidence in the subsequent time period after the analysis start date (January 1st 2000). The slope of the monthly rate of lifestyle advice and treatment over time was estimated (189 months in total), before policy change was introduced (months 111 and 159), along with the difference in the starting level (intercept) and slope of this rate in the period after each policy change was introduced, using ordinary least squares regression with Newey-West corrected standard errors (which account for autocorrelation). Estimates were adjusted for age and sex and all slopes are presented as incidence per year. P values indicate significance of the difference between the slope/intercept in the post policy period, compared to estimates from the pre-policy period.

As a post-hoc analysis, the impact of the introduction of the QOF in 2004[22] (month 59) was explored as an additional break-point in the interrupted time-series analysis. Sensitivity analyses explored the impact of excluding those deemed to be at high risk due to 1) high cardiovascular risk score documented in the primary care physician notes and 2) high cardiovascular risk score documented in the notes or estimated using the QRISK2 algorithm[23] and available risk factor data.

Data access and cleaning methods

SS had access to the entire CPRD database from which eligible patients were identified and all data extracted. Where multiple blood pressure values appeared in the record on any one day, the lowest value was selected. Treatments/diagnoses of conditions were determined from the presence of at least one coded prescription/diagnostic code respectively. Baseline data were extracted from within each patient's up to standard registration period and were drawn from the latest available record prior to study entry. Values deemed *a priori* to be clinically implausible were excluded (e.g. weight values of less than 20kg and more than 200kg). Where there was evidence of prior smoking but the most recent smoking status recorded indicated no smoking, this was corrected by classifying patients as former smokers.

Measures taken to reduce bias

To reduce the possibility of selection bias, all patients fulfilling the eligibility criteria for the study were included. Mild hypertension was defined according to measured blood pressure values rather than diagnostic codes, to reduce the potential for reporting bias, particularly in the pre-QOF era when coding was less standardised. For the primary analysis, breakpoints for the interrupted time series analysis were pre-specified based on policy changes thought likely to influence the giving of lifestyle advice and treatment, rather than trends observed in the data.

For peer review only - http://bmjopen.bmj.com/site/about/guidelines.xhtml

Data linkage

Data linkage to the Office for National Statistics (ONS) mortality register was used to define patient mortality (patient exit from the study). Linkages to Basic Inpatient Hospital Episode Statistics (HES) were used in conjunction with CPRD records to define patient eligibility for the study (e.g. previous stroke), and define patient ethnicity (where unavailable in Primary Care records). A linkage to the Index of Multiple Deprivation was required to acquire practice level indices of multiple deprivation.

Patient involvement

Patients were not involved in the development or design of this study.

Approval

The study protocol (16_008R) was approved by CPRD's Independent Scientific Advisory Committee (ISAC) in March 2016, prior to obtaining the data cut (protocol given in the online appendix). A project summary was published on the CPRD website (https://www.cprd.com/isac).

All data cleaning and analyses were conducted using STATA version 13.1 and 14.2 (MP parallel edition, StataCorp, Texas, USA). Results are presented as means, medians or proportions, with standard deviations, interquartile range or 95% confidence intervals, unless otherwise stated.

Results

Prevalence of lifestyle advice and treatment

Of the 7,416,968 individuals aged 18-74 years registered at linked, up-to-standard practices within the CPRD during the study period, a total of 108,844 patients (1.46%, 95% CI 1.45%, 1.47%) from 694 practices met the criteria for having uncomplicated mild hypertension during the study period (figure 1). Included patients had a mean age of 51.9 ± 12.9 years, 60.0% (95% CI 59.7%, 60.3%) were female and mean blood pressure (across 3 visits) was 144.3 ± 5.9 /87.3 ± 5.8 mmHg (table 3). Patients spent a median time of 2.6 years (IQR 0.9, 5.5) in the study, after which 13,269 (12.2%, 95% CI 12.0%, 12.4%) were given lifestyle advice alone, 32,578 (29.9%, 95% CI 29.7%, 30.2%) were only prescribed an antihypertensive and 21,128 (19.4%, 95% CI 19.2%, 19.6%) were given lifestyle advice and prescribed treatment (table 3).

Interrupted time series analysis

In the pre-NHS health check period from January 1999 and to April 2009, the proportion of patients receiving lifestyle advice increased from zero by 0.04% per year (95% CI 0.04%, 0.05%) (figure 2a). The introduction of the NHS health check and new QOF indicators in April 2009 was associated with a significant drop in lifestyle advice being given (p<0.001), but thereafter it increased by 0.19% per year (95% CI 0.15%, 0.23%). Following changes to QOF indicators in April 2013, the incidence of lifestyle advice decreased by -0.27% per year (95% CI -0.35%, -0.19%).

Treatment prescriptions were given to approximately 1.29% (95% CI 1.23%, 1.34%) of patients with uncomplicated mild hypertension in the first month of the study, but the incidence then decreased by -0.05% (95% CI -0.06%, -0.05%) per year in the pre-policy period until 2009 (figure 2b). Neither the introduction of the NHS health check, nor changes to QOF indicators significantly affected the incidence of treatment prescriptions which decreased by -0.03% per year and then -0.10% per year in the post NHS health check and QOF indicator change periods respectively (figure 2b).

For peer review only - http://bmjopen.bmj.com/site/about/guidelines.xhtml

Post-hoc analysis

The introduction of the QOF and clinical guidelines for the management of hypertension in 2004 was associated with a step-change increase in the incidence of lifestyle advice of 0.15% (95% CI 0.05%, 0.26%; p=0.006) (figure 3a). Subsequently, there was a small but significant decrease in the incidence of lifestyle advice (-0.05% per year, 95% CI -0.07%, -0.04%). The NHS health check and new QOF indicators in 2009 were no longer associated with a significant drop in lifestyle advice when the introduction of the QOF (in 2004) was included as a cut-point in the post-hoc analysis (p=0.07). No policy or guideline changes were associated with significant changes in the incidence of treatment prescriptions for uncomplicated mild hypertension during the study period in the post-hoc analysis (figure 3b). Exclusion of high risk patients (using either definition) in the sensitivity analysis had no impact on the incidence of lifestyle advice or treatment prescription.

Discussion

The present study examined a large database of electronic health records to establish the incidence of guideline recommended lifestyle advice and non-guideline recommended treatment for uncomplicated mild hypertension in Primary Care. Across the study period, approximately one third of patients received lifestyle advice whilst half were prescribed antihypertensive treatment. The introduction of pay-for-performance indicators in 2004,[22] and more recently a cardiovascular risk screening programme,[18] was associated with an increase in the proportion of patients documented as having been offered lifestyle advice, suggesting a more guideline based approach to the management of uncomplicated mild hypertension. However, there remains an appreciable proportion receiving pharmacological treatment and this seems to be unaffected by changes to guidelines and policy.

Strengths and limitations

This study examined electronic health records from a large, nationally representative sample of the UK population.[20] Participating practices included those which adhere to data quality standards set out by the CPRD to ensure accuracy of data studied. Analyses were undertaken according to a pre-specified protocol (see online appendix) which was reviewed and approved by an independent scientific advisory committee, minimising the possibility of reporting bias and data-mining.

In an attempt to define mild hypertension consistently across the population, it is possible that some patients with a diagnostic code for hypertension but no associated blood pressure values may have been excluded. The focus of this study was on 'uncomplicated' low risk patients, but for pragmatic reasons, no attempt was made in the primary analysis to exclude those with a high risk of cardiovascular disease according to an established risk calculator[23 24] due to large amounts of missing data (e.g. for cholesterol, BMI). Whilst it is possible that some high risk patients may have been included in the final study cohort, sensitivity analysis excluding patients with known high risk scores and those with a high estimated QRISK2 score[23] did not affect the study results.

The incidence of lifestyle advice was defined according to clinical codes related to the giving of lifestyle advice which might be of relevance to the management of hypertension and cardiovascular disease risk (see online appendix eTable 1). It was not possible to identify patients given lifestyle advice where it was either not coded or where it was only recorded in the free text consultation

For peer review only - http://bmjopen.bmj.com/site/about/guidelines.xhtml

notes. The trend of increasing lifestyle advice coinciding with the introduction of the QOF suggests that it may have been under recorded, particularly in the early observational period.

Previous literature

To our knowledge, this is the first study to determine the incidence of lifestyle advice and treatment in patients with uncomplicated mild hypertension. Previous studies[25-29] have focused on the prevalence of hypertension (defined as a blood pressure $\geq$140/90mmHg) and whether or not patients are treated and controlled, without making the important distinction between hypertensive patients where treatment is appropriate and those where it is not, at least according to national guidelines.[17] These surveys estimate the prevalence of hypertension across the world at between 20-45% with estimates of treatment and control ranging from 55%-81% and 30-59% respectively.[28] Our data suggest that in uncomplicated mild hypertension, treatment rates are slightly lower (49% across the study period), perhaps reflecting uncertainty around its efficacy in this population.

We found a trend towards increased recording of lifestyle advice over time, particularly following the introduction of the QOF in 2004,[22] the NHS health check[18] and changes in QOF indicators in 2009. Previous studies examining the impact of the NHS health check have found no effect on the estimated prevalence of hypertension,[30] but in those screened, referrals to specialist clinics and programmes for lifestyle advice increased six-fold.[31] The apparent association between incidence of lifestyle advice and changes in QOF indicators is in contrast to previous studies which have suggested that pay-for-performance in the UK has not affected the quality of care for hypertension.[32] Whilst previous data have indicated that equivalent quality care remains, even after an indicator has been removed,[33] the present data suggest the opposite, with the incidence of recording of lifestyle advice decreasing after the indicator encouraging this was removed in 2014.[34]

The incidence of treatment prescriptions slightly decreased over time during the study with the largest reduction observed in the last study period (post 2013), perhaps due to more accurate blood pressure measurement and targeting of treatment brought about by new NICE guidelines and the introduction of out-of-office blood pressure measurement for diagnosis in 2011.[17] The overall decrease in treatment might suggest a change in GP treatment practice over the past 15 years. However, it is also possible that GP treatment practice has remained constant over time, and the number of hypertensive patients being diagnosed and perceived as low risk has increased, as evidenced by the increasing prevalence of hypertension reported annually in the QOF over the past 10 years (from 12.5% to 13.8%).

Implications for clinical practice

The peak in prevalence of lifestyle advice coinciding with the inclusion of QOF indicators encouraging advice for physical activity, smoking cessation and diet in 2013,[19] suggests that GPs were following pay-for-performance indicators closely. One explanation might be the content of QOF templates on GP computer systems, which are changed depending on the indicators included in the QOF and are likely to have encouraged better coding of lifestyle advice during a GP consultation. The choice of pay-for-performance indicators to include and exclude in the QOF, should therefore be considered carefully, ensuring that such indicators reflect evidence based practice and optimal patient care.

For peer review only - http://bmjopen.bmj.com/site/about/guidelines.xhtml

One could argue that the evidence for giving lifestyle advice in uncomplicated mild hypertension is as inconclusive as the evidence for pharmacological interventions, with many trials having short follow-up and exhibiting risk of bias[17 35] such as inadequate randomisation and allocation concealment,[35] lack of blinding[36] and incomplete outcome data.[37] In addition, there are no trials of the impact of lifestyle advice on 'hard outcomes' such as cardiovascular disease and mortality[17] and the quality of evidence of an association from observational studies is generally low.[38] However, lifestyle advice can potentially be given at lost cost within a patient consultation, and whilst overall reductions in blood pressure may be modest,[35-37] some patients may wish to change their lifestyle in an effort to delay or prevent embarking on a formal treatment regime.[35]

Conclusions

The present study shows that the introduction of pay-for-performance indicators and a cardiovascular risk screening programme have been associated with increased guideline recommended lifestyle advice being offered to patients with uncomplicated mild hypertension, although the overall prevalence remains relatively low. However, nearly half of patients go on to receive treatment, despite a lack of evidence suggesting benefit. This may be explained by a lack of clear evidence to guide treatment decisions. Adequately powered studies, perhaps using routine clinical data at scale are required to accurately define the relationship between treatment and cardiovascular disease and death in patients with uncomplicated mild hypertension.

For peer review only

For peer review only - http://bmjopen.bmj.com/site/about/guidelines.xhtml

Acknowledgements

The authors would like to thank Dr Blanca Gallego Luxan for reviewing and commenting on the study protocol.

Funding

This work was funded by an Medical Research Council (MRC) Strategic Skills Post-doctoral Fellowship (MR/K022032/1) held by JS. JS now receives funding from the National Institute for Health Research (NIHR) Collaboration for Leadership in Applied Health Research and Care Oxford at Oxford Health NHS Foundation Trust and an NIHR Professorship which is held by and also funds RJMcM (NIHR-RP-R2-12-015). FDRH acknowledges support from the NIHR as Director of the NIHR SPCR, Director of the NIHR CLARHC Oxford, Theme Leader of the NIHR Oxford BRC, NIHR Oxford DEC and also from Harris Manchester College. The views and opinions expressed are those of the authors and do not necessarily reflect those of the MRC, NHS, NIHR, or the Department of Health.

Competing interests

RS is a member of the CPRD's Independent Scientific Advisory Committee (ISAC), but was not involved in the approval of this study. All other authors report no relevant conflicts of interest.

Author's contributions

JS conceived and designed the study, secured funding, undertook the main analysis and drafted the manuscript. SS was responsible for the data management, assisted with the analysis and revised the manuscript. RS assisted with the study design, assisted with the analysis and revised the manuscript. JM assisted with the study design and revised the manuscript. UM assisted with the study design and revised the manuscript. RH assisted with the study design and revised the manuscript. RJM conceived the study, assisted with the study design and revised the manuscript.

Data sharing

Data used in this analysis was provided by the Clinical Practice Research Datalink under a Gold Licence and cannot be shared with other parties.

For peer review only

References

1. Lewington S, Clarke R, Qizilbash N, et al. Age-specific relevance of usual blood pressure to vascular mortality: a meta-analysis of individual data for one million adults in 61 prospective studies. *Lancet* 2002;360(9349):1903-13. [published Online First: 2002/12/21]

2. Lozano R, Naghavi M, Foreman K, et al. Global and regional mortality from 235 causes of death for 20 age groups in 1990 and 2010: a systematic analysis for the Global Burden of Disease Study 2010. *Lancet* 2012;380(9859):2095-128. doi: 10.1016/s0140-6736(12)61728-0 [published Online First: 2012/12/19]

3. Whitworth JA. 2003 World Health Organization (WHO)/International Society of Hypertension (ISH) statement on management of hypertension. *Journal of hypertension* 2003;21(11):1983-92. doi: 10.1097/01.hjh.0000084751.37215.d2 [published Online First: 2003/11/05]

4. Sanchez RA, Ayala M, Baglivo H, et al. Latin American guidelines on hypertension. Latin American Expert Group. *Journal of hypertension* 2009;27(5):905-22. doi: 10.1097/HJH.0b013e32832aa6d2 [published Online First: 2009/04/08]

5. Ogihara T, Kikuchi K, Matsuoka H, et al. The Japanese Society of Hypertension Guidelines for the Management of Hypertension (JSH 2009). *Hypertension research : official journal of the Japanese Society of Hypertension* 2009;32(1):3-107. [published Online First: 2009/03/21]

6. Mancia G, Fagard R, Narkiewicz K, et al. 2013 ESH/ESC Guidelines for the management of arterial hypertension: the Task Force for the management of arterial hypertension of the European Society of Hypertension (ESH) and of the European Society of Cardiology (ESC). *Journal of hypertension* 2013;31(7):1281-357. doi: 10.1097/01.hjh.0000431740.32696.cc [published Online First: 2013/07/03]

7. James PA, Oparil S, Carter BL, et al. 2014 evidence-based guideline for the management of high blood pressure in adults: report from the panel members appointed to the Eighth Joint National Committee (JNC 8). *JAMA : the journal of the American Medical Association* 2014;311(5):507-20. doi: 10.1001/jama.2013.284427 [published Online First: 2013/12/20]

8. Hackam DG, Quinn RR, Ravani P, et al. The 2013 Canadian Hypertension Education Program recommendations for blood pressure measurement, diagnosis, assessment of risk, prevention, and treatment of hypertension. *The Canadian journal of cardiology* 2013;29(5):528-42. doi: 10.1016/j.cjca.2013.01.005 [published Online First: 2013/04/02]

9. Toth PJ, Horwitz RI. Conflicting clinical trials and the uncertainty of treating mild hypertension. *The American journal of medicine* 1983;75(3):482-8. [published Online First: 1983/09/01]

10. Tavares A. Pharmacotherapy for mild hypertension. *Sao Paulo medical journal = Revista paulista de medicina* 2012;130(6):417-8. [published Online First: 2013/01/23]

11. Ramsay LE, ul Haq I, Yeo WW, et al. Interpretation of prospective trials in hypertension: do treatment guidelines accurately reflect current evidence? *Journal of hypertension Supplement : official journal of the International Society of Hypertension* 1996;14(5):S187-94. [published Online First: 1996/12/01]

12. Heath I. Waste and harm in the treatment of mild hypertension. *JAMA internal medicine* 2013;173(11):956-7. doi: 10.1001/jamainternmed.2013.970 [published Online First: 2013/05/24]

For peer review only - http://bmjopen.bmj.com/site/about/guidelines.xhtml

13. Hart JT. Historical footnote on the treatment of mild hypertension. *BMJ (Clinical research ed)* 2012;345:e6297. doi: 10.1136/bmj.e6297 [published Online First: 2012/09/26]

14. Diao D, Wright JM, Cundiff DK, et al. Pharmacotherapy for mild hypertension. *The Cochrane database of systematic reviews* 2012;8:Cd006742. doi: 10.1002/14651858.CD006742.pub2 [published Online First: 2012/08/17]

15. Sundstrom J, Arima H, Jackson R, et al. Effects of blood pressure reduction in mild hypertension: a systematic review and meta-analysis. *Annals of internal medicine* 2015;162(3):184-91. doi: 10.7326/m14-0773 [published Online First: 2014/12/23]

16. Wright JT, Jr., Williamson JD, Whelton PK, et al. A Randomized Trial of Intensive versus Standard Blood-Pressure Control. *The New England journal of medicine* 2015;373(22):2103-16. doi: 10.1056/NEJMoa1511939 [published Online First: 2015/11/10]

17. National Clinical Guideline Centre. Hypertension: clinical management of primary hypertension in adults; Clinical guideline 127. London: Royal College of Physicians (UK), 2011.

18. Public Health England. NHS Health Check: our approach to the evidence. https://www.gov.uk/government/uploads/system/uploads/attachment_data/file/224537/NHS_Health_Check_our_approach_to_the_evidence_v2.pdf: NHS England, 2013.

19. NHS Employers GPC, NHS England. General Medical Services (GMS) contract Quality and Outcomes Framework (QOF): guidance for GMS contract 2013/14. Leeds: NHS Employers, 2013.

20. Herrett E, Gallagher AM, Bhaskaran K, et al. Data Resource Profile: Clinical Practice Research Datalink (CPRD). *International journal of epidemiology* 2015;44(3):827-36. doi: 10.1093/ije/dyv098 [published Online First: 2015/06/08]

21. Joint Health Surveys Unit. Health Survey for England 2011. http://digital.nhs.uk/catalogue/PUB09300: Health and Social Care Information Centre, 2012.

22. NHS Employers GPC, NHS England. General Medical Services (GMS) contract Quality and Outcomes Framework (QOF): guidance for GMS contract 2004/05. Leeds: NHS Employers, 2004.

23. Hippisley-Cox J, Coupland C, Vinogradova Y, et al. Predicting cardiovascular risk in England and Wales: prospective derivation and validation of QRISK2. *BMJ (Clinical research ed)* 2008;336(7659):1475-82. doi: 10.1136/bmj.39609.449676.25 [published Online First: 2008/06/25]

24. D'Agostino RB, Sr., Vasan RS, Pencina MJ, et al. General cardiovascular risk profile for use in primary care: the Framingham Heart Study. *Circulation* 2008;117(6):743-53. doi: 10.1161/circulationaha.107.699579 [published Online First: 2008/01/24]

25. Joffres M, Falaschetti E, Gillespie C, et al. Hypertension prevalence, awareness, treatment and control in national surveys from England, the USA and Canada, and correlation with stroke and ischaemic heart disease mortality: a cross-sectional study. *BMJ open* 2013;3(8):e003423. doi: 10.1136/bmjopen-2013-003423 [published Online First: 2013/09/03]

26. Neuhauser HK, Adler C, Rosario AS, et al. Hypertension prevalence, awareness, treatment and control in Germany 1998 and 2008-11. *Journal of human hypertension* 2015;29(4):247-53. doi: 10.1038/jhh.2014.82 [published Online First: 2014/10/03]

For peer review only - http://bmjopen.bmj.com/site/about/guidelines.xhtml

27. Labeit AM, Klotsche J, Pieper L, et al. Changes in the prevalence, treatment and control of hypertension in Germany? A clinical-epidemiological study of 50.000 primary care patients. *PloS one* 2012;7(12):e52229. doi: 10.1371/journal.pone.0052229 [published Online First: 2013/01/04]

28. Ikeda N, Sapienza D, Guerrero R, et al. Control of hypertension with medication: a comparative analysis of national surveys in 20 countries. *Bulletin of the World Health Organization* 2014;92(1):10-19c. doi: 10.2471/blt.13.121954

29. Wolf-Maier K, Cooper RS, Kramer H, et al. Hypertension treatment and control in five European countries, Canada, and the United States. *Hypertension (Dallas, Tex : 1979)* 2004;43(1):10-7. doi: 10.1161/01.hyp.0000103630.72812.10 [published Online First: 2003/11/26]

30. Caley M, Chohan P, Hooper J, et al. The impact of NHS Health Checks on the prevalence of disease in general practices: a controlled study. *The British journal of general practice : the journal of the Royal College of General Practitioners* 2014;64(625):e516-21. doi: 10.3399/bjgp14X681013 [published Online First: 2014/07/30]

31. Robson J, Dostal I, Sheikh A, et al. The NHS Health Check in England: an evaluation of the first 4 years. *BMJ open* 2016;6(1):e008840. doi: 10.1136/bmjopen-2015-008840 [published Online First: 2016/01/15]

32. Serumaga B, Ross-Degnan D, Avery AJ, et al. Effect of pay for performance on the management and outcomes of hypertension in the United Kingdom: interrupted time series study. *BMJ (Clinical research ed)* 2011;342:d108. doi: 10.1136/bmj.d108 [published Online First: 2011/01/27]

33. Kontopantelis E, Springate D, Reeves D, et al. Withdrawing performance indicators: retrospective analysis of general practice performance under UK Quality and Outcomes Framework. *BMJ (Clinical research ed)* 2014;348:g330. doi: 10.1136/bmj.g330 [published Online First: 2014/01/29]

34. NHS Employers GPC, NHS England. General Medical Services (GMS) contract Quality and Outcomes Framework (QOF): guidence for GMS contract 2014/15. Leeds: NHS Employers, 2014.

35. Dickinson HO, Mason JM, Nicolson DJ, et al. Lifestyle interventions to reduce raised blood pressure: a systematic review of randomized controlled trials. *Journal of hypertension* 2006;24(2):215-33. doi: 10.1097/01.hjh.0000199800.72563.26 [published Online First: 2006/03/02]

36. Cornelissen VA, Smart NA. Exercise Training for Blood Pressure: A Systematic Review and Meta-analysis. *Journal of the American Heart Association: Cardiovascular and Cerebrovascular Disease* 2013;2(1) doi: 10.1161/jaha.112.004473

37. Ndanuko RN, Tapsell LC, Charlton KE, et al. Dietary Patterns and Blood Pressure in Adults: A Systematic Review and Meta-Analysis of Randomized Controlled Trials. *Advances in nutrition (Bethesda, Md)* 2016;7(1):76-89. doi: 10.3945/an.115.009753 [published Online First: 2016/01/17]

38. Aburto NJ, Ziolkovska A, Hooper L, et al. Effect of lower sodium intake on health: systematic review and meta-analyses. *BMJ (Clinical research ed)* 2013;346:f1326. doi: 10.1136/bmj.f1326 [published Online First: 2013/04/06]

For peer review only

For peer review only - http://bmjopen.bmj.com/site/about/guidelines.xhtml

Tables

Table 1. Study entry and exit criteria applied to potential participant records used in the study.

**Study entry criteria**	**Study exit (first to occur)**
Date of the third consecutive blood pressure reading between 140/90-159/99mmHg (within 12 months of each other)	Last date at which the most recent linked data are available from the CPRD (study end date, September 2015)
Aged between 18-74 years	Date of third consecutive blood pressure reading ≥above 160/100mmHg (within 12 months of each other) (progression to stage 2 hypertension)
Linked general practice, Hospital Episodes Statistics (HES) and Office for National Statistics (ONS) mortality records	Date of first record of a cardiovascular risk factor (left ventricular hypertrophy, atrial fibrillation, diabetes or chronic kidney disease or family history of premature heart disease) or high cardiovascular risk score (>20%)
Registered to a CPRD practice classified as 'up-to-standard'	Date of death
Classified as a CPRD 'acceptable patient'	Date of the most recent data upload from the practice to which a given patient is registered
No read code for previous cardiovascular disease (stroke, myocardial infarction, angina, coronary heart disease, peripheral vascular disease, heart failure)	Date at which a given patient transfers out of a registered CPRD practice
No read code for cardiovascular risk factor (left ventricular hypertrophy, atrial fibrillation, diabetes or chronic kidney disease or family history of premature heart disease)	Date of first coded lifestyle advice given after study entry (lifestyle advice analysis only)
No record of any blood pressure lowering medication prescription in the 12 months prior to the third consecutive blood pressure reading between 140/90-159/99 mmHg	Date of first prescribed antihypertensive treatment given after study entry (treatment analysis only)

CPRD = Clinical Practice Research Datalink

For peer review only

Table 2. Guideline and policy changes related to the ongoing management of hypertension (columns highlighted in dark grey indicate pre-specified cut points, column in light grey indicates additional post-hoc cut point for interrupted time series analysis)

Guideline/policy/QOF indicator	1998/ 1999	1999/ 2000	2001/ 2002	2001/ 2002	2002/ 2003	2003/ 2004	2004/ 2005	2005/ 2006	2006/ 2007	2007/ 2008	2008/ 2009	2009/ 2010	2010/ 2011	2011/ 2012	2012/ 2013	2013/ 2014	2014/ 2015	2015/ 2016
**NICE Clinical Guideline 18 (2004):** Essential hypertension: managing adult patients in primary care							✓	✓										
**NICE Clinical Guideline 34 (2006):** Management of hypertension in adults in Primary Care									✓	✓	✓	✓	✓					
**NICE Clinical Guideline 127 (2011):** Hypertension in adults: diagnosis and management														✓	✓	✓	✓	✓
**Policy change:** NHS health check cardiovascular risk screening programme introduced												✓	✓	✓	✓	✓	✓	✓
**QOF Hypertension indicator:** The percentage of patients with hypertension in whom there is a record of the blood pressure in the previous 9 months							✓	✓	✓	✓	✓	✓	✓	✓	✓			
**QOF Hypertension indicator:** The percentage of patients with hypertension in whom the last blood pressure (measured in the previous 9 months) is 150/90 or less							✓	✓	✓	✓	✓	✓	✓	✓	✓	✓	✓	✓
**QOF Hypertension indicator:** The percentage of patients aged 79 or under with hypertension in whom the last blood pressure reading (measured in the preceding 9 months) is 140/90 mmHg or less																✓		
**QOF Hypertension indicator:** The percentage of patients with hypertension who have not attained the age of 75 in whom there is an assessment of physical activity, using GPPAQ, in the preceding 12 months																✓		
**QOF Hypertension indicator:** The percentage of patients with hypertension aged 16 or over and who have not attained the age of 75 who score 'less than active' on GPPAQ in the preceding 12 months, who also have a record of a brief intervention																✓		
**QOF Blood pressure indicator:** The percentage of patients aged 40 or over who have a record of							✓	✓	✓	✓	✓	✓	✓	✓	✓	✓	✓	✓

For peer review only

For peer review only - http://bmjopen.bmj.com/site/about/guidelines.xhtml

blood pressure in the preceding 5 years																		
**QOF Smoking indicator:** The percentage of patients with hypertension who smoke whose notes contain a record that smoking cessation advice or referral to a specialist service, where available, has been offered within the previous 15 months							✓	✓	✓	✓	✓	✓	✓	✓	✓	✓	✓	✓
**QOF Smoking indicator:** The percentage of patients with hypertension whose notes record smoking status in the previous 15 months. Except those who have never smoked where smoking status need only be recorded once since diagnosis							✓	✓	✓	✓	✓	✓	✓	✓	✓	✓	✓	✓
**QOF Smoking indicator:** The contractor supports patients who smoke in stopping smoking by a strategy which includes providing literature and offering appropriate therapy																✓	✓	✓
**QOF Primary prevention of cardiovascular disease indicator:** In those patients with a new diagnosis of hypertension: the percentage of patients who have had a face to face cardiovascular risk assessment at the outset of diagnosis using an agreed risk assessment tool.												✓	✓	✓	✓			
**QOF Primary prevention of cardiovascular disease indicator:** The percentage of people diagnosed with hypertension who are given lifestyle advice for increasing physical activity, smoking cessation, safe alcohol consumption and healthy diet.												✓	✓	✓	✓	✓		
**QOF Primary prevention of cardiovascular disease indicator:** In those patients with a new diagnosis of hypertension, who have a recorded CVD risk assessment score of ≥20% in the preceding 12 months: the percentage who are currently treated with statins																✓	✓	✓

QOF=Quality and outcomes Framework; NHS=National Health Service; GPPAQ=General practice physical activity questionnaire; CVD=Cardiovascular disease

For peer review only

For peer review only - http://bmjopen.bmj.com/site/about/guidelines.xhtml

Table 3. Patient characteristics at study entry, stratified by subsequent outcome

Characteristic	Total population		Went on to receive no advice or treatment		Went on to receive advice only		Went on to receive treatment only		Went on to receive advice and treatment	
	**Mean/ number**	**SD/%**	**Mean/ number**	**SD/%**	**Mean/ number**	**SD/%**	**Mean/ number**	**SD/%**	**Mean/ number**	**SD/%**
Total population	108,843	100%	41,868	38.5%	13,269	12.2%	32,578	29.9%	21,128	19.4%
Age (years)	51.9	12.9	50.0	13.8	48.6	13.3	54.7	11.8	53.2	11.4
Sex (% male)	43567	40.0%	15809	37.8%	5443	41.0%	13054	40.1%	9261	43.8%
Time in study (years, median [IQR])	2.6	0.9, 5.5	4.6	2.5, 7.9	1.7	0.4, 4.1	1.6	0.4, 3.8	1.2	0.2, 3.2
Ethnicity										
White ethnicity	59643	54.8%	21447	51.2%	7554	56.9%	17939	55.1%	12703	60.1%
Black ethnicity	1527	1.4%	501	1.2%	162	1.2%	458	1.4%	406	1.9%
South Asian ethnicity	1356	1.2%	437	1.0%	133	1.0%	487	1.5%	299	1.4%
Mixed race ethnicity	9700	8.9%	3691	8.8%	1483	11.2%	2566	7.9%	1960	9.3%
Other ethnicity	1041	1.0%	398	1.0%	111	0.8%	321	1.0%	211	1.0%
Unknown ethnicity	35576	32.7%	15394	36.8%	3826	28.8%	10807	33.2%	5549	26.3%
BMI (kg/m^2)†	29.3	5.8	29.2	5.9	29.4	6.2	29.2	5.6	29.3	5.7
Current smoking status	23592	21.7%	9555	22.8%	2286	17.2%	7498	23.0%	4253	20.1%
Alcohol (units per week)‡	12.1	14.6	11.2	12.5	15.9	19.7	10.6	13.0	13.5	16.0
IMD score of 5 (most deprived)	12606	11.6%	4217	10.1%	2081	15.7%	3116	9.6%	3192	15.1%
Systolic BP (mmHg)	144.3	5.9	143.7	5.9	143.3	6.1	145.2	5.6	145.0	5.7
Diastolic BP (mmHg)	87.3	5.8	86.9	5.9	87.1	5.9	87.5	5.7	87.7	5.7
Mean CVD risk score (mean±SD)*	7.1%	6.3%	6.1%	5.9%	7.1%	6.7%	7.6%	6.2%	8.3%	6.9%
Statin prescription	13426	12.3%	3927	9.4%	1472	11.1%	4608	14.1%	3419	16.2%
Antiplatelet prescription	6029	5.5%	1404	3.4%	480	3.6%	2567	7.9%	1578	7.5%

†Missing BMI data (n=46,599); ‡Missing alcohol data (n=72,785)

*Based on previous risk score or QRISK2 score estimated from existing or imputed data

For peer review only

Figure legends

Figure 1. Flow diagram showing exclusion of patient records and definition of the final cohort

Figure 2. Primary analysis: Incidence of lifestyle advice and treatment by month.

(a) Lifestyle advice (b) Treatment
Incidence estimates adjusted for age and sex; QOF=Quality and Outcomes Framework; NHS=National Health Service

Figure 3. Post-hoc analysis: Incidence of lifestyle advice and treatment by month.

(a) Lifestyle advice (b) Treatment
Incidence estimates adjusted for age and sex; QOF=Quality and Outcomes Framework; NHS=National Health Service

For peer review only

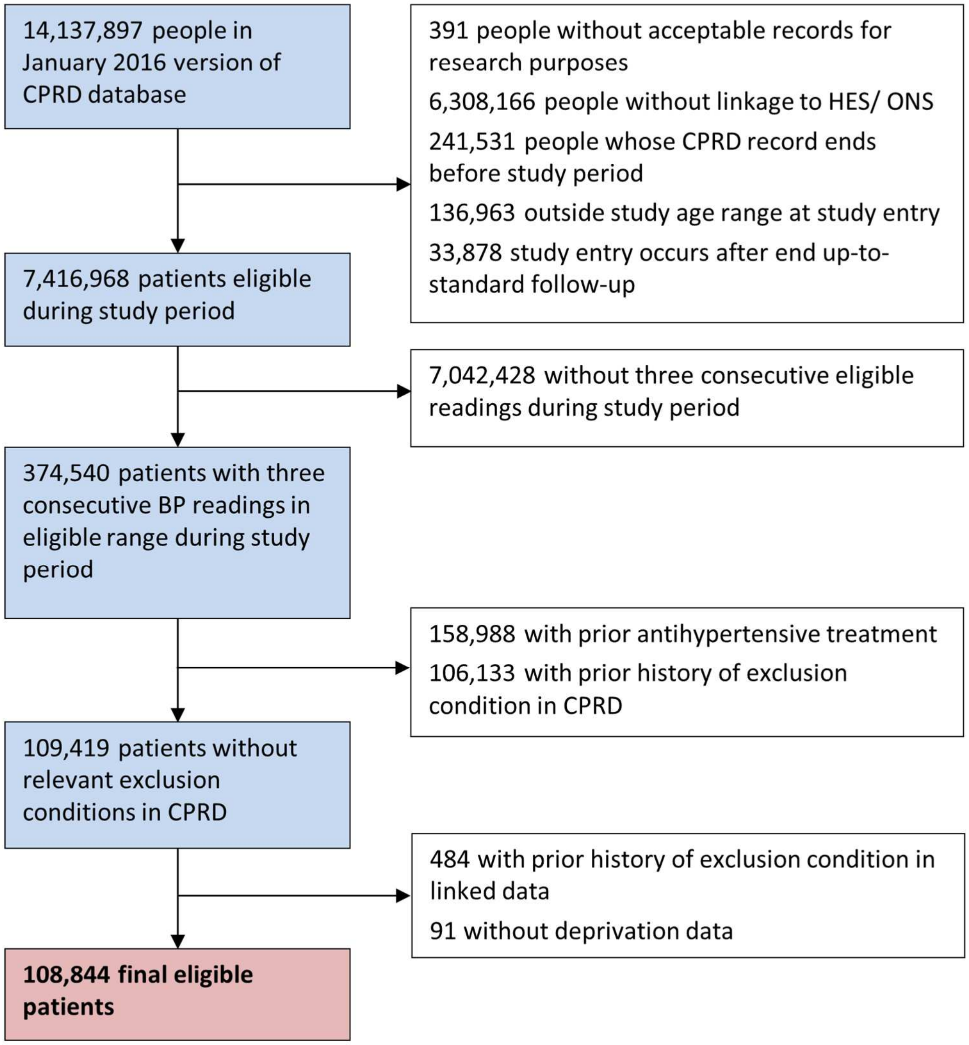

Figure 1

144x160mm (300 x 300 DPI)

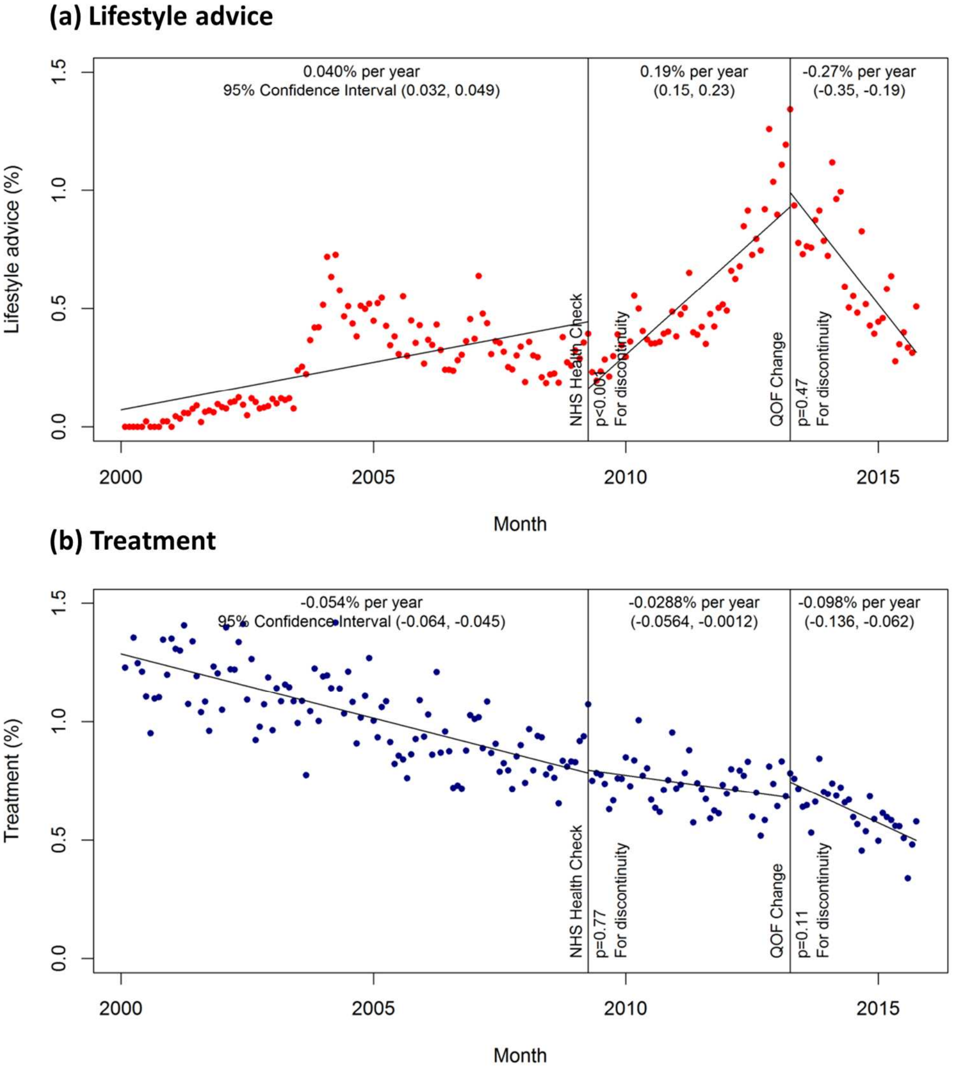

Figure 2

144x160mm (300 x 300 DPI)

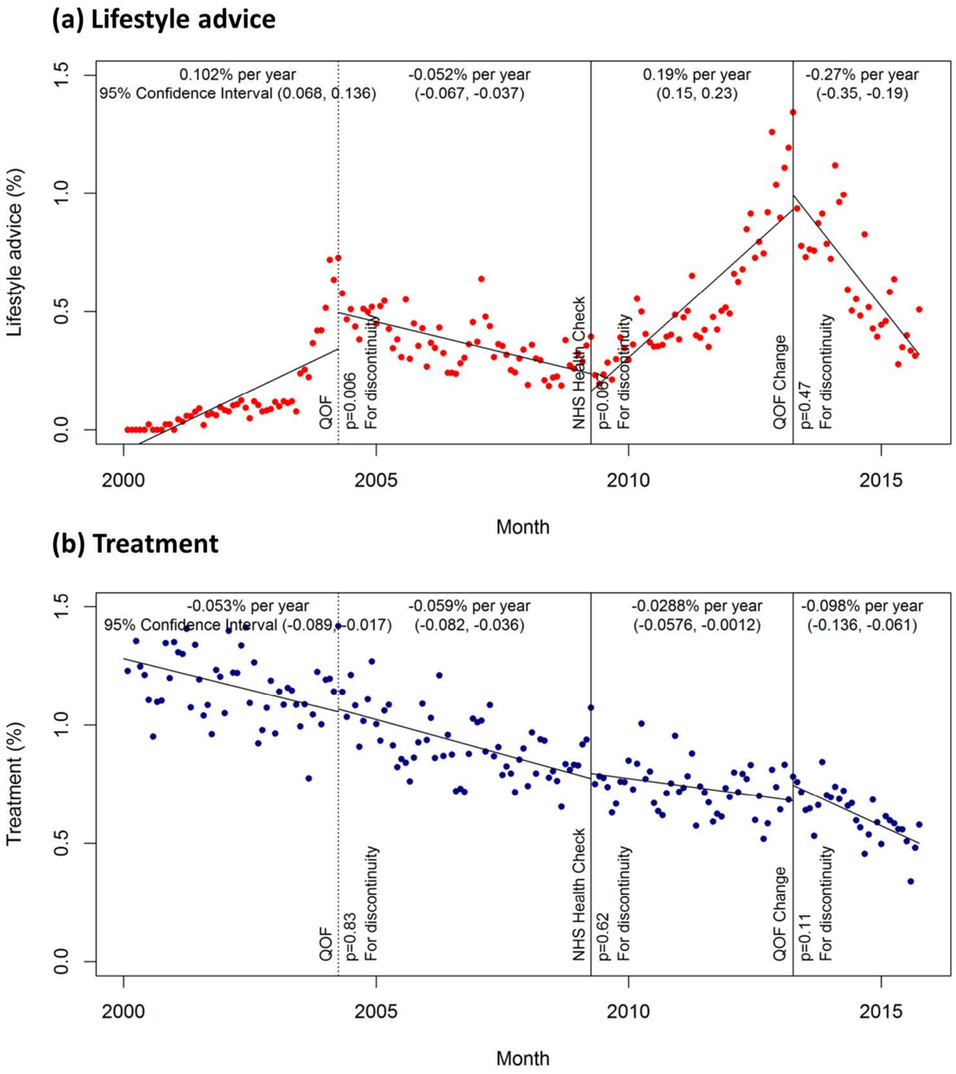

Figure 3

144x160mm (300 x 300 DPI)

Association of guideline and policy changes with incidence of lifestyle advice and treatment for uncomplicated mild hypertension in Primary Care: a longitudinal cohort study in the Clinical Practice Research Datalink

Online appendix

James P Sheppard,[1] Sarah Stevens,[1] Richard Stevens,[1] Jonathan Mant,[2] Una Martin,[3] FD Richard Hobbs[1] and Richard J McManus[1]

[1]University of Oxford, Oxford, UK
[2]University of Cambridge, Cambridge, UK
[3]University of Birmingham, Birmingham, UK

Corresponding author: James P Sheppard
Email: james.sheppard@phc.ox.ac.uk
Telephone: +44 1865 617192
Address: Nuffield Department of Primary Care Health Sciences, Radcliffe Observatory Quarter, University of Oxford, Oxford, OX2 6GG, UK

For peer review only

Contents

1. **eTable 1.** Read codes used to define lifestyle advice outcome in the CPRD
2. **eTable 2.** Read codes used to define antihypertensive treatment outcome in the CPRD
3. ISAC approved protocol for analyses undertaken
4. References

For peer review only

eTable 1. Read codes used to define lifestyle advice outcome in the CPRD

Med code	Read code	Description
18711	67H0.00	Lifestyle advice regarding alcohol
18926	67H1.00	Lifestyle advice regarding smoking
98146	67H7.00	Lifestyle advice regarding diet
98230	67H8.00	Lifestyle advice regarding hypertension
10979	67H2.00	Lifestyle advice regarding exercise
58900	ZC21K00	Advice to change fruit and nut intake
12483	8CAQ.00	Advice about blood glucose control
38021	67IG.00	Oral health advice given
69176	ZC2..11	Advice on diet
69175	ZC2..12	Advice about diet
19414	ZGB..00	Advice relating to treatment and prevention
45933	ZC28.00	Advice to change potassium intake
56711	ZC24.00	Advice to change carbohydrate intake
72645	ZC21700	Advice to change cheese intake
22142	ZG53.00	Advice about weight
54209	ZC2H.00	Advice to change alcohol intake
25041	ZC2CA00	Dietary advice for type II diabetes
102574	67Ip.00	Advice about food intake
19446	ZC26.00	Advice to change fat intake
31922	ZC4..00	Dietary health promotion advice
43187	ZC26200	Advice to change low fat food intake
62415	ZC21c00	Advice change salty food intake
54111	ZC2F.00	Advice to change high energy food intake
42765	ZC2CI00	Dietary advice for lipid disorder
8304	ZC2C711	Dietary advice for weight reduction
9045	ZG23300	Advice on smoking
68419	ZC2E.00	Advice to change convenience food intake
32922	ZC21800	Advice to change egg intake
107619	8CA4M11	High energy diet advice
61863	ZC25.00	Advice to change protein intake
47085	ZC27.00	Advice to change sodium intake
69039	ZC21b00	Advice to change sugar intake
11882	67I9.00	Advice about weight
18759	ZC21.00	Advice to change food intake
24398	ZC23.00	Advice to change dietary fibre intake
59027	ZC26100	Advice to change fatty food intake
72643	ZC21U00	Advice to change table salt intake
7854	ZC2C.00	Dietary advice for disorder
100874	ZC2K.00	Advice to change nutritional product intake
72642	ZC21a00	Advice to change sugary food intake
72640	ZC21900	Advice to change butter intake
50368	ZC2I.00	Advice to change calorie intake
72648	ZC21X00	Advice to change bread intake
7622	8CAL.00	Smoking cessation advice
95114	ZC24300	Advice to change carbohydrate food intake
10642	ZC2C800	Dietary advice for diabetes mellitus
22799	ZC21200	Advice to change dairy food intake
69041	ZC21a11	Advice to change sweet food intake
100099	8IAj.00	Smoking cessation advice declined
38057	ZG22.00	Advice to stop behaviour

For peer review only

72641	ZC21P00	Advice to change vegetable intake
63431	ZC22300	Advice to change coffee intake
66715	ZC2K100	Advice to increase nutritional product intake
11140	ZG23100	Advice on alcohol consumption
57646	ZG11.00	Advice about activity of daily living
54873	ZG1..00	Advice about activity
107333	8CA4Q11	Healthy eating advice
85837	ZG21.00	Advice to avoid behaviour
30246	ZG23.00	Advice relating to health-related behaviour
97163	ZC22200	Advice to change alcoholic drink intake
56677	ZC23100	Advice to change high fibre food intake
31067	ZC2..00	Dietary advice
19735	ZG16100	Advice to exercise
95737	ZC21G00	Advice to change meat intake
101645	ZC23200	Advice to change low fibre food intake
18748	ZG16.00	Advice about exercise
43375	ZC2CO00	Dietary advice for weight loss
101043	8Cd7.00	Advice given about weight management
41042	8CAg.00	Smoking cessation advice provided by community pharmacist
33694	ZC2CJ00	Dietary advice for hyperlipidaemia
95561	ZA11925	Advice on diet
69040	ZC24400	Advice to change refined carbohydrate food intake
68368	ZC21H00	Advice to reduce meat intake
51948	ZG12.00	Advice to undertake activity

For peer review only

eTable 2. Read codes used to define antihypertensive treatment outcome in the CPRD

ACE inhibitors

Product code	GEM code	Description
217	3900007	CAPTOPRIL 4 MG/ML LIQ
59699	21647021	Captopril 5mg/5ml oral solution sugar free
8923	2913007	CAPTOPRIL 100 MG TAB
25051	!1092601	CAPOZIDE
3509	2652007	ENALAPRIL MALEATE 40 MG TAB
27890	!2215902	ENALAPRIL MALEATE
22004	!1100404	CARACE (SPECIAL COMPLIANCE PACK)
24693	!1100403	CARACE (SPECIAL COMPLIANCE PACK)
23382	!1100402	CARACE (SPECIAL COMPLIANCE PACK)
54986	35397020	Perindopril erbumine 8mg/5ml oral suspension
2927	3203007	PERINDOPRIL/TERT-BUTYLAMINE 2 MG TAB
60744	22980021	Perindopril erbumine 8mg / Amlodipine 5mg tablets
56079	47933020	Perindopril tosilate 10mg tablets
57944	47936020	Perindopril tosilate 2.5mg tablets
56157	47939020	Perindopril tosilate 5mg / Indapamide 1.25mg tablets
61117	20667021	Perindopril erbumine 4mg/5ml oral solution
22882	!8505496	RAMIPRIL
18269	52608020	Acepril 12.5mg tablets (Bristol-Myers Squibb Pharmaceuticals Ltd)
3069	52609020	Acepril 25mg tablets (Bristol-Myers Squibb Pharmaceuticals Ltd)
18325	52610020	Acepril 50mg tablets (Bristol-Myers Squibb Pharmaceuticals Ltd)
3310	48565020	Capoten 12.5mg tablets (Bristol-Myers Squibb Pharmaceuticals Ltd)
56509	1375020	Capoten 12.5mg tablets (Dowelhurst Ltd)
1144	48566020	Capoten 25mg tablets (Bristol-Myers Squibb Pharmaceuticals Ltd)
3839	48567020	Capoten 50mg tablets (Bristol-Myers Squibb Pharmaceuticals Ltd)
25998	85701020	Captomex 12.5mg tablets (Actavis UK Ltd)
28820	85702020	Captomex 25mg tablets (Actavis UK Ltd)
24482	85703020	Captomex 50mg tablets (Actavis UK Ltd)
33646	56633020	Captopril 12.5mg Tablet (Generics (UK) Ltd)
34544	56638020	Captopril 12.5mg Tablet (IVAX Pharmaceuticals UK Ltd)
1121	60377020	Captopril 12.5mg tablets
46951	56643020	Captopril 12.5mg tablets (A A H Pharmaceuticals Ltd)
41633	56801020	Captopril 12.5mg tablets (Actavis UK Ltd)
46957	59293020	Captopril 12.5mg tablets (Tillomed Laboratories Ltd)
58195	19216020	Captopril 12.5mg/5ml oral solution
35302	91890020	Captopril 12.5mg/5ml oral suspension
43507	56634020	Captopril 25mg Tablet (Generics (UK) Ltd)
34562	56639020	Captopril 25mg Tablet (IVAX Pharmaceuticals UK Ltd)
34936	56686020	Captopril 25mg Tablet (Lagap)
1143	60378020	Captopril 25mg tablets
43649	56644020	Captopril 25mg tablets (A A H Pharmaceuticals Ltd)
41617	56802020	Captopril 25mg tablets (Actavis UK Ltd)
37655	59733020	Captopril 25mg tablets (Teva UK Ltd)
11641	68130020	Captopril 25mg with Hydrochlorothiazide 12.5mg tablets
52499	19239020	Captopril 25mg/5ml oral solution
59915	21645021	Captopril 25mg/5ml oral solution sugar free
54544	19241020	Captopril 25mg/5ml oral suspension
39512	96425020	Captopril 25mg/5ml oral suspension
52293	20854020	Captopril 2mg capsules

For peer review only

15958	82305020	Captopril 2mg tablets
36742	93652020	Captopril 2mg/5ml oral suspension
17633	87231020	Captopril 3mg/5ml oral solution
34719	56635020	Captopril 50mg Tablet (Generics (UK) Ltd)
34937	56640020	Captopril 50mg Tablet (IVAX Pharmaceuticals UK Ltd)
1807	60379020	Captopril 50mg tablets
41743	59737020	Captopril 50mg tablets (Teva UK Ltd)
10902	68129020	Captopril 50mg with Hydrochlorothiazide 25mg tablets
46851	417021	Captopril 5mg/5ml oral solution
17624	88718020	Captopril 5mg/5ml oral suspension
33336	69419020	Captopril 5mg/5ml Oral suspension (Eldon Laboratories)
44527	98847020	Captopril 5mg/ml oral solution sugar free
43432	92446020	Captopril 6.25mg tablets
28486	89909020	Captopril 6.25mg/5ml oral suspension
45228	91312020	Captopril capsules
56850	84385020	Ecopace 12.5mg tablets (AMCo)
32514	84386020	Ecopace 25mg tablets (AMCo)
15135	69269020	Hydrochlorothiazide with captopril 12.5mg with 25mg Tablet
11133	69268020	Hydrochlorothiazide with captopril 25mg with 50mg Tablet
21943	84490020	Kaplon 12.5mg tablets (Teva UK Ltd)
26995	84491020	Kaplon 25mg tablets (Teva UK Ltd)
32048	84492020	Kaplon 50mg tablets (Teva UK Ltd)
60349	21646021	Noyada 25mg/5ml oral solution (Martindale Pharmaceuticals Ltd)
20849	84462020	Tensopril 12.5mg tablets (Teva UK Ltd)
30039	84463020	Tensopril 25mg tablets (Teva UK Ltd)
23478	84464020	Tensopril 50mg tablets (Teva UK Ltd)
15605	49385020	Cilazapril 250micrograms tablets
23642	50587020	Vascace 0.25mg Tablet (Roche Products Ltd)
12574	49387020	Cilazapril 1mg tablets
12412	54164020	Cilazapril 2.5mg tablets
12411	49386020	Cilazapril 500microgram tablets
13026	54165020	Cilazapril 5mg tablets
16212	50589020	Vascace 1mg tablets (Roche Products Ltd)
16197	55290020	Vascace 2.5mg tablets (Roche Products Ltd)
21053	50588020	Vascace 500microgram tablets (Roche Products Ltd)
16196	55291020	Vascace 5mg tablets (Roche Products Ltd)
42908	61247020	Enalapril 5mg tablets (IVAX Pharmaceuticals UK Ltd)
34768	61253020	Enalapril 20mg tablets (IVAX Pharmaceuticals UK Ltd)
20188	60798020	Enalapril 2.5mg wafer
34453	61301020	Enalapril 20mg tablets (Generics (UK) Ltd)
16708	60797020	Enalapril titration pack
8106	55119020	Innovace 2.5mg tablets (Merck Sharp & Dohme Ltd)
41417	60324020	Enalapril 2.5mg tablets (A A H Pharmaceuticals Ltd)
43563	61391020	Enalapril 2.5mg tablets (Zentiva)
23252	73042020	Pralenal 10 tablets (Opus Pharmaceuticals Ltd)
27871	86081020	Innovace melt 10mg Wafer (Merck Sharp & Dohme Ltd)
8105	58451020	Innovace 20mg tablets (Merck Sharp & Dohme Ltd)
34400	55722020	Enalapril 5mg Tablet (Dowelhurst Ltd)
22439	83449020	Ednyt 20mg Tablet (Dominion Pharma)
34798	61362020	Enalapril 20mg tablets (Sandoz Ltd)
52010	1418020	Enalapril 10mg tablets (Alliance Healthcare (Distribution) Ltd)
53915	61780020	Enalapril 5mg tablets (Dexcel-Pharma Ltd)
34953	61397020	Enalapril 20mg tablets (Zentiva)
31587	86077020	Innovace melt 20mg Wafer (Merck Sharp & Dohme Ltd)

For peer review only

32241	60326020	Enalapril 10mg tablets (A A H Pharmaceuticals Ltd)
46974	61297020	Enalapril 5mg tablets (Generics (UK) Ltd)
60143	38499020	Enalapril 5mg tablets (Medreich Plc)
41694	61246020	Enalapril 2.5mg tablets (IVAX Pharmaceuticals UK Ltd)
22708	77941020	Enalapril 5mg wafer
37087	94156020	Enalapril 5mg/5ml oral suspension
196	60791020	Enalapril 5mg tablets
34712	63374020	Enalapril 20mg tablets (Kent Pharmaceuticals Ltd)
35794	60325020	Enalapril 5mg tablets (A A H Pharmaceuticals Ltd)
50863	19819020	Enalapril 5mg/5ml oral solution (Drug Tariff Special Order)
11197	86080020	Innovace melt 5mg Wafer (Merck Sharp & Dohme Ltd)
29530	86079020	Innovace melt 2.5mg Wafer (Merck Sharp & Dohme Ltd)
43411	61356020	Enalapril 5mg tablets (Sandoz Ltd)
42894	61262020	Enalapril 10mg tablets (Teva UK Ltd)
24041	77943020	Enalapril 20mg wafer
59996	1435020	Enalapril 20mg tablets (Milpharm Ltd)
1904	60796020	Enalapril 20mg tablets
15085	58452020	Innovace Titration pack (Merck Sharp & Dohme Ltd)
8830	55121020	Innovace 10mg tablets (Merck Sharp & Dohme Ltd)
33057	75030020	Ednyt 5mg Tablet (Dominion Pharma)
448	60790020	Enalapril 2.5mg tablets
8800	55120020	Innovace 5mg tablets (Merck Sharp & Dohme Ltd)
61133	1425020	Enalapril 10mg tablets (Phoenix Healthcare Distribution Ltd)
45217	63368020	Enalapril 5mg tablets (Kent Pharmaceuticals Ltd)
28127	61260020	Enalapril 2.5mg tablets (Teva UK Ltd)
31716	61347020	Enalapril 20mg tablets (Actavis UK Ltd)
37080	94154020	Enalapril 5mg/5ml oral solution
42902	61266020	Enalapril 20mg tablets (Teva UK Ltd)
52882	40203020	Enalapril 5mg/5ml oral suspension sugar free
44657	75029020	Ednyt 2.5mg Tablet (Dominion Pharma)
57378	30788020	Enalapril 2mg/5ml oral suspension
1299	60792020	Enalapril 10mg tablets
19208	61344020	Enalapril 10mg tablets (Actavis UK Ltd)
57882	19854020	Enalapril 2.5mg/5ml oral suspension
34952	61298020	Enalapril 10mg tablets (Generics (UK) Ltd)
36753	75031020	Ednyt 10mg Tablet (Dominion Pharma)
53719	1427020	Enalapril 20mg tablets (Alliance Healthcare (Distribution) Ltd)
33078	60335020	Enalapril 20mg tablets (A A H Pharmaceuticals Ltd)
50780	30786020	Enalapril 2mg/5ml oral solution
42723	73041020	Pralenal 5 tablets (Opus Pharmaceuticals Ltd)
58751	19799020	Enalapril 1.25mg/5ml oral suspension
42901	61261020	Enalapril 5mg tablets (Teva UK Ltd)
50334	19817020	Enalapril 4mg/5ml oral suspension
55903	61781020	Enalapril 10mg tablets (Dexcel-Pharma Ltd)
41746	61357020	Enalapril 10mg tablets (Sandoz Ltd)
13755	77942020	Enalapril 10mg wafer
5189	51068020	Enalapril 20mg / Hydrochlorothiazide 12.5mg tablets
1021	51047020	Innozide 20mg/12.5mg tablets (Merck Sharp & Dohme Ltd)
28438	85815020	Triapin 2.5mg/2.5mg modified-release tablets (Sanofi)
17474	78452020	Felodipine 5mg modified-release / Ramipril 5mg tablets
17006	85824020	Triapin 5mg/5mg modified-release tablets (Sanofi)
21162	78451020	Felodipine 2.5mg modified-release / Ramipril 2.5mg tablets
13589	74175020	Staril 20mg tablets (Bristol-Myers Squibb Pharmaceuticals Ltd)
633	74178020	Fosinopril 10mg tablets

For peer review only

4571	74174020	Staril 10mg tablets (Bristol-Myers Squibb Pharmaceuticals Ltd)
5861	74179020	Fosinopril 20mg tablets
18263	59646020	Acezide 25mg/50mg tablets (Bristol-Myers Squibb Pharmaceuticals Ltd)
1520	68133020	Capozide 25mg/50mg tablets (Bristol-Myers Squibb Pharmaceuticals Ltd)
39227	95527020	Capozide LS 12.5mg/25mg tablets (Bristol-Myers Squibb Pharmaceuticals Ltd)
3203	68134020	Capozide LS Tablet (E R Squibb and Sons Ltd)
32166	80831020	Capto-co 25mg+50mg Tablet (IVAX Pharmaceuticals UK Ltd)
11561	84483020	Co-zidocapt 12.5mg/25mg tablets
11351	84482020	Co-zidocapt 25mg/50mg tablets
15031	74293020	Accuretic 12.5mg/10mg tablets (Pfizer Ltd)
15108	74296020	Quinapril 10mg / Hydrochlorothiazide 12.5mg tablets
6408	81266020	Tanatril 5mg tablets (Chiesi Ltd)
12815	81267020	Tanatril 10mg tablets (Chiesi Ltd)
16924	81181020	Imidapril 5mg tablets
18219	81183020	Imidapril 20mg tablets
32560	81268020	Tanatril 20mg tablets (Chiesi Ltd)
12858	81182020	Imidapril 10mg tablets
37978	94277020	Perindopril arginine 5mg / Indapamide 1.25mg tablets
6794	83957020	Perindopril erbumine 4mg / Indapamide 1.25mg tablets
14228	84712020	Coversyl Plus tablets (Servier Laboratories Ltd)
33977	65098020	Lisinopril 10mg tablets (Generics (UK) Ltd)
45337	65231020	Lisinopril 5mg tablets (Actavis UK Ltd)
56505	1458020	Zestril 5mg tablets (Lexon (UK) Ltd)
58294	1461020	Lisinopril 5mg tablets (Accord Healthcare Ltd)
46975	65137020	Lisinopril 5mg tablets (Sandoz Ltd)
30921	64809020	Lisinopril 2.5mg tablets (Teva UK Ltd)
56510	1481020	Zestril 20mg tablets (Sigma Pharmaceuticals Plc)
43413	65206020	Lisinopril 20mg tablets (A A H Pharmaceuticals Ltd)
8268	68567020	Zestril 20mg tablets (AstraZeneca UK Ltd)
3720	68562020	Zestril 2.5mg tablets (AstraZeneca UK Ltd)
41573	65180020	Lisopress 10mg tablets (Teva UK Ltd)
43416	65202020	Lisinopril 10mg tablets (A A H Pharmaceuticals Ltd)
12313	68559020	Carace 20mg tablets (Bristol-Myers Squibb Pharmaceuticals Ltd)
59111	1473020	Lisinopril 20mg tablets (Alliance Healthcare (Distribution) Ltd)
55896	65227020	Lisinopril 2.5mg tablets (Actavis UK Ltd)
45816	71542020	Lisinopril 5mg tablets (Almus Pharmaceuticals Ltd)
41522	65183020	Lisopress 20mg tablets (Teva UK Ltd)
43566	65134020	Lisinopril 2.5mg tablets (Sandoz Ltd)
43418	65199020	Lisinopril 5mg tablets (A A H Pharmaceuticals Ltd)
14387	68555020	Carace 5mg tablets (Bristol-Myers Squibb Pharmaceuticals Ltd)
19198	64822020	Lisinopril 20mg tablets (Teva UK Ltd)
20975	90115020	Lisinopril 7.5mg/5ml oral suspension
65	68572020	Lisinopril 10mg tablets
58461	1437020	Lisinopril 2.5mg tablets (Kent Pharmaceuticals Ltd)
11987	88326020	Lisinopril 5mg/5ml oral solution
54288	69238020	Lisinopril 10mg tablets (Arrow Generics Ltd)
6807	68563020	Zestril 5mg tablets (AstraZeneca UK Ltd)
60010	1462020	Lisinopril 10mg tablets (Kent Pharmaceuticals Ltd)
45300	65236020	Lisinopril 10mg tablets (Actavis UK Ltd)
58258	20373020	Lisinopril 2.5mg/5ml oral suspension
6806	68564020	Zestril 10mg tablets (AstraZeneca UK Ltd)

For peer review only

55639	1472020	Lisinopril 10mg tablets (Accord Healthcare Ltd)
51433	66292020	Lisinopril 20mg tablets (Tillomed Laboratories Ltd)
47159	71545020	Lisinopril 10mg tablets (Almus Pharmaceuticals Ltd)
56279	20371020	Lisinopril 2.5mg/5ml oral solution
34799	65220020	Lisinopril 20mg tablets (Zentiva)
59109	66285020	Lisinopril 5mg tablets (Tillomed Laboratories Ltd)
41538	65173020	Lisopress 2.5mg tablets (Teva UK Ltd)
57048	65217020	Lisinopril 10mg tablets (Zentiva)
41532	65176020	Lisopress 5mg tablets (Teva UK Ltd)
58863	1471020	Lisinopril 10mg tablets (Phoenix Healthcare Distribution Ltd)
58871	14472021	Lisinopril 10mg tablets (Waymade Healthcare Plc)
78	68571020	Lisinopril 5mg tablets
54037	1467020	Lisinopril 10mg tablets (Relonchem Ltd)
16701	68556020	Carace 10mg tablets (Bristol-Myers Squibb Pharmaceuticals Ltd)
60232	65214020	Lisinopril 5mg tablets (Zentiva)
54512	91738020	Lisinopril Oral solution
45324	65239020	Lisinopril 20mg tablets (Actavis UK Ltd)
53551	1484020	Lisinopril 20mg tablets (Phoenix Healthcare Distribution Ltd)
34471	65081020	Lisinopril 5mg tablets (Generics (UK) Ltd)
34696	65102020	Lisinopril 20mg tablets (Generics (UK) Ltd)
10882	68554020	Carace 2.5mg tablets (Bristol-Myers Squibb Pharmaceuticals Ltd)
55456	1448020	Lisinopril 5mg tablets (Alliance Healthcare (Distribution) Ltd)
55002	1486020	Lisinopril 20mg tablets (Accord Healthcare Ltd)
37778	94657020	Lisinopril 5mg/5ml oral suspension
61262	1483020	Lisinopril 20mg tablets (Bristol Laboratories Ltd)
60309	1454020	Lisinopril 5mg tablets (Relonchem Ltd)
54928	1470020	Lisinopril 10mg tablets (Bristol Laboratories Ltd)
19223	64818020	Lisinopril 10mg tablets (Teva UK Ltd)
277	68570020	Lisinopril 2.5mg tablets
58682	66939020	Lisinopril 2.5mg tablets (Generics (UK) Ltd)
58451	71538020	Lisinopril 2.5mg tablets (Almus Pharmaceuticals Ltd)
53820	69229020	Lisinopril 5mg tablets (Arrow Generics Ltd)
69	68575020	Lisinopril 20mg tablets
46979	65143020	Lisinopril 20mg tablets (Sandoz Ltd)
32597	65140020	Lisinopril 10mg tablets (Sandoz Ltd)
43412	65196020	Lisinopril 2.5mg tablets (A A H Pharmaceuticals Ltd)
55588	1482020	Lisinopril 20mg tablets (Sigma Pharmaceuticals Plc)
57588	1446020	Zestril 2.5mg tablets (Mawdsley-Brooks & Company Ltd)
19204	64814020	Lisinopril 5mg tablets (Teva UK Ltd)
54283	20380020	Lisinopril 5mg/5ml oral suspension (Drug Tariff Special Order)
53271	1463020	Lisinopril 10mg tablets (Alliance Healthcare (Distribution) Ltd)
52088	1459020	Lisinopril 5mg tablets (Phoenix Healthcare Distribution Ltd)
60097	65209020	Lisinopril 2.5mg tablets (Zentiva)
38995	95877020	Zestoretic 20 tablets (AstraZeneca UK Ltd)
21231	87820020	Caralpha 20mg/12.5mg tablets (Actavis UK Ltd)
6786	50823020	Lisinopril 10mg / Hydrochlorothiazide 12.5mg tablets
55399	67847020	Lisinopril 20mg / Hydrochlorothiazide 12.5mg tablets (A A H Pharmaceuticals Ltd)
33353	67697020	Lisinopril 20mg / Hydrochlorothiazide 12.5mg tablets (Teva UK Ltd)
56244	1491020	Lisinopril 20mg / Hydrochlorothiazide 12.5mg tablets (Tillomed Laboratories Ltd)
37710	67694020	Lisinopril 10mg / Hydrochlorothiazide 12.5mg tablets (Teva UK Ltd)
39137	95875020	Zestoretic 10 tablets (AstraZeneca UK Ltd)
9764	72035020	Carace 20 Tablet (Bristol-Myers Squibb Pharmaceuticals Ltd)

17655	72036020	Carace 10 Tablet (Bristol-Myers Squibb Pharmaceuticals Ltd)
39147	95901020	Carace 20 Plus tablets (Merck Sharp & Dohme Ltd)
6359	51463020	Zestoretic 10- 10mg+12.5mg Tablet (AstraZeneca UK Ltd)
39242	95903020	Carace 10 Plus tablets (Merck Sharp & Dohme Ltd)
2982	51462020	Zestoretic 20- 20mg+12.5mg Tablet (AstraZeneca UK Ltd)
57539	1503020	Zestoretic 10 tablets (Sigma Pharmaceuticals Plc)
6468	50822020	Lisinopril 20mg / Hydrochlorothiazide 12.5mg tablets
54201	75924020	Lisinopril 20mg / Hydrochlorothiazide 12.5mg tablets (Almus Pharmaceuticals Ltd)
15121	80674020	Moexipril 7.5mg tablets
17120	80675020	Moexipril 15mg tablets
28725	80672020	Perdix 15mg tablets (UCB Pharma Ltd)
28724	80671020	Perdix 7.5mg tablets (UCB Pharma Ltd)
51807	38770020	Coversyl Arginine 5mg tablets (DE Pharmaceuticals)
37930	94699020	Perindopril arginine 5mg tablets
37971	94701020	Perindopril arginine 10mg tablets
38034	94703020	Coversyl Arginine 2.5mg tablets (Servier Laboratories Ltd)
50347	34964020	Coversyl Arginine 5mg tablets (Waymade Healthcare Plc)
37964	94697020	Perindopril arginine 2.5mg tablets
38026	94707020	Coversyl Arginine 10mg tablets (Servier Laboratories Ltd)
37965	94705020	Coversyl Arginine 5mg tablets (Servier Laboratories Ltd)
51258	34961020	Coversyl Arginine Plus 5mg/1.25mg tablets (DE Pharmaceuticals)
37908	94709020	Coversyl Arginine Plus 5mg/1.25mg tablets (Servier Laboratories Ltd)
48214	76375020	Perindopril erbumine 4mg tablets (Actavis UK Ltd)
59972	1543020	Perindopril erbumine 2mg tablets (Alliance Healthcare (Distribution) Ltd)
11983	90557020	Perindopril erbumine 4mg/5ml oral suspension
5800	72569020	Coversyl 4mg tablets (Servier Laboratories Ltd)
61270	45151020	Perindopril erbumine 4mg tablets (Accord Healthcare Ltd)
14960	83699020	Coversyl 8mg tablets (Servier Laboratories Ltd)
59770	15619021	Perindopril erbumine 4mg tablets (Aurobindo Pharma Ltd)
48049	76554020	Perindopril erbumine 2mg tablets (Generics (UK) Ltd)
58874	1550020	Perindopril erbumine 2mg tablets (Somex Pharma)
53058	40612020	Perindopril erbumine 8mg tablets (Sandoz Ltd)
58843	1542020	Perindopril erbumine 2mg tablets (Kent Pharmaceuticals Ltd)
56162	1556020	Perindopril erbumine 4mg tablets (Consilient Health Ltd)
45938	75040020	Perindopril erbumine 8mg tablets (Teva UK Ltd)
56473	1546020	Perindopril erbumine 2mg tablets (Sigma Pharmaceuticals Plc)
56472	1554020	Perindopril erbumine 4mg tablets (Kent Pharmaceuticals Ltd)
60065	1557020	Perindopril erbumine 4mg tablets (Sigma Pharmaceuticals Plc)
97	72573020	Perindopril erbumine 4mg tablets
38510	74776020	Perindopril erbumine 4mg tablets (Apotex UK Ltd)
57801	1558020	Perindopril erbumine 4mg tablets (Glenmark Generics (Europe) Ltd)
5612	72568020	Coversyl 2mg tablets (Servier Laboratories Ltd)
56516	75649020	Perindopril erbumine 2mg tablets (Sandoz Ltd)
50607	92909020	Perindopril arginine 2mg with Indapamide 625 micrograms tablet
6078	79311020	Perindopril erbumine 8mg tablets
49491	1545020	Perindopril erbumine 2mg tablets (Consilient Health Ltd)
57701	76377020	Perindopril erbumine 8mg tablets (Actavis UK Ltd)
61693	15724021	Perindopril erbumine 8mg tablets (Aurobindo Pharma Ltd)
48098	99501020	Perindopril arginine 4mg with Indapamide 1.25mg tablet
33095	73493020	Perindopril erbumine 4mg tablets (A A H Pharmaceuticals Ltd)
43012	91957020	Perindopril erbumine oral solution

For peer review only

35731	73496020	Perindopril erbumine 8mg tablets (A A H Pharmaceuticals Ltd)
54733	9855020	Perindopril erbumine 8mg tablets (Consilient Health Ltd)
59790	39906020	Perindopril erbumine 8mg tablets (Accord Healthcare Ltd)
54899	75033020	Perindopril erbumine 2mg tablets (Teva UK Ltd)
50402	74621020	Perindopril 2mg Tablet (Servier Laboratories Ltd)
48180	75652020	Perindopril erbumine 4mg tablets (Sandoz Ltd)
593	72572020	Perindopril erbumine 2mg tablets
54942	76559020	Perindopril erbumine 8mg tablets (Generics (UK) Ltd)
43813	76373020	Perindopril erbumine 2mg tablets (Actavis UK Ltd)
56508	1552020	Coversyl 4mg tablets (Dowelhurst Ltd)
56506	1541020	Coversyl 2mg tablets (Dowelhurst Ltd)
45319	73490020	Perindopril erbumine 2mg tablets (A A H Pharmaceuticals Ltd)
38285	75036020	Perindopril erbumine 4mg tablets (Teva UK Ltd)
60067	22976021	Perindopril erbumine 4mg / Amlodipine 5mg tablets
60684	22974021	Perindopril erbumine 4mg / Amlodipine 10mg tablets
57333	47942020	Perindopril tosilate 5mg tablets
15096	75604020	Accupro 40mg tablets (Pfizer Ltd)
14478	69845020	Accupro 20mg tablets (Pfizer Ltd)
6765	69848020	Quinapril 5mg tablets
7314	69843020	Accupro 5mg tablets (Pfizer Ltd)
61292	68165020	Quinapril 40mg tablets (Generics (UK) Ltd)
14477	69844020	Accupro 10mg tablets (Pfizer Ltd)
46365	89048020	Quinil 20mg tablets (Tillomed Laboratories Ltd)
3929	69849020	Quinapril 10mg tablets
42285	89050020	Quinil 40mg tablets (Tillomed Laboratories Ltd)
38854	95370020	Quinapril 20mg/5ml oral solution
5159	69850020	Quinapril 20mg tablets
40355	89044020	Quinil 5mg tablets (Tillomed Laboratories Ltd)
9731	75607020	Quinapril 40mg tablets
38899	89046020	Quinil 10mg tablets (Tillomed Laboratories Ltd)
28586	88107020	Lopace 5mg capsules (Discovery Pharmaceuticals Ltd)
52407	1585020	Ramipril 10mg capsules (Kent Pharmaceuticals Ltd)
709	72675020	Ramipril 2.5mg capsules
57658	67312020	Ramipril 1.25mg tablets (A A H Pharmaceuticals Ltd)
47998	68974020	Ramipril 2.5mg capsules (Actavis UK Ltd)
52399	1564020	Ramipril 1.25mg capsules (Kent Pharmaceuticals Ltd)
38308	95107020	Ramipril 2.5/5mg/10mg tablet
34382	67206020	Ramipril 5mg capsules (Zentiva)
654	80174020	Ramipril 2.5/5mg/10mg capsule
45264	68971020	Ramipril 1.25mg capsules (Actavis UK Ltd)
34652	67455020	Ramipril 5mg Capsule (Sovereign Medical Ltd)
29627	88105020	Lopace 2.5mg capsules (Discovery Pharmaceuticals Ltd)
5275	72670020	Tritace 2.5mg capsules (Sanofi)
49164	1588020	Ramipril 10mg capsules (Actavis UK Ltd)
61694	67907020	Ramipril 5mg tablets (Zentiva)
11937	90539020	Ramipril 2.5mg/5ml oral suspension
56855	1590020	Ramipril 10mg capsules (Sigma Pharmaceuticals Plc)
9915	87063020	Tritace 10mg tablets (Sanofi)
34390	67186020	Ramipril 5mg capsules (Genus Pharmaceuticals Ltd)
34412	67140020	Ramipril 5mg capsules (Teva UK Ltd)
56129	1578020	Ramipril 5mg capsules (Kent Pharmaceuticals Ltd)
34657	67209020	Ramipril 10mg capsules (Zentiva)
42081	87446020	Tritace 1.25mg Tablet (Sterwin Medicines)
57073	47143020	Ramipril 1.25mg capsules (Waymade Healthcare Plc)

For peer review only

54620	1575020	Ramipril 2.5mg capsules (Sigma Pharmaceuticals Plc)
52197	1582020	Ramipril 5mg capsules (Sigma Pharmaceuticals Plc)
6364	87059020	Tritace 2.5mg tablets (Sanofi)
56148	10683020	Ramipril 1.25mg tablets (Kent Pharmaceuticals Ltd)
9646	72669020	Tritace 1.25mg capsules (Aventis Pharma)
55299	67297020	Ramipril 1.25mg capsules (A A H Pharmaceuticals Ltd)
82	70737020	Ramipril 10mg capsules
59603	1577020	Ramipril 2.5mg capsules (Phoenix Healthcare Distribution Ltd)
147	72674020	Ramipril 1.25mg capsules
61339	73446020	Ramipril 10mg capsules (Almus Pharmaceuticals Ltd)
34732	67512020	Ramipril 2.5mg Capsule (Dexcel-Pharma Ltd)
61499	73468020	Ramipril 2.5mg tablets (Actavis UK Ltd)
34540	67305020	Ramipril 5mg capsules (A A H Pharmaceuticals Ltd)
45340	68982020	Ramipril 10mg Capsule (Actavis UK Ltd)
50509	20723020	Ramipril 10mg/5ml oral solution
9693	55352020	Tritace 10mg capsules (Sanofi)
57235	74497020	Ramipril 1.25mg tablets (Sandoz Ltd)
80	72676020	Ramipril 5mg capsules
34431	67203020	Ramipril 2.5mg capsules (Zentiva)
55798	47145020	Ramipril 5mg capsules (Waymade Healthcare Plc)
45554	99318020	Ramipril 5mg/5ml oral solution
33811	69974020	Ramipril 2.5mg capsules (Ranbaxy (UK) Ltd)
32857	67125020	Ramipril 1.25mg capsules (Teva UK Ltd)
54298	70068020	Ramipril 2.5mg capsules (Arrow Generics Ltd)
56763	1592020	Ramipril 10mg capsules (Phoenix Healthcare Distribution Ltd)
54941	1579020	Ramipril 5mg capsules (Alliance Healthcare (Distribution) Ltd)
34357	67189020	Ramipril 10mg capsules (Genus Pharmaceuticals Ltd)
5735	72671020	Tritace 5mg capsules (Sanofi)
34893	68264020	Ramipril 10mg Capsule (IVAX Pharmaceuticals UK Ltd)
51714	1572020	Ramipril 2.5mg capsules (Alliance Healthcare (Distribution) Ltd)
40384	67322020	Ramipril 10mg tablets (A A H Pharmaceuticals Ltd)
62039	67901020	Ramipril 1.25mg tablets (Zentiva)
56704	1565020	Ramipril 1.25mg capsules (Alliance Healthcare (Distribution) Ltd)
6288	87053020	Ramipril 5mg tablets
34567	69436020	Ramipril 2.5mg capsules (Generics (UK) Ltd)
34528	67301020	Ramipril 2.5mg capsules (A A H Pharmaceuticals Ltd)
34589	67515020	Ramipril 5mg Capsule (Dexcel-Pharma Ltd)
53612	10700020	Ramipril 10mg tablets (Alliance Healthcare (Distribution) Ltd)
47021	475021	Ramipril 2.5mg/5ml oral solution sugar free
62036	47656020	Ramipril 5mg tablets (Waymade Healthcare Plc)
57864	10698020	Ramipril 5mg tablets (Sigma Pharmaceuticals Plc)
756	87055020	Ramipril 10mg tablets
34943	67309020	Ramipril 10mg capsules (A A H Pharmaceuticals Ltd)
61067	73443020	Ramipril 5mg capsules (Almus Pharmaceuticals Ltd)
34429	69439020	Ramipril 5mg capsules (Generics (UK) Ltd)
56038	39749020	Ramipril 10mg tablets (Pfizer Ltd)
34432	67183020	Ramipril 2.5mg capsules (Genus Pharmaceuticals Ltd)
35007	91818020	Ramipril 10mg/5ml oral suspension
57346	47146020	Ramipril 10mg capsules (Waymade Healthcare Plc)
34877	67460020	Ramipril 10mg Capsule (Sovereign Medical Ltd)
34539	67175020	Ramipril 5mg capsules (Sandoz Ltd)
34698	67770020	Ramipril 1.25mg capsules (Zentiva)
34583	67518020	Ramipril 10mg Capsule (Dexcel-Pharma Ltd)
34490	67129020	Ramipril 2.5mg capsules (Teva UK Ltd)

For peer review only

34505	67170020	Ramipril 2.5mg capsules (Sandoz Ltd)
46890	477021	Ramipril 5mg/5ml oral suspension
61985	67121020	Ramipril 1.25mg tablets (Teva UK Ltd)
32934	88109020	Lopace 10mg capsules (Discovery Pharmaceuticals Ltd)
33894	67144020	Ramipril 10mg capsules (Teva UK Ltd)
761	87049020	Ramipril 1.25mg tablets
34651	69441020	Ramipril 10mg capsules (Generics (UK) Ltd)
59557	1571020	Ramipril 2.5mg capsules (Kent Pharmaceuticals Ltd)
56013	47144020	Ramipril 2.5mg capsules (Waymade Healthcare Plc)
48008	68978020	Ramipril 5mg capsules (Actavis UK Ltd)
56356	1586020	Ramipril 10mg capsules (Alliance Healthcare (Distribution) Ltd)
59788	1591020	Ramipril 10mg capsules (Bristol Laboratories Ltd)
48053	73269020	Ramipril 2.5mg capsules (Almus Pharmaceuticals Ltd)
51701	1583020	Ramipril 5mg capsules (Bristol Laboratories Ltd)
60730	1584020	Ramipril 5mg capsules (Phoenix Healthcare Distribution Ltd)
39355	87452020	Tritace 10mg Tablet (Sterwin Medicines)
6362	87061020	Tritace 5mg tablets (Sanofi)
6261	87057020	Tritace 1.25mg tablets (Sanofi)
6314	87051020	Ramipril 2.5mg tablets
34710	67179020	Ramipril 10mg capsules (Sandoz Ltd)
53621	1576020	Ramipril 2.5mg capsules (Bristol Laboratories Ltd)
56169	70076020	Ramipril 10mg capsules (Arrow Generics Ltd)
31810	68138020	Odrik 1mg capsules (Aventis Pharma)
60757	75136020	Trandolapril 500microgram capsules (Teva UK Ltd)
7419	69080020	Trandolapril 500microgram capsules
16710	71438020	Gopten 500microgram capsules (Abbott Laboratories Ltd)
29130	87608020	Gopten 4mg capsules (Abbott Laboratories Ltd)
4103	69081020	Trandolapril 1mg capsules
9948	87606020	Trandolapril 4mg capsules
54345	10867020	Trandolapril 4mg capsules (Arrow Generics Ltd)
5047	69082020	Trandolapril 2mg capsules
8025	71439020	Gopten 1mg capsules (Abbott Laboratories Ltd)
28902	68139020	Odrik 2mg capsules (Aventis Pharma)
8026	71440020	Gopten 2mg capsules (Abbott Laboratories Ltd)
31307	68137020	Odrik 500microgram capsules (Aventis Pharma)
19690	85865020	Verapamil 180mg modified-release / Trandolapril 2mg capsules
20579	85903020	Tarka modified-release capsules (Abbott Laboratories Ltd)
6200	85253020	Tritace titration pack capsules (Sanofi)
39421	95113020	Tritace titration pack tablets (Sanofi)
24214	6272007	TRITACE 1.25 MG TAB
29964	5959007	TRITACE 2.5 MG TAB
31288	!8505226	TRITACE

For peer review only

Alpha blockers

Product code	GEM code	Description
26238	51808020	Alphavase 1 tablets (Ashbourne Pharmaceuticals Ltd)
13610	51809020	Alphavase 2 tablets (Ashbourne Pharmaceuticals Ltd)
19823	51736020	Alphavase 5 tablets (Ashbourne Pharmaceuticals Ltd)
26237	51807020	Alphavase 500microgram Tablet (Ashbourne Pharmaceuticals Ltd)
11394	48295020	Baratol 25mg Tablet (Shire Pharmaceuticals Ltd)
40256	96646020	Baratol 25mg tablets (Amdipharm Plc)
16198	48296020	Baratol 50mg Tablet (Shire Pharmaceuticals Ltd)
36023	92057020	Cardozin xl 4mg Tablet (Hillcross Pharmaceuticals Ltd)
37243	93699020	Cardozin xl 4mg Tablet (Teva UK Ltd)
46066	99273020	Cardozin XL 4mg tablets (Almus Pharmaceuticals Ltd)
38461	93900020	Cardozin XL 4mg tablets (Arrow Generics Ltd)
4449	69313020	Cardura 1mg tablets (Pfizer Ltd)
4802	69314020	Cardura 2mg tablets (Pfizer Ltd)
8086	69315020	Cardura 4mg Tablet (Pfizer Ltd)
755	85762020	Cardura XL 4mg tablets (Pfizer Ltd)
5618	85763020	Cardura XL 8mg tablets (Pfizer Ltd)
25487	80419020	Cascor 2mg tablets (Ranbaxy (UK) Ltd)
25551	80421020	Cascor 4mg tablets (Ranbaxy (UK) Ltd)
43695	93902020	Colixil XL 4mg tablets (Sandoz Ltd)
8942	48961020	Dibenyline 10mg capsules (Mercury Pharma Group Ltd)
21049	5083007	DIBENYLINE 2.5 MG CAP
29765	5552007	DIBENYLINE 5 MG TAB
7549	58779020	Doxadura 1mg tablets (Discovery Pharmaceuticals Ltd)
7547	83686020	Doxadura 2mg tablets (Discovery Pharmaceuticals Ltd)
10088	83886020	Doxadura 4mg tablets (Discovery Pharmaceuticals Ltd)
35272	93137020	Doxadura XL 4mg tablets (Discovery Pharmaceuticals Ltd)
119	69308020	Doxazosin 1mg tablets
34715	64168020	Doxazosin 1mg tablets (A A H Pharmaceuticals Ltd)
48150	65746020	Doxazosin 1mg tablets (Actavis UK Ltd)
55916	1340020	Doxazosin 1mg tablets (Alliance Healthcare (Distribution) Ltd)
62019	71463020	Doxazosin 1mg tablets (Almus Pharmaceuticals Ltd)
60319	1344020	Doxazosin 1mg tablets (Bristol Laboratories Ltd)
55906	64180020	Doxazosin 1mg tablets (Dexcel-Pharma Ltd)
34601	64033020	Doxazosin 1mg tablets (Generics (UK) Ltd)
41543	64244020	Doxazosin 1mg tablets (IVAX Pharmaceuticals UK Ltd)
59209	65452020	Doxazosin 1mg tablets (Kent Pharmaceuticals Ltd)
45328	64311020	Doxazosin 1mg tablets (Sandoz Ltd)
34342	64067020	Doxazosin 1mg tablets (Teva UK Ltd)
20369	90119020	Doxazosin 1mg/5ml oral suspension
493	69309020	Doxazosin 2mg tablets
34625	64171020	Doxazosin 2mg tablets (A A H Pharmaceuticals Ltd)
56145	65760020	Doxazosin 2mg tablets (Actavis UK Ltd)
50467	1346020	Doxazosin 2mg tablets (Alliance Healthcare (Distribution) Ltd)
61066	1353020	Doxazosin 2mg tablets (Bristol Laboratories Ltd)
45583	64184020	Doxazosin 2mg tablets (Dexcel-Pharma Ltd)
33094	64037020	Doxazosin 2mg tablets (Generics (UK) Ltd)
40891	64251020	Doxazosin 2mg tablets (IVAX Pharmaceuticals UK Ltd)
58276	38497020	Doxazosin 2mg tablets (Medreich Plc)
57074	1352020	Doxazosin 2mg tablets (Sigma Pharmaceuticals Plc)
19193	64070020	Doxazosin 2mg tablets (Teva UK Ltd)

For peer review only

57784	30736020	Doxazosin 2mg/5ml oral suspension
582	84705020	Doxazosin 4mg modified-release tablets
1294	69310020	Doxazosin 4mg tablets
57448	64175020	Doxazosin 4mg tablets (A A H Pharmaceuticals Ltd)
51685	65764020	Doxazosin 4mg tablets (Actavis UK Ltd)
61283	1358020	Doxazosin 4mg tablets (Alliance Healthcare (Distribution) Ltd)
62158	71469020	Doxazosin 4mg tablets (Almus Pharmaceuticals Ltd)
53322	1362020	Doxazosin 4mg tablets (Bristol Laboratories Ltd)
60200	21704021	Doxazosin 4mg tablets (DE Pharmaceuticals)
59862	64188020	Doxazosin 4mg tablets (Dexcel-Pharma Ltd)
34553	64041020	Doxazosin 4mg tablets (Generics (UK) Ltd)
19216	64260020	Doxazosin 4mg tablets (IVAX Pharmaceuticals UK Ltd)
54785	38498020	Doxazosin 4mg tablets (Medreich Plc)
58325	1363020	Doxazosin 4mg tablets (Phoenix Healthcare Distribution Ltd)
45342	64318020	Doxazosin 4mg tablets (Sandoz Ltd)
40678	64073020	Doxazosin 4mg tablets (Teva UK Ltd)
61123	20657021	Doxazosin 4mg/5ml oral solution
35603	93725020	Doxazosin 4mg/5ml oral suspension
5496	84706020	Doxazosin 8mg modified-release tablets
45265	75714020	Doxazosin sr 4mg Tablet (Generics (UK) Ltd)
47807	77113020	Doxazosin xl 4mg Tablet (Hillcross Pharmaceuticals Ltd)
53033	8020	Doxzogen XL 4mg tablets (Generics (UK) Ltd)
19566	!3295102	HYPOVASE
1292	49881020	Hypovase 1mg tablets (Pfizer Ltd)
5183	49882020	Hypovase 2mg tablets (Pfizer Ltd)
4111	49880020	Hypovase 500microgram tablets (Pfizer Ltd)
8198	49886020	Hypovase 5mg Tablet (Pfizer Ltd)
24211	!3296101	HYPOVASE B.D. STARTER PACK
8863	68288020	Hypovase benign prostatic hyperplasia 1mg Tablet (Pfizer Ltd)
23459	68291020	Hypovase benign prostatic hyperplasia 2mg Tablet (Pfizer Ltd)
25047	68287020	Hypovase benign prostatic hyperplasia 500microgram Tablet (Pfizer Ltd)
26693	68286020	Hypovase benign prostatic hyperplasia bd BD Starter pack (Pfizer Ltd)
12518	60761020	Hypovase tablets B.D. starter pack (Pfizer Ltd)
2347	68066020	Hytrin 10mg Tablet (Abbott Laboratories Ltd)
37428	93864020	Hytrin 10mg tablets (AMCo)
8077	68062020	Hytrin 2mg Tablet (Abbott Laboratories Ltd)
36649	93860020	Hytrin 2mg tablets (AMCo)
2346	68063020	Hytrin 5mg Tablet (Abbott Laboratories Ltd)
36780	93862020	Hytrin 5mg tablets (AMCo)
2348	51017020	Hytrin bph 10mg Tablet (Amdipharm Plc)
16201	51572020	Hytrin bph 2mg Tablet (Amdipharm Plc)
5337	51573020	Hytrin bph 5mg Tablet (Amdipharm Plc)
2345	51571020	Hytrin BPH tablets starter pack (AMCo)
8076	72781020	Hytrin tablets starter pack (AMCo)
2816	69659020	Indoramin 20mg tablets
5815	63556020	Indoramin 25mg tablets
9019	63557020	Indoramin 50mg Tablet
45040	99157020	Larbex XL 4mg tablets (Teva UK Ltd)
45584	72200020	Phenoxybenzamine 100mg/2ml solution for infusion ampoules
7759	65687020	Phenoxybenzamine 10mg capsules
40810	94251020	Phenoxybenzamine 10mg/5ml oral suspension
25004	4577007	PHENOXYBENZAMINE HCl 2.5 MG CAP
21466	4576007	PHENOXYBENZAMINE HCl 5 MG CAP
26783	5477007	PHENOXYBENZAMINE HCl 5 MG TAB

For peer review only

54918	93155020	Phenoxybenzamine oral liquid
12545	65694020	Phentolamine 10mg/1ml solution for injection ampoules
60316	54584020	Prazosin 1mg Tablet (Approved Prescription Services Ltd)
591	66154020	Prazosin 1mg tablets
41721	54210020	Prazosin 1mg tablets (A A H Pharmaceuticals Ltd)
46922	54223020	Prazosin 1mg tablets (IVAX Pharmaceuticals UK Ltd)
445	74153020	Prazosin 1mg tablets and Prazosin 500microgram tablets
726	66155020	Prazosin 2mg tablets
41651	54583020	Prazosin 500microgram Tablet (Approved Prescription Services Ltd)
1455	66153020	Prazosin 500microgram tablets
41652	54209020	Prazosin 500microgram tablets (A A H Pharmaceuticals Ltd)
43547	54222020	Prazosin 500microgram tablets (IVAX Pharmaceuticals UK Ltd)
3715	66158020	Prazosin 5mg tablets
55826	54216020	Prazosin 5mg tablets (A A H Pharmaceuticals Ltd)
46526	99138020	Raporsin XL 4mg tablets (Actavis UK Ltd)
23010	65697020	Rogitine 10mg/1ml solution for injection ampoules (Alliance Pharmaceuticals Ltd)
36740	92709020	Slocinx XL 4mg tablets (Zentiva)
4875	68058020	Terazosin 10mg tablets
3470	68054020	Terazosin 1mg tablets
4637	68055020	Terazosin 2mg tablets
57145	65774020	Terazosin 2mg tablets (A A H Pharmaceuticals Ltd)
4694	72784020	Terazosin 2mg tablets and Terazosin 1mg tablets
3924	68056020	Terazosin 5mg tablets
3923	83746020	Terazosin BPH starter pack 7x1mg with 14x2mg with 7x5mg

For peer review only

Angiotensin II receptor antagonists

Product code	GEM code	Description
5117	86113020	Amias 16mg tablets (Takeda UK Ltd)
4155	79053020	Amias 2mg tablets (Takeda UK Ltd)
31072	88790020	Amias 32mg tablets (Takeda UK Ltd)
4685	79054020	Amias 4mg tablets (Takeda UK Ltd)
5013	79055020	Amias 8mg tablets (Takeda UK Ltd)
35189	92767020	Amlodipine 10mg / Valsartan 160mg tablets
35343	92765020	Amlodipine 5mg / Valsartan 160mg tablets
35329	92763020	Amlodipine 5mg / Valsartan 80mg tablets
9196	85710020	Aprovel 150mg tablets (Sanofi)
11348	85711020	Aprovel 300mg tablets (Sanofi)
7338	85709020	Aprovel 75mg tablets (Sanofi)
4741	81321020	Candesartan 16mg tablets
52208	41322020	Candesartan 16mg tablets (A A H Pharmaceuticals Ltd)
57977	41323020	Candesartan 16mg tablets (Alliance Healthcare (Distribution) Ltd)
54414	41324020	Candesartan 16mg tablets (Consilient Health Ltd)
53680	41320020	Candesartan 16mg tablets (Teva UK Ltd)
62035	47163020	Candesartan 16mg tablets (Waymade Healthcare Plc)
529	81323020	Candesartan 2mg tablets
57266	46326020	Candesartan 2mg tablets (Actavis UK Ltd)
59802	41901020	Candesartan 2mg tablets (Teva UK Ltd)
7043	88788020	Candesartan 32mg tablets
54326	41435020	Candesartan 32mg tablets (Teva UK Ltd)
531	81324020	Candesartan 4mg tablets
58646	41311020	Candesartan 4mg tablets (Actavis UK Ltd)
51647	1764020	Candesartan 4mg tablets (Mawdsley-Brooks & Company Ltd)
62140	25450021	Candesartan 4mg tablets (Sandoz Ltd)
53755	41310020	Candesartan 4mg tablets (Teva UK Ltd)
4818	81325020	Candesartan 8mg tablets
51519	41317020	Candesartan 8mg tablets (A A H Pharmaceuticals Ltd)
57273	41316020	Candesartan 8mg tablets (Actavis UK Ltd)
59690	41319020	Candesartan 8mg tablets (Consilient Health Ltd)
51117	1771020	Candesartan 8mg tablets (DE Pharmaceuticals)
50185	41315020	Candesartan 8mg tablets (Teva UK Ltd)
57026	47162020	Candesartan 8mg tablets (Waymade Healthcare Plc)
52559	42156020	Candesartan 8mg tablets (Zentiva)
10316	83981020	CoAprovel 150mg/12.5mg tablets (Sanofi)
11526	83982020	CoAprovel 300mg/12.5mg tablets (Sanofi)
35196	92367020	CoAprovel 300mg/25mg tablets (Sanofi)
6877	87998020	Co-Diovan 160mg/12.5mg tablets (Novartis Pharmaceuticals UK Ltd)
25382	88008020	Co-Diovan 160mg/25mg tablets (Novartis Pharmaceuticals UK Ltd)
764	88424020	Co-Diovan 80mg/12.5mg tablets (Novartis Pharmaceuticals UK Ltd)
52858	10088020	Co-Diovan 80mg/12.5mg tablets (Sigma Pharmaceuticals Plc)
14965	80730020	Cozaar 100mg tablets (Merck Sharp & Dohme Ltd)
52427	10518020	Cozaar 100mg tablets (Necessity Supplies Ltd)
40571	96965020	Cozaar 12.5mg tablets (Merck Sharp & Dohme Ltd)
41232	97245020	Cozaar 2.5mg/ml oral suspension (Merck Sharp & Dohme Ltd)
4226	76329020	Cozaar 25mg tablets (Merck Sharp & Dohme Ltd)
5723	76328020	Cozaar 50mg tablets (Merck Sharp & Dohme Ltd)
37747	94429020	Cozaar-Comp 100mg/12.5mg tablets (Merck Sharp & Dohme Ltd)
21423	89783020	Cozaar-Comp 100mg/25mg tablets (Merck Sharp & Dohme Ltd)
57796	1684020	Cozaar-Comp 50mg/12.5mg tablets (DE Pharmaceuticals)

For peer review only

4540	82246020	Cozaar-Comp 50mg/12.5mg tablets (Merck Sharp & Dohme Ltd)
55160	1673020	Cozaar-Comp 50mg/12.5mg tablets (Sigma Pharmaceuticals Plc)
6518	83507020	Diovan 160mg capsules (Novartis Pharmaceuticals UK Ltd)
45600	98993020	Diovan 160mg Tablet (Novartis Pharmaceuticals UK Ltd)
39199	94445020	Diovan 320mg tablets (Novartis Pharmaceuticals UK Ltd)
11251	83505020	Diovan 40mg capsules (Novartis Pharmaceuticals UK Ltd)
24359	88544020	Diovan 40mg tablets (Novartis Pharmaceuticals UK Ltd)
11252	83506020	Diovan 80mg capsules (Novartis Pharmaceuticals UK Ltd)
6939	84000020	Eprosartan 300mg tablets
13123	84001020	Eprosartan 400mg tablets
12836	84002020	Eprosartan 600mg tablets
35096	92773020	Exforge 10mg/160mg tablets (Novartis Pharmaceuticals UK Ltd)
35697	92771020	Exforge 5mg/160mg tablets (Novartis Pharmaceuticals UK Ltd)
35317	92769020	Exforge 5mg/80mg tablets (Novartis Pharmaceuticals UK Ltd)
60780	38011020	Generic Sevikar HCT 20mg/5mg/12.5mg tablets
60007	38012020	Generic Sevikar HCT 40mg/10mg/12.5mg tablets
38367	94427020	Hydrochlorothiazide with losartan 12.5mg with 100mg Tablet
14738	79035020	Hydrochlorothiazide with losartan 12.5mg with 50mg Tablet
24632	89781020	Hydrochlorothiazide with losartan 25mg with 100mg Tablet
24484	87996020	Hydrochlorothiazide with valsartan 12.5mg with 160mg Tablet
24268	88422020	Hydrochlorothiazide with valsartan 12.5mg with 80mg Tablet
23456	88006020	Hydrochlorothiazide with valsartan 25mg with 160mg Tablet
31160	!8502589	IRBESARTAN
11448	80124020	Irbesartan 150mg / Hydrochlorothiazide 12.5mg tablets
1293	85706020	Irbesartan 150mg tablets
58108	44383020	Irbesartan 150mg tablets (A A H Pharmaceuticals Ltd)
58201	44381020	Irbesartan 150mg tablets (Actavis UK Ltd)
60597	43819020	Irbesartan 150mg tablets (Teva UK Ltd)
11469	80125020	Irbesartan 300mg / Hydrochlorothiazide 12.5mg tablets
35481	92365020	Irbesartan 300mg / Hydrochlorothiazide 25mg tablets
2971	85707020	Irbesartan 300mg tablets
55017	45155020	Irbesartan 300mg tablets (Accord Healthcare Ltd)
59393	47159020	Irbesartan 300mg tablets (Sandoz Ltd)
52972	1748020	Irbesartan 300mg tablets (Sigma Pharmaceuticals Plc)
61781	43823020	Irbesartan 300mg tablets (Teva UK Ltd)
36939	94158020	Irbesartan 300mg/5ml oral suspension
828	85705020	Irbesartan 75mg tablets
37650	94425020	Losartan 100mg / Hydrochlorothiazide 12.5mg tablets
48039	78259020	Losartan 100mg / Hydrochlorothiazide 12.5mg tablets (Teva UK Ltd)
10323	89779020	Losartan 100mg / Hydrochlorothiazide 25mg tablets
52189	10152020	Losartan 100mg / Hydrochlorothiazide 25mg tablets (A A H Pharmaceuticals Ltd)
624	79160020	Losartan 100mg tablets
49588	10510020	Losartan 100mg tablets (A A H Pharmaceuticals Ltd)
61288	10519020	Losartan 100mg tablets (Accord Healthcare Ltd)
54404	77737020	Losartan 100mg tablets (Actavis UK Ltd)
61053	10513020	Losartan 100mg tablets (Alliance Healthcare (Distribution) Ltd)
55446	43882020	Losartan 100mg tablets (Bristol Laboratories Ltd)
60506	77323020	Losartan 100mg tablets (Dexcel-Pharma Ltd)
57028	10512020	Losartan 100mg tablets (Generics (UK) Ltd)
56970	10517020	Losartan 100mg tablets (Pfizer Ltd)
47006	77302020	Losartan 100mg tablets (Teva UK Ltd)
52658	36312020	Losartan 100mg/5ml oral suspension
39944	96963020	Losartan 12.5mg tablets

For peer review only

52886	35998020	Losartan 12.5mg tablets (A A H Pharmaceuticals Ltd)
58967	35999020	Losartan 12.5mg tablets (Alliance Healthcare (Distribution) Ltd)
59340	77317020	Losartan 12.5mg tablets (Dexcel-Pharma Ltd)
40711	97243020	Losartan 2.5mg/ml oral suspension sugar free
520	76333020	Losartan 25mg tablets
50971	1640020	Losartan 25mg tablets (A A H Pharmaceuticals Ltd)
58274	1646020	Losartan 25mg tablets (Accord Healthcare Ltd)
54740	77731020	Losartan 25mg tablets (Actavis UK Ltd)
61495	14777021	Losartan 25mg tablets (Aptil Pharma Ltd)
51186	1643020	Losartan 25mg tablets (Arrow Generics Ltd)
58649	43813020	Losartan 25mg tablets (Bristol Laboratories Ltd)
48398	77320020	Losartan 25mg tablets (Dexcel-Pharma Ltd)
49492	1641020	Losartan 25mg tablets (Generics (UK) Ltd)
55718	1644020	Losartan 25mg tablets (Phoenix Healthcare Distribution Ltd)
59271	40515020	Losartan 25mg tablets (Sandoz Ltd)
59086	41899020	Losartan 25mg tablets (Wockhardt UK Ltd)
61754	35460020	Losartan 25mg/5ml oral suspension
6437	79786020	Losartan 50mg / Hydrochlorothiazide 12.5mg tablets
56975	1677020	Losartan 50mg / Hydrochlorothiazide 12.5mg tablets (A A H Pharmaceuticals Ltd)
56204	77510020	Losartan 50mg / Hydrochlorothiazide 12.5mg tablets (Actavis UK Ltd)
1780	76332020	Losartan 50mg tablets
56104	1658020	Losartan 50mg tablets (A A H Pharmaceuticals Ltd)
54049	1669020	Losartan 50mg tablets (Accord Healthcare Ltd)
51601	77734020	Losartan 50mg tablets (Actavis UK Ltd)
54735	1661020	Losartan 50mg tablets (Alliance Healthcare (Distribution) Ltd)
59750	14778021	Losartan 50mg tablets (Aptil Pharma Ltd)
54843	77349020	Losartan 50mg tablets (Dexcel-Pharma Ltd)
55296	1660020	Losartan 50mg tablets (Generics (UK) Ltd)
59351	1666020	Losartan 50mg tablets (Pfizer Ltd)
54057	77299020	Losartan 50mg tablets (Teva UK Ltd)
52659	35425020	Losartan 50mg/5ml oral solution
59903	35427020	Losartan 50mg/5ml oral suspension
17686	81476020	Micardis 20mg tablets (Boehringer Ingelheim Ltd)
13821	81474020	Micardis 40mg tablets (Boehringer Ingelheim Ltd)
17545	81475020	Micardis 80mg tablets (Boehringer Ingelheim Ltd)
18202	81233020	MicardisPlus 40mg/12.5mg tablets (Boehringer Ingelheim Ltd)
17689	49280020	MicardisPlus 80mg/12.5mg tablets (Boehringer Ingelheim Ltd)
38889	95336020	MicardisPlus 80mg/25mg tablets (Boehringer Ingelheim Ltd)
6217	78179020	Olmesartan medoxomil 10mg tablets
39786	96351020	Olmesartan medoxomil 10mg/5ml oral suspension
40316	96923020	Olmesartan medoxomil 20mg / Amlodipine 5mg tablets
18200	90629020	Olmesartan medoxomil 20mg / Hydrochlorothiazide 12.5mg tablets
18903	90631020	Olmesartan medoxomil 20mg / Hydrochlorothiazide 25mg tablets
6285	78212020	Olmesartan medoxomil 20mg tablets
40668	96927020	Olmesartan medoxomil 40mg / Amlodipine 10mg tablets
40639	96925020	Olmesartan medoxomil 40mg / Amlodipine 5mg tablets
43322	98337020	Olmesartan medoxomil 40mg / Hydrochlorothiazide 12.5mg tablets
6351	78176020	Olmesartan medoxomil 40mg tablets
14983	78194020	Olmetec 10mg tablets (Daiichi Sankyo UK Ltd)
18910	78191020	Olmetec 20mg tablets (Daiichi Sankyo UK Ltd)
20117	57407020	Olmetec 40mg tablets (Daiichi Sankyo UK Ltd)
29634	90635020	Olmetec Plus 20mg/12.5mg tablets (Daiichi Sankyo UK Ltd)
27520	90637020	Olmetec Plus 20mg/25mg tablets (Daiichi Sankyo UK Ltd)

For peer review only

43915	98339020	Olmetec Plus 40mg/12.5mg tablets (Daiichi Sankyo UK Ltd)
39984	96929020	Sevikar 20mg/5mg tablets (Daiichi Sankyo UK Ltd)
41203	96933020	Sevikar 40mg/10mg tablets (Daiichi Sankyo UK Ltd)
41205	96931020	Sevikar 40mg/5mg tablets (Daiichi Sankyo UK Ltd)
46355	99779020	Sevikar HCT 20mg/5mg/12.5mg tablets (Daiichi Sankyo UK Ltd)
47616	99785020	Sevikar HCT 40mg/10mg/12.5mg tablets (Daiichi Sankyo UK Ltd)
53220	99791020	Sevikar HCT 40mg/10mg/25mg tablets (Daiichi Sankyo UK Ltd)
47573	99783020	Sevikar HCT 40mg/5mg/12.5mg tablets (Daiichi Sankyo UK Ltd)
47727	99789020	Sevikar HCT 40mg/5mg/25mg tablets (Daiichi Sankyo UK Ltd)
6243	74674020	Telmisartan 20mg tablets
61177	33020	Telmisartan 20mg tablets (Sigma Pharmaceuticals Plc)
14870	81237020	Telmisartan 40mg / Hydrochlorothiazide 12.5mg tablets
5988	74672020	Telmisartan 40mg tablets
16161	58831020	Telmisartan 80mg / Hydrochlorothiazide 12.5mg tablets
38459	95334020	Telmisartan 80mg / Hydrochlorothiazide 25mg tablets
12874	74673020	Telmisartan 80mg tablets
9745	84004020	Teveten 300mg tablets (Abbott Healthcare Products Ltd)
16285	84005020	Teveten 400mg tablets (Abbott Healthcare Products Ltd)
16371	84006020	Teveten 600mg tablets (Abbott Healthcare Products Ltd)
11864	87994020	Valsartan 160mg / Hydrochlorothiazide 12.5mg tablets
14283	88002020	Valsartan 160mg / Hydrochlorothiazide 25mg tablets
4645	83503020	Valsartan 160mg capsules
55187	39798020	Valsartan 160mg capsules (Arrow Generics Ltd)
53833	38960020	Valsartan 160mg capsules (Generics (UK) Ltd)
61442	1715020	Valsartan 160mg capsules (Teva UK Ltd)
55821	78684020	Valsartan 160mg capsules (Teva UK Ltd)
60076	47156020	Valsartan 160mg capsules (Waymade Healthcare Plc)
44778	91979020	Valsartan 160mg tablets
37573	94443020	Valsartan 320mg tablets
59029	99475020	Valsartan 3mg/1ml oral solution
575	83501020	Valsartan 40mg capsules
58669	1689020	Valsartan 40mg capsules (Teva UK Ltd)
54726	78678020	Valsartan 40mg capsules (Teva UK Ltd)
14943	88542020	Valsartan 40mg tablets
16060	88420020	Valsartan 80mg / Hydrochlorothiazide 12.5mg tablets
3222	83502020	Valsartan 80mg capsules
59448	38828020	Valsartan 80mg capsules (A A H Pharmaceuticals Ltd)
58910	1699020	Valsartan 80mg capsules (Sigma Pharmaceuticals Plc)
38395	91976020	Valsartan 80mg tablets

For peer review only

Beta blockers

Product code	GEM code	Description
26788	4486007	PROPRANOLOL 2.5 MG ELI
23604	!6000601	PROPRANOLOL S/R
29803	4484007	PROPRANOLOL 3 MG ELI
19003	4394007	SPIRONOLACTONE/PROPRANOLOL 50 MG TAB
4021	6228007	PROPRANOLOL 20 MG TAB
26105	5680007	PROPRANOLOL paed 4 MG TAB
46493	4483007	PROPRANOLOL 15 MG SYR
32470	4480007	PROPRANOLOL 1 MG LIQ
25818	5678007	PROPRANOLOL 30 MG SUS
22634	4485007	PROPRANOLOL 10 MG SUS
27036	5679007	PROPRANOLOL POWDERS 5 MG POW
44310	!3431105	INDERAL
25052	!3121601	HALF-INDERAL LA
61573	30311020	Atenolol 25mg/5ml oral solution
8765	5317007	ATENOLOL/CHLORTHALIDONE 50 MG TAB
24677	!0394102	ATENOLOL
25037	!0394103	ATENOLOL
26807	!7046101	TENORETIC
52611	31454020	Bisoprolol 10mg/5ml oral solution
18722	!8504177	CARVEDILOL
22796	!8504114	CARVEDILOL 3.125 MG
7491	6201007	LABETALOL TAB
24520	4879007	HYDROCHLOROTHIAZIDE /METOPROLOL TARTRATE 25 MG TAB
28493	3289007	METOPROLOL FUMARATE 95 MG TAB
22151	!4529101	METOPROLOL 100MG/CHLORTHALIDONE 12.5MG
41892	!4530101	METOPROLOL 100MG/HYDROCHLOROTHIAZ.12.5MG
17876	3667007	METOPROLOL FUMARATE 190 MG TAB
41897	!1385102	CO-BETALOC
24378	!0681102	BETALOC S.A. (CALENDAR PACK)
20813	!0681101	BETALOC S.A.
16669	2319007	LOPRESOR SR 200 MG TAB
27086	6704007	NADOLOL 80MG/BENDROFLUAZIDE 5MG MG TAB
8113	59925020	Acebutolol 200mg capsules
8023	52703020	Sectral 400mg tablets (Sanofi)
12296	52701020	Sectral 100mg capsules (Sanofi)
8172	59924020	Acebutolol 100mg capsules
8555	52702020	Sectral 200mg capsules (Sanofi)
7620	59926020	Acebutolol 400mg tablets
45309	71703020	Acebutolol 400mg tablets (A A H Pharmaceuticals Ltd)
14126	59935020	Acebutolol 200mg / Hydrochlorothiazide 12.5mg tablets
8189	51499020	Secadrex 200mg/12.5mg tablets (Sanofi)
7429	51862020	Tenormin 5mg/10ml solution for injection ampoules (AstraZeneca UK Ltd)
197	60300020	Atenolol 5mg/10ml solution for injection ampoules
34754	51439020	Atenolol 100mg tablets (Sandoz Ltd)
53826	1006020	Atenolol 25mg tablets (Boston Healthcare Ltd)
4983	68844020	Atenolol with amiloride and hydrochlorothiazide capsules
44858	56835020	Atenolol 25mg tablets (Actavis UK Ltd)
34265	51438020	Atenolol 50mg tablets (Sandoz Ltd)
51998	1011020	Atenolol 25mg tablets (Co-Pharma Ltd)
34976	59302020	Atenolol 25mg tablets (Tillomed Laboratories Ltd)

53204	973020	Atenolol 50mg tablets (Alliance Healthcare (Distribution) Ltd)
33850	56836020	Atenolol 50mg tablets (Actavis UK Ltd)
19172	57790020	Atenolol 25mg tablets (IVAX Pharmaceuticals UK Ltd)
13394	51861020	Tenormin 25mg/5ml syrup (AstraZeneca UK Ltd)
21133	86681020	Atenamin 50mg Tablet (OPD Pharm)
30636	72687020	Vasaten 50mg Tablet (Shire Pharmaceuticals Ltd)
46908	56095020	Atenolol 100mg tablets (Kent Pharmaceuticals Ltd)
33085	51023020	Atenolol 100mg tablets (A A H Pharmaceuticals Ltd)
59982	1012020	Atenolol 25mg tablets (Accord Healthcare Ltd)
34882	52909020	Atenolol 50mg Tablet (Berk Pharmaceuticals Ltd)
34365	53708020	Atenolol 50mg tablets (Teva UK Ltd)
31536	56093020	Atenolol 25mg tablets (Kent Pharmaceuticals Ltd)
59695	980020	Atenolol 50mg tablets (Boston Healthcare Ltd)
53414	985020	Atenolol 50mg tablets (Accord Healthcare Ltd)
52500	70253020	Atenolol 50mg tablets (Almus Pharmaceuticals Ltd)
34585	51440020	Atenolol 25mg tablets (Sandoz Ltd)
20502	72697020	Atenix 100 tablets (Ashbourne Pharmaceuticals Ltd)
29398	86682020	Atenamin 100mg Tablet (OPD Pharm)
2587	51860020	Tenormin 100mg tablets (AstraZeneca UK Ltd)
5	60153020	Atenolol 50mg tablets
20728	86680020	Atenamin 25mg Tablet (OPD Pharm)
54542	1008020	Atenolol 25mg tablets (Zanza Laboratories Ltd)
33092	51022020	Atenolol 50mg tablets (A A H Pharmaceuticals Ltd)
19191	53707020	Atenolol 100mg tablets (Teva UK Ltd)
53802	1007020	Atenolol 25mg tablets (Sigma Pharmaceuticals Plc)
33184	52914020	Atenolol 100mg tablets (Wockhardt UK Ltd)
29368	53709020	Atenolol 25mg tablets (Teva UK Ltd)
57817	74385020	Atenolol 50mg tablets (Zentiva)
19182	55394020	Atenolol 50mg tablets (IVAX Pharmaceuticals UK Ltd)
17322	72698020	Atenix 25 tablets (Ashbourne Pharmaceuticals Ltd)
3526	69296020	Amiloride with atenolol with hydrochlorothiazide capsules
55778	983020	Atenolol 50mg tablets (Phoenix Healthcare Distribution Ltd)
54752	984020	Atenolol 50mg tablets (Co-Pharma Ltd)
34695	56094020	Atenolol 50mg tablets (Kent Pharmaceuticals Ltd)
10191	72696020	Atenix 50 tablets (Ashbourne Pharmaceuticals Ltd)
28177	68841020	Hydrochlorothiazide with atenolol and amiloride Capsule
26211	68815020	Antipressan 25mg tablets (Teva UK Ltd)
24	60152020	Atenolol 100mg tablets
47870	70250020	Atenolol 25mg tablets (Almus Pharmaceuticals Ltd)
24195	68814020	Antipressan 100mg tablets (Teva UK Ltd)
49953	1009020	Atenolol 25mg tablets (Bristol Laboratories Ltd)
24191	68813020	Antipressan 50mg tablets (Teva UK Ltd)
52310	39793020	Atenolol 25mg tablets (Crescent Pharma Ltd)
34492	60231020	Atenolol 25mg tablets (Generics (UK) Ltd)
2590	72526020	Tenormin 25mg tablets (AstraZeneca UK Ltd)
26	67920020	Atenolol 25mg tablets
36261	59303020	Atenolol 50mg tablets (Tillomed Laboratories Ltd)
6066	67919020	Atenolol 25mg/5ml oral solution sugar free
34575	52915020	Atenolol 25mg tablets (Wockhardt UK Ltd)
18950	72693020	Totamol 25mg Tablet (C P Pharmaceuticals Ltd)
33657	51024020	Atenolol 25mg tablets (A A H Pharmaceuticals Ltd)
34443	52913020	Atenolol 50mg tablets (Wockhardt UK Ltd)
31934	55395020	Atenolol 100mg tablets (IVAX Pharmaceuticals UK Ltd)
33650	60232020	Atenolol 50mg tablets (Generics (UK) Ltd)

For peer review only

50702	998020	Atenolol 25mg tablets (Alliance Healthcare (Distribution) Ltd)
15176	72691020	Totamol 50mg Tablet (C P Pharmaceuticals Ltd)
33079	60233020	Atenolol 100mg tablets (Generics (UK) Ltd)
2432	54818020	Tenormin LS 50mg tablets (AstraZeneca UK Ltd)
15730	72692020	Totamol 100mg Tablet (C P Pharmaceuticals Ltd)
51643	1013020	Atenolol 25mg/5ml oral solution sugar free (Alliance Healthcare (Distribution) Ltd)
46931	56837020	Atenolol 100mg tablets (Actavis UK Ltd)
56445	77037020	Atenolol 25mg/5ml oral solution sugar free (A A H Pharmaceuticals Ltd)
53215	982020	Atenolol 50mg tablets (Bristol Laboratories Ltd)
7543	56162020	Kalten capsules (M & A Pharmachem Ltd)
9178	83986020	Atenolol 25mg / Bendroflumethiazide 1.25mg capsules
18743	83989020	Tenben 25mg/1.25mg capsules (Galen Ltd)
1288	51857020	Tenoret 50mg/12.5mg tablets (AstraZeneca UK Ltd)
5721	73883020	Co-tenidone 100mg/25mg tablets
34449	60352020	Co-tenidone 50mg/12.5mg tablets (Generics (UK) Ltd)
46952	56911020	Co-tenidone 100mg/25mg tablets (Actavis UK Ltd)
31470	57164020	Tenchlor 50mg/12.5mg tablets (Teva UK Ltd)
24280	80150020	Totaretic 100mg+25mg Tablet (C P Pharmaceuticals Ltd)
31708	56910020	Co-tenidone 50mg/12.5mg tablets (Actavis UK Ltd)
1124	54815020	Tenoretic 100mg/25mg tablets (AstraZeneca UK Ltd)
32094	53521020	Co-tenidone 50mg/12.5mg tablets (A A H Pharmaceuticals Ltd)
26741	80149020	Totaretic 50mg+12.5mg Tablet (C P Pharmaceuticals Ltd)
26248	57165020	Tenchlor 100mg/25mg tablets (Teva UK Ltd)
34034	55247020	Co-tenidone 50mg/12.5mg tablets (IVAX Pharmaceuticals UK Ltd)
34825	54653020	Co-tenidone 50mg/12.5mg tablets (Teva UK Ltd)
41572	54654020	Co-tenidone 100mg/25mg tablets (Teva UK Ltd)
34899	53522020	Co-tenidone 100mg/25mg tablets (A A H Pharmaceuticals Ltd)
13526	63763020	Atenix Co 100 tablets (Ashbourne Pharmaceuticals Ltd)
37725	60353020	Co-tenidone 100mg/25mg tablets (Generics (UK) Ltd)
34012	55246020	Co-tenidone 100mg/25mg tablets (IVAX Pharmaceuticals UK Ltd)
9783	73882020	Co-tenidone 50mg/12.5mg tablets
21873	63762020	Atenix Co 50 tablets (Ashbourne Pharmaceuticals Ltd)
4542	68847020	Atenolol 50mg / Nifedipine 20mg modified-release capsules
52728	1077020	Beta-Adalat modified-release capsules (Lexon (UK) Ltd)
61719	1072020	Beta-Adalat modified-release capsules (Waymade Healthcare Plc)
8642	68652020	Tenif 50mg/20mg modified-release capsules (AstraZeneca UK Ltd)
1684	68629020	Beta-Adalat modified-release capsules (Bayer Plc)
43251	75323020	Bisoprolol 1.25mg tablets (Generics (UK) Ltd)
58763	47116020	Bisoprolol 2.5mg tablets (Waymade Healthcare Plc)
33839	62689020	Bisoprolol 10mg tablets (Actavis UK Ltd)
17615	83190020	Cardicor 5mg tablets (Merck Serono Ltd)
33909	90617020	Congescor 1.25mg tablets (Tillomed Laboratories Ltd)
19178	62996020	Bisoprolol 10mg tablets (Ranbaxy (UK) Ltd)
56240	40506020	Bisoprolol 3.75mg tablets (Sandoz Ltd)
472	68438020	Bisoprolol 5mg tablets
55791	40507020	Bisoprolol 3.75mg tablets (Actavis UK Ltd)
58498	15612021	Bisoprolol 2.5mg tablets (Medreich Plc)
52686	31458020	Bisoprolol 2.5mg/5ml oral solution
822	91125020	Bisoprolol 1.5mg/5ml oral suspension
52548	78457020	Bisoprolol 1.25mg tablets (Almus Pharmaceuticals Ltd)
3588	68353020	Monocor 5mg tablets (Wyeth Pharmaceuticals)
594	83178020	Bisoprolol 2.5mg tablets

For peer review only

53664	40505020	Bisoprolol 2.5mg tablets (Sandoz Ltd)
61651	1149020	Bisoprolol 7.5mg tablets (Almus Pharmaceuticals Ltd)
10892	68369020	Emcor 10mg tablets (Merck Serono Ltd)
38991	75624020	Bisoprolol 7.5mg tablets (A A H Pharmaceuticals Ltd)
24083	62681020	Bisoprolol 5mg tablets (Teva UK Ltd)
50514	1122020	Bisoprolol 2.5mg tablets (Chanelle Medical UK Ltd)
34821	62677020	Bisoprolol 10mg tablets (Generics (UK) Ltd)
47041	75328020	Bisoprolol 2.5mg tablets (Generics (UK) Ltd)
18185	83191020	Cardicor 7.5mg tablets (Merck Serono Ltd)
53885	74139020	Bisoprolol 1.25mg tablets (A A H Pharmaceuticals Ltd)
59148	41897020	Bisoprolol 2.5mg tablets (Zentiva)
58974	1116020	Bisoprolol 2.5mg tablets (Alliance Healthcare (Distribution) Ltd)
19200	63135020	Bisoprolol 5mg tablets (IVAX Pharmaceuticals UK Ltd)
61340	38485020	Bisoprolol 5mg tablets (DE Pharmaceuticals)
55929	45144020	Bisoprolol 5mg tablets (Accord Healthcare Ltd)
59495	43811020	Bisoprolol 1.25mg tablets (Teva UK Ltd)
61564	47117020	Bisoprolol 3.75mg tablets (Waymade Healthcare Plc)
37118	73449020	Bisoprolol 2.5mg tablets (A A H Pharmaceuticals Ltd)
7553	90711020	Bisoprolol 5mg/5ml oral suspension
39846	84559020	Vivacor 5mg tablets (Lexon (UK) Ltd)
50224	41307020	Congescor 2.5mg tablets (Teva UK Ltd)
55298	1097020	Bisoprolol 10mg tablets (Sigma Pharmaceuticals Plc)
57176	45145020	Bisoprolol 10mg tablets (Accord Healthcare Ltd)
57934	67815020	Bisoprolol 5mg tablets (Sandoz Ltd)
32552	90619020	Congescor 2.5mg tablets (Tillomed Laboratories Ltd)
599	68440020	Bisoprolol 1.25mg tablets
57626	19127020	Bisoprolol 1.25mg/5ml oral solution
1290	68439020	Bisoprolol 10mg tablets
59969	1090020	Bisoprolol 5mg tablets (Almus Pharmaceuticals Ltd)
59037	62942020	Bisoprolol 5mg tablets (A A H Pharmaceuticals Ltd)
43564	62726020	Bisoprolol 5mg Tablet (PLIVA Pharma Ltd)
4771	68375020	Emcor LS 5mg tablets (Merck Serono Ltd)
54479	1103020	Bisoprolol 1.25mg tablets (Alliance Healthcare (Distribution) Ltd)
21905	86502020	Bipranix 10mg tablets (Ashbourne Pharmaceuticals Ltd)
14058	83182020	Cardicor 1.25mg tablets (Merck Serono Ltd)
58982	38487020	Bisoprolol 10mg tablets (Medreich Plc)
58109	19125020	Bisoprolol 1.25mg/5ml oral suspension
7091	83179020	Bisoprolol 3.75mg tablets
52635	1082020	Bisoprolol 5mg tablets (Alliance Healthcare (Distribution) Ltd)
39646	96562020	Bisoprolol 0.625mg/5ml oral solution
41591	62682020	Bisoprolol 10mg tablets (Teva UK Ltd)
44000	98730020	Bisoprolol 2.5mg/5ml oral suspension
14030	83183020	Cardicor 2.5mg tablets (Merck Serono Ltd)
19858	83192020	Cardicor 10mg tablets (Merck Serono Ltd)
50403	73571020	Bisoprolol 1.25mg Tablet (Teva UK Ltd)
5713	83180020	Bisoprolol 7.5mg tablets
60761	15611021	Bisoprolol 1.25mg tablets (Medreich Plc)
60896	38486020	Bisoprolol 5mg tablets (Medreich Plc)
61115	19119020	Bisoprolol 5mg/5ml oral solution
19853	83184020	Cardicor 3.75mg tablets (Merck Serono Ltd)
57023	1125020	Bisoprolol 2.5mg tablets (Almus Pharmaceuticals Ltd)
50300	41306020	Congescor 1.25mg tablets (Teva UK Ltd)
51528	40504020	Bisoprolol 1.25mg tablets (Actavis UK Ltd)
53334	62943020	Bisoprolol 10mg tablets (A A H Pharmaceuticals Ltd)

For peer review only

56486	1094020	Monocor 10mg tablets (Dowelhurst Ltd)
21966	86500020	Bipranix 5mg tablets (Ashbourne Pharmaceuticals Ltd)
58973	63965020	Bisoprolol 10mg tablets (Niche Generics Ltd)
37837	73574020	Bisoprolol 2.5mg Tablet (Teva UK Ltd)
53916	78463020	Bisoprolol 2.5mg tablets (Almus Pharmaceuticals Ltd)
32630	77372020	Vivacor 10mg tablets (Lexon (UK) Ltd)
34963	62688020	Bisoprolol 5mg tablets (Actavis UK Ltd)
57578	1123020	Cardicor 2.5mg tablets (Necessity Supplies Ltd)
60502	38490020	Bisoprolol 3.75mg tablets (DE Pharmaceuticals)
56459	45146020	Bisoprolol 2.5mg tablets (Accord Healthcare Ltd)
32114	62676020	Bisoprolol 5mg tablets (Generics (UK) Ltd)
56768	77972020	Bisoprolol 2.5mg tablets (Niche Generics Ltd)
5968	68354020	Monocor 10mg tablets (Wyeth Pharmaceuticals)
58455	40508020	Bisoprolol 7.5mg tablets (Sandoz Ltd)
58511	40503020	Bisoprolol 1.25mg tablets (Sandoz Ltd)
17462	75023020	Bisoprolol 10mg / Hydrochlorothiazide 6.25mg tablets
17149	75020020	Monozide 10 tablets (Wyeth Pharmaceuticals)
34741	67913020	Carvedilol 3.125mg tablets (IVAX Pharmaceuticals UK Ltd)
47107	567021	Carvedilol 5mg/5ml oral suspension
34501	67708020	Carvedilol 12.5mg tablets (Actavis UK Ltd)
49142	31922020	Carvedilol 3.125mg/5ml oral suspension
18414	72241020	Eucardic 12.5mg tablets (Roche Products Ltd)
46936	67784020	Carvedilol 3.125mg tablets (A A H Pharmaceuticals Ltd)
46935	67701020	Carvedilol 3.125mg tablets (Actavis UK Ltd)
4410	83758020	Carvedilol 6.25mg tablets
54106	35298020	Carvedilol 1.5mg/5ml oral suspension
14146	83760020	Eucardic 6.25mg tablets (Roche Products Ltd)
14117	72243020	Eucardic 3.125mg tablets (Roche Products Ltd)
19202	67720020	Carvedilol 6.25mg tablets (Teva UK Ltd)
19437	72242020	Eucardic 25mg tablets (Roche Products Ltd)
34740	67705020	Carvedilol 6.25mg tablets (Actavis UK Ltd)
59549	19333020	Carvedilol 5mg/5ml oral suspension
61663	67717020	Carvedilol 3.125mg tablets (Teva UK Ltd)
33374	69739020	Carvedilol 12.5mg tablets (Genus Pharmaceuticals Ltd)
7049	72237020	Carvedilol 25mg tablets
817	72238020	Carvedilol 3.125mg tablets
2629	72236020	Carvedilol 12.5mg tablets
8262	68837020	Celiprolol 200mg tablets
16776	68966020	Celectol 400mg Tablet (Pantheon Healthcare Ltd)
35940	91237020	Celectol 400mg tablets (Zentiva)
56485	1155020	Celectol 200mg tablets (Waymade Healthcare Plc)
41740	63783020	Celiprolol 200mg tablets (Teva UK Ltd)
57573	1153020	Celectol 200mg tablets (Dowelhurst Ltd)
4265	68965020	Celectol 200mg Tablet (Pantheon Healthcare Ltd)
7974	68838020	Celiprolol 400mg tablets
42795	61054020	Celiprolol 200mg tablets (Generics (UK) Ltd)
35054	91235020	Celectol 200mg tablets (Zentiva)
30541	57093020	Esmolol HCl 250mg/ml concentrate solution for infusion
26922	56705020	Brevibloc Premixed 100mg/10ml solution for injection vials (Baxter Healthcare Ltd)
32135	56706020	Brevibloc Concentrate 2.5g/10ml solution for infusion ampoules (Baxter Healthcare Ltd)
39819	89270020	Esmolol 2.5g/250ml infusion bags
7066	68976020	Metoprolol 100mg / Hydrochlorothiazide 12.5mg tablets

For peer review only

10627	48722020	Co-Betaloc tablets (Pfizer Ltd)
59222	43810020	Labetalol 100mg/20ml solution for injection ampoules (Focus Pharmaceuticals Ltd)
19068	63903020	Labetalol 50mg/10ml solution for injection pre-filled syringes
38370	90779020	Labetalol 100mg/20ml solution for injection ampoules
19998	51987020	Trandate 100mg/20ml solution for injection ampoules (Focus Pharmaceuticals Ltd)
35778	58483020	Labrocol 100mg Tablet (Lagap)
40240	49924020	Labetalol 400mg tablets (A A H Pharmaceuticals Ltd)
34177	49922020	Labetalol 100mg tablets (A A H Pharmaceuticals Ltd)
1295	68333020	Labetalol 400mg tablets
22793	58484020	Labrocol 200mg Tablet (Lagap)
1597	63899020	Labetalol 100mg tablets
41827	60515020	Labetalol 100mg tablets (Generics (UK) Ltd)
47674	49918020	Labetalol 200mg Tablet (C P Pharmaceuticals Ltd)
4725	68332020	Labetalol 50mg tablets
45250	66745020	Labetalol 400mg tablets (Sandoz Ltd)
44083	57013020	Labetalol 200mg tablets (Actavis UK Ltd)
47673	54575020	Labetalol 400mg Tablet (Approved Prescription Services Ltd)
8707	54881020	Trandate 200mg tablets (Focus Pharmaceuticals Ltd)
2775	63900020	Labetalol 200mg tablets
34171	49917020	Labetalol 100mg Tablet (C P Pharmaceuticals Ltd)
8807	52545020	Trandate 400mg tablets (Focus Pharmaceuticals Ltd)
30770	49923020	Labetalol 200mg tablets (A A H Pharmaceuticals Ltd)
9016	54880020	Trandate 100mg tablets (Focus Pharmaceuticals Ltd)
34188	51424020	Labetalol 200mg Tablet (Celltech Pharma Europe Ltd)
16645	58485020	Labrocol 400mg Tablet (Lagap)
9273	54879020	Trandate 50mg tablets (Focus Pharmaceuticals Ltd)
29998	69479020	Metoros 190mg Tablet (Novartis Pharmaceuticals UK Ltd)
27719	69482020	Metoros ls 95mg Tablet (Geigy Pharmaceuticals)
24461	53655020	Betaloc I.V. 5mg/5ml solution for injection ampoules (AstraZeneca UK Ltd)
14502	52381020	Metoprolol 5mg/5ml solution for injection ampoules
33659	68979020	Hydrochlorothiazide with metoprolol tartrate 25mg with 200mg Modified-release tablet
40167	54126020	Metoprolol 100mg tablets (IVAX Pharmaceuticals UK Ltd)
34509	60551020	Metoprolol 100mg tablets (Generics (UK) Ltd)
34094	50072020	Metoprolol 50mg tablets (A A H Pharmaceuticals Ltd)
3474	53660020	Betaloc-SA 200mg tablets (AstraZeneca UK Ltd)
20082	50243020	Lopresor SR 200mg tablets (Recordati Pharmaceuticals Ltd)
8068	64670020	Metoprolol 200mg modified-release tablets
29762	63850020	Mepranix 50mg Tablet (Ashbourne Pharmaceuticals Ltd)
57240	20539020	Metoprolol 50mg/5ml oral suspension (Drug Tariff Special Order)
11793	67160020	Metoprolol 50mg/5ml oral suspension
10429	50246020	Lopresor 50mg Tablet (Novartis Pharmaceuticals UK Ltd)
8071	48396020	Betaloc 50mg tablets (AstraZeneca UK Ltd)
34925	65979020	Metoprolol 50mg tablets (Sandoz Ltd)
32836	60550020	Metoprolol 50mg tablets (Generics (UK) Ltd)
739	64666020	Metoprolol 50mg tablets
3344	48397020	Betaloc 100mg tablets (AstraZeneca UK Ltd)
34430	57037020	Metoprolol 50mg tablets (Actavis UK Ltd)
47536	770021	Metoprolol tartrate 12.5mg/5ml Oral suspension
55979	20535020	Metoprolol 25mg/5ml oral suspension
46614	195021	Lopresor 50mg tablets (Recordati Pharmaceuticals Ltd)

For peer review only

34584	54125020	Metoprolol 50mg tablets (IVAX Pharmaceuticals UK Ltd)
45289	91796020	Metoprolol tartrate Oral solution
51447	20527020	Metoprolol 12.5mg/5ml oral suspension
29427	68980020	Hydrochlorothiazide with metoprolol tartrate 12.5mg with 100mg tablet
8147	50252020	Lopresoretic Tablet (Novartis Pharmaceuticals UK Ltd)
34854	57038020	Metoprolol 100mg tablets (Actavis UK Ltd)
753	64667020	Metoprolol 100mg tablets
13499	50247020	Lopresor 100mg Tablet (Novartis Pharmaceuticals UK Ltd)
34125	50073020	Metoprolol 100mg tablets (A A H Pharmaceuticals Ltd)
30400	63851020	Mepranix 100mg Tablet (Ashbourne Pharmaceuticals Ltd)
15488	68972020	Metoprolol tartrate with chlortalidone Tablet
34407	56767020	Metoprolol 50mg tablets (Teva UK Ltd)
34890	53461020	Metoprolol 50mg Tablet (Berk Pharmaceuticals Ltd)
34092	56768020	Metoprolol 100mg tablets (Teva UK Ltd)
46740	197021	Lopresor 100mg tablets (Recordati Pharmaceuticals Ltd)
18287	53671020	Co-Betaloc SA tablets (Pfizer Ltd)
20093	68975020	Metoprolol 200mg modified-release / Hydrochlorothiazide 25mg tablets
10716	54539020	Corgard 80mg tablets (Sanofi)
13415	54538020	Corgard 40mg tablets (Sanofi-Synthelabo Ltd)
11338	67627020	Bendroflumethiazide 5mg with Nadolol 40mg tablets
8935	64895020	Nadolol 40mg tablets
2499	64896020	Nadolol 80mg tablets
23134	64899020	Nadolol 40mg / Bendroflumethiazide 5mg tablets
14438	54543020	Corgaretic 80mg tablets (Sanofi-Synthelabo Ltd)
27946	64900020	Nadolol 80mg / Bendroflumethiazide 5mg tablets
5330	48767020	Corgaretic 40mg tablets (Sanofi-Synthelabo Ltd)
59961	21461021	Nebivolol 10mg tablets
7528	80460020	Nebilet 5mg tablets (A. Menarini Farmaceutica Internazionale SRL)
47300	76819020	Nebivolol 2.5mg tablets (Glenmark Generics (Europe) Ltd)
40761	97367020	Nebivolol 2.5mg tablets
751	80458020	Nebivolol 5mg tablets
44808	77127020	Nebivolol 2.5mg tablets (A A H Pharmaceuticals Ltd)
54487	44459020	Nebivolol 2.5mg tablets (Sigma Pharmaceuticals Plc)
52145	61691020	Cyclopenthiazide 0.25mg with oxprenolol 160mg modified-release tablets
2361	54886020	Trasicor 80mg Tablet (Novartis Pharmaceuticals UK Ltd)
3748	59365020	Oxprenolol 160mg Tablet
7474	54884020	Trasicor 20mg Tablet (Novartis Pharmaceuticals UK Ltd)
27357	49156020	Oxprenolol 40mg Tablet (Actavis UK Ltd)
29180	90895020	Trasicor 80mg tablets (Amdipharm Plc)
8673	73918020	Oxprenolol with cyclopenthiazide 160mg+0.25mg Modified-release tablet
35062	90891020	Trasicor 20mg tablets (Amdipharm Plc)
29230	59496020	Slow-pren 160mg Tablet (IVAX Pharmaceuticals UK Ltd)
10777	54889020	Trasicor 160mg Tablet (Novartis Pharmaceuticals UK Ltd)
3516	59360020	Oxprenolol 20mg tablets
21885	74896020	Oxyprenix SR 160mg tablets
4025	51584020	Slow-Trasicor 160mg tablets (AMCo)
24094	90893020	Trasicor 40mg tablets (Amdipharm Plc)
1334	65274020	Oxprenolol 160mg modified-release tablets
2780	59362020	Oxprenolol 80mg tablets
25644	57667020	Apsolox 80mg Tablet (Approved Prescription Services Ltd)

For peer review only

33569	49177020	Oxprenolol sr 160mg Modified-release tablet (Hillcross Pharmaceuticals Ltd)
8290	54885020	Trasicor 40mg Tablet (Novartis Pharmaceuticals UK Ltd)
1333	59361020	Oxprenolol 40mg tablets
13871	73921020	Co-prenozide 160mg/0.25mg modified-release tablets
4429	51994020	Trasidrex modified-release tablets (Mercury Pharma Group Ltd)
4588	52291020	Visken 5mg Tablet (Sovereign Medical Ltd)
25462	61503020	Clopamide 5mg with Pindolol 10mg tablets
20012	52292020	Visken 15mg Tablet (Sovereign Medical Ltd)
5284	65802020	Pindolol 5mg tablets
55853	60484020	Pindolol 15mg Tablet (Hillcross Pharmaceuticals Ltd)
14673	65803020	Pindolol 15mg tablets
35695	78143020	Visken 5mg tablets (AMCo)
32787	78135020	Visken 15mg tablets (AMCo)
9143	52285020	Viskaldix tablets (AMCo)
14057	65806020	Pindolol 10mg / Clopamide 5mg tablets
53177	92021020	Propranolol oral solution
27486	66311020	Propranolol 1mg/1ml solution for injection ampoules
10294	54252020	Inderal 1mg/1ml solution for injection ampoules (AstraZeneca UK Ltd)
5478	59376020	Propranolol 10mg/5ml oral solution sugar free
58491	924020	Propranolol 40mg tablets (Alliance Healthcare (Distribution) Ltd)
34378	50435020	Propranolol 10mg tablets (A A H Pharmaceuticals Ltd)
55416	71580020	Propranolol 40mg tablets (Almus Pharmaceuticals Ltd)
55949	20600020	Propranolol 40mg/5ml oral solution
58297	918020	Propranolol 10mg tablets (Kent Pharmaceuticals Ltd)
31776	63160020	Propranolol 40mg tablets (Generics (UK) Ltd)
45877	99275020	Beta-Prograne 160mg modified-release capsules (Teva UK Ltd)
1048	54495020	Inderal 80mg tablets (AstraZeneca UK Ltd)
34783	50422020	Propranolol 10mg tablets (Actavis UK Ltd)
48682	183020	Propranolol 50mg/5ml oral solution sugar free
3167	59374020	Propranolol 160mg tablets
35938	64558020	Propranolol 80mg modified-release capsules (A A H Pharmaceuticals Ltd)
14808	55786020	Bedranol SR 80mg capsules (Sandoz Ltd)
9185	75255020	Propranolol 80mg/5ml oral solution
34208	51777020	Propranolol SR 160mg Modified-release capsule (C P Pharmaceuticals Ltd)
29763	63927020	Propanix 160mg Tablet (Ashbourne Pharmaceuticals Ltd)
36603	50847020	Propranolol SR 160mg Modified-release capsule (Hillcross Pharmaceuticals Ltd)
45297	64022020	Propranolol 40mg tablets (IVAX Pharmaceuticals UK Ltd)
23587	66314020	Sloprolol 160mg Capsule (C P Pharmaceuticals Ltd)
34214	50424020	Propranolol 160mg tablets (Actavis UK Ltd)
31833	59468020	Angilol 80mg Tablet (DDSA Pharmaceuticals Ltd)
33602	85990020	Slo-Pro 160mg capsules (Generics (UK) Ltd)
41555	50436020	Propranolol 40mg tablets (A A H Pharmaceuticals Ltd)
25359	79243020	Rapranol SR 160mg capsules (Ranbaxy (UK) Ltd)
23131	67621020	Bendroflumethiazide 5mg with Propanolol 160mg modified-release capsules
297	59368020	Propranolol 10mg tablets
40241	56193020	Propranolol LA 160mg Capsule (Approved Prescription Services Ltd)
33644	50437020	Propranolol 80mg tablets (A A H Pharmaceuticals Ltd)

For peer review only

33836	57662020	Apsolol 160mg Tablet (Approved Prescription Services Ltd)
56173	955020	Half Beta-Prograne 80mg modified-release capsules (Actavis UK Ltd)
28128	54244020	Propranolol 80mg Modified-release capsule (Actavis UK Ltd)
1050	54494020	Inderal 40mg tablets (AstraZeneca UK Ltd)
33376	78494020	Probeta LA 160mg Capsule (Trinity Pharmaceuticals Ltd)
23326	71607020	Betadur cr 160mg Modified-release capsule (Monmouth Pharmaceuticals Ltd)
59597	75862020	Propranolol 160mg modified-release capsules (A A H Pharmaceuticals Ltd)
3005	49929020	Inderal LA 160mg capsules (AstraZeneca UK Ltd)
28996	87099020	Bedranol SR 160mg capsules (Sandoz Ltd)
1448	66308020	Propranolol 160mg modified-release capsules
3827	64050020	Propanix 40mg Tablet (Ashbourne Pharmaceuticals Ltd)
57063	957020	Bedranol SR 80mg capsules (Almus Pharmaceuticals Ltd)
45765	96602020	Syprol 40mg/5ml oral solution (Rosemont Pharmaceuticals Ltd)
21839	54823020	Berkolol 80mg Tablet (Berk Pharmaceuticals Ltd)
31214	63161020	Propranolol 80mg tablets (Generics (UK) Ltd)
8978	63928020	Propanix 160mg Modified-release capsule (Ashbourne Pharmaceuticals Ltd)
54623	99279020	Beta-Prograne 160mg modified-release capsules (Actavis UK Ltd)
27700	50423020	Propranolol 40mg tablets (Actavis UK Ltd)
45494	71577020	Propranolol 10mg tablets (Almus Pharmaceuticals Ltd)
32162	59465020	Propranolol 80mg Modified-release capsule (Lagap)
45343	51778020	Propranolol SR 80mg Modified-release capsule (C P Pharmaceuticals Ltd)
3087	75253020	Propranolol 40mg/5ml oral solution sugar free
54297	34198020	Propranolol 50mg/5ml oral solution
769	66307020	Propranolol 80mg modified-release capsules
707	59369020	Propranolol 40mg tablets
47543	627021	Half Beta-Prograne 80mg modified-release capsules (Actavis UK Ltd)
55849	63164020	Propranolol 160mg tablets (Generics (UK) Ltd)
17783	57624020	Spiroprop Tablet (Pharmacia Ltd)
21838	64051020	Propanix 80mg Tablet (Ashbourne Pharmaceuticals Ltd)
34949	54245020	Propranolol 160mg Modified-release capsule (Actavis UK Ltd)
59415	20140021	Propranolol 40mg tablets (Accord Healthcare Ltd)
17082	79593020	Syprol 5mg/5ml oral solution (Rosemont Pharmaceuticals Ltd)
26895	79594020	Syprol 10mg/5ml oral solution (Rosemont Pharmaceuticals Ltd)
15619	57243020	Half-betadur cr 80mg Capsule (Monmouth Pharmaceuticals Ltd)
25367	84837020	Rapranol SR 80mg capsules (Ranbaxy (UK) Ltd)
42152	79595020	Syprol 50mg/5ml oral solution (Rosemont Pharmaceuticals Ltd)
46363	99271020	Half Beta-Prograne 80mg modified-release capsules (Teva UK Ltd)
58407	53353020	Propranolol 80mg tablets (Teva UK Ltd)
39233	68806020	Propranolol 80mg modified-release capsules (Teva UK Ltd)
38433	55581020	Propranolol 50mg/5ml Oral solution (Rosemont Pharmaceuticals Ltd)
22208	84849020	Half propanix la 80mg Modified-release capsule (Ashbourne Pharmaceuticals Ltd)
60565	70592020	Propranolol 40mg tablets (Ranbaxy (UK) Ltd)
34884	66703020	Propranolol 160mg Modified-release capsule (Sandoz Ltd)
47907	538021	Bedranol SR 160mg capsules (Almus Pharmaceuticals Ltd)
36576	63159020	Propranolol 10mg tablets (Generics (UK) Ltd)
20468	72042020	Half Beta-Prograne 80mg modified-release capsules (Tillomed

For peer review only

		Laboratories Ltd)
21866	54822020	Berkolol 40mg Tablet (Berk Pharmaceuticals Ltd)
8331	54498020	Inderal 160mg Tablet (AstraZeneca UK Ltd)
34867	56034020	Propranolol 80mg Capsule (IVAX Pharmaceuticals UK Ltd)
57567	34143020	Propranolol 10mg/5ml oral suspension
34804	53351020	Propranolol 10mg tablets (Teva UK Ltd)
2414	54493020	Inderal 10mg tablets (AstraZeneca UK Ltd)
26229	56515020	Beta-Prograne 160mg modified-release capsules (Tillomed Laboratories Ltd)
220	59375020	Propranolol 5mg/5ml oral solution
940	59370020	Propranolol 80mg tablets
14552	64049020	Propanix 10mg Tablet (Ashbourne Pharmaceuticals Ltd)
27964	57658020	Apsolol 40mg Tablet (Approved Prescription Services Ltd)
26228	85670020	Propanix LA 160mg Modified-release capsule (Ashbourne Pharmaceuticals Ltd)
28048	59466020	Angilol 10mg Tablet (DDSA Pharmaceuticals Ltd)
11711	75254020	Propranolol 50mg/5ml oral solution
61727	20139021	Propranolol 10mg tablets (Accord Healthcare Ltd)
22912	67620020	Bendroflumethiazide 2.5mg with Propanolol 80mg capsules
52777	925020	Propranolol 40mg tablets (Kent Pharmaceuticals Ltd)
26255	78284020	Lopranol la 160mg Capsule (Opus Pharmaceuticals Ltd)
60934	948020	Propranolol 80mg modified-release capsules (Kent Pharmaceuticals Ltd)
34868	53352020	Propranolol 40mg tablets (Teva UK Ltd)
47833	536021	Bedranol SR 80mg capsules (Almus Pharmaceuticals Ltd)
56764	47096020	Propranolol 40mg tablets (Waymade Healthcare Plc)
57342	928020	Propranolol 40mg tablets (Phoenix Healthcare Distribution Ltd)
49863	182020	Propranolol 5mg/5ml oral solution sugar free
34945	59464020	Propranolol 160mg Modified-release capsule (Lagap)
52136	69495020	Bedranol sr 160mg Capsule (Lagap)
52609	942020	Inderal LA 160mg capsules (Sigma Pharmaceuticals Plc)
55228	927020	Propranolol 40mg tablets (Boston Healthcare Ltd)
34185	56194020	Propranolol LA 80mg Modified-release capsule (Approved Prescription Services Ltd)
1006	53253020	Half Inderal LA 80mg capsules (AstraZeneca UK Ltd)
24218	54826020	Berkolol 160mg Tablet (Berk Pharmaceuticals Ltd)
12495	54821020	Berkolol 10mg Tablet (Berk Pharmaceuticals Ltd)
43525	64019020	Propranolol 10mg tablets (IVAX Pharmaceuticals UK Ltd)
28788	85953020	Half propatard la 80mg Modified-release capsule (Galen Ltd)
12054	69745020	Propranolol 80mg / Bendroflumethiazide 2.5mg capsules
4796	54256020	Inderetic 80mg/2.5mg capsules (AstraZeneca UK Ltd)
8369	54259020	Inderex 160mg/5mg modified-release capsules (AstraZeneca UK Ltd)
8987	69746020	Propranolol 160mg modified-release / Bendroflumethiazide 5mg capsules
27727	66690020	Sotalol 2mg/ml injection
33578	79217020	Sotacor 40mg/4ml solution for injection ampoules (Bristol-Myers Squibb Pharmaceuticals Ltd)
38498	95260020	Sotalol 40mg/4ml solution for injection ampoules
17679	66689020	Sotalol 10mg/ml injection
24635	66701020	Sotacor 10mg/ml Injection (Bristol-Myers Squibb Pharmaceuticals Ltd)
1572	66682020	Sotalol 80mg tablets
3691	66694020	Sotalol 160mg with hydrochlorothiazide 25mg tablet

For peer review only

4004	51641020	Sotacor 80mg tablets (Bristol-Myers Squibb Pharmaceuticals Ltd)
51492	20827020	Sotalol 25mg/5ml oral solution
39423	58956020	Sotalol 80mg tablets (A A H Pharmaceuticals Ltd)
35710	91253020	Sotalol 25mg/5ml oral suspension
34640	59307020	Sotalol 40mg Tablet (Tillomed Laboratories Ltd)
34690	61147020	Sotalol 80mg tablets (Sandoz Ltd)
5858	54829020	Beta-Cardone 40mg tablets (Focus Pharmaceuticals Ltd)
34600	65314020	Sotalol 40mg tablets (Teva UK Ltd)
13487	54831020	Beta-Cardone 200mg tablets (Focus Pharmaceuticals Ltd)
6751	54830020	Beta-Cardone 80mg tablets (Focus Pharmaceuticals Ltd)
8061	66693020	Sotalol 80mg with hydrochlorothiazide 12.5mg tablet
15042	66704020	Tolerzide Tablet (Bristol-Myers Squibb Pharmaceuticals Ltd)
34371	63653020	Sotalol 40mg tablets (A A H Pharmaceuticals Ltd)
786	66681020	Sotalol 40mg tablets
9292	66683020	Sotalol 160mg tablets
13051	66686020	Sotalol 200mg tablets
43549	65838020	Sotalol 40mg tablets (IVAX Pharmaceuticals UK Ltd)
12456	51646020	Sotazide Tablet (Bristol-Myers Squibb Pharmaceuticals Ltd)
11380	51642020	Sotacor 160mg tablets (Bristol-Myers Squibb Pharmaceuticals Ltd)
34520	56537020	Sotalol 80mg tablets (Generics (UK) Ltd)
21182	69280020	Hydrochlorothiazide with timolol and amiloride 25mg with 10mg with 2.5mg Tablet
30519	69277020	Amiloride with timolol with hydrochlorothiazide tablets
25730	69274020	Timolol maleate with amiloride and hydrochlorothiazide Tablet
7852	54409020	Blocadren 10mg Tablet (Merck Sharp & Dohme Ltd)
7853	69193020	Timolol 10mg tablets
12517	67082020	Timolol maleate with bendroflumethiazide 20mg + 5mg Tablet
21025	67090020	Prestim forte Tablet (LEO Pharma)
12037	48400020	Betim 10mg Tablet (ICN Pharmaceuticals France S.A.)
19142	67631020	Bendroflumethiazide 2.5mg with Timolol maleate 10mg tablets
29610	88268020	Betim 10mg tablets (Meda Pharmaceuticals Ltd)
12651	67081020	Timolol 10mg / Bendroflumethiazide 2.5mg tablets
8623	52834020	Prestim Tablet (ICN Pharmaceuticals France S.A.)
25363	88272020	Prestim tablets (Meda Pharmaceuticals Ltd)
4605	54433020	Moducren tablets (Merck Sharp & Dohme Ltd)
12497	5741007	OXPRENOLOL 10 MG TAB
13309	4319007	TRASICOR 2 MG INJ
25764	!5661101	PINDOLOL 10MG/CLOPAMIDE 5MG
3041	3534007	SOTALOL HCl 40 MG INJ
12119	4397007	SOTALOL HCl S/R 80 MG TAB
23598	!6704106	SOTALOL HYDROCHLORIDE S/R
26290	3229007	SOTACOR 40 MG INJ
48745	37067020	Timolol 10mg / Amiloride 2.5mg / Hydrochlorothiazide 25mg tablets
8788	3217007	TIMOLOL 10MG/BENDROFLUAZIDE 2.5MG TAB

Calcium channel blockers

Product code	GEM code	Description
60744	22980021	Perindopril erbumine 8mg / Amlodipine 5mg tablets
52440	41782020	Amlodipine 10mg/5ml oral solution
9094	3901007	DILTIAZEM HCl SR 300 MG CAP
30491	!1893701	DILTIAZEM HYDROCHLORIDE
15659	5075007	DILTIAZEM HCL S/R 180 CAP
8024	3142007	DILTIAZEM HCl XL 300 MG CAP
9211	3072007	ADIZEM-XL 180 MG CAP
19015	5949007	ADIZEM CONTINUS 120 MG TAB
25026	!4904103	NIFEDIPINE RETARD
25044	!4904104	NIFEDIPINE RETARD
7823	6750007	NIFEDIPINE TAB 5 mg
25055	!4904102	NIFEDIPINE
25027	!0062301	ADALAT RETARD 10
25054	!0061102	ADALAT
27910	!0062101	ADALAT 5
23458	!7642107	VERAPAMIL SR
23730	5613007	VERAPAMIL 100 MG TAB
18631	!8504107	VERAPAMIL HCL MR
10897	3117007	VERAPAMIL S/F 40 MG/5ML SOL
21665	!1483101	CORDILOX
18690	4427007	SECURON (CALENDAR PACK) 120 MG TAB
3061	57178020	Diltiazem 12hr 180mg modified-release capsules
1995	57177020	Diltiazem 12hr 120mg modified-release capsules
5326	75621020	Diltiazem 24hr 300mg modified-release capsules
9708	75520020	Diltiazem 24hr 120mg modified-release capsules
4923	75521020	Diltiazem 24hr 180mg modified-release capsules
8558	75162020	Adizem xl 120mg Capsule (Napp Pharmaceuticals Ltd)
5194	74598020	Dilzem sr 120mg Capsule (Elan Pharma)
42731	68577020	Diltiazem sr 120mg Capsule (Hillcross Pharmaceuticals Ltd)
42804	65086020	Diltiazem HCl 180mg Capsule (PLIVA Pharma Ltd)
36583	81080020	Zemret xl 180mg Capsule (Neo Laboratories Ltd)
9240	75163020	Adizem xl 180mg Capsule (Napp Pharmaceuticals Ltd)
7562	58764020	Cardene 30mg capsules (Astellas Pharma Ltd)
8201	65032020	Nicardipine 30mg capsules
2926	65031020	Nicardipine 20mg capsules
45292	64571020	Nicardipine 30mg capsules (A A H Pharmaceuticals Ltd)
11943	58763020	Cardene 20mg capsules (Astellas Pharma Ltd)
21872	70717020	Angiopine 5mg Capsule (Ashbourne Pharmaceuticals Ltd)
34522	49116020	Nifedipine 5mg capsules (A A H Pharmaceuticals Ltd)
662	52564020	Adalat 5mg capsules (Bayer Plc)
452	65052020	Nifedipine 10mg capsules
34975	54545020	Nifedipine 5mg capsules (Teva UK Ltd)
42912	54546020	Nifedipine 10mg capsules (Teva UK Ltd)
34607	54160020	Nifedipine 5mg capsules (IVAX Pharmaceuticals UK Ltd)
22142	70808020	Calcilat 10mg Capsule (Eastern Pharmaceuticals Ltd)
43511	49117020	Nifedipine 10mg capsules (A A H Pharmaceuticals Ltd)
269	65053020	Nifedipine 5mg capsules
40074	65054020	Nifedipine 20mg Capsule
55455	1943020	Nifedipine 10mg capsules (Co-Pharma Ltd)
15117	68850020	Nifedipine with atenolol 20mg + 50mg Capsule
20878	70715020	Angiopine 10 capsules (Ashbourne Pharmaceuticals Ltd)

For peer review only

34247	49104020	Nifedipine 10mg Capsule (Berk Pharmaceuticals Ltd)
26265	79357020	Calanif 5mg Capsule (Berk Pharmaceuticals Ltd)
43515	57071020	Nifedipine 10mg capsules (Actavis UK Ltd)
46445	54159020	Nifedipine 10mg capsules (IVAX Pharmaceuticals UK Ltd)
22019	79356020	Calanif 10mg Capsule (Berk Pharmaceuticals Ltd)
2521	52565020	Adalat 10mg capsules (Bayer Plc)
2663	72565020	Diltiazem 240mg modified-release capsules
18606	81472020	Diltiazem and hydrochlorothiazide 150mg+12.5mg modified-release capsules
2453	68603020	Diltiazem 60mg modified-release capsules
11567	81446020	Ramipril 5mg with felodipine 5mg modified-release tablet
11965	81445020	Ramipril 2.5mg with felodipine 2.5mg modified-release tablet
8759	56680020	Verapamil hcl 120mg modified release tablets
4542	68847020	Atenolol 50mg / Nifedipine 20mg modified-release capsules
52728	1077020	Beta-Adalat modified-release capsules (Lexon (UK) Ltd)
61719	1072020	Beta-Adalat modified-release capsules (Waymade Healthcare Plc)
8642	68652020	Tenif 50mg/20mg modified-release capsules (AstraZeneca UK Ltd)
1684	68629020	Beta-Adalat modified-release capsules (Bayer Plc)
28949	84959020	Bi-carzem sr 120mg Modified-release capsule (Tillomed Laboratories Ltd)
20642	84957020	Bi-carzem sr 60mg Modified-release capsule (Tillomed Laboratories Ltd)
5513	74596020	Dilzem sr 60mg Capsule (Elan Pharma)
3676	75265020	Dilzem xl mr 180mg Modified-release capsule (Elan Pharma)
27135	68574020	Diltiazem sr 90mg Capsule (Hillcross Pharmaceuticals Ltd)
2811	57067020	Adizem sr 180mg Modified-release capsule (Napp Pharmaceuticals Ltd)
47415	77105020	Diltiazem sr 60mg Capsule (Hillcross Pharmaceuticals Ltd)
34377	60316020	Diltiazem HCl 90mg Modified-release capsule (Hillcross Pharmaceuticals Ltd)
5296	62587020	Tildiem la 300mg Modified-release capsule (Sanofi)
26270	77217020	Optil xl 300mg Modified-release capsule (Opus Pharmaceuticals Ltd)
60620	21730021	Adizem-XL 240mg capsules (Waymade Healthcare Plc)
1130	84427020	Viazem XL 300mg capsules (Thornton & Ross Ltd)
30197	90599020	Diltiazem 120mg modified-release capsules
7398	84428020	Viazem XL 360mg capsules (Thornton & Ross Ltd)
47732	97275020	Zemret 180 XL capsules (Tillomed Laboratories Ltd)
26309	77216020	Optil xl 240mg Modified-release capsule (Opus Pharmaceuticals Ltd)
1686	57176020	Diltiazem 90mg modified-release capsules
15221	75963020	Dilcardia xl 180mg Modified-release capsule (Generics (UK) Ltd)
17666	84422020	Viazem XL 180mg capsules (Thornton & Ross Ltd)
45759	65091020	Diltiazem HCl 240mg Capsule (PLIVA Pharma Ltd)
23733	69392020	Optil sr 90mg Modified-release capsule (Opus Pharmaceuticals Ltd)
18403	60315020	Diltiazem HCl 180mg Modified-release capsule (Hillcross Pharmaceuticals Ltd)
793	75164020	Adizem xl 240mg Capsule (Napp Pharmaceuticals Ltd)
20890	86888020	Zemtard 240 XL capsules (Galen Ltd)
536	62588020	Tildiem la 200mg Modified-release capsule (Sanofi)
35696	88983020	Kenzem SR 120mg capsules (Kent Pharmaceuticals Ltd)
59863	1895020	Dilzem XL 240 capsules (Lexon (UK) Ltd)
13033	86668020	Angitil XL 240 capsules (Chiesi Ltd)
18852	86924020	Disogram SR 120mg capsules (Ranbaxy (UK) Ltd)
44192	97277020	Zemret 240 XL capsules (Tillomed Laboratories Ltd)
26759	80908020	Zildil SR 60mg capsules (Chanelle Medical UK Ltd)

For peer review only

25777	86145020	Dilcardia SR 120mg capsules (Generics (UK) Ltd)
2592	84421020	Viazem XL 120mg capsules (Thornton & Ross Ltd)
9410	79520020	Angitil SR 120 capsules (Chiesi Ltd)
18830	82004020	Disogram SR 90mg capsules (Ranbaxy (UK) Ltd)
4308	74597020	Dilzem sr 90mg Capsule (Elan Pharma)
13075	87834020	Dilzem XL 180 capsules (Teva UK Ltd)
3370	75264020	Dilzem xl mr 120mg Modified-release capsule (Elan Pharma)
60415	1891020	Dilzem XL 180 capsules (Sigma Pharmaceuticals Plc)
48282	1882020	Diltiazem 90mg modified-release capsules (A A H Pharmaceuticals Ltd)
38831	95539020	Adizem-SR 180mg capsules (Napp Pharmaceuticals Ltd)
27401	88981020	Kenzem SR 90mg capsules (Kent Pharmaceuticals Ltd)
62207	21725021	Adizem-SR 120mg capsules (Waymade Healthcare Plc)
11770	87826020	Dilzem SR 60 capsules (Teva UK Ltd)
13302	87828020	Dilzem SR 90 capsules (Teva UK Ltd)
13926	75622020	Diltiazem 360mg modified-release capsules
23233	84958020	Bi-carzem sr 90mg Modified-release capsule (Tillomed Laboratories Ltd)
26463	81081020	Zemret xl 240mg Capsule (Neo Laboratories Ltd)
47724	95577020	Bi-Carzem XL 240mg capsules (Tillomed Laboratories Ltd)
38865	95559020	Adizem-XL 120mg capsules (Napp Pharmaceuticals Ltd)
27685	65095020	Diltiazem HCl 300mg Capsule (PLIVA Pharma Ltd)
39171	95543020	Bi-Carzem SR 60mg capsules (Tillomed Laboratories Ltd)
19426	79164020	Disogram SR 240mg capsules (Ranbaxy (UK) Ltd)
37774	88979020	Kenzem SR 60mg capsules (Kent Pharmaceuticals Ltd)
26460	75964020	Dilcardia xl 240mg Modified-release capsule (Generics (UK) Ltd)
52276	39293020	Adizem-XL 180mg capsules (DE Pharmaceuticals)
61532	26725021	Diltiazem 120mg modified-release capsules (Sigma Pharmaceuticals Plc)
6309	57068020	Adizem xl 300mg Capsule (Napp Pharmaceuticals Ltd)
26269	69394020	Optil sr 180mg Modified-release capsule (Opus Pharmaceuticals Ltd)
49289	1884020	Diltiazem 120mg modified-release capsules (Alliance Healthcare (Distribution) Ltd)
17425	84733020	Zemtard 120 XL capsules (Galen Ltd)
17406	84734020	Zemtard 180 XL capsules (Galen Ltd)
4635	72177020	Diltiazem 200mg modified-release capsules
10267	81943020	Adizem-XL 200mg capsules (Napp Pharmaceuticals Ltd)
38882	95563020	Adizem-XL 240mg capsules (Napp Pharmaceuticals Ltd)
13240	87832020	Dilzem XL 120 capsules (Teva UK Ltd)
636	86603020	Diltiazem 60mg modified-release capsules
48288	1886020	Diltiazem 120mg modified-release capsules (A A H Pharmaceuticals Ltd)
48457	1881020	Diltiazem 90mg modified-release capsules (Alliance Healthcare (Distribution) Ltd)
4408	76295020	Slozem 240mg capsules (Merck Serono Ltd)
52701	1902020	Tildiem LA 200 capsules (Mawdsley-Brooks & Company Ltd)
44887	86212020	Bi-carzem xl 300mg Capsule (Tillomed Laboratories Ltd)
4808	75522020	Diltiazem 240mg modified-release capsules
16038	87830020	Dilzem SR 120 capsules (Teva UK Ltd)
2686	75266020	Dilzem xl mr 240mg Modified-release capsule (Elan Pharma)
54799	1878020	Tildiem LA 300 capsules (Mawdsley-Brooks & Company Ltd)
17586	80135020	Slozem 300mg capsules (Merck Serono Ltd)
18834	82002020	Disogram SR 60mg capsules (Ranbaxy (UK) Ltd)
30242	90601020	Diltiazem 180mg modified-release capsules

For peer review only

59585	47168020	Uard 120XL capsules (Ennogen Healthcare Ltd)
41489	95547020	Bi-Carzem SR 120mg capsules (Tillomed Laboratories Ltd)
13127	87836020	Dilzem XL 240 capsules (Teva UK Ltd)
36664	81082020	Zemret xl 300mg Capsule (Neo Laboratories Ltd)
18379	86144020	Dilcardia SR 90mg capsules (Generics (UK) Ltd)
31489	86211020	Bi-carzem xl 240mg Capsule (Tillomed Laboratories Ltd)
57208	47167020	Diltiazem 120mg modified-release capsules (Cubic Pharmaceuticals Ltd)
17492	84732020	Zemtard 300 XL capsules (Galen Ltd)
38855	95561020	Adizem-XL 180mg capsules (Napp Pharmaceuticals Ltd)
38634	95575020	Adizem-XL 300mg capsules (Napp Pharmaceuticals Ltd)
48272	1893020	Diltiazem 60mg modified-release capsules (Alliance Healthcare (Distribution) Ltd)
32089	49567020	Diltiazem HCl 120mg Modified-release capsule (Hillcross Pharmaceuticals Ltd)
18874	68687020	Disogram SR 180mg capsules (Ranbaxy (UK) Ltd)
48870	39292020	Adizem-SR 90mg capsules (DE Pharmaceuticals)
47608	97279020	Zemret 300 XL capsules (Tillomed Laboratories Ltd)
23505	81470020	Adizem xl plus 150mg+12.5mg Modified-release capsule (Napp Pharmaceuticals Ltd)
61245	26728021	Diltiazem 60mg modified-release capsules (Sigma Pharmaceuticals Plc)
38632	95541020	Adizem-SR 90mg capsules (Napp Pharmaceuticals Ltd)
11223	79519020	Angitil SR 90 capsules (Chiesi Ltd)
38545	95521020	Tildiem LA 200 capsules (Sanofi)
517	57066020	Adizem sr 120mg Modified-release capsule (Napp Pharmaceuticals Ltd)
15288	86669020	Angitil XL 300 capsules (Chiesi Ltd)
5348	72176020	Diltiazem 300mg modified-release capsules
39298	95545020	Bi-Carzem SR 90mg capsules (Tillomed Laboratories Ltd)
42819	69923020	Diltiazem xl 240mg Capsule (Hillcross Pharmaceuticals Ltd)
3118	72560020	Adizem sr 90mg Modified-release capsule (Napp Pharmaceuticals Ltd)
38876	95519020	Tildiem LA 300 capsules (Sanofi)
32658	75962020	Dilcardia xl 120mg Modified-release capsule (Generics (UK) Ltd)
59098	1889020	Dilzem XL 180 capsules (Lexon (UK) Ltd)
13027	84423020	Viazem XL 240mg capsules (Thornton & Ross Ltd)
56758	47166020	Diltiazem 90mg modified-release capsules (Cubic Pharmaceuticals Ltd)
18404	49568020	Diltiazem 60mg modified-release capsules (A A H Pharmaceuticals Ltd)
31737	80910020	Zildil SR 120mg capsules (Chanelle Medical UK Ltd)
5054	79521020	Angitil SR 180 capsules (Chiesi Ltd)
38818	95537020	Adizem-SR 120mg capsules (Napp Pharmaceuticals Ltd)
47530	76823020	Horizem SR 60mg capsules (Horizon lifecare)
19440	77223020	Disogram SR 300mg capsules (Ranbaxy (UK) Ltd)
5234	76294020	Slozem 180mg capsules (Merck Serono Ltd)
26267	69393020	Optil sr 120mg Modified-release capsule (Opus Pharmaceuticals Ltd)
21145	86146020	Dilcardia SR 60mg capsules (Generics (UK) Ltd)
2528	76293020	Slozem 120mg capsules (Merck Serono Ltd)
3302	54862020	Cardene SR 30mg capsules (Astellas Pharma Ltd)
9386	52064020	Nicardipine 45mg modified-release capsules
5477	52063020	Nicardipine 30mg modified-release capsules
12875	54863020	Cardene SR 45mg capsules (Astellas Pharma Ltd)
737	73827020	Nifedipine 20mg modified-release capsules

For peer review only

4856	73758020	Coracten SR 20mg capsules (UCB Pharma Ltd)
2605	73828020	Nifedipine 10mg modified-release capsules
5162	79993020	Nifedipine 30mg modified-release capsules
3712	79074020	Coracten XL 30mg capsules (UCB Pharma Ltd)
4939	79075020	Coracten XL 60mg capsules (UCB Pharma Ltd)
9750	79994020	Nifedipine 60mg modified-release capsules
2746	73759020	Coracten SR 10mg capsules (UCB Pharma Ltd)
8975	68473020	Verapamil 180mg modified-release capsules
12392	68478020	Univer 180mg modified-release capsules (Teva UK Ltd)
8945	68479020	Univer 240mg modified-release capsules (Teva UK Ltd)
6510	68477020	Univer 120mg modified-release capsules (Teva UK Ltd)
18223	85883020	Trandolapril with verapamil 2mg + 180mg Modified-release capsule
1574	68472020	Verapamil 120mg modified-release capsules
3943	68474020	Verapamil 240mg modified-release capsules
19690	85865020	Verapamil 180mg modified-release / Trandolapril 2mg capsules
20579	85903020	Tarka modified-release capsules (Abbott Laboratories Ltd)
56467	1909020	Tildiem 60mg modified-release tablets (DE Pharmaceuticals)
38964	95553020	Adizem-SR 120mg tablets (Napp Pharmaceuticals Ltd)
34581	68132020	Diltiazem HCl 60mg Modified-release tablet (Kent Pharmaceuticals Ltd)
22619	68606020	Britiazim 60mg Modified-release tablet (Thames Laboratories Ltd)
4732	72564020	Diltiazem 90mg modified-release tablets
21918	85943020	Optil 60mg modified-release tablets (Opus Pharmaceuticals Ltd)
21763	49566020	Diltiazem 60mg modified-release tablets (A A H Pharmaceuticals Ltd)
34475	53802020	Diltiazem HCl 90mg Modified-release tablet (IVAX Pharmaceuticals UK Ltd)
9374	72559020	Adizem 60mg Modified-release tablet (Napp Pharmaceuticals Ltd)
32262	49558020	Diltiazem HCl 60mg Tablet (C P Pharmaceuticals Ltd)
11973	79934020	Calcicard CR 120mg tablets (Teva UK Ltd)
57859	47164020	Diltiazem 90mg modified-release tablets (Cubic Pharmaceuticals Ltd)
49001	11003020	Diltiazem 120mg modified-release tablets (Alliance Healthcare (Distribution) Ltd)
27136	60317020	Diltiazem 90mg modified-release tablets (A A H Pharmaceuticals Ltd)
34824	53803020	Diltiazem HCl 120mg Modified-release tablet (IVAX Pharmaceuticals UK Ltd)
29676	80745020	Calazem 60mg Modified-release tablet (Berk Pharmaceuticals Ltd)
21795	50256020	Retalzem 60 modified-release tablets (Kent Pharmaceuticals Ltd)
13410	70830020	Angiozem 60mg modified-release tablets (Ashbourne Pharmaceuticals Ltd)
41635	53801020	Diltiazem 60mg modified-release tablets (IVAX Pharmaceuticals UK Ltd)
62065	23040021	Diltiazem 90mg modified-release tablets (Colorama Pharmaceuticals Ltd)
21778	54633020	Diltiazem 60mg modified-release tablets (Teva UK Ltd)
1836	71948020	Diltiazem 60mg modified-release tablets
18975	68163020	Calcicard 60mg Tablet (3M Health Care Ltd)
46937	56930020	Diltiazem 60mg modified-release tablets (Actavis UK Ltd)
16850	83067020	Angiozem CR 120mg tablets (Ashbourne Pharmaceuticals Ltd)
49390	1871020	Diltiazem 90mg modified-release tablets (Alliance Healthcare (Distribution) Ltd)
57594	1906020	Tildiem 60mg modified-release tablets (Waymade Healthcare Plc)
21773	60454020	Diltiazem HCl 60mg Tablet (Generics (UK) Ltd)
939	60296020	Tildiem Retard 90mg tablets (Sanofi)
32870	60682020	Diltiazem 60mg modified-release tablets (Sterwin Medicines)

For peer review only

61010	20298021	Diltiazem 120mg modified-release tablets (Cubic Pharmaceuticals Ltd)
219	72563020	Diltiazem 120mg modified-release tablets
43430	61274020	Diltiazem 120mg modified-release tablets (A A H Pharmaceuticals Ltd)
62064	25559021	Diltiazem 120mg modified-release tablets (Mawdsley-Brooks & Company Ltd)
9723	79935020	Calcicard CR 90mg tablets (Teva UK Ltd)
2888	62586020	Tildiem 60mg modified-release tablets (Sanofi)
12639	56931020	Diltiazem HCl 90mg Modified-release tablet (Actavis UK Ltd)
12705	83066020	Angiozem CR 90mg tablets (Ashbourne Pharmaceuticals Ltd)
38066	59378020	Diltiazem HCl 60mg Modified-release tablet (Lagap)
51261	11007020	Tildiem Retard 120mg tablets (Mawdsley-Brooks & Company Ltd)
31676	56932020	Diltiazem HCl 120mg Modified-release tablet (Actavis UK Ltd)
1289	60297020	Tildiem Retard 120mg tablets (Sanofi)
4852	72558020	Adizem sr 120mg Modified-release tablet (Napp Pharmaceuticals Ltd)
26337	76787020	Cabren 10mg modified-release tablets (Teva UK Ltd)
29044	88260020	Neofel XL 10mg tablets (Kent Pharmaceuticals Ltd)
17566	87067020	Felotens XL 10mg tablets (Thornton & Ross Ltd)
38434	93025020	Keloc SR 10mg tablets (Teva UK Ltd)
20459	87085020	Felendil xl 10mg Modified-release tablet (Ratiopharm UK Ltd)
7280	65383020	Plendil 10mg modified-release tablets (AstraZeneca UK Ltd)
29145	91113020	Felendil xl 2.5mg Modified-release tablet (Ratiopharm UK Ltd)
9334	65384020	Plendil 2.5mg modified-release tablets (AstraZeneca UK Ltd)
58339	2044020	Neofel XL 2.5mg tablets (Almus Pharmaceuticals Ltd)
48009	68173020	Felodipine 5mg Modified-release tablet (Sandoz Ltd)
33932	88800020	Parmid XL 5mg tablets (Sandoz Ltd)
491	52181020	Felodipine 2.5mg modified-release tablets
9437	65382020	Plendil 5mg modified-release tablets (AstraZeneca UK Ltd)
25572	86937020	Felogen XL 5mg tablets (Generics (UK) Ltd)
501	52179020	Felodipine 5mg modified-release tablets
43790	92017020	Vascalpha 10mg modified-release tablets (Almus Pharmaceuticals Ltd)
14305	87544020	Vascalpha 10mg modified-release tablets (Actavis UK Ltd)
39357	93501020	Neofel XL 2.5mg tablets (Kent Pharmaceuticals Ltd)
35592	92751020	Cardioplen XL 2.5mg tablets (Chiesi Ltd)
44859	66904020	Felodipine sr 5mg Tablet (Approved Prescription Services Ltd)
33091	66476020	Felodipine 10mg modified-release tablets (A A H Pharmaceuticals Ltd)
55740	2041020	Neofel XL 2.5mg tablets (Actavis UK Ltd)
43394	89315020	Pinefeld XL 10mg tablets (Tillomed Laboratories Ltd)
40633	92015020	Vascalpha 5mg modified-release tablets (Almus Pharmaceuticals Ltd)
37897	94403020	Felotens XL 2.5mg tablets (Thornton & Ross Ltd)
568	52180020	Felodipine 10mg modified-release tablets
36620	88802020	Parmid XL 10mg tablets (Sandoz Ltd)
55306	99312020	Folpik XL 5mg tablets (Teva UK Ltd)
30557	87103020	Felogen XL 10mg tablets (Generics (UK) Ltd)
32922	68176020	Felodipine 10mg Modified-release tablet (Sandoz Ltd)
60652	23052021	Parmid XL 2.5mg tablets (Sandoz Ltd)
24365	87956020	Cardioplen XL 5mg tablets (Chiesi Ltd)
30991	76784020	Cabren 5mg modified-release tablets (Teva UK Ltd)
30915	78440020	Cabren 2.5mg modified-release tablets (Teva UK Ltd)
60884	21738021	Felodipine 2.5mg modified-release tablets (Phoenix Healthcare Distribution Ltd)
10153	87083020	Felendil xl 5mg Modified-release tablet (Ratiopharm UK Ltd)
17557	87065020	Felotens XL 5mg tablets (Thornton & Ross Ltd)
24366	87958020	Cardioplen XL 10mg tablets (Chiesi Ltd)

For peer review only

43512	66473020	Felodipine 5mg modified-release tablets (A A H Pharmaceuticals Ltd)
60569	20172021	Felodipine 2.5mg modified-release tablets (Waymade Healthcare Plc)
28721	88258020	Neofel XL 5mg tablets (Kent Pharmaceuticals Ltd)
35084	87542020	Vascalpha 5mg modified-release tablets (Actavis UK Ltd)
28438	85815020	Triapin 2.5mg/2.5mg modified-release tablets (Sanofi)
17474	78452020	Felodipine 5mg modified-release / Ramipril 5mg tablets
17006	85824020	Triapin 5mg/5mg modified-release tablets (Sanofi)
21162	78451020	Felodipine 2.5mg modified-release / Ramipril 2.5mg tablets
24228	83201020	Nimodrel 20mg modified-release tablet (Opus Pharmaceuticals Ltd)
52017	1952020	Adalat LA 30 tablets (Mawdsley-Brooks & Company Ltd)
5181	71013020	Angiopine MR 20mg tablets (Ashbourne Pharmaceuticals Ltd)
47027	63403020	Nifedipine 10mg Modified-release tablet (Kent Pharmaceuticals Ltd)
45685	99249020	Adanif XL 30mg tablets (Focus Pharmaceuticals Ltd)
13699	83276020	Angiopine la 40mg Tablet (Ashbourne Pharmaceuticals Ltd)
33025	90565020	Nimodrel XL 30mg tablets (Zurich Pharmaceuticals)
22217	83200020	Nimodrel 10mg modified-release tablet (Opus Pharmaceuticals Ltd)
20257	75490020	Cardilate MR 20mg tablets (IVAX Pharmaceuticals UK Ltd)
1262	65057020	Nifedipine 12 20mg Modified-release tablet
49762	1947020	Nifedipine 10mg modified-release tablets (Alliance Healthcare (Distribution) Ltd)
60856	23048021	Nifedipine 10mg modified-release tablets (Sigma Pharmaceuticals Plc)
11512	84876020	Nifedipress MR 10 tablets (Dexcel-Pharma Ltd)
34146	61467020	Nifedipine mr 10mg Modified-release tablet (IVAX Pharmaceuticals UK Ltd)
53500	1950020	Adalat LA 30 tablets (DE Pharmaceuticals)
20311	57957020	Nifedipress mr 20mg Modified-release tablet (Generics (UK) Ltd)
47285	73272020	Nifedipine xl 60mg Tablet (Hillcross Pharmaceuticals Ltd)
25132	90751020	Nifopress MR 20mg tablets (Teva UK Ltd)
59163	47174020	Nifedipine 20mg modified-release tablets (Cubic Pharmaceuticals Ltd)
53278	1953020	Adalat LA 30 tablets (Necessity Supplies Ltd)
9485	50475020	Hypolar Retard 20 tablets (Sandoz Ltd)
1300	54944020	Nifensar xl 20mg Modified-release tablet (Rhone-Poulenc Rorer Ltd)
11769	77698020	Calchan MR 20 tablets (Ranbaxy (UK) Ltd)
17448	84211020	Nifedipress mr 10mg Modified-release tablet (Sterwin Medicines)
8213	75515020	Nifedipine 24 20mg Modified-release tablet
21886	85847020	Nifedipress MR 20 tablets (Actavis UK Ltd)
58557	1975020	Adalat LA 20 tablets (Necessity Supplies Ltd)
41979	85923020	Adipine la 30mg Modified-release tablet (Chiesi Ltd)
14861	77697020	Calchan MR 10 tablets (Ranbaxy (UK) Ltd)
43818	98791020	Adalat LA 60 tablets (Bayer Plc)
43753	98789020	Adalat LA 30 tablets (Bayer Plc)
35646	91231020	Neozipine XL 60mg tablets (Kent Pharmaceuticals Ltd)
9573	54677020	Slofedipine XL 30mg tablets (Zentiva)
34101	61468020	Nifedipine mr 20mg Modified-release tablet (IVAX Pharmaceuticals UK Ltd)
10136	84877020	Nifedipress MR 20 tablets (Dexcel-Pharma Ltd)
13139	88895020	Adipine XL 30mg tablets (Chiesi Ltd)
43410	75517020	Nifedipine extra 60mg Modified-release tablet
21216	87081020	Hypolar Retard 10mg tablets (Sandoz Ltd)
12613	81213020	Unipine xl 30mg Modified-release tablet (Genus Pharmaceuticals Ltd)
19170	82932020	Tensipine MR 10 tablets (Thornton & Ross Ltd)
34187	59923020	Nifedipine 10mg Modified-release tablet (Generics (UK) Ltd)
28688	60425020	Nifedipine 10mg modified-release tablets (A A H Pharmaceuticals Ltd)
541	84301020	Adalat LA 20 tablets (Bayer Plc)

For peer review only

17325	75491020	Cardilate MR 10mg tablets (Teva UK Ltd)
16073	86867020	Nifedipress MR 10 tablets (Teva UK Ltd)
51917	1957020	Adalat LA 60 tablets (Sigma Pharmaceuticals Plc)
5806	82933020	Tensipine MR 20 tablets (Thornton & Ross Ltd)
3930	80874020	Nifedipine 60mg modified-release tablets
12606	76104020	Nifelease 20mg Modified-release tablet (Eastern Pharmaceuticals Ltd)
25919	49118020	Nifedipine 20mg modified-release tablets (A A H Pharmaceuticals Ltd)
57653	1970020	Adalat LA 20 tablets (Sigma Pharmaceuticals Plc)
4239	80337020	Adipine MR 10 tablets (Chiesi Ltd)
30199	68509020	Nifedipine 30mg modified-release tablets
25646	86340020	Nivaten retard 20mg Modified-release tablet (Actavis UK Ltd)
37530	91229020	Neozipine XL 30mg tablets (Kent Pharmaceuticals Ltd)
47217	85924020	Adipine la 60mg Modified-release tablet (Chiesi Ltd)
47614	60424020	Nifedipine 30mg modified-release tablets (A A H Pharmaceuticals Ltd)
5277	83624020	Fortipine LA 40 tablets (AMCo)
10246	88897020	Adipine XL 60mg tablets (Chiesi Ltd)
410	68508020	Nifedipine 10mg modified-release tablets
55824	59219020	Nifedipine 20mg Modified-release tablet (Berk Pharmaceuticals Ltd)
22696	84383020	Slofedipine 20mg tablets (Sterwin Medicines)
34115	68510020	Nifedipine 60mg Modified-release tablet
3711	79719020	Adipine MR 20 tablets (Chiesi Ltd)
13672	71014020	Angiopine MR 10mg tablets (Ashbourne Pharmaceuticals Ltd)
10135	86770020	Nifedipress mr 10mg Modified-release tablet (Sandoz Ltd)
9269	80875020	Nifedipine 40mg modified-release tablets
47887	90567020	Nimodrel XL 60mg tablets (Zurich Pharmaceuticals)
30473	86711020	Coroday MR 20mg tablets (Generics (UK) Ltd)
4227	64631020	Adalat la 60mg Tablet (Bayer Plc)
20591	86868020	Nifedipress MR 20 tablets (Teva UK Ltd)
7541	57458020	Nifopress Retard 20mg tablets (AMCo)
23736	86128020	Hypolar XL 30 tablets (Sandoz Ltd)
15715	85589020	Genalat retard 20mg Modified-release tablet (Wyeth Pharmaceuticals)
58990	47173020	Nifedipine 10mg modified-release tablets (Cubic Pharmaceuticals Ltd)
1449	80873020	Nifedipine 24 30mg Modified-release tablet
39800	94186020	Valni XL 60mg tablets (Zentiva)
56469	1958020	Adalat LA 60 tablets (Necessity Supplies Ltd)
53629	1972020	Adalat retard 20mg tablets (Lexon (UK) Ltd)
37025	90709020	Nifedipine 20mg modified-release tablets
21245	85846020	Nifedipress mr 10mg Modified-release tablet (Actavis UK Ltd)
9553	54678020	Slofedipine XL 60 tablets (Zentiva)
57531	1954020	Adalat LA 60 tablets (Waymade Healthcare Plc)
2280	52574020	Adalat retard 10mg tablets (Bayer Plc)
49338	1966020	Nifedipine 20mg modified-release tablets (Alliance Healthcare (Distribution) Ltd)
17338	84579020	Nifedotard 20 mr 20mg Modified-release tablet (Galen Ltd)
46887	99251020	Adanif XL 60mg tablets (Focus Pharmaceuticals Ltd)
17342	86339020	Nivaten retard 10mg Modified-release tablet (Actavis UK Ltd)
43222	97381020	Valni 20 Retard tablets (Tillomed Laboratories Ltd)
2343	52575020	Adalat retard 20mg tablets (Bayer Plc)
37184	94184020	Valni XL 30mg tablets (Zentiva)
17599	88811020	Verapress MR 240mg tablets (Sandoz Ltd)
13856	84570020	Verapress MR 240mg tablets (Actavis UK Ltd)
19457	84468020	Ranvera MR 240mg tablets (Ranbaxy (UK) Ltd)
13251	78368020	Vera-Til SR 240mg tablets (Tillomed Laboratories Ltd)

For peer review only

47222	75703020	Verapamil 120mg modified-release tablets (A A H Pharmaceuticals Ltd)
700	84862020	Vera-Til SR 120mg tablets (Tillomed Laboratories Ltd)
16328	78409020	Verapress MR 240mg tablets (Dexcel-Pharma Ltd)
59264	872020	Securon SR 240mg tablets (DE Pharmaceuticals)
45308	71935020	Verapamil 240mg modified-release tablets (Generics (UK) Ltd)
9569	65404020	Verapamil 120mg modified-release tablets
1298	65403020	Verapamil 240mg modified-release tablets
11972	86724020	Vertab SR 240 tablets (Chiesi Ltd)
19459	51963020	Verapamil 240mg modified-release tablets (A A H Pharmaceuticals Ltd)
47230	59413020	Verapamil 240mg modified-release tablets (Teva UK Ltd)
46884	65991020	Verapamil hc 240mg Modified-release tablet (Sandoz Ltd)
13965	71435020	Cordilox MR 240mg tablets (Teva UK Ltd)
3343	56683020	Half Securon SR 120mg tablets (Abbott Laboratories Ltd)
30462	85740020	Ethimil MR 240mg tablets (Genus Pharmaceuticals Ltd)
3342	58870020	Securon SR 240mg tablets (Abbott Laboratories Ltd)
43879	97884020	Vera-Til SR 240mg tablets (Actavis UK Ltd)
42625	97882020	Vera-Til SR 120mg tablets (Actavis UK Ltd)
45051	56381020	Verapamil hc 240mg Modified-release tablet (Actavis UK Ltd)
51461	865020	Securon SR 240mg tablets (Waymade Healthcare Plc)
29637	89660020	Verapress MR 240mg tablets (Teva UK Ltd)
47529	98903020	Nifedipine 20mg/ml oral drops
46233	91269020	Amlodipine Oral solution
46724	415021	Amlodipine 5mg/5ml oral solution
55257	19777020	Diltiazem 60mg/5ml oral solution
47707	93331020	Nifedipine Oral solution
31490	81761020	Zolvera 40mg/5ml oral solution (Rosemont Pharmaceuticals Ltd)
11777	75258020	Verapamil 40mg/5ml oral solution sugar free
54983	18978020	Amlodipine 2.5mg/5ml oral suspension
61374	29717020	Amlodipine 4mg/5ml oral suspension
45070	99159020	Amlodipine 10mg/5ml oral suspension
16162	91263020	Amlodipine 5mg/5ml oral suspension
47002	413021	Amlodipine 10mg/5ml sugar free Oral suspension
11922	90541020	Diltiazem 60mg/5ml oral suspension
26774	91013020	Nifedipine 10mg/5ml Oral suspension
53357	20553020	Nifedipine 10mg/5ml oral suspension
37726	92999020	Nifedipine 100mg/5ml oral suspension
53990	20557020	Nifedipine 5mg/5ml oral suspension
27295	70927020	Securon IV 5mg/2ml solution for injection ampoules (Abbott Laboratories Ltd)
19325	48764020	Cordilox 2.5mg/ml Injection (IVAX Pharmaceuticals UK Ltd)
26674	68346020	Verapamil 5mg/2ml solution for injection ampoules
31761	88254020	Amlostin 10mg tablets (Discovery Pharmaceuticals Ltd)
17640	88252020	Amlostin 5mg tablets (Discovery Pharmaceuticals Ltd)
61422	2002020	Amlodipine 5mg tablets (Accord Healthcare Ltd)
59001	68889020	Amlodipine 10mg tablets (Generics (UK) Ltd)
54696	68498020	Amlodipine 10mg tablets (Sandoz Ltd)
39914	67735020	Amlodipine 5mg tablets (Teva UK Ltd)
749	88863020	Amlodipine 5mg tablets
56147	2023020	Amlodipine 10mg tablets (Accord Healthcare Ltd)
43880	75222020	Amlodipine 5mg tablets (Almus Pharmaceuticals Ltd)
6856	88865020	Amlodipine 10mg tablets
32917	67688020	Amlodipine 5mg tablets (IVAX Pharmaceuticals UK Ltd)

For peer review only

54654	75178020	Amlodipine 10mg tablets (Almus Pharmaceuticals Ltd)
42210	68111020	Amlodipine 10mg tablets (Zentiva)
59762	67739020	Amlodipine 10mg tablets (Teva UK Ltd)
39804	70039020	Amlodipine 5mg tablets (Dr Reddy's Laboratories (UK) Ltd)
58580	38962020	Amlodipine 10mg tablets (APC Pharmaceuticals & Chemicals (Europe) Ltd)
46792	99771020	Olmesartan medoxomil with amlodipine and hydrochlorothiazide 40mg + 5mg + 12.5mg Tablet
49636	38506020	Amlodipine 10mg tablets (DE Pharmaceuticals)
3917	72822020	Istin 5mg tablets (Pfizer Ltd)
55358	99777020	Olmesartan medoxomil with amlodipine and hydrochlorothiazide 40mg + 10mg + 25mg Tablet
45279	68495020	Amlodipine 5mg tablets (Sandoz Ltd)
46687	99769020	Olmesartan medoxomil with amlodipine and hydrochlorothiazide 20mg + 5mg + 12.5mg Tablet
32595	67634020	Amlodipine 5mg tablets (A A H Pharmaceuticals Ltd)
46715	99773020	Olmesartan medoxomil with amlodipine and hydrochlorothiazide 40mg + 10mg + 12.5mg Tablet
34093	67637020	Amlodipine 10mg tablets (A A H Pharmaceuticals Ltd)
60244	2021020	Amlodipine 10mg tablets (Phoenix Healthcare Distribution Ltd)
56334	2018020	Amlodipine 10mg tablets (Bristol Laboratories Ltd)
36202	68410020	Amlodipine 10mg tablets (Actavis UK Ltd)
47467	99775020	Olmesartan medoxomil with amlodipine and hydrochlorothiazide 40mg + 5mg + 25mg Tablet
53868	68406020	Amlodipine 5mg tablets (Actavis UK Ltd)
54515	2008020	Amlodipine 10mg tablets (Alliance Healthcare (Distribution) Ltd)
54633	1996020	Amlodipine 5mg tablets (Bristol Laboratories Ltd)
5914	72823020	Istin 10mg tablets (Pfizer Ltd)
43470	75838020	Amlodipine 5mg tablets (Wockhardt UK Ltd)
40316	96923020	Olmesartan medoxomil 20mg / Amlodipine 5mg tablets
39984	96929020	Sevikar 20mg/5mg tablets (Daiichi Sankyo UK Ltd)
35096	92773020	Exforge 10mg/160mg tablets (Novartis Pharmaceuticals UK Ltd)
47573	99783020	Sevikar HCT 40mg/5mg/12.5mg tablets (Daiichi Sankyo UK Ltd)
53220	99791020	Sevikar HCT 40mg/10mg/25mg tablets (Daiichi Sankyo UK Ltd)
8310	69563020	Isradipine 2.5mg tablets
8257	69560020	Prescal 2.5mg tablets (Novartis Pharmaceuticals UK Ltd)
11966	74740020	Motens 2mg tablets (GlaxoSmithKline UK Ltd)
5158	74744020	Lacidipine 2mg tablets
3221	74745020	Lacidipine 4mg tablets
56994	77966020	Lacidipine 4mg tablets (Teva UK Ltd)
57680	78002020	Lacidipine 4mg tablets (A A H Pharmaceuticals Ltd)
9670	74741020	Motens 4mg tablets (GlaxoSmithKline UK Ltd)
60699	2051020	Lacidipine 2mg tablets (Sigma Pharmaceuticals Plc)
61611	23689021	Lercanidipine 10mg tablets (DE Pharmaceuticals)
5570	85619020	Zanidip 10mg tablets (Recordati Pharmaceuticals Ltd)
56767	77347020	Lercanidipine 20mg tablets (Generics (UK) Ltd)
14300	90707020	Zanidip 20mg tablets (Recordati Pharmaceuticals Ltd)
5593	79553020	Lercanidipine 10mg tablets
57444	14783021	Lercanidipine 10mg tablets (Aptil Pharma Ltd)
59233	77287020	Lercanidipine 20mg tablets (Actavis UK Ltd)
47331	77345020	Lercanidipine 10mg tablets (Generics (UK) Ltd)
13243	90705020	Lercanidipine 20mg tablets
1854	64630020	Adalat la 30mg Tablet (Bayer Plc)
38107	73256020	Nifedipine sr 30mg Tablet (Hillcross Pharmaceuticals Ltd)

For peer review only

10595	68599020	Nimotop 30mg tablets (Bayer Plc)
11547	74190020	Nimodipine 30mg tablets
40668	96927020	Olmesartan medoxomil 40mg / Amlodipine 10mg tablets
40639	96925020	Olmesartan medoxomil 40mg / Amlodipine 5mg tablets
41203	96933020	Sevikar 40mg/10mg tablets (Daiichi Sankyo UK Ltd)
41205	96931020	Sevikar 40mg/5mg tablets (Daiichi Sankyo UK Ltd)
46355	99779020	Sevikar HCT 20mg/5mg/12.5mg tablets (Daiichi Sankyo UK Ltd)
60067	22976021	Perindopril erbumine 4mg / Amlodipine 5mg tablets
60684	22974021	Perindopril erbumine 4mg / Amlodipine 10mg tablets
35317	92769020	Exforge 5mg/80mg tablets (Novartis Pharmaceuticals UK Ltd)
26252	53578020	Berkatens 160mg Tablet (Berk Pharmaceuticals Ltd)
32590	60639020	Verapamil 40mg tablets (Generics (UK) Ltd)
46009	63423020	Verapamil 120mg tablets (Kent Pharmaceuticals Ltd)
35729	54551020	Verapamil 80mg tablets (Teva UK Ltd)
28843	50788020	Verapamil hc 80mg Tablet (Celltech Pharma Europe Ltd)
33471	56374020	Verapamil 40mg tablets (Actavis UK Ltd)
8524	58375020	Securon 40mg Tablet (Abbott Laboratories Ltd)
12104	53065020	Cordilox 160mg tablets (IVAX Pharmaceuticals UK Ltd)
3057	58377020	Securon 120mg tablets (Abbott Laboratories Ltd)
41693	60641020	Verapamil 120mg tablets (Generics (UK) Ltd)
1118	65395020	Verapamil 40mg tablets
28844	53573020	Berkatens 120mg Tablet (Berk Pharmaceuticals Ltd)
34959	50799020	Verapamil 40mg tablets (A A H Pharmaceuticals Ltd)
16677	48759020	Cordilox 80mg tablets (IVAX Pharmaceuticals UK Ltd)
39009	54550020	Verapamil 40mg tablets (Teva UK Ltd)
10688	69592020	Verapamil 160mg tablets
1120	65396020	Verapamil 80mg tablets
1748	53062020	Cordilox 120mg tablets (IVAX Pharmaceuticals UK Ltd)
41586	56375020	Verapamil 80mg tablets (Actavis UK Ltd)
41679	56209020	Verapamil 80mg tablets (IVAX Pharmaceuticals UK Ltd)
8884	48758020	Cordilox 40mg tablets (IVAX Pharmaceuticals UK Ltd)
1747	65397020	Verapamil 120mg tablets
40405	54552020	Verapamil 120mg tablets (Teva UK Ltd)
46955	60640020	Verapamil 80mg tablets (Generics (UK) Ltd)
22826	56814020	Securon 160mg Tablet (Abbott Laboratories Ltd)
19175	56208020	Verapamil 40mg tablets (IVAX Pharmaceuticals UK Ltd)
23872	53571020	Berkatens 40mg Tablet (Berk Pharmaceuticals Ltd)
25059	53572020	Berkatens 80mg Tablet (Berk Pharmaceuticals Ltd)
10832	58376020	Securon 80mg Tablet (Abbott Laboratories Ltd)
31711	50800020	Verapamil 80mg tablets (A A H Pharmaceuticals Ltd)
29	72826020	Amlodipine besilate 5mg tablets
729	87704020	Amlodipine maleate 5mg tablets
6477	87706020	Amlodipine maleate 10mg tablets
71	72827020	Amlodipine besilate 10mg tablets
35304	92957020	Valsartan 160mg with amlodipine 10mg tablets
35173	92953020	Valsartan 160mg with amlodipine 5mg tablets
35174	92951020	Valsartan 80mg with amlodipine 5mg tablets
1538	68602020	Diltiazem 60mg tablets
47616	99785020	Sevikar HCT 40mg/10mg/12.5mg tablets (Daiichi Sankyo UK Ltd)
47727	99789020	Sevikar HCT 40mg/5mg/25mg tablets (Daiichi Sankyo UK Ltd)
35343	92765020	Amlodipine 5mg / Valsartan 160mg tablets
35189	92767020	Amlodipine 10mg / Valsartan 160mg tablets
35697	92771020	Exforge 5mg/160mg tablets (Novartis Pharmaceuticals UK Ltd)
35329	92763020	Amlodipine 5mg / Valsartan 80mg tablets

For peer review only

For peer review only

Thiazides and thiazide-like diuretics

Product code	GEM code	Description
23505	81470020	Adizem xl plus 150mg+12.5mg Modified-release capsule (Napp Pharmaceuticals Ltd)
3701	69292020	Amiloride 2.5mg / hydrochlorothiazide 25mg tablets
2002	69291020	Amiloride 5mg / hydrochlorothiazide 50mg tablets
4034	69293020	Amiloride 5mg / hydrochlorothiazide 50mg/5ml solution
3526	69296020	Amiloride with atenolol with hydrochlorothiazide capsules
30519	69277020	Amiloride with timolol with hydrochlorothiazide tablets
8526	48201020	Aprinox 2.5mg tablets (AMCo)
7698	48202020	Aprinox 5mg tablets (Amdipharm Plc)
13526	63763020	Atenix Co 100 tablets (Ashbourne Pharmaceuticals Ltd)
21873	63762020	Atenix Co 50 tablets (Ashbourne Pharmaceuticals Ltd)
1788	67598020	Atenolol 100mg with Chlortalidone 25mg tablets
581	67597020	Atenolol 50mg with Chlortalidone 12.5mg tablets
4983	68844020	Atenolol with amiloride and hydrochlorothiazide capsules
15457	48303020	Baycaron 25mg Tablet (Bayer Plc)
1211	67326020	Bendroflumethiazide 2.5mg / Potassium chloride 630mg (potassium 8.4mmol) modified-release tablets
34803	62827020	Bendroflumethiazide 2.5mg Tablet (Regent Laboratories Ltd)
2	58976020	Bendroflumethiazide 2.5mg tablets
33651	48846020	Bendroflumethiazide 2.5mg tablets (A A H Pharmaceuticals Ltd)
34059	55391020	Bendroflumethiazide 2.5mg tablets (Actavis UK Ltd)
40886	68065020	Bendroflumethiazide 2.5mg tablets (Almus Pharmaceuticals Ltd)
33415	63225020	Bendroflumethiazide 2.5mg tablets (Generics (UK) Ltd)
27689	57793020	Bendroflumethiazide 2.5mg tablets (IVAX Pharmaceuticals UK Ltd)
47844	66033020	Bendroflumethiazide 2.5mg tablets (Kent Pharmaceuticals Ltd)
34602	66500020	Bendroflumethiazide 2.5mg tablets (Sovereign Medical Ltd)
31670	54691020	Bendroflumethiazide 2.5mg tablets (Teva UK Ltd)
27256	48838020	Bendroflumethiazide 2.5mg tablets (Wockhardt UK Ltd)
22912	67620020	Bendroflumethiazide 2.5mg with Propanolol 80mg capsules
19142	67631020	Bendroflumethiazide 2.5mg with Timolol maleate 10mg tablets
7351	90559020	Bendroflumethiazide 2.5mg/5ml oral suspension
58	58977020	Bendroflumethiazide 5mg tablets
23427	48847020	Bendroflumethiazide 5mg tablets (A A H Pharmaceuticals Ltd)
34124	55390020	Bendroflumethiazide 5mg tablets (Actavis UK Ltd)
40149	57796020	Bendroflumethiazide 5mg tablets (IVAX Pharmaceuticals UK Ltd)
41517	54690020	Bendroflumethiazide 5mg tablets (Teva UK Ltd)
31820	48839020	Bendroflumethiazide 5mg tablets (Wockhardt UK Ltd)
11338	67627020	Bendroflumethiazide 5mg with Nadolol 40mg tablets
23131	67621020	Bendroflumethiazide 5mg with Propanolol 160mg modified-release capsules
53812	92033020	Bendroflumethiazide oral solution
30272	64126020	Benthiazide with Triamterene capsules
21803	48386020	Berkozide 2.5mg Tablet (Berk Pharmaceuticals Ltd)
21867	48387020	Berkozide 5mg Tablet (Berk Pharmaceuticals Ltd)
3203	68134020	Capozide LS Tablet (E R Squibb and Sons Ltd)
32166	80831020	Capto-co 25mg+50mg Tablet (IVAX Pharmaceuticals UK Ltd)
11641	68130020	Captopril 25mg with Hydrochlorothiazide 12.5mg tablets
10902	68129020	Captopril 50mg with Hydrochlorothiazide 25mg tablets
17655	72036020	Carace 10 Tablet (Bristol-Myers Squibb Pharmaceuticals Ltd)
39147	95901020	Carace 20 Plus tablets (Merck Sharp & Dohme Ltd)
9764	72035020	Carace 20 Tablet (Bristol-Myers Squibb Pharmaceuticals Ltd)

For peer review only - http://bmjopen.bmj.com/site/about/guidelines.xhtml

62066	23031021	Cardide SR 1.5mg tablets (Teva UK Ltd)
18973	52358020	Centyl 2.5mg Tablet (Edwin Burgess Ltd)
29991	52359020	Centyl 5mg Tablet (Edwin Burgess Ltd)
13246	90731020	Chlorothiazide 150mg/5ml oral suspension
54679	98525020	Chlorothiazide 250mg tablets
59834	32028020	Chlorothiazide 250mg/5ml oral solution
6816	86950020	Chlorothiazide 250mg/5ml oral suspension
56804	19362020	Chlorothiazide 25mg/5ml oral suspension
8836	61214020	Chlorothiazide 500mg tablets
60603	19370020	Chlorothiazide 50mg/5ml oral suspension
54341	30321020	Chlorothiazide 5mg/5ml oral suspension
55889	91770020	Chlorothiazide oral solution
3548	61268020	Chlortalidone 100mg tablets
19055	61275020	Chlortalidone 12.5mg with Atenolol 50mg tablets
16786	61276020	Chlortalidone 25mg with Atenolol 100mg tablets
605	61267020	Chlortalidone 50mg tablets
18733	70712020	Co-amilozide 5mg with 50mg/ml oral solution
10316	83981020	CoAprovel 150mg/12.5mg tablets (Sanofi)
11526	83982020	CoAprovel 300mg/12.5mg tablets (Sanofi)
35196	92367020	CoAprovel 300mg/25mg tablets (Sanofi)
5721	73883020	Co-tenidone 100mg/25mg tablets
34899	53522020	Co-tenidone 100mg/25mg tablets (A A H Pharmaceuticals Ltd)
46952	56911020	Co-tenidone 100mg/25mg tablets (Actavis UK Ltd)
37725	60353020	Co-tenidone 100mg/25mg tablets (Generics (UK) Ltd)
34012	55246020	Co-tenidone 100mg/25mg tablets (IVAX Pharmaceuticals UK Ltd)
41572	54654020	Co-tenidone 100mg/25mg tablets (Teva UK Ltd)
9783	73882020	Co-tenidone 50mg/12.5mg tablets
32094	53521020	Co-tenidone 50mg/12.5mg tablets (A A H Pharmaceuticals Ltd)
31708	56910020	Co-tenidone 50mg/12.5mg tablets (Actavis UK Ltd)
34449	60352020	Co-tenidone 50mg/12.5mg tablets (Generics (UK) Ltd)
34034	55247020	Co-tenidone 50mg/12.5mg tablets (IVAX Pharmaceuticals UK Ltd)
34825	54653020	Co-tenidone 50mg/12.5mg tablets (Teva UK Ltd)
51258	34961020	Coversyl Arginine Plus 5mg/1.25mg tablets (DE Pharmaceuticals)
37908	94709020	Coversyl Arginine Plus 5mg/1.25mg tablets (Servier Laboratories Ltd)
52145	61691020	Cyclopenthiazide 0.25mg with oxprenolol 160mg modified-release tablets
1170	61684020	Cyclopenthiazide 500microgram tablets
2833	61687020	CYCLOPENTHIAZIDE -K tablets
26220	68015020	Delvas Tablet (Berk Pharmaceuticals Ltd)
18606	81472020	Diltiazem and hydrochlorothiazide 150mg+12.5mg modified-release capsules
4044	49062020	Diurexan 20mg tablets (Meda Pharmaceuticals Ltd)
33724	86952020	Diuril 250mg/5ml oral suspension (Imported (United States))
5189	51068020	Enalapril 20mg / Hydrochlorothiazide 12.5mg tablets
18267	64533020	Enduron 5mg Tablet (Abbott Laboratories Ltd)
17252	49321020	Esidrex 25mg Tablet (Novartis Pharmaceuticals UK Ltd)
13363	49322020	Esidrex 50mg Tablet (Novartis Pharmaceuticals UK Ltd)
41885	95348020	Ethibide XL 1.5mg tablets (Genus Pharmaceuticals Ltd)
13525	58231020	Hydrenox 50mg Tablet (Knoll Ltd)
542	63248020	Hydrochlorothiazide 25mg tablets
3517	63249020	Hydrochlorothiazide 50mg tablets
48132	94685020	Hydrochlorothiazide Capsule
57488	92809020	Hydrochlorothiazide Oral solution

For peer review only

19890	63252020	Hydrochlorothiazide with amiloride 25mgwith2.5mg Tablet
22923	63254020	Hydrochlorothiazide with amiloride 50mg with 5mg Tablet
28177	68841020	Hydrochlorothiazide with atenolol and amiloride Capsule
15135	69269020	Hydrochlorothiazide with captopril 12.5mg with 25mg Tablet
11133	69268020	Hydrochlorothiazide with captopril 25mg with 50mg Tablet
38367	94427020	Hydrochlorothiazide with losartan 12.5mg with 100mg Tablet
14738	79035020	Hydrochlorothiazide with losartan 12.5mg with 50mg Tablet
24632	89781020	Hydrochlorothiazide with losartan 25mg with 100mg Tablet
29427	68980020	Hydrochlorothiazide with metoprolol tartrate 12.5mg with 100mg tablet
33659	68979020	Hydrochlorothiazide with metoprolol tartrate 25mg with 200mg Modified-release tablet
35380	90735020	Hydrochlorothiazide with olmesartan medoxomil 12.5mg with 20mg tablet
39021	90733020	Hydrochlorothiazide with olmesartan medoxomil 25mg with 20mg tablet
21182	69280020	Hydrochlorothiazide with timolol and amiloride 25mg with 10mg with 2.5mg Tablet
15127	63257020	Hydrochlorothiazide with triamterene 25mgwith50mg Tablet
24484	87996020	Hydrochlorothiazide with valsartan 12.5mg with 160mg Tablet
24268	88422020	Hydrochlorothiazide with valsartan 12.5mg with 80mg Tablet
23456	88006020	Hydrochlorothiazide with valsartan 25mg with 160mg Tablet
12110	63279020	Hydroflumethiazide 50mg Tablet
29529	63282020	Hydroflumethiazide with spironolactone 25mg+25mg Tablet
45916	63283020	Hydroflumethiazide with spironolactone 50mg+50mg Tablet
3054	49873020	Hygroton 100mg Tablet (Alliance Pharmaceuticals Ltd)
3997	49872020	Hygroton 50mg tablets (Alliance Pharmaceuticals Ltd)
8891	49876020	Hygroton -k Tablet (Novartis Pharmaceuticals UK Ltd)
25500	68145020	Hypertane 50 Tablet (Schwarz Pharma Ltd)
5112	63541020	Indapamide 1.5mg modified-release tablets
46675	75757020	Indapamide 1.5mg modified-release tablets (A A H Pharmaceuticals Ltd)
60020	14463021	Indapamide 1.5mg modified-release tablets (Waymade Healthcare Plc)
2612	63540020	Indapamide 2.5mg tablets
48099	60381020	Indapamide 2.5mg tablets (A A H Pharmaceuticals Ltd)
43516	57007020	Indapamide 2.5mg tablets (Actavis UK Ltd)
54316	683020	Indapamide 2.5mg tablets (Alliance Healthcare (Distribution) Ltd)
56296	685020	Indapamide 2.5mg tablets (Boston Healthcare Ltd)
56760	690020	Indapamide 2.5mg tablets (Co-Pharma Ltd)
34551	60492020	Indapamide 2.5mg tablets (Generics (UK) Ltd)
40907	68581020	Indapamide 2.5mg tablets (Genus Pharmaceuticals Ltd)
55259	682020	Indapamide 2.5mg tablets (Kent Pharmaceuticals Ltd)
42906	63983020	Indapamide 2.5mg tablets (Niche Generics Ltd)
49529	689020	Indapamide 2.5mg tablets (Phoenix Healthcare Distribution Ltd)
33083	56755020	Indapamide 2.5mg tablets (Teva UK Ltd)
48079	59993020	Indapamide 2.5mg tablets (Zentiva)
44168	97012020	Indipam XL 1.5mg tablets (Actavis UK Ltd)
1021	51047020	Innozide 20mg/12.5mg tablets (Merck Sharp & Dohme Ltd)
11448	80124020	Irbesartan 150mg / Hydrochlorothiazide 12.5mg tablets
11469	80125020	Irbesartan 300mg / Hydrochlorothiazide 12.5mg tablets
35481	92365020	Irbesartan 300mg / Hydrochlorothiazide 25mg tablets
28157	53155020	Kalspare ls Tablet (Dominion Pharma)
12546	53154020	Kalspare Tablet (Dominion Pharma)

For peer review only

6786	50823020	Lisinopril 10mg / Hydrochlorothiazide 12.5mg tablets
37710	67694020	Lisinopril 10mg / Hydrochlorothiazide 12.5mg tablets (Teva UK Ltd)
6468	50822020	Lisinopril 20mg / Hydrochlorothiazide 12.5mg tablets
55399	67847020	Lisinopril 20mg / Hydrochlorothiazide 12.5mg tablets (A A H Pharmaceuticals Ltd)
54201	75924020	Lisinopril 20mg / Hydrochlorothiazide 12.5mg tablets (Almus Pharmaceuticals Ltd)
33353	67697020	Lisinopril 20mg / Hydrochlorothiazide 12.5mg tablets (Teva UK Ltd)
56244	1491020	Lisinopril 20mg / Hydrochlorothiazide 12.5mg tablets (Tillomed Laboratories Ltd)
8147	50252020	Lopresoretic Tablet (Novartis Pharmaceuticals UK Ltd)
43184	97115020	Mapemid XL 1.5mg tablets (Teva UK Ltd)
17143	64368020	Mefruside 25mg Tablet
8464	64409020	Meprobamate with bendroflumethiazide Tablet
8602	50380020	Metenix 5mg tablets (Sanofi)
20057	64530020	Methyclothiazide 5mg Tablet
53674	45436020	Metolazone 2.5mg tablets
49752	33486020	Metolazone 2.5mg/5ml oral solution
4334	63211020	Metolazone 500microgram low dose Tablet
4332	64663020	Metolazone 5mg tablets
55777	33494020	Metolazone 5mg/5ml oral solution
54329	33496020	Metolazone 5mg/5ml oral suspension
54643	94928020	Metolazone Oral solution
15488	68972020	Metoprolol tartrate with chlortalidone Tablet
3293	50482020	Moduretic Oral solution (Bristol-Myers Squibb Pharmaceuticals Ltd)
348	50481020	Moduretic Tablet (Bristol-Myers Squibb Pharmaceuticals Ltd)
27957	78343020	Natramid 2.5mg Tablet (Trinity Pharmaceuticals Ltd)
7641	58678020	Natrilix 2.5mg tablets (Servier Laboratories Ltd)
3056	84103020	Natrilix SR 1.5mg tablets (Servier Laboratories Ltd)
2046	50633020	Navidrex 500microgram tablets (AMCo)
1125	50636020	Navidrex -k Tablet (Novartis Pharmaceuticals UK Ltd)
24189	75589020	Neo-bendromax 2.5mg Tablet (Ashbourne Pharmaceuticals Ltd)
24190	75590020	Neo-bendromax 5mg Tablet (Ashbourne Pharmaceuticals Ltd)
46302	99566020	Neo-Naclex 2.5mg tablets (AMCo)
1209	50651020	Neo-Naclex 5mg tablets (Mercury Pharma Group Ltd)
1213	50654020	Neo-Naclex-K modified-release tablets (Mercury Pharma Group Ltd)
12360	50657020	Nephril 1mg Tablet (Pfizer Ltd)
26275	75814020	Nindaxa 2.5 tablets (Ashbourne Pharmaceuticals Ltd)
8058	63267020	Normetic Tablet (Abbott Laboratories Ltd)
18200	90629020	Olmesartan medoxomil 20mg / Hydrochlorothiazide 12.5mg tablets
18903	90631020	Olmesartan medoxomil 20mg / Hydrochlorothiazide 25mg tablets
43322	98337020	Olmesartan medoxomil 40mg / Hydrochlorothiazide 12.5mg tablets
46687	99769020	Olmesartan medoxomil with amlodipine and hydrochlorothiazide 20mg + 5mg + 12.5mg Tablet
46715	99773020	Olmesartan medoxomil with amlodipine and hydrochlorothiazide 40mg + 10mg + 12.5mg Tablet
55358	99777020	Olmesartan medoxomil with amlodipine and hydrochlorothiazide 40mg + 10mg + 25mg Tablet
46792	99771020	Olmesartan medoxomil with amlodipine and hydrochlorothiazide 40mg + 5mg + 12.5mg Tablet
47467	99775020	Olmesartan medoxomil with amlodipine and hydrochlorothiazide 40mg + 5mg + 25mg Tablet
29634	90635020	Olmetec Plus 20mg/12.5mg tablets (Daiichi Sankyo UK Ltd)
27520	90637020	Olmetec Plus 20mg/25mg tablets (Daiichi Sankyo UK Ltd)

For peer review only

43915	98339020	Olmetec Plus 40mg/12.5mg tablets (Daiichi Sankyo UK Ltd)
26256	78942020	Opumide 2.5mg Tablet (Opus Pharmaceuticals Ltd)
8673	73918020	Oxprenolol with cyclopenthiazide 160mg+0.25mg Modified-release tablet
50607	92909020	Perindopril arginine 2mg with Indapamide 625 micrograms tablet
48098	99501020	Perindopril arginine 4mg with Indapamide 1.25mg tablet
37978	94277020	Perindopril arginine 5mg / Indapamide 1.25mg tablets
6794	83957020	Perindopril erbumine 4mg / Indapamide 1.25mg tablets
56157	47939020	Perindopril tosilate 5mg / Indapamide 1.25mg tablets
21025	67090020	Prestim forte Tablet (LEO Pharma)
8623	52834020	Prestim Tablet (ICN Pharmaceuticals France S.A.)
59616	678020	Rawel XL 1.5mg tablets (Consilient Health Ltd)
17720	51466020	Saluric 500mg Tablet (Merck Sharp & Dohme Ltd)
31131	75349020	Spiro-co 25mg+25mg Tablet (IVAX Pharmaceuticals UK Ltd)
25505	75350020	Spiro-co 50mg+50mg Tablet (IVAX Pharmaceuticals UK Ltd)
8521	66716020	Spironolactone 25mg with hydroflumethiazide 25mg tablet
7961	66717020	Spironolactone 50mg with hydroflumethiazide 50mg tablet
8303	51854020	Tenavoid Tablet (Edwin Burgess Ltd)
26248	57165020	Tenchlor 100mg/25mg tablets (Teva UK Ltd)
31470	57164020	Tenchlor 50mg/12.5mg tablets (Teva UK Ltd)
1288	51857020	Tenoret 50mg/12.5mg tablets (AstraZeneca UK Ltd)
1124	54815020	Tenoretic 100mg/25mg tablets (AstraZeneca UK Ltd)
41861	95960020	Tensaid XL 1.5mg tablets (Generics (UK) Ltd)
25730	69274020	Timolol maleate with amiloride and hydrochlorothiazide Tablet
12517	67082020	Timolol maleate with bendroflumethiazide 20mg + 5mg Tablet
24280	80150020	Totaretic 100mg+25mg Tablet (C P Pharmaceuticals Ltd)
26741	80149020	Totaretic 50mg+12.5mg Tablet (C P Pharmaceuticals Ltd)
37294	67220020	Triamterene with chlortalidone 50mg + 25mg Tablet
9223	67204020	Triamterene with hydrochlorothiazide 50mg + 25mg Tablet
39447	95950020	Varbim XL 1.5mg tablets (Teva UK Ltd)
24008	72539020	Vasetic Tablet (Shire Pharmaceuticals Ltd)
7618	65442020	Xipamide 20mg tablets
19352	71715020	Xuret 0.5mg Tablet (Galen Ltd)
61846	45437020	Zaroxolyn 2.5mg tablets (IDIS)
6359	51463020	Zestoretic 10- 10mg+12.5mg Tablet (AstraZeneca UK Ltd)
39137	95875020	Zestoretic 10 tablets (AstraZeneca UK Ltd)
57539	1503020	Zestoretic 10 tablets (Sigma Pharmaceuticals Plc)
2982	51462020	Zestoretic 20- 20mg+12.5mg Tablet (AstraZeneca UK Ltd)
38995	95877020	Zestoretic 20 tablets (AstraZeneca UK Ltd)
26219	79843020	Zida-co 5mg+50mg Tablet (Opus Pharmaceuticals Ltd)

For peer review only

Centrally acting antihypertensives

Product code	GEM code	Description
60136	30024020	Clonidine 100micrograms/5ml oral solution
54467	28325020	Clonidine 300micrograms/24hours transdermal patches
27894	!1375104	CLONIDINE HYDROCHLORIDE
58090	30048020	Clonidine 75micrograms/5ml oral solution
28790	!1152103	CATAPRES
25088	!1152102	CATAPRES
27883	6602007	CATAPRES 15 MCG/ML INJ
20808	!1153101	CATAPRES PERLONGETS
2649	2258007	METHYLDOPA 250 MG CAP
25836	4723007	METHYLDOPA 200 MG TAB
49684	28322020	Clonidine 100micrograms/24hours transdermal patches
58529	18103020	Clonidine 200micrograms/24hours transdermal patches
6694	61497020	Clonidine 300microgram tablets
5289	72345020	Clonidine 250microgram modified-release capsules
16248	61500020	Clonidine 150micrograms/1ml solution for injection ampoules
2878	61496020	Clonidine 100microgram tablets
23380	48571020	Catapres 300microgram tablets (Boehringer Ingelheim Ltd)
4215	48570020	Catapres 100microgram tablets (Boehringer Ingelheim Ltd)
30293	58536020	Catapres 150micrograms/1ml solution for injection ampoules (Boehringer Ingelheim Ltd)
52555	19534020	Clonidine 50micrograms/5ml oral solution
53142	19536020	Clonidine 50micrograms/5ml oral suspension
8296	54917020	Catapres PL Perlongets 250microgram capsules (Boehringer Ingelheim Ltd)
24196	54853020	Dopamet 250mg Tablet (Berk Pharmaceuticals Ltd)
1707	59031020	Methyldopa 250mg tablets
3070	59032020	Methyldopa 500mg tablets
32913	50045020	Methyldopa 250mg tablets (Actavis UK Ltd)
28738	64556020	Methyldopa with hydrochlorothiazide Tablet
29570	54852020	Dopamet 125mg Tablet (Berk Pharmaceuticals Ltd)
7642	48071020	Aldomet 500mg Tablet (Merck Sharp & Dohme Ltd)
8033	48069020	Aldomet 125mg Tablet (Merck Sharp & Dohme Ltd)
7626	48070020	Aldomet 250mg Tablet (Merck Sharp & Dohme Ltd)
25275	51709020	Metalpha 500mg Tablet (Ashbourne Pharmaceuticals Ltd)
9225	77535020	Methyldopa 250mg Capsule
41661	50040020	Methyldopa 250mg Tablet (C P Pharmaceuticals Ltd)
43988	98722020	Aldomet 250mg tablets (Aspen Pharma Trading Ltd)
21346	64564020	Hydromet Tablet (MSD Thomas Morson Pharmaceuticals)
43989	98724020	Aldomet 500mg tablets (Aspen Pharma Trading Ltd)
14390	54403020	Aldomet 250mg/5ml Liquid (Merck Sharp & Dohme Ltd)
25289	54854020	Dopamet 500mg Tablet (Berk Pharmaceuticals Ltd)
23761	64552020	Methyldopa 250mg/5ml oral suspension
3049	59030020	Methyldopa 125mg tablets
18252	51708020	Metalpha 250mg Tablet (Ashbourne Pharmaceuticals Ltd)
26919	64553020	Methyldopa 50mg/ml Injection
7416	54406020	Aldomet 50mg/ml Injection (Merck Sharp & Dohme Ltd)
43531	68547020	Moxonidine 400microgram tablets (Sandoz Ltd)
9876	81117020	Physiotens 300microgram tablets (Abbott Healthcare Products Ltd)
60898	69282020	Moxonidine 200microgram tablets (Generics (UK) Ltd)
11177	81115020	Physiotens 200microgram tablets (Abbott Healthcare Products Ltd)
7174	81109020	Moxonidine 400microgram tablets

For peer review only

10253	81110020	Moxonidine 300microgram tablets
9749	81116020	Physiotens 400microgram tablets (Abbott Healthcare Products Ltd)
33322	68542020	Moxonidine 200microgram tablets (Sandoz Ltd)
40310	69037020	Moxonidine 200microgram tablets (Teva UK Ltd)
61036	45215020	Physiotens 300microgram tablets (Actavis UK Ltd)
4993	81108020	Moxonidine 200microgram tablets

For peer review only

Renin inhibitors

Product code	GEM code	Description
36878	93930020	Rasilez 150mg tablets (Novartis Pharmaceuticals UK Ltd)
36879	93932020	Rasilez 300mg tablets (Novartis Pharmaceuticals UK Ltd)
36909	93928020	Aliskiren 300mg tablets
36629	93926020	Aliskiren 150mg tablets

Vasodilators

Product code	GEM code	Description
25506	3361007	DIAZOXIDE 15 MG INJ
21749	5813007	HYDRALAZINE HCl 100 MG TAB
214	5817007	HYDRALAZINE 1 MG SYR
31971	4887007	HYDRALAZINE 6.25 MG SYR
4507	4885007	HYDRALAZINE HCl 12.5 MG TAB
23746	6300007	HYDRALAZINE HCl 10 MG TAB
22772	!8504292	SILDENAFIL
22819	!8504294	SILDENAFIL
22786	!8504293	SILDENAFIL
40527	95326020	Ambrisentan 10mg tablets
40528	95322020	Ambrisentan 5mg tablets
29561	78290020	Bosentan 125mg tablets
29560	78287020	Bosentan 62.5mg tablets
58632	78296020	Tracleer 125mg tablets (Actelion Pharmaceuticals UK Ltd)
47654	78293020	Tracleer 62.5mg tablets (Actelion Pharmaceuticals UK Ltd)
32267	62432020	Diazoxide 300mg/20ml solution for injection ampoules
13317	48198020	Apresoline 20mg powder for solution for injection ampoules (AMCo)
2362	48196020	Apresoline 25mg tablets (AMCo)
2680	48197020	Apresoline 50mg Tablet (Sovereign Medical Ltd)
18861	89188020	Hydralazine 10mg/5ml oral suspension
504	63242020	Hydralazine 20mg powder for solution for injection ampoules
573	58989020	Hydralazine 25mg tablets
31220	49792020	Hydralazine 25mg tablets (A A H Pharmaceuticals Ltd)
43500	49788020	Hydralazine 25mg tablets (Actavis UK Ltd)
1296	58990020	Hydralazine 50mg tablets
41639	49789020	Hydralazine 50mg tablets (Actavis UK Ltd)
59512	20076020	Hydralazine 50mg/5ml oral solution
61116	20078020	Hydralazine 50mg/5ml oral suspension
30341	87552020	Iloprost 20micrograms/2ml nebuliser liquid ampoules
29256	87554020	Ventavis 10micrograms/ml nebuliser solution 2ml ampoules (Schering Health Care Ltd)
53896	2181020	Iloprost 100micrograms/1ml solution for infusion ampoules
36840	93019020	Iloprost 10micrograms/1ml nebuliser liquid ampoules
51328	14602020	Iloprost 50micrograms/0.5ml solution for infusion ampoules
38519	93021020	Ventavis 10micrograms/ml nebuliser solution 1ml ampoules (Bayer Plc)
14495	64774020	Loniten 10mg tablets (Pfizer Ltd)
9697	64772020	Loniten 2.5mg tablets (Pfizer Ltd)
9463	64773020	Loniten 5mg tablets (Pfizer Ltd)
2968	64769020	Minoxidil 10mg tablets

For peer review only

2970	64767020	Minoxidil 2.5mg tablets
2967	64768020	Minoxidil 5mg tablets
46249	99911020	Revatio 10mg/12.5ml solution for injection vials (Pfizer Ltd)
30967	90581020	Revatio 20mg tablets (Pfizer Ltd)
46795	99909020	Sildenafil 10mg/12.5ml solution for injection vials
27137	90579020	Sildenafil 20mg tablets
45641	99463020	Sildenafil 40mg/50ml solution for injection vials
36612	66660020	Sodium nitroprusside 50mg powder for solution for infusion vials
47264	99180020	Adcirca 20mg tablets (Eli Lilly and Company Ltd)
6207	84307020	Tadalafil 20mg tablets

For peer review only

ISAC protocol 16_008R

Approved: 14th March 2016

PROTOCOL INFORMATION

In order to help ensure that protocols submitted for review contain adequate information for protocol evaluation, ISAC have produced guidance on the content of protocols for research using CPRD data. This guidance is available on the CPRD website (www.cprd.com/ISAC). All protocols using CPRD data which are submitted for review by ISAC must contain information on all the areas detailed below. If a specific area required by ISAC is not applicable to your protocol, please provide the justification underneath the relevant heading.

The protocol section (next page) has pre-defined headings and the protocol must be written using these headings. Additional headings are not acceptable; however, supplementary information may be placed in one or more of the appendices providing this information is essential and an appropriate reference to it is made within the protocol. Unless very short, codes lists should be placed in an Appendix. Applications will be regarded as invalid and returned to the applicant if any of the headings below are missing or if additional sections are included.

Please note that ISAC will not consider any application where the protocol exceeds 12 pages (excluding sections A-F of the application form and annexes). Annexes should be kept to a minimum and contain only vital information that could not be provided in the main protocol section. A font-size of at least 12 should be used. Protocols not exceeding 15 pages would be acceptable if ISAC has required a resubmission where additional information is requested.

Please note, your protocol will not be reviewed by ISAC if it falls short of the above requirements. You are advised to speak to the Secretariat if you have any queries.

Voluntary registration of ISAC approved studies:

Epidemiological studies are increasingly being included in registries of research around the world, including those primarily set up for clinical trials. To increase awareness amongst researchers of ongoing research, ISAC encourages voluntary registration of epidemiological research conducted using MHRA databases. This will not replace information on ISAC approved protocols that may be published on the CPRD website. It is for the applicant to determine the most appropriate registry for their study. Please inform the ISAC secretariat that you have registered a protocol and provide the location.

For peer review only

For peer review only - http://bmjopen.bmj.com/site/about/guidelines.xhtml

Protocol Section

A. Lay Summary (Max. 200 words)

Little is known about whether patients with moderately high blood pressure (mild hypertension) and a low risk of heart attack/stroke should be given medication to lower blood pressure. A definitive trial is unlikely to happen because it would be too expensive and require too many patients. Yet many guidelines encourage pharmacological treatment, and many patients receive it despite little evidence of benefit.

This study will examine the extent to which patients are prescribed pharmacological treatment for low risk mild hypertension, and explore the safety and effectiveness of this treatment using data from a large database of Primary Care records. Death from all causes will be compared in patients prescribed blood pressure lowering medication and those not prescribed medication, matched by characteristics such as age, sex, smoking status, prescription of other medications and history of disease. This approach will allow the effectiveness of pharmacological treatment to be examined in a large number of patients, followed-up for many years. This study will inform hypertension management guidelines, potentially leading to improved patient care.

B. Technical Summary (Max. 200 words)

Evidence to guide the pharmacological treatment of patients with uncomplicated (low cardiovascular disease risk), mild hypertension (blood pressure 140/90-159/99mmHg) is lacking. A definitive trial is unlikely due to the costs and numbers of patients required because of low event rates in this population. Yet many guidelines encourage treatment, and many patients are thought to receive it, despite little evidence of benefit.

This study will examine the extent to which patients are prescribed treatment for uncomplicated, mild hypertension in routine practice and the safety and efficacy of this treatment for using data from a large database of Primary Care records. The primary outcome of the study will be the rate of all-cause mortality in patients with uncomplicated mild hypertension prescribed antihypertensive treatment vs. those not prescribed therapy, matched using propensity scores estimating the likelihood of receiving treatment. Secondary outcomes will include the rate of cardiovascular morbidity/mortality, side effects to medication and rate of cancer in treated vs. non treated patients. These outcomes will be assessed using Cox proportional hazards modelling. The proportion of patients given lifestyle advice and/or pharmacological treatment for uncomplicated mild hypertension will also be estimated by year, and predictors of lifestyle advice/pharmacological treatment will be examined using logistic regression.

C. Objectives, Specific Aims and Rationale

Objective

Examine the extent to which patients are prescribed pharmacological treatment for uncomplicated, mild hypertension in routine practice (descriptive objective) and whether such treatment is safe and effective and reducing the risk of death and cardiovascular disease (hypothesis testing).

For peer review only - http://bmjopen.bmj.com/site/about/guidelines.xhtml

Specific aims

Descriptive objective

1. Establish the proportion of patients given lifestyle advice and/or pharmacological treatment for uncomplicated mild hypertension.
2. Examine the impact of the introduction of the Quality and Outcomes Framework (QOF) and NHS health check programme on the proportion of patients given lifestyle advice and/or pharmacological treatment for uncomplicated mild hypertension.
3. Examine factors which predict the likelihood of being given lifestyle advice/pharmacological treatment for uncomplicated mild hypertension in routine practice.

Hypothesis testing

1. **(Primary analysis)** Examine the impact of antihypertensive prescription on all-cause mortality in patients with uncomplicated (i.e. low cardiovascular disease risk mild hypertension (i.e. sustained blood pressure between 140/90-159/99mmHg).
2. Examine the impact of antihypertensive prescription on cardiovascular disease morbidity and mortality (myocardial infarction [MI], non-MI acute coronary syndrome, stroke, heart failure) in patients with uncomplicated mild hypertension.
3. Examine the impact of antihypertensive prescription on cancer morbitdity and mortality in patients with uncomplicated mild hypertension.
4. Examine the impact of antihypertensive prescription on hospital admissions related to side effects to medication (hypotension, syncope, bradycardia, electrolyte abnormalities, injurious falls and acute kidney injury/renal failure) in patients with uncomplicated mild hypertension.

Rationale

As discussed above and below, many patients with mild hypertension receive treatment, despite little evidence of benefit. The present proposal describes two distinct analyses using data from the Clinical Practice Research Datalink (CPRD). The first will examine the extent to which guidelines for the management of uncomplicated mild hypertension are followed and how this has changed over time. Factors which predict the likelihood of receiving antihypertensive treatment will be examined and this information will be used in a propensity score model to explore the safety and efficacy of antihypertensive treatment in these patients. These analyses will establish how many patients exist with uncomplicated mild hypertension, how it is currently managed in routine practice, and provide useful non-randomised data on treatment efficacy which will help guide future management decisions or inform whether future trial evidence is required.

D. Background

High blood pressure (hypertension) is a key risk factor for the development of cardiovascular disease[1] and a major cause of morbidity and mortality worldwide.[2] Classifications of hypertension are by definition arbitrary, but many guidelines recommend pharmacological treatment of blood pressure when it is sustained above 140/90mmHg, regardless of the underlying risk, end organ damage, diabetes or history of cardiovascular disease (table 1).[3-8] These recommendations have long been considered controversial,[9-13] particularly with regard to the treatment of uncomplicated (*i.e.* low cardiovascular disease risk) people with 'mild' hypertension (*i.e.* sustained blood pressure between 140/90-159/99mmHg).

There is little evidence to support initiation of pharmacological treatment in patients with uncomplicated mild hypertension and that which exists remains inconclusive. The recent SPRINT trial[14] did enrol a large

For peer review only - http://bmjopen.bmj.com/site/about/guidelines.xhtml

number of patients with mild hypertension (6,174 patients had a baseline systolic blood pressure <145mmHg) and compared a strategy of intensive blood pressure lowering against usual care. They observed significant reductions in death and cardiovascular mortality with intensive treatment, but all enrolled patients had at least one cardiovascular risk factor (in addition to high blood pressure) and thus would not be considered ‘uncomplicated’. A recent Cochrane review by Diao *et al.,*[15] examined 8,912 patients from four clinical trials investigating the benefits of treating uncomplicated mild hypertension compared to placebo. They found no significant reduction in mortality with treatment (RR 0.85, 95% CI 0.63-1.15), nor was any reduction in coronary artery disease, stroke or total cardiovascular events observed in a subgroup of 7,080 patients. However, both the authors of that review and subsequent commentators[10 15] point to a lack of power in previous trials and meta-analyses to show significant results.

More recently, Sundström and colleagues[16] attempted to resolve this issue using individual patient data from the Blood Pressure Lowering Treatment Triallists Collaboration and aggregate data from trials identified in a systematic review. Whilst they found significant associations between blood pressure reductions with treatment and stroke or total death, the population studied still included a significant proportion of patients with diabetes, and thus would be considered high risk (not ‘uncomplicated’) under current clinical guidelines.[17] There are simply too few randomised data in low risk patients to make adequately powered treatment comparisons. Indeed, based on data from the Cochrane Review, a trial of treatment vs. placebo for mild hypertension would need to recruit over 100,000 patients, followed up for 5 years, to achieve statistical significance. The cost of funding such a trial mean it is unlikely to ever be conducted.

As a result, clinical guidelines make important recommendations based on ‘expert opinion’ alone. These guidelines are predictably contradictory, with those in the UK promoting lifestyle modification in low risk patients[17] whilst guidelines from Europe[6] and America[4 7 8] encourage prescription of drug therapy. The impact of these recommendations is not trivial. A recent study from the UK[18] suggested the costs of treating patients with uncomplicated mild hypertension could reach £229 million in the first year alone, although these estimates are based on regional data extrapolated to national population estimates. Against this background, a number of policy changes have occurred in the UK over the past 10 years which are likely to have affected general practitioner’s treatment decisions. In particular, the introduction of the NHS health check in 2009[19] which will likely have increased the number of patients being diagnosed with uncomplicated mild hypertension, and QOF indicators briefly introduced in 2013 promoting treatment in this population.[20]

The proposed study will establish the national prevalence of uncomplicated mild hypertension in Primary Care, how it is currently managed in routine practice, and provide useful non-randomised data on treatment efficacy which will help guide future management decisions or inform whether future trial evidence is required and feasible.

E. Study Type

Hypothesis testing.

F. Study Design

Longitudinal cohort study.

For peer review only - http://bmjopen.bmj.com/site/about/guidelines.xhtml

For peer review only

G. Sample Size

Using estimates of treatment effect size from the recent Cochrane Review by Diao *et al.,* (risk ratio of 0.85 for all-cause mortality with treatment), and assuming a survival rate of 97.9% in the control group over 5 years, approximately 102,776 patients (51,388 in each group) and 1,600 events will be required to accurately define the relationship between antihypertensive treatment and all-cause mortality with 90% power and up to 20% withdrawal/crossover. The present analysis will utilise data from all available patients in the CPRD (with data linkage to ONS and HES) who fulfil the study eligibility criteria. We have examined the number of potentially eligible patients in a random sample of 20 general practices (413,279 patients) contributing to the CPRD database. Approximately 56,785 patients were identified with a diagnosis of hypertension, of which 8,791 (2.1%) fulfilled the study eligibility criteria (including 2,796 patients prescribed medication). The median length of follow-up in this population was 5.6 years. Extrapolating these data to the total number of patients within the CPRD database with linked data (10,022,053 patients) suggests a sample of 213,169 patients (including 67,803 patients on treatment) would be available.

H. Data Linkage Required (if applicable)

Data linkage to the ONS and Basic HES are required to define primary and secondary outcomes in the study. The ONS mortality register will be used to define the primary outcome of the study, all-cause mortality (and also censor follow-up at death). Specific linkages required will include data and ICD-10 coded cause of death (see appendix 2). Linkages to Basic Inpatient HES will be combined with data from the ONS to define secondary outcomes in the study, specifically death or admission to hospital with cardiovascular disease (defined previously), cancer or side effects to medication (hypotension, syncope, bradycardia, electrolyte abnormalities, injurious falls and acute kidney injury/renal failure) (see appendix 2). HES data will also be used to define patients eligibility for the study (e.g. previous stroke), and define the study population (e.g. ethnicity where unavailable in Primary Care records) – but only where it is recorded before the index date so as not to bias the completeness of data by those suffering an event during the study period. Data required from Basic Inpatient HES will include Primary diagnosis, secondary diagnosis, patient characteristics (e.g. sex, ethnicity), date of admission and date of discharge. All deaths and hospital admissions occurring after a patient's index date will be included. A linkage to the Index of Multiple Deprivation is required to acquire practice and patient level deciles of multiple deprivation. However, patients with missing IMD deprivation data will not be excluded, since these data will not be used to define any study outcomes.

Linkage to the MINAP dataset has not been requested due to limitations in the resources available for the study. However, this is not expected to a significant impact on the findings of the study, since:

- A recent study of national database recording of non-fatal MI found that CPRD and HES alone capture 92% of all events.[21]
- The MINAP dataset is unlikely to contribute any additional mortality data used for defining the primary outcome of this study which is not already captured in the ONS mortality register, CPRD or HES.
- Even if the databases used do not capture all outcomes of interest, we do not see any reason to expect the likelihood of an outcome being recorded in MINAP alone to be confounded by treatment status.

For peer review only - http://bmjopen.bmj.com/site/about/guidelines.xhtml

I. Study Population

Individual patient data will be extracted from the medical records of all patients registered at general practices contributing to the CPRD in the UK. Patients will be entered into the study on the 'index date', and exit at the first of the following time points to occur:

- date at which the most recent linked data are available from the CPRD (March 2015),
- Date of the most recent data upload from the practice to which a given patient is registered
- Date at which a given patient transfers out of a registered CPRD practice
- Date of death

Definition of the index date

The index will be defined as 12 months after the date of the third consecutive blood pressure reading between 140/90-159/99mmHg (readings must be taken within 12 months of each other) occurring after the study start date 01/01/1998. This date has been selected because it represents the date from which all relevant data linkages were first available (see below for details). Eligible patients will be those fulfilling the following criteria:

- Linked general practice, Hospital Episodes Statistics (HES) and Office for National Statistics (ONS) mortality records.
- Aged between 18-74 years.
- No Read code for stroke, myocardial infarction (MI), angina, coronary heart disease, peripheral vascular disease, heart failure, left ventricular hypertrophy, atrial fibrillation, diabetes or chronic kidney disease or family history of premature heart disease
- Three consecutive blood pressure readings between 140/90-159/99mmHg (within 12 months of each other).
- No record of any blood pressure lowering medication prescription in the 12 months prior to the third consecutive blood pressure reading between 140/90-159/99 mmHg.
- Classified as a CPRD 'acceptable patient'
- Registered to a CPRD practice classified as 'up-to-standard'

Exclusions will be defined using read coded diagnoses listed in the appendix (see appendix 2 and 3).

J. Selection of comparison group(s) or controls

Intervention group

The intervention in the present study will be sustained prescription of blood pressure lowering therapy to low risk patients with stage 1 hypertension. Patients considered in the treatment group will be those fulfilling the criteria above, who are initiated on blood pressure lowering treatment in the 12 months between the date of the third consecutive BP readings and the index date. The analysis will employ an intention-to-treat approach, so after initiation of treatment, patients will remain in the study/allocation until the last day of follow-up or until they are censored due to mortality, or cardiovascular morbidity.

Control group

Patients in the control group will be those fulfilling the eligibility criteria who were not initiated on blood pressure lowering treatment in the 12 months between the date of the third consecutive BP readings and the index date.Those prescribed therapy more than 12 months after the original index date will be included in the control group.

Patients eligible for the control and treatment groups will be matched using the index date and propensity scores, which reduce the bias introduced in observational studies because patients are not randomised to

For peer review only - http://bmjopen.bmj.com/site/about/guidelines.xhtml

their treatment allocation. Propensity scores indicate the likelihood a patient will be prescribed treatment on the basis of their known (pre-treatment) characteristics and other known information which might influence the decision to treat. This method is advantageous because it allows patients to be matched on multiple characteristics using a single variable (the propensity score), although this means associations between specific covariates and outcomes cannot be assessed.

K. Exposures, Outcomes and Covariates

Exposures

The exposure variable (blood pressure lowering treatment) in the primary analysis in this study will be defined as a prescription of an angiotensin-converting-enzyme inhibitor, angiotensin-II-receptor blocker, calcium channel blocker, thiazide or thiazide-like diuretic, beta-blocker, alpha-blocker or any other antihypertensive listed in the British National Formulary (see appendix 1). Reads codes for being offered lifestyle advice will also be extracted (see appendix 1).

Outcomes

The main aim of this study is to examine the safety and efficacy of treatment in uncomplicated, mild hypertensives. All-cause mortality has been chosen as the primary outcome for this analysis because it permits assessment of the possible benefits of treatment (reduction in death from cardiovascular disease) and the safety of treatment (in terms of risk of non-cardiovascular death) combined in a single outcome. Secondary outcomes will include:

Descriptive secondary outcomes

- The proportion of patients given lifestyle advice and/or pharmacological treatment for uncomplicated mild hypertension.
- The likelihood of patients being given lifestyle advice and/or pharmacological treatment for uncomplicated mild hypertension before and after the introduction of QOF and the NHS health check programme.
- Factors which predict the likelihood of being given lifestyle advice/ pharmacological treatment

Hypothesis testing secondary outcomes

- death or hospitalisation from major cardiovascular events (MI, non-MI acute coronary syndrome, stroke, heart failure or death from cardiovascular disease) using the same definition as that used in the SPRINT trial[14]
- death or hospitalisation from stroke
- death or hospitalisation from MI or non-MI acute coronary syndrome
- death or hospitalisation from cancer
- hospitalisation with suspected side effects to medication (hypotension, syncope, bradycardia, electrolyte abnormalities, injurious falls and acute kidney injury/renal failure)

Outcomes will be captured from hospital admissions (including date of admission and discharge and primary diagnosis), read coded diagnoses in the CPRD and death certificates occurring after the index date using linked data from the Basic Inpatient HES and ONS mortality register (for codes see appendix 2).

Covariates

Data relating to baseline patient characteristics (age, sex, ethnicity [where available], deprivation), lifestyle factors (smoking status [never, current, ex-smoker], excessive alcohol consumption [more than 6/8 units a day or 35/50 units a week for men/women], and BMI [where available]), pre-treatment blood pressure

For peer review only - http://bmjopen.bmj.com/site/about/guidelines.xhtml

readings (in the preceeding 12 months), non-cardiovascular related co-morbidities (rheumatoid arthritis, high total cholesterol [>5mmol/l]) and all statin/antiplatelet medications prescribed will be extracted from the medical records of eligible patients. For subgroup analyses taking into account a patient's apparent adherence to treatment, 'daily equivalent dose' data will be extracted from CPRD and the medication possession ratio (percentage of days when medication was available [MPR]) will be estimated from the sum of tablets prescribed, divided by the number of days of follow-up.

L. Data/ Statistical analysis

The primary analysis will use a propensity score-matched longitudinal cohort study design to examine the safety and efficacy of antihypertensive treatment. Secondary analyses will include an interrupted time-series analysis of prescriptions and lifestyle advice given to patients diagnosed with uncomplicated mild hypertension, comparing before and after the introduction of the NHS health check programme.

Descriptive objectives

Aim 1 – Descriptive analyses

Descriptive statistics will be used to estimate the proportion of patients being offered lifestyle advice or therapy following a diagnosis of uncomplicated mild hypertension (aim 1). Estimates will be adjusted for age and sex and results will be stratified by year of diagnosis.

Aim 2 – Interrupted time-series analysis

The impact of the introduction of the NHS health check programme (April 2009) and changes to QOF indicators (April 2013) and on prescription rates will be examined using an interrupted time-series analysis. Rates of pharmacological treatment and or lifestyle advice will be estimated before and after these specific policy changes and the overall level and slope of the rates in the pre and post policy change periods will be compared using segmented regression.

Aim 3 – Propensity score model

Predictors of lifestyle advice and antihypertensive medication prescription following a diagnosis of uncomplicated mild hypertension (defined as three consecutive blood pressure readings between 140/90-159/99mmHg within a 12 month period) will be explored in a logistic regression model examining the association between the outcome of interest and patients and practice characteristics: age, sex, ethnicity, blood pressure, deprivation (practice level), smoking status, BMI, number of existing antihypertensive medication prescriptions, presence of statin or antiplatelet prescriptions, the presence of rheumatoid arthritis or hypercholesterolemia, calendar year of the index date and the general practice to which the patient is registered. The model examining predictors of pharmacological treatment will be used to generate a propensity score matched cohort for analysis of the primary outcome. The success of propensity score matching will be examined by comparing the characteristics patients in each group pre- and post-matching using chi-squared tests and t-tests. To assess the reduction in bias achieved by propensity score matching, the proportional reduction in the difference between each patient characteristic in the treatment and control groups will estimated and compared pre- and post-matching.

Hypothesis testing

Aim 4 - Primary analysis

Using an intention-to-treat approach, the efficacy of antihypertensive treatment will be examined with Cox proportional hazards modelling, comparing all-cause mortality in those prescribed (any) antihypertensive treatment vs. those not prescribed treatment. Where hazards are not proportional, non-parametric

For peer review only - http://bmjopen.bmj.com/site/about/guidelines.xhtml

methods such as inverse probability weights will be considered. Kaplan-Meier plots will be produced to display the cumulative incidence of all-cause mortality in the treatment group compared to the control group. Hazard ratios will be generated and adjusted by any other factors, unbalanced at baseline, not already included in the initial propensity score matching.

Aims 5-7 – Secondary analyses

Where appropriate (and sufficiently powered to detect an effect), hazard ratios will be calculated using Cox proportional hazards modelling, examining the association between treatment status and secondary endpoints in the study: major cardiovascular disease event, hospitalisation and/or death from stroke, or MI or non-MI acute coronary syndrome and pre-specified side effects to medication.

Subgroup analyses

Subgroup analyses will be conducted to examine the impact of patient adherence to medication on the effectiveness of treatment. We will stratify our analysis by quantiles of medication possession ratio and the treatment effects will be compared between groups. Other pre-specified subgroup analyses will include comparison of the treatment effect stratified by age (defined according to 10 year age bands, where possible), antihypertensive drug class (if sufficiently powered) and in males vs. females.

M. Plan for addressing confounding

The validity of propensity score matching will be examined using 'negative controls': the impact of antihypertensive treatment on an outcome not known to be affected by such treatment. Here, the impact of antihypertensive therapy on hospitalisation and/or death from cancer will be studied as the negative control (aim 6). If treatment with antihypertensives has a significant impact on this outcome, we can conclude there is something missing in the propensity score (i.e. an unmeasurable factor of propensity for treatment such as being generally unwell or an unhealthy lifestyle) causing an imbalance between the treatment and control groups, rather than a true treatment effect.

N. Plan for addressing missing data

There is potential for missing data in this study, particularly with variables such as ethnicity which are recorded with varying degrees of accuracy in routine practice. Because this analysis is focused on treatment of uncomplicated mild hypertension, accurate selection of the sample population is important and therefore any patients with insufficient data available to define their blood pressure status at baseline (index date) will be excluded. Where there is no record of blood pressure lowering, statin or antiplatelet treatment, it will be assumed the patients were not exposed to blood pressure lowering, statin or antiplatelet treatment.

Patient eligibility includes only using 'acceptable patients' in the analysis, and therefore there is no need to impute age and sex. Where there is no record of smoking history or alcohol consumption, patients will be assumed to be non-smokers and non-drinkers. Likewise, those with no record of rheumatoid arthritis or hypercholesterolemia will be assumed to have no history of these conditions. All other covariates used in the propensity score model will be imputed using multiple imputation. Ethnicity and IMD will be treated as categorical variable in the imputation model. BMI will be treated as a continuous variable.

The primary analysis examining the efficacy of treatment will be conducted by intention-to-treat, and patients moving practice after the index date (and therefore being lost to follow-up) will be censored at the point at which they are no longer active in the database.

For peer review only - http://bmjopen.bmj.com/site/about/guidelines.xhtml

O. Limitations of the study design, data sources and analytical methods

This study will use an observational cohort design, and as such, there is an inherent selection bias of patients in both intervention and control groups. The impact of this bias will be limited by propensity score matching but such an approach assumes that all factors influencing the propensity for medication prescription have been measured and accounted for within the score. In the present study, we will examine the validity of this assumption by studying the impact of treatment on a negative control: hospitalisation and/or death from cancer. We will conclude the assumption has been met if antihypertensive treatment is shown to have no impact on cancer outcomes, as would be expected from previous trial evidence.

Previous randomised studies examining the efficacy of treatment for uncomplicated stage 1 hypertension have been underpowered to demonstrate a true treatment effect. This study will use data from the largest linked primary care database in the world with a potential sample population of approximately 210,000 patients, sufficient to power the study for the primary and subgroup analyses. In pre-specified subgroup analyses, we will examine the impact of potential confounding factors such as patient adherence to medication.

Strategy for dealing with potential errors resulting from multiple testing

This study includes one descriptive objective to examine the extent to which patients are prescribed pharmacological treatment for uncomplicated, mild hypertension and one hypothesis testing objective to examine whether such treatment is safe and effective at reducing the risk of death and cardiovascular disease. To avoid potential errors arising from multiple testing, a primary outcome has been clearly defined as the impact of antihypertensive treatment on all-cause mortality. Secondary outcomes will examine the impact of antihypertensive treatment on major cardiovascular events (composite endpoint), death or hospitalisation from stroke, death or hospitalisation from MI or non-MI acute coronary syndrome, death or hospitalisation from cancer or hospitalisation with suspected side effects to medication (hypotension, syncope, bradycardia, electrolyte abnormalities, injurious falls and acute kidney injury/renal failure). The primary outcome will be given priority in the final analysis write-up and any related reports and presentations.

Primary and secondary outcomes will be defined using ONS, inpatient HES and read-coded primary care data. The accuracy of such outcome data has been examined previously in patients with acute myocardial infarction[21] and whilst recording of risk factor and co-morbid information was consistent across primary care, hospital admissions and disease registry records, the crude incidence of acute myocardial infarction was underestimated by up to 50% if only one data source was used, compared with using all three sources. The use of linked CPRD data in the present study is therefore crucial to ensure outcome data are ascertained accurately.

P. Patient or user group involvement (if applicable)

We have not carried out specific PPI for this particular project, although PPI work we have recently conducted for similar studies is relevant. In particular, we have conducted focus groups to examine patient views on blood pressure treatment and primary prevention of cardiovascular disease. Specific issues highlighted included that healthy older individuals, like many others, sometimes take their state of good health for granted and few considered that they might be at risk of strokes or heart attacks. The haphazard nature of blood pressure/cholesterol checks by their GP seemed confusing and many opted for an attitude of fatalism over future health. They were often informed by health experiences of friends and popular media opinion and were wary of the potential side-effects of medication (hence preferred not to take it).

For peer review only - http://bmjopen.bmj.com/site/about/guidelines.xhtml

These issues are pertinent in the population of interest, and evidence provided by the present analysis will support shared decision making between patients and doctors as well as informing future cardiovascular disease prevention programmes and policy.

Q. Plans for disseminating and communicating study results, including the presence or absence of any restrictions on the extent and timing of publication

We expect at least two publications arising from this research: one describing the prevalence of pharmacological treatment and lifestyle advice for uncomplicated mild hypertension, and how this has changed over time. The second will explore the safety and efficacy of treatment for mild hypertension. A third publication may be possible exploring the efficacy of treatment in predefined sub-groups in detail. Findings will be presented at scientific meetings and published in journals at the earliest possible convenience. We will endeavour to publish our study findings in lay format on our University website and through lay and social media where appropriate.

For peer review only

For peer review only - http://bmjopen.bmj.com/site/about/guidelines.xhtml

References

1. Lewington S, Clarke R, Qizilbash N, et al. Age-specific relevance of usual blood pressure to vascular mortality: a meta-analysis of individual data for one million adults in 61 prospective studies. *Lancet* 2002;360(9349):1903-13. [published Online First: 2002/12/21]

2. Lozano R, Naghavi M, Foreman K, et al. Global and regional mortality from 235 causes of death for 20 age groups in 1990 and 2010: a systematic analysis for the Global Burden of Disease Study 2010. *Lancet* 2012;380(9859):2095-128. doi: 10.1016/s0140-6736(12)61728-0 [published Online First: 2012/12/19]

3. Whitworth JA. 2003 World Health Organization (WHO)/International Society of Hypertension (ISH) statement on management of hypertension. *Journal of hypertension* 2003;21(11):1983-92. doi: 10.1097/01.hjh.0000084751.37215.d2 [published Online First: 2003/11/05]

4. Sanchez RA, Ayala M, Baglivo H, et al. Latin American guidelines on hypertension. Latin American Expert Group. *Journal of hypertension* 2009;27(5):905-22. doi: 10.1097/HJH.0b013e32832aa6d2 [published Online First: 2009/04/08]

5. Ogihara T, Kikuchi K, Matsuoka H, et al. The Japanese Society of Hypertension Guidelines for the Management of Hypertension (JSH 2009). *Hypertension research : official journal of the Japanese Society of Hypertension* 2009;32(1):3-107. [published Online First: 2009/03/21]

6. Mancia G, Fagard R, Narkiewicz K, et al. 2013 ESH/ESC Guidelines for the management of arterial hypertension: the Task Force for the management of arterial hypertension of the European Society of Hypertension (ESH) and of the European Society of Cardiology (ESC). *Journal of hypertension* 2013;31(7):1281-357. doi: 10.1097/01.hjh.0000431740.32696.cc [published Online First: 2013/07/03]

7. James PA, Oparil S, Carter BL, et al. 2014 evidence-based guideline for the management of high blood pressure in adults: report from the panel members appointed to the Eighth Joint National Committee (JNC 8). *JAMA : the journal of the American Medical Association* 2014;311(5):507-20. doi: 10.1001/jama.2013.284427 [published Online First: 2013/12/20]

8. Hackam DG, Quinn RR, Ravani P, et al. The 2013 Canadian Hypertension Education Program recommendations for blood pressure measurement, diagnosis, assessment of risk, prevention, and treatment of hypertension. *The Canadian journal of cardiology* 2013;29(5):528-42. doi: 10.1016/j.cjca.2013.01.005 [published Online First: 2013/04/02]

9. Toth PJ, Horwitz RI. Conflicting clinical trials and the uncertainty of treating mild hypertension. *The American journal of medicine* 1983;75(3):482-8. [published Online First: 1983/09/01]

10. Tavares A. Pharmacotherapy for mild hypertension. *Sao Paulo medical journal = Revista paulista de medicina* 2012;130(6):417-8. [published Online First: 2013/01/23]

11. Ramsay LE, ul Haq I, Yeo WW, et al. Interpretation of prospective trials in hypertension: do treatment guidelines accurately reflect current evidence? *Journal of hypertension Supplement : official journal of the International Society of Hypertension* 1996;14(5):S187-94. [published Online First: 1996/12/01]

12. Heath I. Waste and harm in the treatment of mild hypertension. *JAMA internal medicine* 2013;173(11):956-7. doi: 10.1001/jamainternmed.2013.970 [published Online First: 2013/05/24]

13. Hart JT. Historical footnote on the treatment of mild hypertension. *BMJ (Clinical research ed)* 2012;345:e6297. doi: 10.1136/bmj.e6297 [published Online First: 2012/09/26]

For peer review only

For peer review only - http://bmjopen.bmj.com/site/about/guidelines.xhtml

14. Wright JT, Jr., Williamson JD, Whelton PK, et al. A Randomized Trial of Intensive versus Standard Blood-Pressure Control. *The New England journal of medicine* 2015;373(22):2103-16. doi: 10.1056/NEJMoa1511939 [published Online First: 2015/11/10]

15. Diao D, Wright JM, Cundiff DK, et al. Pharmacotherapy for mild hypertension. *The Cochrane database of systematic reviews* 2012;8:Cd006742. doi: 10.1002/14651858.CD006742.pub2 [published Online First: 2012/08/17]

16. Sundstrom J, Arima H, Jackson R, et al. Effects of blood pressure reduction in mild hypertension: a systematic review and meta-analysis. *Annals of internal medicine* 2015;162(3):184-91. doi: 10.7326/m14-0773 [published Online First: 2014/12/23]

17. National Clinical Guideline Centre. Hypertension: clinical management of primary hypertension in adults; Clinical guideline 127. London: Royal College of Physicians (UK), 2011.

18. Sheppard JP, Fletcher K, McManus RJ, et al. Prevalence and costs of treating uncomplicated stage 1 hypertension in primary care: a cross-sectional analysis. *The British journal of general practice : the journal of the Royal College of General Practitioners* 2014;64(627):e641-8. doi: 10.3399/bjgp14X681817 [published Online First: 2014/10/01]

19. Public Health England. NHS Health Check: our approach to the evidence. https://www.gov.uk/government/uploads/system/uploads/attachment_data/file/224537/NHS_Health_Check_our_approach_to_the_evidence_v2.pdf: NHS England, 2013.

20. General practitioners committee Ne. 2013/14 general medical services (GMS) contract quality and outcomes framework (QOF): Guidence for GMS contract 2013/14: NHS Employers, 2013.

21. Herrett E, Shah AD, Boggon R, et al. Completeness and diagnostic validity of recording acute myocardial infarction events in primary care, hospital care, disease registry, and national mortality records: cohort study. *BMJ (Clinical research ed)* 2013;346:f2350. doi: 10.1136/bmj.f2350 [published Online First: 2013/05/23]

For peer review only

For peer review only - http://bmjopen.bmj.com/site/about/guidelines.xhtml

The RECORD statement – checklist of items, extended from the STROBE statement, that should be reported in observational studies using routinely collected health data.

	Item No.	STROBE items	Location in manuscript where items are reported	RECORD items	Location in manuscript where items are reported
**Title and abstract**					
	1	(a) Indicate the study's design with a commonly used term in the title or the abstract (b) Provide in the abstract an informative and balanced summary of what was done and what was found		RECORD 1.1: The type of data used should be specified in the title or abstract. When possible, the name of the databases used should be included.<br><br>RECORD 1.2: If applicable, the geographic region and timeframe within which the study took place should be reported in the title or abstract.<br><br>RECORD 1.3: If linkage between databases was conducted for the study, this should be clearly stated in the title or abstract.	p1<br><br>p2<br><br>p2
**Introduction**					
Background rationale	2	Explain the scientific background and rationale for the investigation being reported			P4
Objectives	3	State specific objectives, including any prespecified hypotheses			P4
**Methods**					
Study Design	4	Present key elements of study design early in the paper			P4
Setting	5	Describe the setting, locations, and relevant dates, including periods of recruitment, exposure, follow-up, and data collection			P4-5
Participants	6	*(a) Cohort study* - Give the eligibility criteria, and the		RECORD 6.1: The methods of study population selection (such as codes or	P4-5

For peer review only - http://bmjopen.bmj.com/site/about/guidelines.xhtml

		sources and methods of selection of participants. Describe methods of follow-up<br>*Case-control study* - Give the eligibility criteria, and the sources and methods of case ascertainment and control selection. Give the rationale for the choice of cases and controls<br>*Cross-sectional study* - Give the eligibility criteria, and the sources and methods of selection of participants<br><br>*(b) Cohort study* - For matched studies, give matching criteria and number of exposed and unexposed<br>*Case-control study* - For matched studies, give matching criteria and the number of controls per case		algorithms used to identify subjects) should be listed in detail. If this is not possible, an explanation should be provided.<br><br>RECORD 6.2: Any validation studies of the codes or algorithms used to select the population should be referenced. If validation was conducted for this study and not published elsewhere, detailed methods and results should be provided.<br><br>RECORD 6.3: If the study involved linkage of databases, consider use of a flow diagram or other graphical display to demonstrate the data linkage process, including the number of individuals with linked data at each stage.	 <br><br><br>n/a<br><br><br><br><br><br><br>Figure 1
Variables	7	Clearly define all outcomes, exposures, predictors, potential confounders, and effect modifiers. Give diagnostic criteria, if applicable.		RECORD 7.1: A complete list of codes and algorithms used to classify exposures, outcomes, confounders, and effect modifiers should be provided. If these cannot be reported, an explanation should be provided.	P5
Data sources/ measurement	8	For each variable of interest, give sources of data and details of methods of assessment (measurement).<br>Describe comparability of assessment methods if there is more than one group			P5
Bias	9	Describe any efforts to address potential sources of bias			P6
Study size	10	Explain how the study size was			P5

For peer review only

		arrived at			
Quantitative variables	11	Explain how quantitative variables were handled in the analyses. If applicable, describe which groupings were chosen, and why			P5
Statistical methods	12	(a) Describe all statistical methods, including those used to control for confounding<br>(b) Describe any methods used to examine subgroups and interactions<br>(c) Explain how missing data were addressed<br>(d) *Cohort study* - If applicable, explain how loss to follow-up was addressed<br>*Case-control study* - If applicable, explain how matching of cases and controls was addressed<br>*Cross-sectional study* - If applicable, describe analytical methods taking account of sampling strategy<br>(e) Describe any sensitivity analyses			p6
Data access and cleaning methods		..		RECORD 12.1: Authors should describe the extent to which the investigators had access to the database population used to create the study population.<br><br>RECORD 12.2: Authors should provide information on the data cleaning methods used in the study.	P6<br><br>P6
Linkage		..		RECORD 12.3: State whether the study included person-level, institutional-	P7

For peer review only

				level, or other data linkage across two or more databases. The methods of linkage and methods of linkage quality evaluation should be provided.	
**Results**					
Participants	13	(a) Report the numbers of individuals at each stage of the study (*e.g.*, numbers potentially eligible, examined for eligibility, confirmed eligible, included in the study, completing follow-up, and analysed)<br>(b) Give reasons for non-participation at each stage.<br>(c) Consider use of a flow diagram		RECORD 13.1: Describe in detail the selection of the persons included in the study (*i.e.*, study population selection) including filtering based on data quality, data availability and linkage. The selection of included persons can be described in the text and/or by means of the study flow diagram.	p7 and figure 1
Descriptive data	14	(a) Give characteristics of study participants (*e.g.*, demographic, clinical, social) and information on exposures and potential confounders<br>(b) Indicate the number of participants with missing data for each variable of interest<br>(c) *Cohort study* - summarise follow-up time (*e.g.*, average and total amount)			p7, table 3
Outcome data	15	*Cohort study* - Report numbers of outcome events or summary measures over time<br>*Case-control study* - Report numbers in each exposure category, or summary measures of exposure<br>*Cross-sectional study* - Report numbers of outcome events or summary measures			p7-8
Main results	16	(a) Give unadjusted estimates			P7-8

For peer review only

		and, if applicable, confounder-adjusted estimates and their precision (e.g., 95% confidence interval). Make clear which confounders were adjusted for and why they were included<br>(b) Report category boundaries when continuous variables were categorized<br>(c) If relevant, consider translating estimates of relative risk into absolute risk for a meaningful time period			
Other analyses	17	Report other analyses done—e.g., analyses of subgroups and interactions, and sensitivity analyses			p8
**Discussion**					
Key results	18	Summarise key results with reference to study objectives			P8
Limitations	19	Discuss limitations of the study, taking into account sources of potential bias or imprecision. Discuss both direction and magnitude of any potential bias		RECORD 19.1: Discuss the implications of using data that were not created or collected to answer the specific research question(s). Include discussion of misclassification bias, unmeasured confounding, missing data, and changing eligibility over time, as they pertain to the study being reported.	P8-9
Interpretation	20	Give a cautious overall interpretation of results considering objectives, limitations, multiplicity of analyses, results from similar studies, and other relevant evidence			P9-10
Generalisability	21	Discuss the generalisability (external validity) of the study results			P9-10

For peer review only

Other Information					
Funding	22	Give the source of funding and the role of the funders for the present study and, if applicable, for the original study on which the present article is based			p11
Accessibility of protocol, raw data, and programming code		..		RECORD 22.1: Authors should provide information on how to access any supplemental information such as the study protocol, raw data, or programming code.	Online appendix

*Reference: Benchimol EI, Smeeth L, Guttmann A, Harron K, Moher D, Petersen I, Sørensen HT, von Elm E, Langan SM, the RECORD Working Committee. The REporting of studies Conducted using Observational Routinely-collected health Data (RECORD) Statement. *PLoS Medicine* 2015; in press.

*Checklist is protected under Creative Commons Attribution (CC BY) license.

For peer review only

BMJ Open

Association of guideline and policy changes with incidence of lifestyle advice and treatment for uncomplicated mild hypertension in Primary Care: a longitudinal cohort study in the Clinical Practice Research Datalink

Journal:	*BMJ Open*
Manuscript ID	bmjopen-2018-021827.R1
Article Type:	Research
Date Submitted by the Author:	02-May-2018

Complete List of Authors:	Sheppard, James; University of Oxford, Nuffield Department of Primary Care Health Sciences<br>Stevens, Sarah; University of Oxford, Nuffield Department of Primary Care Health Sciences<br>Stevens, Richard; University of Oxford, Nuffield Dept Primary Care Health Sciences<br>Mant, Jonathan; University of Cambridge, General Practice and Primary Care Research Unit<br>Martin, Una; University of Birmingham, Institute of Clinical Sciences<br>Hobbs, Richard; University of Oxford, Dept of Primary Health Care<br>McManus, Richard; University of Oxford, Dept of Primary Care Health Sciences

**Primary Subject Heading**:	General practice / Family practice
Secondary Subject Heading:	Cardiovascular medicine, Health services research
Keywords:	Blood pressure, Interrupted time series, Electronic health records, Cardiovascular disease prevention, general practice

SCHOLARONE™
Manuscripts

Association of guideline and policy changes with incidence of lifestyle advice and treatment for uncomplicated mild hypertension in Primary Care: a longitudinal cohort study in the Clinical Practice Research Datalink

James P Sheppard, Ph.D,[1] Sarah Stevens, M.Sc,[1] Richard Stevens, Ph.D,[1] Jonathan Mant, MD,[2] Una Martin FRCP,[3] FD Richard Hobbs, FRCGP,[1] and Richard J McManus, FRCGP[1]

[1]University of Oxford, Oxford, UK
[2]University of Cambridge, Cambridge, UK
[3]University of Birmingham, Birmingham, UK

Corresponding author: James P Sheppard
Email: james.sheppard@phc.ox.ac.uk
Telephone: +44 1865 617192
Address: Nuffield Department of Primary Care Health Sciences, Radcliffe Observatory Quarter, University of Oxford, Oxford, OX2 6GG, UK

Word count: 3,423 (excluding title page, abstract, references, tables and figures)
Number of tables: 3
Number of figures: 3
Number of appendices: 4

For peer review only - http://bmjopen.bmj.com/site/about/guidelines.xhtml

Abstract

Objectives: Evidence to support initiation of pharmacological treatment in patients with uncomplicated (low risk) mild hypertension is inconclusive. As such, clinical guidelines are contradictory and healthcare policy has changed regularly. The aim of this study was to determine the incidence of lifestyle advice and drug therapy in this population and whether secular trends were associated with policy changes.

Design: Longitudinal cohort study.

Setting: Primary Care practices contributing to the Clinical Practice Research Datalink in England.

Participants: Data were extracted from the linked electronic health records of patients aged 18-74 years, with stage 1 hypertension (blood pressure between 140/90-159/99mmHg), no cardiovascular disease (CVD) risk factors and no treatment, from 1998-2015. Patients exited if follow-up records became unavailable, they progressed to stage 2 hypertension, developed a CVD risk factor or received lifestyle advice/treatment.

Primary outcome measures: The association between policy changes and incidence of lifestyle advice or treatment, examined using an interrupted time series analysis.

Results: A total of 108,843 patients were defined as having uncomplicated mild hypertension (mean age 51.9±12.9 years, 60.0% female). Patients were in the study spent for a median 2.6 years (IQR 0.9, 5.5), after which 12.2% (95%CI 12.0%-12.4%) were given lifestyle advice, 29.9% (95%CI 29.7%-30.2%) were prescribed medication and 19.4% (95%CI 19.2%-19.6%) were given both. The introduction of the Quality Outcomes Framework (QOF) and subsequent changes to QOF indicators were followed by significant increases in the incidence of lifestyle advice. Treatment prescriptions decreased slightly over time, but were not associated with policy changes.

Conclusions: Despite secular trends that accord with UK guidance, many patients are still prescribed treatment for mild hypertension. Adequately powered studies are needed to determine if this is appropriate.

Word count: 275

Keywords: Blood pressure, Interrupted time series, electronic health records, cardiovascular disease prevention, general practice

For peer review only - http://bmjopen.bmj.com/site/about/guidelines.xhtml

Strengths and weaknesses of this study

- This study examined electronic health records from a large, nationally representative sample of the UK population.
- Analyses were undertaken according to a pre-specified protocol which was reviewed and approved by an independent scientific advisory committee.
- Mild hypertension was defined consistently according to blood pressure readings, meaning that those with a diagnostic code for hypertension but no associated blood pressure values may have been excluded.
- Low risk patients were defined according to individual risk factors, rather than an established risk calculator due to large amounts of missing data (e.g. for cholesterol, BMI), potentially underestimating the number of truly low risk patients available for analysis.
- The incidence of lifestyle advice was defined according to clinical codes but it was not possible to identify patients given lifestyle advice where it was either not coded or where it was only recorded in the free text consultation notes.

For peer review only

For peer review only - http://bmjopen.bmj.com/site/about/guidelines.xhtml

Introduction

High blood pressure (hypertension) is a key risk factor for the development of cardiovascular disease,[1] a major cause of mortality worldwide.[2] The threshold for hypertension is a sustained blood pressure above 140/90mmHg, and many guidelines recommend pharmacological treatment is initiated immediately at this level, or after a period of unsuccessful lifestyle modification, regardless of the underlying risk of cardiovascular disease.[3-8] These recommendations are considered controversial, particularly with regard to treatment of uncomplicated (*i.e.* low cardiovascular disease risk) people with 'mild' hypertension (*i.e.* sustained blood pressure between 140/90-159/99mmHg [stage 1]).[9-14]

There is little evidence to support initiation of pharmacological treatment in patients with uncomplicated mild hypertension. A Cochrane review[15] of trials in this population found no reduction in mortality or cardiovascular events with treatment, but was underpowered to detect significant differences.[10 15] More recent studies[16 17] have shown benefit with treatment in patients with mild hypertension. However, these included a significant proportion of patients with diabetes or at least one other cardiovascular risk factor and therefore participants would not be considered 'uncomplicated' under current clinical guidelines.[18]

Clinical guidelines often make recommendations based on 'expert opinion' where evidence is lacking or inconsistent. Such guidelines are predictably contradictory, with those in the UK promoting lifestyle modification in low risk patients[18] whilst guidelines from Europe[6] and the US[4 7 8] encourage prescription of drug therapy. Recently, an international expert consultation recommended early initiation of blood pressure lowering treatment with lifestyle advice reserved only for patients with grade 1 isolated hypertension.[14] Against this background, a number of guideline and policy changes have occurred in the UK over the past 10 years which may have affected general practitioner's treatment decisions. In particular, the introduction of a nationwide cardiovascular risk screening programme (the NHS health check) in 2009,[19] and pay-for-performance indicators (the Quality and Outcomes Framework [QOF]), which promoted both more lifestyle advice and stricter treatment targets in this population.[20]

The aim of this study was to use routine patient records from the Clinical Practice Research Datalink (CPRD)[21] to establish the proportion of patients given lifestyle advice and/or pharmacological treatment for uncomplicated mild hypertension in Primary Care and the association of policy with changes to these proportions over time.

Methods

Design

This was a longitudinal cohort study in conducted in Primary Care from 1998 to 2015 using data from the Clinical Practice Research Datalink (CPRD), a database of electronic health records from England.[21] The incidence of lifestyle advice and treatment prescriptions was estimated by month, adjusted for age and sex. The association of guideline and policy changes was examined using an interrupted time series analysis with introduction of the NHS health check[19] and changes in QOF indicators[20] defined as pre-specified break points. The CPRD population has been shown previously to approximate to the UK population.[21]

For peer review only - http://bmjopen.bmj.com/site/about/guidelines.xhtml

Study population

Individual patient data were extracted from the medical records of all patients registered at general practices contributing to the CPRD in England. Patients were entered into the study on the date of the third consecutive blood pressure reading between 140/90-159/99mmHg occurring after the study start date 01/01/1998. The start date was chosen because it represents the date from which all relevant data linkages were first available. Patients exited the study if they had three consecutive blood pressure readings above 160/100mmHg (i.e. progressed to stage 2 hypertension), developed a risk factor for cardiovascular disease, received lifestyle advice or treatment or follow-up records became unavailable (patient or practice left the CPRD) (table 1). The last day of follow-up for those remaining in the study was 30th September 2015 (last day of follow-up in linked data).

Outcomes

The primary outcome of this study was evidence that lifestyle advice and/or pharmacological treatment had been given during follow-up. Lifestyle advice was defined as Read code for any lifestyle advice relating to smoking, alcohol, diet, hypertension, physical activity, weight, oral health or lipid disorders (see online appendix, eTable 1). Pharmacological treatment was defined as a coded prescription of an angiotensin-converting-enzyme inhibitor, angiotensin-II-receptor blocker, calcium channel blocker, thiazide or thiazide-like diuretic, beta-blocker, alpha-blocker or any other antihypertensive listed in the British National Formulary (see online appendix, eTable 2).

Covariates

Data relating to baseline patient characteristics (age, sex, ethnicity, [patient level] deprivation), lifestyle factors (smoking status [never, current, ex-smoker], alcohol consumption [units per week], and BMI), pre-treatment blood pressure readings (in the preceding 12 months), related co-morbidities (rheumatoid arthritis, hypercholesterolemia [Read code for hypercholesterolemia or most recent total cholesterol value ≥7.5mmol/l]) and all prescribed statin/antiplatelet medications were extracted from the CPRD database and used to define the study population.

Quantitative data

Ethnicity was classified into white, black, South Asian, mixed race, other and unknown groups. The Index of Multiple Deprivation (IMD) was provided as quintiles of deprivation in England, with the highest quintile indicating the highest level of deprivation. Smoking status was defined as never, previous, or current smoker. Those with unknown smoking status were assumed to have never smoked. Where there was no record of blood pressure lowering, statin or antiplatelet treatment, it was assumed that patients were not prescribed treatment.

Only patients with records deemed 'acceptable for research purposes' by CPRD were included in the analysis, and hence age and sex information was complete. Where there was no record of alcohol consumption, rheumatoid arthritis or hypercholesterolemia, patients were assumed to be non-drinkers and no history of these conditions. Where there was no record of blood pressure lowering, statin or antiplatelet treatment, it was assumed that patients were not exposed to any treatment. A total of 91 patients (0.001%) were missing IMD data and were excluded. To enable calculation of a cardiovascular risk score in sensitivity analysis, missing values for cholesterol and BMI were simulated once, assuming a normal distribution, and using means and standard deviations in each age-sex strata from the 2011 Health Survey for England.[22]

For peer review only

For peer review only - http://bmjopen.bmj.com/site/about/guidelines.xhtml

Sample size

As a descriptive study, no formal sample size calculation was required. All patients from the CPRD fulfilling the eligibility criteria were included.

Statistical analysis

Descriptive statistics were used to describe the study population and estimate the proportion of patients being offered lifestyle advice or therapy (or both) following a diagnosis of uncomplicated mild hypertension.

The impact of the introduction of the NHS health check programme in April 2009[19] (month 111) and changes to QOF indicators (April 2009 and 2013 [month 159])[20] (see table 2) on lifestyle advice and treatment prescription rates were examined using an interrupted time series analysis. A two year run-in phase (1998-1999) was used, where patients could enter and leave the cohort, but incidence rates were not estimated. This was to ensure the cohort accumulated sufficient numbers of patients to enable accurate estimates of monthly incidence in the subsequent time period after the analysis start date (January 1st 2000). The slope of the monthly rate of lifestyle advice and treatment over time was estimated (189 months in total), before policy change was introduced (months 111 and 159), along with the difference in the starting level (intercept) and slope of this rate in the period after each policy change was introduced, using ordinary least squares regression with Newey-West corrected standard errors (which account for autocorrelation). Estimates were adjusted for age and sex and all slopes are presented as incidence per year. P values indicate significance of the difference between the slope/intercept in the post policy period, compared to estimates from the pre-policy period.

As a post-hoc analysis, the impact of the introduction of the QOF in 2004[23] (month 59) was explored as an additional break-point in the interrupted time-series analysis. Sensitivity analyses explored the impact of excluding those deemed to be at high risk due to 1) high cardiovascular risk score documented in the primary care physician notes and 2) high cardiovascular risk score documented in the notes or estimated using the QRISK2 algorithm[24] and available risk factor data.

Data access and cleaning methods

SS had access to the entire CPRD database from which eligible patients were identified and all data extracted. Where multiple blood pressure values appeared in the record on any one day, the lowest value was selected. Treatments/diagnoses of conditions were determined from the presence of at least one coded prescription/diagnostic code respectively. Baseline data were extracted from within each patient's up to standard registration period and were drawn from the latest available record prior to study entry. Values deemed *a priori* to be clinically implausible were excluded (e.g. weight values of less than 20kg and more than 200kg). Where there was evidence of prior smoking but the most recent smoking status recorded indicated no smoking, this was corrected by classifying patients as former smokers.

Measures taken to reduce bias

To reduce the possibility of selection bias, all patients fulfilling the eligibility criteria for the study were included. Mild hypertension was defined according to measured blood pressure values rather than diagnostic codes, to reduce the potential for reporting bias, particularly in the pre-QOF era

For peer review only - http://bmjopen.bmj.com/site/about/guidelines.xhtml

when coding was less standardised. For the primary analysis, breakpoints for the interrupted time series analysis were pre-specified based on policy changes thought likely to influence the giving of lifestyle advice and treatment, rather than trends observed in the data.

Data linkage

Data linkage to the Office for National Statistics (ONS) mortality register was used to define patient mortality (patient exit from the study). Linkages to Basic Inpatient Hospital Episode Statistics (HES) were used in conjunction with CPRD records to define patient eligibility for the study (e.g. previous stroke), and define patient ethnicity (where unavailable in Primary Care records). A linkage to the Index of Multiple Deprivation was required to acquire practice level indices of multiple deprivation.

Patient involvement

Patients were not involved in the development or design of this study.

Approval

Ethics approval for observational research using the CPRD with approval from the Independent Scientific Advisory Committee (ISAC) was granted by a National Research Ethics Service committee (Trent Multi-Research Ethics Committee, REC reference number 05/MRE04/87). The present study protocol (16_008R) was approved by this committee in March 2016, prior to obtaining the data cut (protocol given in the online appendix). A project summary was published on the CPRD website (https://www.cprd.com/isac).

All data cleaning and analyses were conducted using STATA version 13.1 and 14.2 (MP parallel edition, StataCorp, Texas, USA). Results are presented as means, medians or proportions, with standard deviations, interquartile range or 95% confidence intervals, unless otherwise stated.

Results

Prevalence of lifestyle advice and treatment

Of the 7,416,968 individuals aged 18-74 years registered at linked, up-to-standard practices within the CPRD during the study period, a total of 108,843 patients (1.46%, 95% CI 1.45%, 1.47%) from 694 practices (333 ± 192 patients per practice) met the criteria for having uncomplicated mild hypertension during the study period (figure 1). Included patients had a mean age of 51.9 ± 12.9 years, 60.0% (95% CI 59.7%, 60.3%) were female and mean blood pressure (across 3 visits) was 144.3 ± 5.9 /87.3 ± 5.8 mmHg (table 3). Patients spent a median time of 2.6 years in the study (IQR 0.9, 5.5) and exited the cohort due to development of stage 2 hypertension (6,269 patients [5.8%]), a cardiovascular risk factors or score >20% (3,496 patients [5.0%]), advice being given (18,647 patients [17.1%]), treatment prescribed (35,305 patients [32.4%]), end of study follow-up (38,515 patients [35.4%]) or a combination of reasons (4,656 patients [4.3%]). During the study period, a total of 13,269 (12.2%, 95% CI 12.0%, 12.4%) were given lifestyle advice alone, 32,578 (29.9%, 95% CI 29.7%, 30.2%) were only prescribed an antihypertensive and 21,128 (19.4%, 95% CI 19.2%, 19.6%) were given lifestyle advice and prescribed treatment (table 3). The most common type of lifestyle advice given was related to smoking (20.6%, 95% CI 20.4%, 20.9%; eTable 3, online appendix).

For peer review only - http://bmjopen.bmj.com/site/about/guidelines.xhtml

Interrupted time series analysis

In the pre-NHS health check period from January 1999 and to April 2009, the proportion of patients receiving lifestyle advice increased from zero by 0.04% per year (95% CI 0.04%, 0.05%) (figure 2a). The introduction of the NHS health check and new QOF indicators in April 2009 was associated with a significant drop in lifestyle advice being given (p<0.001), but thereafter it increased by 0.19% per year (95% CI 0.15%, 0.23%). Following changes to QOF indicators in April 2013, the incidence of lifestyle advice decreased by -0.27% per year (95% CI -0.35%, -0.19%).

Treatment prescriptions were given to approximately 1.29% (95% CI 1.23%, 1.34%) of patients with uncomplicated mild hypertension in the first month of the study, but the incidence then decreased by -0.05% (95% CI -0.06%, -0.05%) per year in the pre-policy period until 2009 (figure 2b). Neither the introduction of the NHS health check, nor changes to QOF indicators significantly affected the incidence of treatment prescriptions which decreased by -0.03% per year and then -0.10% per year in the post NHS health check and QOF indicator change periods respectively (figure 2b).

Post-hoc analysis

The introduction of the QOF and clinical guidelines for the management of hypertension in 2004 was associated with a step-change increase in the incidence of lifestyle advice of 0.15% (95% CI 0.05%, 0.26%; p=0.006) (figure 3a). Subsequently, there was a small but significant decrease in the incidence of lifestyle advice (-0.05% per year, 95% CI -0.07%, -0.04%). The NHS health check and new QOF indicators in 2009 were no longer associated with a significant drop in lifestyle advice when the introduction of the QOF (in 2004) was included as a cut-point in the post-hoc analysis (p=0.07). No policy or guideline changes were associated with significant changes in the incidence of treatment prescriptions for uncomplicated mild hypertension during the study period in the post-hoc analysis (figure 3b). Exclusion of high risk patients (using either definition) in the sensitivity analysis had no impact on the incidence of lifestyle advice or treatment prescription.

Discussion

The present study examined a large database of electronic health records to establish the incidence of guideline recommended lifestyle advice and non-guideline recommended treatment for uncomplicated mild hypertension in Primary Care. Across the study period, approximately one third of patients received lifestyle advice whilst half were prescribed antihypertensive treatment. The introduction of pay-for-performance indicators in 2004,[23] and more recently a cardiovascular risk screening programme,[19] was associated with an increase in the proportion of patients documented as having been offered lifestyle advice, suggesting a more guideline based approach to the management of uncomplicated mild hypertension. However, there remains an appreciable proportion offered pharmacological treatment and this seems to be unaffected by changes to guidelines and policy.

Strengths and limitations

This study examined electronic health records from a large, nationally representative sample of the UK population.[21] Participating practices included those which adhere to data quality standards set out by the CPRD to ensure accuracy of data studied. Analyses were undertaken according to a pre-specified protocol (see online appendix) which was reviewed and approved by an independent scientific advisory committee, minimising the possibility of reporting bias and data-mining.

For peer review only - http://bmjopen.bmj.com/site/about/guidelines.xhtml

In an attempt to define mild hypertension consistently across the population, it is possible that some patients with a diagnostic code for hypertension but no associated blood pressure values may have been excluded. The focus of this study was on 'uncomplicated' low risk patients, but for pragmatic reasons, no attempt was made in the primary analysis to exclude those with a high risk of cardiovascular disease according to an established risk calculator[24 25] due to large amounts of missing data (e.g. for cholesterol, BMI). Patients did exit the cohort if they had a coded high CVD risk score during follow-up. Whilst it is possible that some high risk patients may have been included in the study cohort initially, sensitivity analysis excluding patients with known high risk scores and those with a high estimated QRISK2 score[24] did not affect the study results.

The incidence of lifestyle advice was defined according to clinical codes related to the giving of lifestyle advice which might be of relevance to the management of hypertension and cardiovascular disease risk (see online appendix eTable 1). It was not possible to identify patients given lifestyle advice where it was either not coded or where it was only recorded in the free text consultation notes. The trend of increasing lifestyle advice coinciding with the introduction of the QOF suggests that it may have been under recorded, particularly in the early observational period.

Previous literature

To our knowledge, this is the first study to determine the incidence of lifestyle advice and treatment in patients with uncomplicated mild hypertension. Previous studies[26-30] have focused on the prevalence of hypertension (defined as a blood pressure $\geq$140/90mmHg) and whether or not patients are treated and controlled, without making the important distinction between hypertensive patients where treatment is appropriate and those where it is not, at least according to national guidelines.[18] These surveys estimate the prevalence of hypertension across the world at between 20-45% with estimates of treatment and control ranging from 55%-81% and 30-59% respectively.[29] Our data suggest that in uncomplicated mild hypertension, treatment rates are slightly lower (49% across the study period), perhaps reflecting uncertainty around its efficacy in this population.

We found a trend towards increased recording of lifestyle advice over time, particularly following the introduction of the QOF in 2004,[23] the NHS health check[19] and changes in QOF indicators in 2009. Previous studies examining the impact of the NHS health check have found no effect on the estimated prevalence of hypertension,[31] but in those screened, referrals to specialist clinics and programmes for lifestyle advice increased six-fold.[32] The apparent association between incidence of lifestyle advice and changes in QOF indicators is in contrast to previous studies which have suggested that pay-for-performance in the UK has not affected the quality of care for hypertension.[33] Whilst previous data have indicated that equivalent quality care remains, even after an indicator has been removed,[34] the present data suggest the opposite, with the incidence of recording of lifestyle advice decreasing after the indicator encouraging this was removed in 2014.[35]

The incidence of treatment prescriptions slightly decreased over time during the study with the largest reduction observed in the last study period (post 2013), perhaps due to more accurate blood pressure measurement and targeting of treatment brought about by new NICE guidelines and the introduction of out-of-office blood pressure measurement for diagnosis in 2011.[18] The overall decrease in treatment might suggest a change in GP treatment practice over the past 15 years. However, it is also possible that GP treatment practice has remained constant over time, and the

For peer review only - http://bmjopen.bmj.com/site/about/guidelines.xhtml

number of hypertensive patients being diagnosed and perceived as low risk has increased, as evidenced by the increasing prevalence of hypertension reported annually in the QOF over the past 10 years (from 12.5% to 13.8%).

Implications for clinical practice

The peak in prevalence of lifestyle advice coinciding with the inclusion of QOF indicators encouraging advice for physical activity, smoking cessation and diet in 2013,[20] suggests that GPs were following pay-for-performance indicators closely. One explanation might be the content of QOF templates on GP computer systems, which are changed depending on the indicators included in the QOF and are likely to have encouraged better coding of lifestyle advice during a GP consultation. The choice of pay-for-performance indicators to include and exclude in the QOF, should therefore be considered carefully, ensuring that such indicators reflect evidence based practice and optimal patient care.

One could argue that the evidence for giving lifestyle advice in uncomplicated mild hypertension is as inconclusive as the evidence for pharmacological interventions, with many trials having short follow-up and exhibiting risk of bias[18 36] such as inadequate randomisation and allocation concealment,[36] lack of blinding[37] and incomplete outcome data.[38] In addition, there are no trials of the impact of lifestyle advice on 'hard outcomes' such as cardiovascular disease and mortality[18] and the quality of evidence of an association from observational studies is generally low.[39] However, lifestyle advice can potentially be given at lost cost within a patient consultation, and whilst overall reductions in blood pressure may be modest,[36-38] some patients may wish to change their lifestyle in an effort to delay or prevent embarking on a formal treatment regime.[36]

Conclusions

The present study shows that the introduction of pay-for-performance indicators and a cardiovascular risk screening programme have been associated with increased guideline recommended lifestyle advice being offered to patients with uncomplicated mild hypertension, although the overall prevalence remains relatively low. However, nearly half of patients go on to be prescribed treatment, despite a lack of evidence suggesting benefit. This may be explained by a lack of clear evidence to guide treatment decisions. Adequately powered studies, perhaps using routine clinical data at scale are required to accurately define the relationship between treatment and cardiovascular disease and death in patients with uncomplicated mild hypertension.

For peer review only

For peer review only - http://bmjopen.bmj.com/site/about/guidelines.xhtml

Acknowledgements

The authors would like to thank Dr Blanca Gallego Luxan for reviewing and commenting on the study protocol.

Funding

This work was funded by an Medical Research Council (MRC) Strategic Skills Post-doctoral Fellowship (MR/K022032/1) held by JS. JS now receives funding from the National Institute for Health Research (NIHR) Collaboration for Leadership in Applied Health Research and Care Oxford at Oxford Health NHS Foundation Trust and an NIHR Professorship which is held by and also funds RJMcM (NIHR-RP-R2-12-015). FDRH acknowledges support from the NIHR as Director of the NIHR SPCR, Director of the NIHR CLARHC Oxford, Theme Leader of the NIHR Oxford BRC, NIHR Oxford DEC and also from Harris Manchester College. The views and opinions expressed are those of the authors and do not necessarily reflect those of the MRC, NHS, NIHR, or the Department of Health.

Competing interests

RS is a member of the CPRD's Independent Scientific Advisory Committee (ISAC), but was not involved in the approval of this study. All other authors report no relevant conflicts of interest.

Author's contributions

JS conceived and designed the study, secured funding, undertook the main analysis and drafted the manuscript. SS was responsible for the data management, assisted with the analysis and revised the manuscript. RS assisted with the study design, assisted with the analysis and revised the manuscript. JM assisted with the study design and revised the manuscript. UM assisted with the study design and revised the manuscript. RH assisted with the study design and revised the manuscript. RJM conceived the study, assisted with the study design and revised the manuscript.

Data sharing

Data used in this analysis was provided by the Clinical Practice Research Datalink under a Gold Licence and cannot be shared with other parties.

For peer review only

For peer review only - http://bmjopen.bmj.com/site/about/guidelines.xhtml

References

1. Lewington S, Clarke R, Qizilbash N, et al. Age-specific relevance of usual blood pressure to vascular mortality: a meta-analysis of individual data for one million adults in 61 prospective studies. *Lancet* 2002;360(9349):1903-13. [published Online First: 2002/12/21]

2. Lozano R, Naghavi M, Foreman K, et al. Global and regional mortality from 235 causes of death for 20 age groups in 1990 and 2010: a systematic analysis for the Global Burden of Disease Study 2010. *Lancet* 2012;380(9859):2095-128. doi: 10.1016/s0140-6736(12)61728-0 [published Online First: 2012/12/19]

3. Whitworth JA. 2003 World Health Organization (WHO)/International Society of Hypertension (ISH) statement on management of hypertension. *Journal of hypertension* 2003;21(11):1983-92. doi: 10.1097/01.hjh.0000084751.37215.d2 [published Online First: 2003/11/05]

4. Sanchez RA, Ayala M, Baglivo H, et al. Latin American guidelines on hypertension. Latin American Expert Group. *Journal of hypertension* 2009;27(5):905-22. doi: 10.1097/HJH.0b013e32832aa6d2 [published Online First: 2009/04/08]

5. Ogihara T, Kikuchi K, Matsuoka H, et al. The Japanese Society of Hypertension Guidelines for the Management of Hypertension (JSH 2009). *Hypertension research : official journal of the Japanese Society of Hypertension* 2009;32(1):3-107. [published Online First: 2009/03/21]

6. Mancia G, Fagard R, Narkiewicz K, et al. 2013 ESH/ESC Guidelines for the management of arterial hypertension: the Task Force for the management of arterial hypertension of the European Society of Hypertension (ESH) and of the European Society of Cardiology (ESC). *Journal of hypertension* 2013;31(7):1281-357. doi: 10.1097/01.hjh.0000431740.32696.cc [published Online First: 2013/07/03]

7. James PA, Oparil S, Carter BL, et al. 2014 evidence-based guideline for the management of high blood pressure in adults: report from the panel members appointed to the Eighth Joint National Committee (JNC 8). *JAMA : the journal of the American Medical Association* 2014;311(5):507-20. doi: 10.1001/jama.2013.284427 [published Online First: 2013/12/20]

8. Hackam DG, Quinn RR, Ravani P, et al. The 2013 Canadian Hypertension Education Program recommendations for blood pressure measurement, diagnosis, assessment of risk, prevention, and treatment of hypertension. *The Canadian journal of cardiology* 2013;29(5):528-42. doi: 10.1016/j.cjca.2013.01.005 [published Online First: 2013/04/02]

9. Toth PJ, Horwitz RI. Conflicting clinical trials and the uncertainty of treating mild hypertension. *The American journal of medicine* 1983;75(3):482-8. [published Online First: 1983/09/01]

10. Tavares A. Pharmacotherapy for mild hypertension. *Sao Paulo medical journal = Revista paulista de medicina* 2012;130(6):417-8. [published Online First: 2013/01/23]

11. Ramsay LE, ul Haq I, Yeo WW, et al. Interpretation of prospective trials in hypertension: do treatment guidelines accurately reflect current evidence? *Journal of hypertension Supplement : official journal of the International Society of Hypertension* 1996;14(5):S187-94. [published Online First: 1996/12/01]

12. Heath I. Waste and harm in the treatment of mild hypertension. *JAMA internal medicine* 2013;173(11):956-7. doi: 10.1001/jamainternmed.2013.970 [published Online First: 2013/05/24]

For peer review only - http://bmjopen.bmj.com/site/about/guidelines.xhtml

13. Hart JT. Historical footnote on the treatment of mild hypertension. *BMJ (Clinical research ed)* 2012;345:e6297. doi: 10.1136/bmj.e6297 [published Online First: 2012/09/26]

14. Morales Salinas A, Coca A, Olsen MH, et al. Clinical Perspective on Antihypertensive Drug Treatment in Adults With Grade 1 Hypertension and Low-to-Moderate Cardiovascular Risk: An International Expert Consultation. *Current problems in cardiology* 2017;42(7):198-225. doi: 10.1016/j.cpcardiol.2017.03.001 [published Online First: 2017/05/30]

15. Diao D, Wright JM, Cundiff DK, et al. Pharmacotherapy for mild hypertension. *The Cochrane database of systematic reviews* 2012;8:Cd006742. doi: 10.1002/14651858.CD006742.pub2 [published Online First: 2012/08/17]

16. Sundstrom J, Arima H, Jackson R, et al. Effects of blood pressure reduction in mild hypertension: a systematic review and meta-analysis. *Annals of internal medicine* 2015;162(3):184-91. doi: 10.7326/m14-0773 [published Online First: 2014/12/23]

17. Wright JT, Jr., Williamson JD, Whelton PK, et al. A Randomized Trial of Intensive versus Standard Blood-Pressure Control. *The New England journal of medicine* 2015;373(22):2103-16. doi: 10.1056/NEJMoa1511939 [published Online First: 2015/11/10]

18. National Clinical Guideline Centre. Hypertension: clinical management of primary hypertension in adults; Clinical guideline 127. London: Royal College of Physicians (UK), 2011.

19. Public Health England. NHS Health Check: our approach to the evidence. https://www.gov.uk/government/uploads/system/uploads/attachment_data/file/224537/NHS_Health_Check_our_approach_to_the_evidence_v2.pdf: NHS England, 2013.

20. NHS Employers GPC, NHS England. General Medical Services (GMS) contract Quality and Outcomes Framework (QOF): guidence for GMS contract 2013/14. Leeds: NHS Employers, 2013.

21. Herrett E, Gallagher AM, Bhaskaran K, et al. Data Resource Profile: Clinical Practice Research Datalink (CPRD). *International Journal of Epidemiology* 2015;44(3):827-36. doi: 10.1093/ije/dyv098

22. Joint Health Surveys Unit. Health Survey for England 2011. http://digital.nhs.uk/catalogue/PUB09300: Health and Social Care Information Centre, 2012.

23. NHS Employers GPC, NHS England. General Medical Services (GMS) contract Quality and Outcomes Framework (QOF): guidence for GMS contract 2004/05. Leeds: NHS Employers, 2004.

24. Hippisley-Cox J, Coupland C, Vinogradova Y, et al. Predicting cardiovascular risk in England and Wales: prospective derivation and validation of QRISK2. *BMJ (Clinical research ed)* 2008;336(7659):1475-82. doi: 10.1136/bmj.39609.449676.25 [published Online First: 2008/06/25]

25. D'Agostino RB, Sr., Vasan RS, Pencina MJ, et al. General cardiovascular risk profile for use in primary care: the Framingham Heart Study. *Circulation* 2008;117(6):743-53. doi: 10.1161/circulationaha.107.699579 [published Online First: 2008/01/24]

26. Joffres M, Falaschetti E, Gillespie C, et al. Hypertension prevalence, awareness, treatment and control in national surveys from England, the USA and Canada, and correlation with stroke and ischaemic heart disease mortality: a cross-sectional study. *BMJ open* 2013;3(8):e003423. doi: 10.1136/bmjopen-2013-003423 [published Online First: 2013/09/03]

For peer review only - http://bmjopen.bmj.com/site/about/guidelines.xhtml

27. Neuhauser HK, Adler C, Rosario AS, et al. Hypertension prevalence, awareness, treatment and control in Germany 1998 and 2008-11. *Journal of human hypertension* 2015;29(4):247-53. doi: 10.1038/jhh.2014.82 [published Online First: 2014/10/03]

28. Labeit AM, Klotsche J, Pieper L, et al. Changes in the prevalence, treatment and control of hypertension in Germany? A clinical-epidemiological study of 50.000 primary care patients. *PloS one* 2012;7(12):e52229. doi: 10.1371/journal.pone.0052229 [published Online First: 2013/01/04]

29. Ikeda N, Sapienza D, Guerrero R, et al. Control of hypertension with medication: a comparative analysis of national surveys in 20 countries. *Bulletin of the World Health Organization* 2014;92(1):10-19c. doi: 10.2471/blt.13.121954

30. Wolf-Maier K, Cooper RS, Kramer H, et al. Hypertension treatment and control in five European countries, Canada, and the United States. *Hypertension (Dallas, Tex : 1979)* 2004;43(1):10-7. doi: 10.1161/01.hyp.0000103630.72812.10 [published Online First: 2003/11/26]

31. Caley M, Chohan P, Hooper J, et al. The impact of NHS Health Checks on the prevalence of disease in general practices: a controlled study. *The British journal of general practice : the journal of the Royal College of General Practitioners* 2014;64(625):e516-21. doi: 10.3399/bjgp14X681013 [published Online First: 2014/07/30]

32. Robson J, Dostal I, Sheikh A, et al. The NHS Health Check in England: an evaluation of the first 4 years. *BMJ open* 2016;6(1):e008840. doi: 10.1136/bmjopen-2015-008840 [published Online First: 2016/01/15]

33. Serumaga B, Ross-Degnan D, Avery AJ, et al. Effect of pay for performance on the management and outcomes of hypertension in the United Kingdom: interrupted time series study. *BMJ (Clinical research ed)* 2011;342:d108. doi: 10.1136/bmj.d108 [published Online First: 2011/01/27]

34. Kontopantelis E, Springate D, Reeves D, et al. Withdrawing performance indicators: retrospective analysis of general practice performance under UK Quality and Outcomes Framework. *BMJ (Clinical research ed)* 2014;348:g330. doi: 10.1136/bmj.g330 [published Online First: 2014/01/29]

35. NHS Employers GPC, NHS England. General Medical Services (GMS) contract Quality and Outcomes Framework (QOF): guidence for GMS contract 2014/15. Leeds: NHS Employers, 2014.

36. Dickinson HO, Mason JM, Nicolson DJ, et al. Lifestyle interventions to reduce raised blood pressure: a systematic review of randomized controlled trials. *Journal of hypertension* 2006;24(2):215-33. doi: 10.1097/01.hjh.0000199800.72563.26 [published Online First: 2006/03/02]

37. Cornelissen VA, Smart NA. Exercise Training for Blood Pressure: A Systematic Review and Meta-analysis. *Journal of the American Heart Association: Cardiovascular and Cerebrovascular Disease* 2013;2(1) doi: 10.1161/jaha.112.004473

38. Ndanuko RN, Tapsell LC, Charlton KE, et al. Dietary Patterns and Blood Pressure in Adults: A Systematic Review and Meta-Analysis of Randomized Controlled Trials. *Advances in nutrition (Bethesda, Md)* 2016;7(1):76-89. doi: 10.3945/an.115.009753 [published Online First: 2016/01/17]

39. Aburto NJ, Ziolkovska A, Hooper L, et al. Effect of lower sodium intake on health: systematic review and meta-analyses. *BMJ (Clinical research ed)* 2013;346:f1326. doi: 10.1136/bmj.f1326 [published Online First: 2013/04/06]

For peer review only - http://bmjopen.bmj.com/site/about/guidelines.xhtml

Tables

Table 1. Study entry and exit criteria applied to potential participant records used in the study.

Study entry criteria	Study exit (first to occur)
Date of the third consecutive blood pressure reading between 140/90-159/99mmHg (within 12 months of each other)	Last date at which the most recent linked data are available from the CPRD (study end date, September 2015)
Aged between 18-74 years	Date of third consecutive blood pressure reading $\geq$above 160/100mmHg (within 12 months of each other) (progression to stage 2 hypertension)
Linked general practice, Hospital Episodes Statistics (HES) and Office for National Statistics (ONS) mortality records	Date of first record of a cardiovascular risk factor (left ventricular hypertrophy, atrial fibrillation, diabetes or chronic kidney disease or family history of premature heart disease) or high cardiovascular risk score (>20%)
Registered to a CPRD practice classified as 'up-to-standard'	Date of death
Classified as a CPRD 'acceptable patient'	Date of the most recent data upload from the practice to which a given patient is registered
No read code for previous cardiovascular disease (stroke, myocardial infarction, angina, coronary heart disease, peripheral vascular disease, heart failure)	Date at which a given patient transfers out of a registered CPRD practice
No read code for cardiovascular risk factor (left ventricular hypertrophy, atrial fibrillation, diabetes or chronic kidney disease or family history of premature heart disease)	Date of first coded lifestyle advice given after study entry (lifestyle advice analysis only)
No record of any blood pressure lowering medication prescription in the 12 months prior to the third consecutive blood pressure reading between 140/90-159/99 mmHg	Date of first prescribed antihypertensive treatment given after study entry (treatment analysis only)

CPRD = Clinical Practice Research Datalink

For peer review only

For peer review only - http://bmjopen.bmj.com/site/about/guidelines.xhtml

Table 2. Guideline and policy changes related to the ongoing management of hypertension (columns highlighted in dark grey indicate pre-specified cut points, column in light grey indicates additional post-hoc cut point for interrupted time series analysis)

Guideline/policy/QOF indicator	1998/1999	1999/2000	2001/2002	2001/2002	2002/2003	2003/2004	2004/2005	2005/2006	2006/2007	2007/2008	2008/2009	2009/2010	2010/2011	2011/2012	2012/2013	2013/2014	2014/2015	2015/2016
**NICE Clinical Guideline 18 (2004):** Essential hypertension: managing adult patients in primary care							✓	✓										
**NICE Clinical Guideline 34 (2006):** Management of hypertension in adults in Primary Care									✓	✓	✓	✓	✓					
**NICE Clinical Guideline 127 (2011):** Hypertension in adults: diagnosis and management														✓	✓	✓	✓	✓
**Policy change:** NHS health check cardiovascular risk screening programme introduced												✓	✓	✓	✓	✓	✓	✓
**QOF Hypertension indicator:** The percentage of patients with hypertension in whom there is a record of the blood pressure in the previous 9 months							✓	✓	✓	✓	✓	✓	✓	✓	✓			
**QOF Hypertension indicator:** The percentage of patients with hypertension in whom the last blood pressure (measured in the previous 9 months) is 150/90 or less							✓	✓	✓	✓	✓	✓	✓	✓	✓	✓	✓	✓
**QOF Hypertension indicator:** The percentage of patients aged 79 or under with hypertension in whom the last blood pressure reading (measured in the preceding 9 months) is 140/90 mmHg or less																✓		
**QOF Hypertension indicator:** The percentage of patients with hypertension who have not attained the age of 75 in whom there is an assessment of physical activity, using GPPAQ, in the preceding 12 months																✓		
**QOF Hypertension indicator:** The percentage of patients with hypertension aged 16 or over and who have not attained the age of 75 who score 'less than active' on GPPAQ in the preceding 12 months, who also have a record of a brief intervention																✓		
**QOF Blood pressure indicator:** The percentage of patients aged 40 or over who have a record of							✓	✓	✓	✓	✓	✓	✓	✓	✓	✓	✓	✓

For peer review only

For peer review only - http://bmjopen.bmj.com/site/about/guidelines.xhtml

blood pressure in the preceding 5 years																		
**QOF Smoking indicator:** The percentage of patients with hypertension who smoke whose notes contain a record that smoking cessation advice or referral to a specialist service, where available, has been offered within the previous 15 months							✓	✓	✓	✓	✓	✓	✓	✓	✓	✓	✓	✓
**QOF Smoking indicator:** The percentage of patients with hypertension whose notes record smoking status in the previous 15 months. Except those who have never smoked where smoking status need only be recorded once since diagnosis							✓	✓	✓	✓	✓	✓	✓	✓	✓	✓	✓	✓
**QOF Smoking indicator:** The contractor supports patients who smoke in stopping smoking by a strategy which includes providing literature and offering appropriate therapy																✓	✓	✓
**QOF Primary prevention of cardiovascular disease indicator:** In those patients with a new diagnosis of hypertension: the percentage of patients who have had a face to face cardiovascular risk assessment at the outset of diagnosis using an agreed risk assessment tool.												✓	✓	✓	✓			
**QOF Primary prevention of cardiovascular disease indicator:** The percentage of people diagnosed with hypertension who are given lifestyle advice for increasing physical activity, smoking cessation, safe alcohol consumption and healthy diet.												✓	✓	✓	✓	✓		
**QOF Primary prevention of cardiovascular disease indicator:** In those patients with a new diagnosis of hypertension, who have a recorded CVD risk assessment score of ≥20% in the preceding 12 months: the percentage who are currently treated with statins																✓	✓	✓

QOF=Quality and outcomes Framework; NHS=National Health Service; GPPAQ=General practice physical activity questionnaire; CVD=Cardiovascular disease

For peer review only

Table 3. Patient characteristics at study entry, stratified by subsequent outcome

Characteristic	Total population		Went on to receive no advice or treatment		Went on to receive advice only		Went on to receive treatment only		Went on to receive advice and treatment	
	Mean/ number	SD/%	Mean/ number	SD/%	Mean/ number	SD/%	Mean/ number	SD/%	Mean/ number	SD/%
Total population	108,843	100%	41,868	38.5%	13,269	12.2%	32,578	29.9%	21,128	19.4%
Age (years)	51.9	12.9	50.0	13.8	48.6	13.3	54.7	11.8	53.2	11.4
Sex (% male)	43567	40.0%	15,809	37.8%	5,443	41.0%	13,054	40.1%	9,261	43.8%
Time in study (years, median [IQR])	2.6	0.9, 5.5	4.6	2.5, 7.9	1.7	0.4, 4.1	1.6	0.4, 3.8	1.2	0.2, 3.2
Ethnicity										
White ethnicity	59,643	54.8%	21,447	51.2%	7,554	56.9%	17,939	55.1%	12,703	60.1%
Black ethnicity	1,527	1.4%	501	1.2%	162	1.2%	458	1.4%	406	1.9%
South Asian ethnicity	1,356	1.2%	437	1.0%	133	1.0%	487	1.5%	299	1.4%
Mixed race ethnicity	9,700	8.9%	3,691	8.8%	1,483	11.2%	2,566	7.9%	1,960	9.3%
Other ethnicity	1,041	1.0%	398	1.0%	111	0.8%	321	1.0%	211	1.0%
Unknown ethnicity	35,576	32.7%	15,394	36.8%	3,826	28.8%	10,807	33.2%	5,549	26.3%
BMI (kg/m^2)†	29.3	5.8	29.2	5.9	29.4	6.2	29.2	5.6	29.3	5.7
Current smoking status	23,592	21.7%	9,555	22.8%	2,286	17.2%	7,498	23.0%	4,253	20.1%
Alcohol (units per week)‡	12.1	14.6	11.2	12.5	15.9	19.7	10.6	13.0	13.5	16.0
IMD score of 5 (most deprived)	12,606	11.6%	4,217	10.1%	2,081	15.7%	3,116	9.6%	3,192	15.1%
Systolic BP (mmHg)	144.3	5.9	143.7	5.9	143.3	6.1	145.2	5.6	145.0	5.7
Diastolic BP (mmHg)	87.3	5.8	86.9	5.9	87.1	5.9	87.5	5.7	87.7	5.7
Mean CVD risk score (mean±SD)*	7.1%	6.3%	6.1%	5.9%	7.1%	6.7%	7.6%	6.2%	8.3%	6.9%
Age defined moderate CVD risk (%)ǂ	41,510	38.1%	14151	33.8%	3,887	29.3%	14,950	45.9%	8,522	40.3%
Statin prescription	13,426	12.3%	3,927	9.4%	1,472	11.1%	4,608	14.1%	3,419	16.2%
Antiplatelet prescription	6,029	5.5%	1,404	3.4%	480	3.6%	2,567	7.9%	1,578	7.5%

†Missing BMI data (n=46,599); ‡Missing alcohol data (n=72,785)

*Based on previous risk score or QRISK2 score estimated from existing or imputed data

ǂProportion of men aged ≥55 years and women aged ≥60 years who according to Morales Salinas et al., (2017)[14] should be classified as having moderate cardiovascular risk.

For peer review only - http://bmjopen.bmj.com/site/about/guidelines.xhtml

Figure legends

Figure 1. Flow diagram showing exclusion of patient records and definition of the final cohort

Figure 2. Primary analysis: Incidence of lifestyle advice and treatment by month.

(a) Lifestyle advice (b) Treatment
Incidence estimates adjusted for age and sex; QOF=Quality and Outcomes Framework; NHS=National Health Service

Figure 3. Post-hoc analysis: Incidence of lifestyle advice and treatment by month.

(a) Lifestyle advice (b) Treatment
Incidence estimates adjusted for age and sex; QOF=Quality and Outcomes Framework; NHS=National Health Service

For peer review only

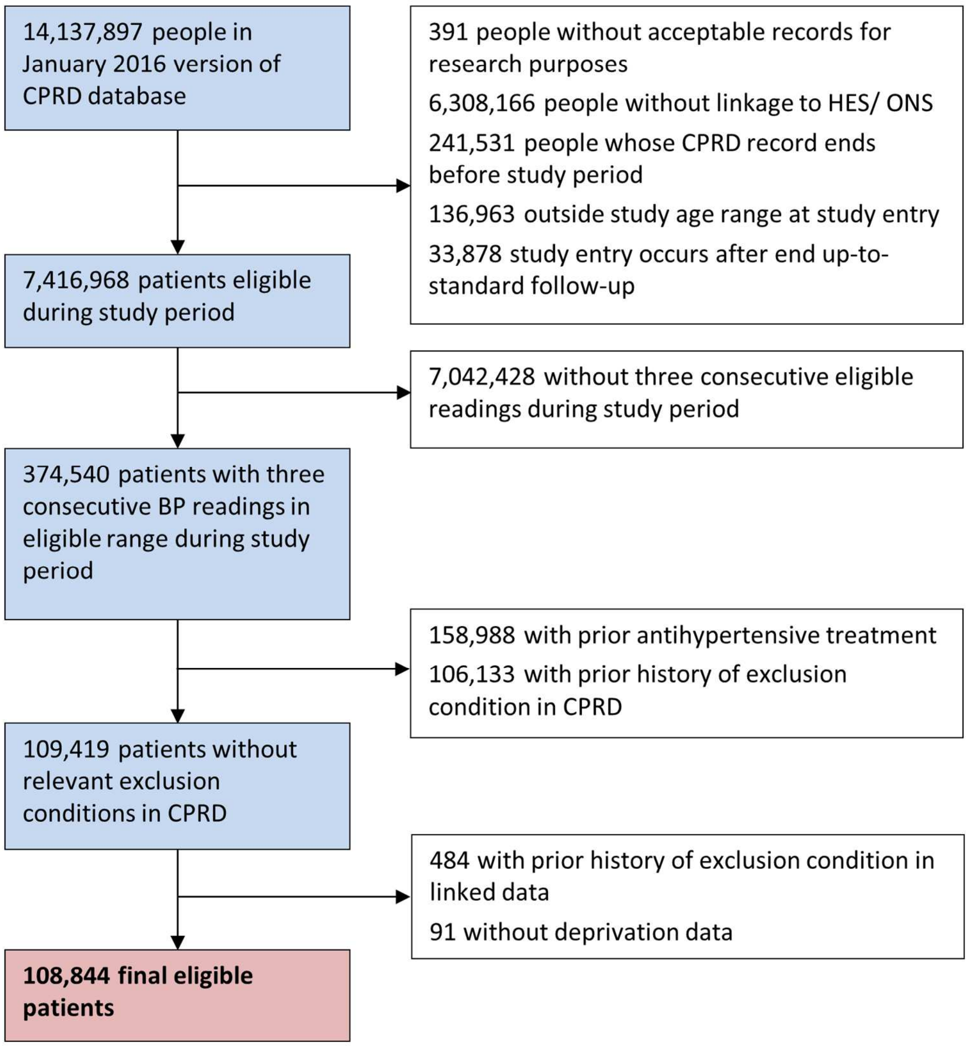

Figure 1

144x160mm (300 x 300 DPI)

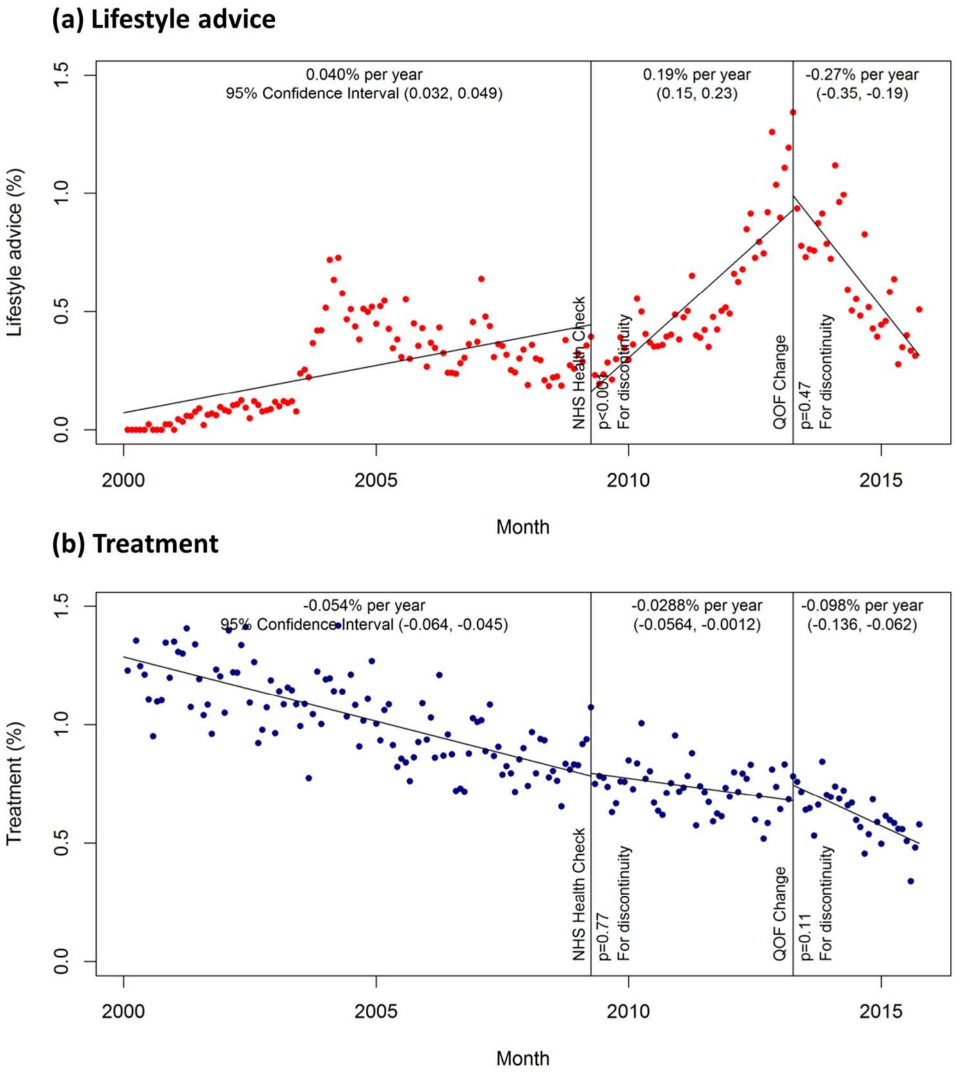

Figure 2

144x160mm (300 x 300 DPI)

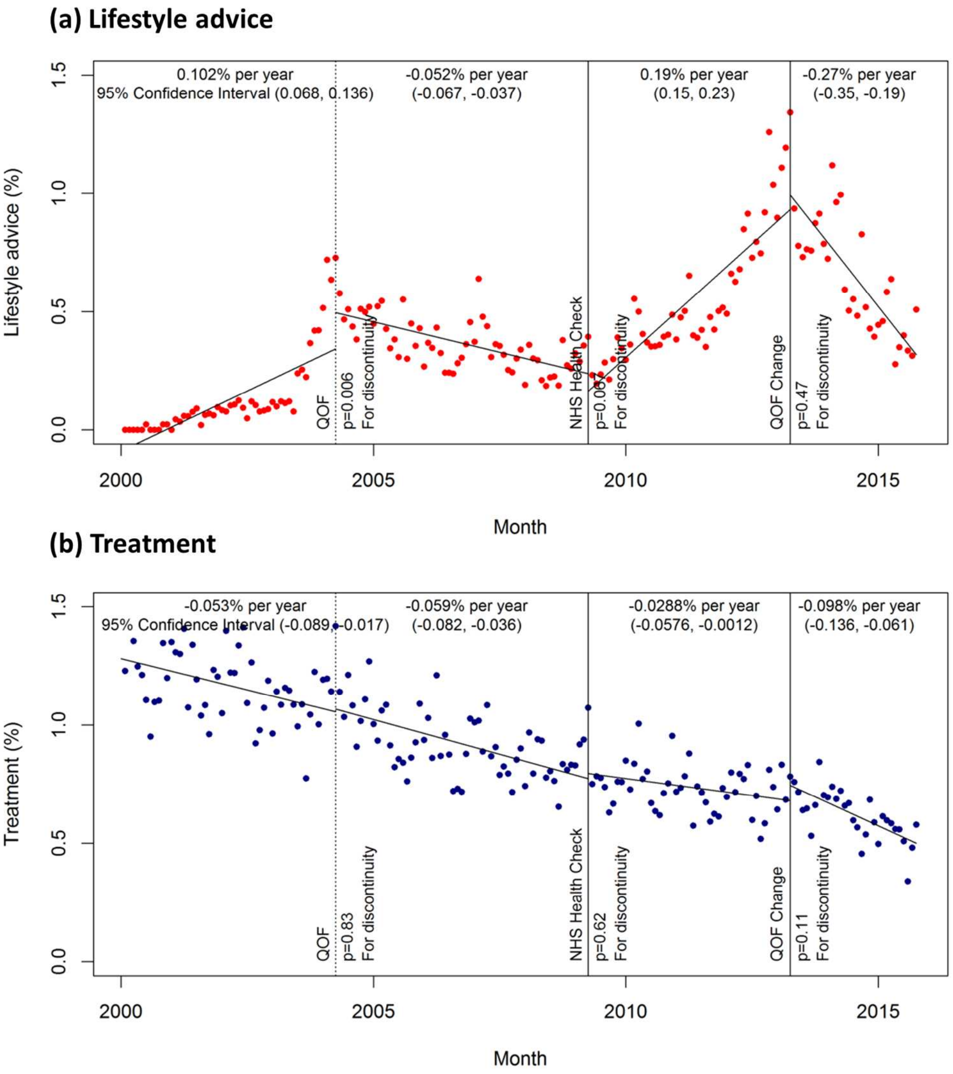

Figure 3

144x160mm (300 x 300 DPI)

Association of guideline and policy changes with incidence of lifestyle advice and treatment for uncomplicated mild hypertension in Primary Care: a longitudinal cohort study in the Clinical Practice Research Datalink

Online appendix

James P Sheppard,[1] Sarah Stevens,[1] Richard Stevens,[1] Jonathan Mant,[2] Una Martin,[3] FD Richard Hobbs[1] and Richard J McManus[1]

[1]University of Oxford, Oxford, UK
[2]University of Cambridge, Cambridge, UK
[3]University of Birmingham, Birmingham, UK

Corresponding author: James P Sheppard
Email: james.sheppard@phc.ox.ac.uk
Telephone: +44 1865 617192
Address: Nuffield Department of Primary Care Health Sciences, Radcliffe Observatory Quarter, University of Oxford, Oxford, OX2 6GG, UK

For peer review only

Contents

1. **eTable 1.** Read codes used to define lifestyle advice outcome in the CPRD
2. **eTable 2.** Read codes used to define antihypertensive treatment outcome in the CPRD
3. **eTable 3.** Frequency of different types of lifestyle advice given during follow-up
4. ISAC approved protocol for analyses undertaken
5. References

For peer review only

eTable 1. Read codes used to define lifestyle advice outcome in the CPRD

Med code	Read code	Description
18711	67H0.00	Lifestyle advice regarding alcohol
18926	67H1.00	Lifestyle advice regarding smoking
98146	67H7.00	Lifestyle advice regarding diet
98230	67H8.00	Lifestyle advice regarding hypertension
10979	67H2.00	Lifestyle advice regarding exercise
58900	ZC21K00	Advice to change fruit and nut intake
12483	8CAQ.00	Advice about blood glucose control
38021	67IG.00	Oral health advice given
69176	ZC2..11	Advice on diet
69175	ZC2..12	Advice about diet
19414	ZGB..00	Advice relating to treatment and prevention
45933	ZC28.00	Advice to change potassium intake
56711	ZC24.00	Advice to change carbohydrate intake
72645	ZC21700	Advice to change cheese intake
22142	ZG53.00	Advice about weight
54209	ZC2H.00	Advice to change alcohol intake
25041	ZC2CA00	Dietary advice for type II diabetes
102574	67Ip.00	Advice about food intake
19446	ZC26.00	Advice to change fat intake
31922	ZC4..00	Dietary health promotion advice
43187	ZC26200	Advice to change low fat food intake
62415	ZC21c00	Advice change salty food intake
54111	ZC2F.00	Advice to change high energy food intake
42765	ZC2CI00	Dietary advice for lipid disorder
8304	ZC2C711	Dietary advice for weight reduction
9045	ZG23300	Advice on smoking
68419	ZC2E.00	Advice to change convenience food intake
32922	ZC21800	Advice to change egg intake
107619	8CA4M11	High energy diet advice
61863	ZC25.00	Advice to change protein intake
47085	ZC27.00	Advice to change sodium intake
69039	ZC21b00	Advice to change sugar intake
11882	67I9.00	Advice about weight
18759	ZC21.00	Advice to change food intake
24398	ZC23.00	Advice to change dietary fibre intake
59027	ZC26100	Advice to change fatty food intake
72643	ZC21U00	Advice to change table salt intake
7854	ZC2C.00	Dietary advice for disorder
100874	ZC2K.00	Advice to change nutritional product intake
72642	ZC21a00	Advice to change sugary food intake
72640	ZC21900	Advice to change butter intake
50368	ZC2I.00	Advice to change calorie intake
72648	ZC21X00	Advice to change bread intake
7622	8CAL.00	Smoking cessation advice
95114	ZC24300	Advice to change carbohydrate food intake
10642	ZC2C800	Dietary advice for diabetes mellitus
22799	ZC21200	Advice to change dairy food intake
69041	ZC21a11	Advice to change sweet food intake
100099	8IAj.00	Smoking cessation advice declined
38057	ZG22.00	Advice to stop behaviour

For peer review only

72641	ZC21P00	Advice to change vegetable intake
63431	ZC22300	Advice to change coffee intake
66715	ZC2K100	Advice to increase nutritional product intake
11140	ZG23100	Advice on alcohol consumption
57646	ZG11.00	Advice about activity of daily living
54873	ZG1..00	Advice about activity
107333	8CA4Q11	Healthy eating advice
85837	ZG21.00	Advice to avoid behaviour
30246	ZG23.00	Advice relating to health-related behaviour
97163	ZC22200	Advice to change alcoholic drink intake
56677	ZC23100	Advice to change high fibre food intake
31067	ZC2..00	Dietary advice
19735	ZG16100	Advice to exercise
95737	ZC21G00	Advice to change meat intake
101645	ZC23200	Advice to change low fibre food intake
18748	ZG16.00	Advice about exercise
43375	ZC2CO00	Dietary advice for weight loss
101043	8Cd7.00	Advice given about weight management
41042	8CAg.00	Smoking cessation advice provided by community pharmacist
33694	ZC2CJ00	Dietary advice for hyperlipidaemia
95561	ZA11925	Advice on diet
69040	ZC24400	Advice to change refined carbohydrate food intake
68368	ZC21H00	Advice to reduce meat intake
51948	ZG12.00	Advice to undertake activity

For peer review only

eTable 2. Read codes used to define antihypertensive treatment outcome in the CPRD

ACE inhibitors

Product code	GEM code	Description
217	3900007	CAPTOPRIL 4 MG/ML LIQ
59699	21647021	Captopril 5mg/5ml oral solution sugar free
8923	2913007	CAPTOPRIL 100 MG TAB
25051	!1092601	CAPOZIDE
3509	2652007	ENALAPRIL MALEATE 40 MG TAB
27890	!2215902	ENALAPRIL MALEATE
22004	!1100404	CARACE (SPECIAL COMPLIANCE PACK)
24693	!1100403	CARACE (SPECIAL COMPLIANCE PACK)
23382	!1100402	CARACE (SPECIAL COMPLIANCE PACK)
54986	35397020	Perindopril erbumine 8mg/5ml oral suspension
2927	3203007	PERINDOPRIL/TERT-BUTYLAMINE 2 MG TAB
60744	22980021	Perindopril erbumine 8mg / Amlodipine 5mg tablets
56079	47933020	Perindopril tosilate 10mg tablets
57944	47936020	Perindopril tosilate 2.5mg tablets
56157	47939020	Perindopril tosilate 5mg / Indapamide 1.25mg tablets
61117	20667021	Perindopril erbumine 4mg/5ml oral solution
22882	!8505496	RAMIPRIL
18269	52608020	Acepril 12.5mg tablets (Bristol-Myers Squibb Pharmaceuticals Ltd)
3069	52609020	Acepril 25mg tablets (Bristol-Myers Squibb Pharmaceuticals Ltd)
18325	52610020	Acepril 50mg tablets (Bristol-Myers Squibb Pharmaceuticals Ltd)
3310	48565020	Capoten 12.5mg tablets (Bristol-Myers Squibb Pharmaceuticals Ltd)
56509	1375020	Capoten 12.5mg tablets (Dowelhurst Ltd)
1144	48566020	Capoten 25mg tablets (Bristol-Myers Squibb Pharmaceuticals Ltd)
3839	48567020	Capoten 50mg tablets (Bristol-Myers Squibb Pharmaceuticals Ltd)
25998	85701020	Captomex 12.5mg tablets (Actavis UK Ltd)
28820	85702020	Captomex 25mg tablets (Actavis UK Ltd)
24482	85703020	Captomex 50mg tablets (Actavis UK Ltd)
33646	56633020	Captopril 12.5mg Tablet (Generics (UK) Ltd)
34544	56638020	Captopril 12.5mg Tablet (IVAX Pharmaceuticals UK Ltd)
1121	60377020	Captopril 12.5mg tablets
46951	56643020	Captopril 12.5mg tablets (A A H Pharmaceuticals Ltd)
41633	56801020	Captopril 12.5mg tablets (Actavis UK Ltd)
46957	59293020	Captopril 12.5mg tablets (Tillomed Laboratories Ltd)
58195	19216020	Captopril 12.5mg/5ml oral solution
35302	91890020	Captopril 12.5mg/5ml oral suspension
43507	56634020	Captopril 25mg Tablet (Generics (UK) Ltd)
34562	56639020	Captopril 25mg Tablet (IVAX Pharmaceuticals UK Ltd)
34936	56686020	Captopril 25mg Tablet (Lagap)
1143	60378020	Captopril 25mg tablets
43649	56644020	Captopril 25mg tablets (A A H Pharmaceuticals Ltd)
41617	56802020	Captopril 25mg tablets (Actavis UK Ltd)
37655	59733020	Captopril 25mg tablets (Teva UK Ltd)
11641	68130020	Captopril 25mg with Hydrochlorothiazide 12.5mg tablets
52499	19239020	Captopril 25mg/5ml oral solution
59915	21645021	Captopril 25mg/5ml oral solution sugar free
54544	19241020	Captopril 25mg/5ml oral suspension
39512	96425020	Captopril 25mg/5ml oral suspension
52293	20854020	Captopril 2mg capsules

For peer review only

15958	82305020	Captopril 2mg tablets
36742	93652020	Captopril 2mg/5ml oral suspension
17633	87231020	Captopril 3mg/5ml oral solution
34719	56635020	Captopril 50mg Tablet (Generics (UK) Ltd)
34937	56640020	Captopril 50mg Tablet (IVAX Pharmaceuticals UK Ltd)
1807	60379020	Captopril 50mg tablets
41743	59737020	Captopril 50mg tablets (Teva UK Ltd)
10902	68129020	Captopril 50mg with Hydrochlorothiazide 25mg tablets
46851	417021	Captopril 5mg/5ml oral solution
17624	88718020	Captopril 5mg/5ml oral suspension
33336	69419020	Captopril 5mg/5ml Oral suspension (Eldon Laboratories)
44527	98847020	Captopril 5mg/ml oral solution sugar free
43432	92446020	Captopril 6.25mg tablets
28486	89909020	Captopril 6.25mg/5ml oral suspension
45228	91312020	Captopril capsules
56850	84385020	Ecopace 12.5mg tablets (AMCo)
32514	84386020	Ecopace 25mg tablets (AMCo)
15135	69269020	Hydrochlorothiazide with captopril 12.5mg with 25mg Tablet
11133	69268020	Hydrochlorothiazide with captopril 25mg with 50mg Tablet
21943	84490020	Kaplon 12.5mg tablets (Teva UK Ltd)
26995	84491020	Kaplon 25mg tablets (Teva UK Ltd)
32048	84492020	Kaplon 50mg tablets (Teva UK Ltd)
60349	21646021	Noyada 25mg/5ml oral solution (Martindale Pharmaceuticals Ltd)
20849	84462020	Tensopril 12.5mg tablets (Teva UK Ltd)
30039	84463020	Tensopril 25mg tablets (Teva UK Ltd)
23478	84464020	Tensopril 50mg tablets (Teva UK Ltd)
15605	49385020	Cilazapril 250micrograms tablets
23642	50587020	Vascace 0.25mg Tablet (Roche Products Ltd)
12574	49387020	Cilazapril 1mg tablets
12412	54164020	Cilazapril 2.5mg tablets
12411	49386020	Cilazapril 500microgram tablets
13026	54165020	Cilazapril 5mg tablets
16212	50589020	Vascace 1mg tablets (Roche Products Ltd)
16197	55290020	Vascace 2.5mg tablets (Roche Products Ltd)
21053	50588020	Vascace 500microgram tablets (Roche Products Ltd)
16196	55291020	Vascace 5mg tablets (Roche Products Ltd)
42908	61247020	Enalapril 5mg tablets (IVAX Pharmaceuticals UK Ltd)
34768	61253020	Enalapril 20mg tablets (IVAX Pharmaceuticals UK Ltd)
20188	60798020	Enalapril 2.5mg wafer
34453	61301020	Enalapril 20mg tablets (Generics (UK) Ltd)
16708	60797020	Enalapril titration pack
8106	55119020	Innovace 2.5mg tablets (Merck Sharp & Dohme Ltd)
41417	60324020	Enalapril 2.5mg tablets (A A H Pharmaceuticals Ltd)
43563	61391020	Enalapril 2.5mg tablets (Zentiva)
23252	73042020	Pralenal 10 tablets (Opus Pharmaceuticals Ltd)
27871	86081020	Innovace melt 10mg Wafer (Merck Sharp & Dohme Ltd)
8105	58451020	Innovace 20mg tablets (Merck Sharp & Dohme Ltd)
34400	55722020	Enalapril 5mg Tablet (Dowelhurst Ltd)
22439	83449020	Ednyt 20mg Tablet (Dominion Pharma)
34798	61362020	Enalapril 20mg tablets (Sandoz Ltd)
52010	1418020	Enalapril 10mg tablets (Alliance Healthcare (Distribution) Ltd)
53915	61780020	Enalapril 5mg tablets (Dexcel-Pharma Ltd)
34953	61397020	Enalapril 20mg tablets (Zentiva)
31587	86077020	Innovace melt 20mg Wafer (Merck Sharp & Dohme Ltd)

For peer review only

32241	60326020	Enalapril 10mg tablets (A A H Pharmaceuticals Ltd)
46974	61297020	Enalapril 5mg tablets (Generics (UK) Ltd)
60143	38499020	Enalapril 5mg tablets (Medreich Plc)
41694	61246020	Enalapril 2.5mg tablets (IVAX Pharmaceuticals UK Ltd)
22708	77941020	Enalapril 5mg wafer
37087	94156020	Enalapril 5mg/5ml oral suspension
196	60791020	Enalapril 5mg tablets
34712	63374020	Enalapril 20mg tablets (Kent Pharmaceuticals Ltd)
35794	60325020	Enalapril 5mg tablets (A A H Pharmaceuticals Ltd)
50863	19819020	Enalapril 5mg/5ml oral solution (Drug Tariff Special Order)
11197	86080020	Innovace melt 5mg Wafer (Merck Sharp & Dohme Ltd)
29530	86079020	Innovace melt 2.5mg Wafer (Merck Sharp & Dohme Ltd)
43411	61356020	Enalapril 5mg tablets (Sandoz Ltd)
42894	61262020	Enalapril 10mg tablets (Teva UK Ltd)
24041	77943020	Enalapril 20mg wafer
59996	1435020	Enalapril 20mg tablets (Milpharm Ltd)
1904	60796020	Enalapril 20mg tablets
15085	58452020	Innovace Titration pack (Merck Sharp & Dohme Ltd)
8830	55121020	Innovace 10mg tablets (Merck Sharp & Dohme Ltd)
33057	75030020	Ednyt 5mg Tablet (Dominion Pharma)
448	60790020	Enalapril 2.5mg tablets
8800	55120020	Innovace 5mg tablets (Merck Sharp & Dohme Ltd)
61133	1425020	Enalapril 10mg tablets (Phoenix Healthcare Distribution Ltd)
45217	63368020	Enalapril 5mg tablets (Kent Pharmaceuticals Ltd)
28127	61260020	Enalapril 2.5mg tablets (Teva UK Ltd)
31716	61347020	Enalapril 20mg tablets (Actavis UK Ltd)
37080	94154020	Enalapril 5mg/5ml oral solution
42902	61266020	Enalapril 20mg tablets (Teva UK Ltd)
52882	40203020	Enalapril 5mg/5ml oral suspension sugar free
44657	75029020	Ednyt 2.5mg Tablet (Dominion Pharma)
57378	30788020	Enalapril 2mg/5ml oral suspension
1299	60792020	Enalapril 10mg tablets
19208	61344020	Enalapril 10mg tablets (Actavis UK Ltd)
57882	19854020	Enalapril 2.5mg/5ml oral suspension
34952	61298020	Enalapril 10mg tablets (Generics (UK) Ltd)
36753	75031020	Ednyt 10mg Tablet (Dominion Pharma)
53719	1427020	Enalapril 20mg tablets (Alliance Healthcare (Distribution) Ltd)
33078	60335020	Enalapril 20mg tablets (A A H Pharmaceuticals Ltd)
50780	30786020	Enalapril 2mg/5ml oral solution
42723	73041020	Pralenal 5 tablets (Opus Pharmaceuticals Ltd)
58751	19799020	Enalapril 1.25mg/5ml oral suspension
42901	61261020	Enalapril 5mg tablets (Teva UK Ltd)
50334	19817020	Enalapril 4mg/5ml oral suspension
55903	61781020	Enalapril 10mg tablets (Dexcel-Pharma Ltd)
41746	61357020	Enalapril 10mg tablets (Sandoz Ltd)
13755	77942020	Enalapril 10mg wafer
5189	51068020	Enalapril 20mg / Hydrochlorothiazide 12.5mg tablets
1021	51047020	Innozide 20mg/12.5mg tablets (Merck Sharp & Dohme Ltd)
28438	85815020	Triapin 2.5mg/2.5mg modified-release tablets (Sanofi)
17474	78452020	Felodipine 5mg modified-release / Ramipril 5mg tablets
17006	85824020	Triapin 5mg/5mg modified-release tablets (Sanofi)
21162	78451020	Felodipine 2.5mg modified-release / Ramipril 2.5mg tablets
13589	74175020	Staril 20mg tablets (Bristol-Myers Squibb Pharmaceuticals Ltd)
633	74178020	Fosinopril 10mg tablets

For peer review only

4571	74174020	Staril 10mg tablets (Bristol-Myers Squibb Pharmaceuticals Ltd)
5861	74179020	Fosinopril 20mg tablets
18263	59646020	Acezide 25mg/50mg tablets (Bristol-Myers Squibb Pharmaceuticals Ltd)
1520	68133020	Capozide 25mg/50mg tablets (Bristol-Myers Squibb Pharmaceuticals Ltd)
39227	95527020	Capozide LS 12.5mg/25mg tablets (Bristol-Myers Squibb Pharmaceuticals Ltd)
3203	68134020	Capozide LS Tablet (E R Squibb and Sons Ltd)
32166	80831020	Capto-co 25mg+50mg Tablet (IVAX Pharmaceuticals UK Ltd)
11561	84483020	Co-zidocapt 12.5mg/25mg tablets
11351	84482020	Co-zidocapt 25mg/50mg tablets
15031	74293020	Accuretic 12.5mg/10mg tablets (Pfizer Ltd)
15108	74296020	Quinapril 10mg / Hydrochlorothiazide 12.5mg tablets
6408	81266020	Tanatril 5mg tablets (Chiesi Ltd)
12815	81267020	Tanatril 10mg tablets (Chiesi Ltd)
16924	81181020	Imidapril 5mg tablets
18219	81183020	Imidapril 20mg tablets
32560	81268020	Tanatril 20mg tablets (Chiesi Ltd)
12858	81182020	Imidapril 10mg tablets
37978	94277020	Perindopril arginine 5mg / Indapamide 1.25mg tablets
6794	83957020	Perindopril erbumine 4mg / Indapamide 1.25mg tablets
14228	84712020	Coversyl Plus tablets (Servier Laboratories Ltd)
33977	65098020	Lisinopril 10mg tablets (Generics (UK) Ltd)
45337	65231020	Lisinopril 5mg tablets (Actavis UK Ltd)
56505	1458020	Zestril 5mg tablets (Lexon (UK) Ltd)
58294	1461020	Lisinopril 5mg tablets (Accord Healthcare Ltd)
46975	65137020	Lisinopril 5mg tablets (Sandoz Ltd)
30921	64809020	Lisinopril 2.5mg tablets (Teva UK Ltd)
56510	1481020	Zestril 20mg tablets (Sigma Pharmaceuticals Plc)
43413	65206020	Lisinopril 20mg tablets (A A H Pharmaceuticals Ltd)
8268	68567020	Zestril 20mg tablets (AstraZeneca UK Ltd)
3720	68562020	Zestril 2.5mg tablets (AstraZeneca UK Ltd)
41573	65180020	Lisopress 10mg tablets (Teva UK Ltd)
43416	65202020	Lisinopril 10mg tablets (A A H Pharmaceuticals Ltd)
12313	68559020	Carace 20mg tablets (Bristol-Myers Squibb Pharmaceuticals Ltd)
59111	1473020	Lisinopril 20mg tablets (Alliance Healthcare (Distribution) Ltd)
55896	65227020	Lisinopril 2.5mg tablets (Actavis UK Ltd)
45816	71542020	Lisinopril 5mg tablets (Almus Pharmaceuticals Ltd)
41522	65183020	Lisopress 20mg tablets (Teva UK Ltd)
43566	65134020	Lisinopril 2.5mg tablets (Sandoz Ltd)
43418	65199020	Lisinopril 5mg tablets (A A H Pharmaceuticals Ltd)
14387	68555020	Carace 5mg tablets (Bristol-Myers Squibb Pharmaceuticals Ltd)
19198	64822020	Lisinopril 20mg tablets (Teva UK Ltd)
20975	90115020	Lisinopril 7.5mg/5ml oral suspension
65	68572020	Lisinopril 10mg tablets
58461	1437020	Lisinopril 2.5mg tablets (Kent Pharmaceuticals Ltd)
11987	88326020	Lisinopril 5mg/5ml oral solution
54288	69238020	Lisinopril 10mg tablets (Arrow Generics Ltd)
6807	68563020	Zestril 5mg tablets (AstraZeneca UK Ltd)
60010	1462020	Lisinopril 10mg tablets (Kent Pharmaceuticals Ltd)
45300	65236020	Lisinopril 10mg tablets (Actavis UK Ltd)
58258	20373020	Lisinopril 2.5mg/5ml oral suspension
6806	68564020	Zestril 10mg tablets (AstraZeneca UK Ltd)

For peer review only

55639	1472020	Lisinopril 10mg tablets (Accord Healthcare Ltd)
51433	66292020	Lisinopril 20mg tablets (Tillomed Laboratories Ltd)
47159	71545020	Lisinopril 10mg tablets (Almus Pharmaceuticals Ltd)
56279	20371020	Lisinopril 2.5mg/5ml oral solution
34799	65220020	Lisinopril 20mg tablets (Zentiva)
59109	66285020	Lisinopril 5mg tablets (Tillomed Laboratories Ltd)
41538	65173020	Lisopress 2.5mg tablets (Teva UK Ltd)
57048	65217020	Lisinopril 10mg tablets (Zentiva)
41532	65176020	Lisopress 5mg tablets (Teva UK Ltd)
58863	1471020	Lisinopril 10mg tablets (Phoenix Healthcare Distribution Ltd)
58871	14472021	Lisinopril 10mg tablets (Waymade Healthcare Plc)
78	68571020	Lisinopril 5mg tablets
54037	1467020	Lisinopril 10mg tablets (Relonchem Ltd)
16701	68556020	Carace 10mg tablets (Bristol-Myers Squibb Pharmaceuticals Ltd)
60232	65214020	Lisinopril 5mg tablets (Zentiva)
54512	91738020	Lisinopril Oral solution
45324	65239020	Lisinopril 20mg tablets (Actavis UK Ltd)
53551	1484020	Lisinopril 20mg tablets (Phoenix Healthcare Distribution Ltd)
34471	65081020	Lisinopril 5mg tablets (Generics (UK) Ltd)
34696	65102020	Lisinopril 20mg tablets (Generics (UK) Ltd)
10882	68554020	Carace 2.5mg tablets (Bristol-Myers Squibb Pharmaceuticals Ltd)
55456	1448020	Lisinopril 5mg tablets (Alliance Healthcare (Distribution) Ltd)
55002	1486020	Lisinopril 20mg tablets (Accord Healthcare Ltd)
37778	94657020	Lisinopril 5mg/5ml oral suspension
61262	1483020	Lisinopril 20mg tablets (Bristol Laboratories Ltd)
60309	1454020	Lisinopril 5mg tablets (Relonchem Ltd)
54928	1470020	Lisinopril 10mg tablets (Bristol Laboratories Ltd)
19223	64818020	Lisinopril 10mg tablets (Teva UK Ltd)
277	68570020	Lisinopril 2.5mg tablets
58682	66939020	Lisinopril 2.5mg tablets (Generics (UK) Ltd)
58451	71538020	Lisinopril 2.5mg tablets (Almus Pharmaceuticals Ltd)
53820	69229020	Lisinopril 5mg tablets (Arrow Generics Ltd)
69	68575020	Lisinopril 20mg tablets
46979	65143020	Lisinopril 20mg tablets (Sandoz Ltd)
32597	65140020	Lisinopril 10mg tablets (Sandoz Ltd)
43412	65196020	Lisinopril 2.5mg tablets (A A H Pharmaceuticals Ltd)
55588	1482020	Lisinopril 20mg tablets (Sigma Pharmaceuticals Plc)
57588	1446020	Zestril 2.5mg tablets (Mawdsley-Brooks & Company Ltd)
19204	64814020	Lisinopril 5mg tablets (Teva UK Ltd)
54283	20380020	Lisinopril 5mg/5ml oral suspension (Drug Tariff Special Order)
53271	1463020	Lisinopril 10mg tablets (Alliance Healthcare (Distribution) Ltd)
52088	1459020	Lisinopril 5mg tablets (Phoenix Healthcare Distribution Ltd)
60097	65209020	Lisinopril 2.5mg tablets (Zentiva)
38995	95877020	Zestoretic 20 tablets (AstraZeneca UK Ltd)
21231	87820020	Caralpha 20mg/12.5mg tablets (Actavis UK Ltd)
6786	50823020	Lisinopril 10mg / Hydrochlorothiazide 12.5mg tablets
55399	67847020	Lisinopril 20mg / Hydrochlorothiazide 12.5mg tablets (A A H Pharmaceuticals Ltd)
33353	67697020	Lisinopril 20mg / Hydrochlorothiazide 12.5mg tablets (Teva UK Ltd)
56244	1491020	Lisinopril 20mg / Hydrochlorothiazide 12.5mg tablets (Tillomed Laboratories Ltd)
37710	67694020	Lisinopril 10mg / Hydrochlorothiazide 12.5mg tablets (Teva UK Ltd)
39137	95875020	Zestoretic 10 tablets (AstraZeneca UK Ltd)
9764	72035020	Carace 20 Tablet (Bristol-Myers Squibb Pharmaceuticals Ltd)

For peer review only

17655	72036020	Carace 10 Tablet (Bristol-Myers Squibb Pharmaceuticals Ltd)
39147	95901020	Carace 20 Plus tablets (Merck Sharp & Dohme Ltd)
6359	51463020	Zestoretic 10- 10mg+12.5mg Tablet (AstraZeneca UK Ltd)
39242	95903020	Carace 10 Plus tablets (Merck Sharp & Dohme Ltd)
2982	51462020	Zestoretic 20- 20mg+12.5mg Tablet (AstraZeneca UK Ltd)
57539	1503020	Zestoretic 10 tablets (Sigma Pharmaceuticals Plc)
6468	50822020	Lisinopril 20mg / Hydrochlorothiazide 12.5mg tablets
54201	75924020	Lisinopril 20mg / Hydrochlorothiazide 12.5mg tablets (Almus Pharmaceuticals Ltd)
15121	80674020	Moexipril 7.5mg tablets
17120	80675020	Moexipril 15mg tablets
28725	80672020	Perdix 15mg tablets (UCB Pharma Ltd)
28724	80671020	Perdix 7.5mg tablets (UCB Pharma Ltd)
51807	38770020	Coversyl Arginine 5mg tablets (DE Pharmaceuticals)
37930	94699020	Perindopril arginine 5mg tablets
37971	94701020	Perindopril arginine 10mg tablets
38034	94703020	Coversyl Arginine 2.5mg tablets (Servier Laboratories Ltd)
50347	34964020	Coversyl Arginine 5mg tablets (Waymade Healthcare Plc)
37964	94697020	Perindopril arginine 2.5mg tablets
38026	94707020	Coversyl Arginine 10mg tablets (Servier Laboratories Ltd)
37965	94705020	Coversyl Arginine 5mg tablets (Servier Laboratories Ltd)
51258	34961020	Coversyl Arginine Plus 5mg/1.25mg tablets (DE Pharmaceuticals)
37908	94709020	Coversyl Arginine Plus 5mg/1.25mg tablets (Servier Laboratories Ltd)
48214	76375020	Perindopril erbumine 4mg tablets (Actavis UK Ltd)
59972	1543020	Perindopril erbumine 2mg tablets (Alliance Healthcare (Distribution) Ltd)
11983	90557020	Perindopril erbumine 4mg/5ml oral suspension
5800	72569020	Coversyl 4mg tablets (Servier Laboratories Ltd)
61270	45151020	Perindopril erbumine 4mg tablets (Accord Healthcare Ltd)
14960	83699020	Coversyl 8mg tablets (Servier Laboratories Ltd)
59770	15619021	Perindopril erbumine 4mg tablets (Aurobindo Pharma Ltd)
48049	76554020	Perindopril erbumine 2mg tablets (Generics (UK) Ltd)
58874	1550020	Perindopril erbumine 2mg tablets (Somex Pharma)
53058	40612020	Perindopril erbumine 8mg tablets (Sandoz Ltd)
58843	1542020	Perindopril erbumine 2mg tablets (Kent Pharmaceuticals Ltd)
56162	1556020	Perindopril erbumine 4mg tablets (Consilient Health Ltd)
45938	75040020	Perindopril erbumine 8mg tablets (Teva UK Ltd)
56473	1546020	Perindopril erbumine 2mg tablets (Sigma Pharmaceuticals Plc)
56472	1554020	Perindopril erbumine 4mg tablets (Kent Pharmaceuticals Ltd)
60065	1557020	Perindopril erbumine 4mg tablets (Sigma Pharmaceuticals Plc)
97	72573020	Perindopril erbumine 4mg tablets
38510	74776020	Perindopril erbumine 4mg tablets (Apotex UK Ltd)
57801	1558020	Perindopril erbumine 4mg tablets (Glenmark Generics (Europe) Ltd)
5612	72568020	Coversyl 2mg tablets (Servier Laboratories Ltd)
56516	75649020	Perindopril erbumine 2mg tablets (Sandoz Ltd)
50607	92909020	Perindopril arginine 2mg with Indapamide 625 micrograms tablet
6078	79311020	Perindopril erbumine 8mg tablets
49491	1545020	Perindopril erbumine 2mg tablets (Consilient Health Ltd)
57701	76377020	Perindopril erbumine 8mg tablets (Actavis UK Ltd)
61693	15724021	Perindopril erbumine 8mg tablets (Aurobindo Pharma Ltd)
48098	99501020	Perindopril arginine 4mg with Indapamide 1.25mg tablet
33095	73493020	Perindopril erbumine 4mg tablets (A A H Pharmaceuticals Ltd)
43012	91957020	Perindopril erbumine oral solution

For peer review only

35731	73496020	Perindopril erbumine 8mg tablets (A A H Pharmaceuticals Ltd)
54733	9855020	Perindopril erbumine 8mg tablets (Consilient Health Ltd)
59790	39906020	Perindopril erbumine 8mg tablets (Accord Healthcare Ltd)
54899	75033020	Perindopril erbumine 2mg tablets (Teva UK Ltd)
50402	74621020	Perindopril 2mg Tablet (Servier Laboratories Ltd)
48180	75652020	Perindopril erbumine 4mg tablets (Sandoz Ltd)
593	72572020	Perindopril erbumine 2mg tablets
54942	76559020	Perindopril erbumine 8mg tablets (Generics (UK) Ltd)
43813	76373020	Perindopril erbumine 2mg tablets (Actavis UK Ltd)
56508	1552020	Coversyl 4mg tablets (Dowelhurst Ltd)
56506	1541020	Coversyl 2mg tablets (Dowelhurst Ltd)
45319	73490020	Perindopril erbumine 2mg tablets (A A H Pharmaceuticals Ltd)
38285	75036020	Perindopril erbumine 4mg tablets (Teva UK Ltd)
60067	22976021	Perindopril erbumine 4mg / Amlodipine 5mg tablets
60684	22974021	Perindopril erbumine 4mg / Amlodipine 10mg tablets
57333	47942020	Perindopril tosilate 5mg tablets
15096	75604020	Accupro 40mg tablets (Pfizer Ltd)
14478	69845020	Accupro 20mg tablets (Pfizer Ltd)
6765	69848020	Quinapril 5mg tablets
7314	69843020	Accupro 5mg tablets (Pfizer Ltd)
61292	68165020	Quinapril 40mg tablets (Generics (UK) Ltd)
14477	69844020	Accupro 10mg tablets (Pfizer Ltd)
46365	89048020	Quinil 20mg tablets (Tillomed Laboratories Ltd)
3929	69849020	Quinapril 10mg tablets
42285	89050020	Quinil 40mg tablets (Tillomed Laboratories Ltd)
38854	95370020	Quinapril 20mg/5ml oral solution
5159	69850020	Quinapril 20mg tablets
40355	89044020	Quinil 5mg tablets (Tillomed Laboratories Ltd)
9731	75607020	Quinapril 40mg tablets
38899	89046020	Quinil 10mg tablets (Tillomed Laboratories Ltd)
28586	88107020	Lopace 5mg capsules (Discovery Pharmaceuticals Ltd)
52407	1585020	Ramipril 10mg capsules (Kent Pharmaceuticals Ltd)
709	72675020	Ramipril 2.5mg capsules
57658	67312020	Ramipril 1.25mg tablets (A A H Pharmaceuticals Ltd)
47998	68974020	Ramipril 2.5mg capsules (Actavis UK Ltd)
52399	1564020	Ramipril 1.25mg capsules (Kent Pharmaceuticals Ltd)
38308	95107020	Ramipril 2.5/5mg/10mg tablet
34382	67206020	Ramipril 5mg capsules (Zentiva)
654	80174020	Ramipril 2.5/5mg/10mg capsule
45264	68971020	Ramipril 1.25mg capsules (Actavis UK Ltd)
34652	67455020	Ramipril 5mg Capsule (Sovereign Medical Ltd)
29627	88105020	Lopace 2.5mg capsules (Discovery Pharmaceuticals Ltd)
5275	72670020	Tritace 2.5mg capsules (Sanofi)
49164	1588020	Ramipril 10mg capsules (Actavis UK Ltd)
61694	67907020	Ramipril 5mg tablets (Zentiva)
11937	90539020	Ramipril 2.5mg/5ml oral suspension
56855	1590020	Ramipril 10mg capsules (Sigma Pharmaceuticals Plc)
9915	87063020	Tritace 10mg tablets (Sanofi)
34390	67186020	Ramipril 5mg capsules (Genus Pharmaceuticals Ltd)
34412	67140020	Ramipril 5mg capsules (Teva UK Ltd)
56129	1578020	Ramipril 5mg capsules (Kent Pharmaceuticals Ltd)
34657	67209020	Ramipril 10mg capsules (Zentiva)
42081	87446020	Tritace 1.25mg Tablet (Sterwin Medicines)
57073	47143020	Ramipril 1.25mg capsules (Waymade Healthcare Plc)

For peer review only

54620	1575020	Ramipril 2.5mg capsules (Sigma Pharmaceuticals Plc)
52197	1582020	Ramipril 5mg capsules (Sigma Pharmaceuticals Plc)
6364	87059020	Tritace 2.5mg tablets (Sanofi)
56148	10683020	Ramipril 1.25mg tablets (Kent Pharmaceuticals Ltd)
9646	72669020	Tritace 1.25mg capsules (Aventis Pharma)
55299	67297020	Ramipril 1.25mg capsules (A A H Pharmaceuticals Ltd)
82	70737020	Ramipril 10mg capsules
59603	1577020	Ramipril 2.5mg capsules (Phoenix Healthcare Distribution Ltd)
147	72674020	Ramipril 1.25mg capsules
61339	73446020	Ramipril 10mg capsules (Almus Pharmaceuticals Ltd)
34732	67512020	Ramipril 2.5mg Capsule (Dexcel-Pharma Ltd)
61499	73468020	Ramipril 2.5mg tablets (Actavis UK Ltd)
34540	67305020	Ramipril 5mg capsules (A A H Pharmaceuticals Ltd)
45340	68982020	Ramipril 10mg Capsule (Actavis UK Ltd)
50509	20723020	Ramipril 10mg/5ml oral solution
9693	55352020	Tritace 10mg capsules (Sanofi)
57235	74497020	Ramipril 1.25mg tablets (Sandoz Ltd)
80	72676020	Ramipril 5mg capsules
34431	67203020	Ramipril 2.5mg capsules (Zentiva)
55798	47145020	Ramipril 5mg capsules (Waymade Healthcare Plc)
45554	99318020	Ramipril 5mg/5ml oral solution
33811	69974020	Ramipril 2.5mg capsules (Ranbaxy (UK) Ltd)
32857	67125020	Ramipril 1.25mg capsules (Teva UK Ltd)
54298	70068020	Ramipril 2.5mg capsules (Arrow Generics Ltd)
56763	1592020	Ramipril 10mg capsules (Phoenix Healthcare Distribution Ltd)
54941	1579020	Ramipril 5mg capsules (Alliance Healthcare (Distribution) Ltd)
34357	67189020	Ramipril 10mg capsules (Genus Pharmaceuticals Ltd)
5735	72671020	Tritace 5mg capsules (Sanofi)
34893	68264020	Ramipril 10mg Capsule (IVAX Pharmaceuticals UK Ltd)
51714	1572020	Ramipril 2.5mg capsules (Alliance Healthcare (Distribution) Ltd)
40384	67322020	Ramipril 10mg tablets (A A H Pharmaceuticals Ltd)
62039	67901020	Ramipril 1.25mg tablets (Zentiva)
56704	1565020	Ramipril 1.25mg capsules (Alliance Healthcare (Distribution) Ltd)
6288	87053020	Ramipril 5mg tablets
34567	69436020	Ramipril 2.5mg capsules (Generics (UK) Ltd)
34528	67301020	Ramipril 2.5mg capsules (A A H Pharmaceuticals Ltd)
34589	67515020	Ramipril 5mg Capsule (Dexcel-Pharma Ltd)
53612	10700020	Ramipril 10mg tablets (Alliance Healthcare (Distribution) Ltd)
47021	475021	Ramipril 2.5mg/5ml oral solution sugar free
62036	47656020	Ramipril 5mg tablets (Waymade Healthcare Plc)
57864	10698020	Ramipril 5mg tablets (Sigma Pharmaceuticals Plc)
756	87055020	Ramipril 10mg tablets
34943	67309020	Ramipril 10mg capsules (A A H Pharmaceuticals Ltd)
61067	73443020	Ramipril 5mg capsules (Almus Pharmaceuticals Ltd)
34429	69439020	Ramipril 5mg capsules (Generics (UK) Ltd)
56038	39749020	Ramipril 10mg tablets (Pfizer Ltd)
34432	67183020	Ramipril 2.5mg capsules (Genus Pharmaceuticals Ltd)
35007	91818020	Ramipril 10mg/5ml oral suspension
57346	47146020	Ramipril 10mg capsules (Waymade Healthcare Plc)
34877	67460020	Ramipril 10mg Capsule (Sovereign Medical Ltd)
34539	67175020	Ramipril 5mg capsules (Sandoz Ltd)
34698	67770020	Ramipril 1.25mg capsules (Zentiva)
34583	67518020	Ramipril 10mg Capsule (Dexcel-Pharma Ltd)
34490	67129020	Ramipril 2.5mg capsules (Teva UK Ltd)

For peer review only

34505	67170020	Ramipril 2.5mg capsules (Sandoz Ltd)
46890	477021	Ramipril 5mg/5ml oral suspension
61985	67121020	Ramipril 1.25mg tablets (Teva UK Ltd)
32934	88109020	Lopace 10mg capsules (Discovery Pharmaceuticals Ltd)
33894	67144020	Ramipril 10mg capsules (Teva UK Ltd)
761	87049020	Ramipril 1.25mg tablets
34651	69441020	Ramipril 10mg capsules (Generics (UK) Ltd)
59557	1571020	Ramipril 2.5mg capsules (Kent Pharmaceuticals Ltd)
56013	47144020	Ramipril 2.5mg capsules (Waymade Healthcare Plc)
48008	68978020	Ramipril 5mg capsules (Actavis UK Ltd)
56356	1586020	Ramipril 10mg capsules (Alliance Healthcare (Distribution) Ltd)
59788	1591020	Ramipril 10mg capsules (Bristol Laboratories Ltd)
48053	73269020	Ramipril 2.5mg capsules (Almus Pharmaceuticals Ltd)
51701	1583020	Ramipril 5mg capsules (Bristol Laboratories Ltd)
60730	1584020	Ramipril 5mg capsules (Phoenix Healthcare Distribution Ltd)
39355	87452020	Tritace 10mg Tablet (Sterwin Medicines)
6362	87061020	Tritace 5mg tablets (Sanofi)
6261	87057020	Tritace 1.25mg tablets (Sanofi)
6314	87051020	Ramipril 2.5mg tablets
34710	67179020	Ramipril 10mg capsules (Sandoz Ltd)
53621	1576020	Ramipril 2.5mg capsules (Bristol Laboratories Ltd)
56169	70076020	Ramipril 10mg capsules (Arrow Generics Ltd)
31810	68138020	Odrik 1mg capsules (Aventis Pharma)
60757	75136020	Trandolapril 500microgram capsules (Teva UK Ltd)
7419	69080020	Trandolapril 500microgram capsules
16710	71438020	Gopten 500microgram capsules (Abbott Laboratories Ltd)
29130	87608020	Gopten 4mg capsules (Abbott Laboratories Ltd)
4103	69081020	Trandolapril 1mg capsules
9948	87606020	Trandolapril 4mg capsules
54345	10867020	Trandolapril 4mg capsules (Arrow Generics Ltd)
5047	69082020	Trandolapril 2mg capsules
8025	71439020	Gopten 1mg capsules (Abbott Laboratories Ltd)
28902	68139020	Odrik 2mg capsules (Aventis Pharma)
8026	71440020	Gopten 2mg capsules (Abbott Laboratories Ltd)
31307	68137020	Odrik 500microgram capsules (Aventis Pharma)
19690	85865020	Verapamil 180mg modified-release / Trandolapril 2mg capsules
20579	85903020	Tarka modified-release capsules (Abbott Laboratories Ltd)
6200	85253020	Tritace titration pack capsules (Sanofi)
39421	95113020	Tritace titration pack tablets (Sanofi)
24214	6272007	TRITACE 1.25 MG TAB
29964	5959007	TRITACE 2.5 MG TAB
31288	!8505226	TRITACE

For peer review only

Alpha blockers

Product code	GEM code	Description
26238	51808020	Alphavase 1 tablets (Ashbourne Pharmaceuticals Ltd)
13610	51809020	Alphavase 2 tablets (Ashbourne Pharmaceuticals Ltd)
19823	51736020	Alphavase 5 tablets (Ashbourne Pharmaceuticals Ltd)
26237	51807020	Alphavase 500microgram Tablet (Ashbourne Pharmaceuticals Ltd)
11394	48295020	Baratol 25mg Tablet (Shire Pharmaceuticals Ltd)
40256	96646020	Baratol 25mg tablets (Amdipharm Plc)
16198	48296020	Baratol 50mg Tablet (Shire Pharmaceuticals Ltd)
36023	92057020	Cardozin xl 4mg Tablet (Hillcross Pharmaceuticals Ltd)
37243	93699020	Cardozin xl 4mg Tablet (Teva UK Ltd)
46066	99273020	Cardozin XL 4mg tablets (Almus Pharmaceuticals Ltd)
38461	93900020	Cardozin XL 4mg tablets (Arrow Generics Ltd)
4449	69313020	Cardura 1mg tablets (Pfizer Ltd)
4802	69314020	Cardura 2mg tablets (Pfizer Ltd)
8086	69315020	Cardura 4mg Tablet (Pfizer Ltd)
755	85762020	Cardura XL 4mg tablets (Pfizer Ltd)
5618	85763020	Cardura XL 8mg tablets (Pfizer Ltd)
25487	80419020	Cascor 2mg tablets (Ranbaxy (UK) Ltd)
25551	80421020	Cascor 4mg tablets (Ranbaxy (UK) Ltd)
43695	93902020	Colixil XL 4mg tablets (Sandoz Ltd)
8942	48961020	Dibenyline 10mg capsules (Mercury Pharma Group Ltd)
21049	5083007	DIBENYLINE 2.5 MG CAP
29765	5552007	DIBENYLINE 5 MG TAB
7549	58779020	Doxadura 1mg tablets (Discovery Pharmaceuticals Ltd)
7547	83686020	Doxadura 2mg tablets (Discovery Pharmaceuticals Ltd)
10088	83886020	Doxadura 4mg tablets (Discovery Pharmaceuticals Ltd)
35272	93137020	Doxadura XL 4mg tablets (Discovery Pharmaceuticals Ltd)
119	69308020	Doxazosin 1mg tablets
34715	64168020	Doxazosin 1mg tablets (A A H Pharmaceuticals Ltd)
48150	65746020	Doxazosin 1mg tablets (Actavis UK Ltd)
55916	1340020	Doxazosin 1mg tablets (Alliance Healthcare (Distribution) Ltd)
62019	71463020	Doxazosin 1mg tablets (Almus Pharmaceuticals Ltd)
60319	1344020	Doxazosin 1mg tablets (Bristol Laboratories Ltd)
55906	64180020	Doxazosin 1mg tablets (Dexcel-Pharma Ltd)
34601	64033020	Doxazosin 1mg tablets (Generics (UK) Ltd)
41543	64244020	Doxazosin 1mg tablets (IVAX Pharmaceuticals UK Ltd)
59209	65452020	Doxazosin 1mg tablets (Kent Pharmaceuticals Ltd)
45328	64311020	Doxazosin 1mg tablets (Sandoz Ltd)
34342	64067020	Doxazosin 1mg tablets (Teva UK Ltd)
20369	90119020	Doxazosin 1mg/5ml oral suspension
493	69309020	Doxazosin 2mg tablets
34625	64171020	Doxazosin 2mg tablets (A A H Pharmaceuticals Ltd)
56145	65760020	Doxazosin 2mg tablets (Actavis UK Ltd)
50467	1346020	Doxazosin 2mg tablets (Alliance Healthcare (Distribution) Ltd)
61066	1353020	Doxazosin 2mg tablets (Bristol Laboratories Ltd)
45583	64184020	Doxazosin 2mg tablets (Dexcel-Pharma Ltd)
33094	64037020	Doxazosin 2mg tablets (Generics (UK) Ltd)
40891	64251020	Doxazosin 2mg tablets (IVAX Pharmaceuticals UK Ltd)
58276	38497020	Doxazosin 2mg tablets (Medreich Plc)
57074	1352020	Doxazosin 2mg tablets (Sigma Pharmaceuticals Plc)
19193	64070020	Doxazosin 2mg tablets (Teva UK Ltd)

For peer review only

57784	30736020	Doxazosin 2mg/5ml oral suspension
582	84705020	Doxazosin 4mg modified-release tablets
1294	69310020	Doxazosin 4mg tablets
57448	64175020	Doxazosin 4mg tablets (A A H Pharmaceuticals Ltd)
51685	65764020	Doxazosin 4mg tablets (Actavis UK Ltd)
61283	1358020	Doxazosin 4mg tablets (Alliance Healthcare (Distribution) Ltd)
62158	71469020	Doxazosin 4mg tablets (Almus Pharmaceuticals Ltd)
53322	1362020	Doxazosin 4mg tablets (Bristol Laboratories Ltd)
60200	21704021	Doxazosin 4mg tablets (DE Pharmaceuticals)
59862	64188020	Doxazosin 4mg tablets (Dexcel-Pharma Ltd)
34553	64041020	Doxazosin 4mg tablets (Generics (UK) Ltd)
19216	64260020	Doxazosin 4mg tablets (IVAX Pharmaceuticals UK Ltd)
54785	38498020	Doxazosin 4mg tablets (Medreich Plc)
58325	1363020	Doxazosin 4mg tablets (Phoenix Healthcare Distribution Ltd)
45342	64318020	Doxazosin 4mg tablets (Sandoz Ltd)
40678	64073020	Doxazosin 4mg tablets (Teva UK Ltd)
61123	20657021	Doxazosin 4mg/5ml oral solution
35603	93725020	Doxazosin 4mg/5ml oral suspension
5496	84706020	Doxazosin 8mg modified-release tablets
45265	75714020	Doxazosin sr 4mg Tablet (Generics (UK) Ltd)
47807	77113020	Doxazosin xl 4mg Tablet (Hillcross Pharmaceuticals Ltd)
53033	8020	Doxzogen XL 4mg tablets (Generics (UK) Ltd)
19566	!3295102	HYPOVASE
1292	49881020	Hypovase 1mg tablets (Pfizer Ltd)
5183	49882020	Hypovase 2mg tablets (Pfizer Ltd)
4111	49880020	Hypovase 500microgram tablets (Pfizer Ltd)
8198	49886020	Hypovase 5mg Tablet (Pfizer Ltd)
24211	!3296101	HYPOVASE B.D. STARTER PACK
8863	68288020	Hypovase benign prostatic hyperplasia 1mg Tablet (Pfizer Ltd)
23459	68291020	Hypovase benign prostatic hyperplasia 2mg Tablet (Pfizer Ltd)
25047	68287020	Hypovase benign prostatic hyperplasia 500microgram Tablet (Pfizer Ltd)
26693	68286020	Hypovase benign prostatic hyperplasia bd BD Starter pack (Pfizer Ltd)
12518	60761020	Hypovase tablets B.D. starter pack (Pfizer Ltd)
2347	68066020	Hytrin 10mg Tablet (Abbott Laboratories Ltd)
37428	93864020	Hytrin 10mg tablets (AMCo)
8077	68062020	Hytrin 2mg Tablet (Abbott Laboratories Ltd)
36649	93860020	Hytrin 2mg tablets (AMCo)
2346	68063020	Hytrin 5mg Tablet (Abbott Laboratories Ltd)
36780	93862020	Hytrin 5mg tablets (AMCo)
2348	51017020	Hytrin bph 10mg Tablet (Amdipharm Plc)
16201	51572020	Hytrin bph 2mg Tablet (Amdipharm Plc)
5337	51573020	Hytrin bph 5mg Tablet (Amdipharm Plc)
2345	51571020	Hytrin BPH tablets starter pack (AMCo)
8076	72781020	Hytrin tablets starter pack (AMCo)
2816	69659020	Indoramin 20mg tablets
5815	63556020	Indoramin 25mg tablets
9019	63557020	Indoramin 50mg Tablet
45040	99157020	Larbex XL 4mg tablets (Teva UK Ltd)
45584	72200020	Phenoxybenzamine 100mg/2ml solution for infusion ampoules
7759	65687020	Phenoxybenzamine 10mg capsules
40810	94251020	Phenoxybenzamine 10mg/5ml oral suspension
25004	4577007	PHENOXYBENZAMINE HCl 2.5 MG CAP
21466	4576007	PHENOXYBENZAMINE HCl 5 MG CAP
26783	5477007	PHENOXYBENZAMINE HCl 5 MG TAB

For peer review only

54918	93155020	Phenoxybenzamine oral liquid
12545	65694020	Phentolamine 10mg/1ml solution for injection ampoules
60316	54584020	Prazosin 1mg Tablet (Approved Prescription Services Ltd)
591	66154020	Prazosin 1mg tablets
41721	54210020	Prazosin 1mg tablets (A A H Pharmaceuticals Ltd)
46922	54223020	Prazosin 1mg tablets (IVAX Pharmaceuticals UK Ltd)
445	74153020	Prazosin 1mg tablets and Prazosin 500microgram tablets
726	66155020	Prazosin 2mg tablets
41651	54583020	Prazosin 500microgram Tablet (Approved Prescription Services Ltd)
1455	66153020	Prazosin 500microgram tablets
41652	54209020	Prazosin 500microgram tablets (A A H Pharmaceuticals Ltd)
43547	54222020	Prazosin 500microgram tablets (IVAX Pharmaceuticals UK Ltd)
3715	66158020	Prazosin 5mg tablets
55826	54216020	Prazosin 5mg tablets (A A H Pharmaceuticals Ltd)
46526	99138020	Raporsin XL 4mg tablets (Actavis UK Ltd)
23010	65697020	Rogitine 10mg/1ml solution for injection ampoules (Alliance Pharmaceuticals Ltd)
36740	92709020	Slocinx XL 4mg tablets (Zentiva)
4875	68058020	Terazosin 10mg tablets
3470	68054020	Terazosin 1mg tablets
4637	68055020	Terazosin 2mg tablets
57145	65774020	Terazosin 2mg tablets (A A H Pharmaceuticals Ltd)
4694	72784020	Terazosin 2mg tablets and Terazosin 1mg tablets
3924	68056020	Terazosin 5mg tablets
3923	83746020	Terazosin BPH starter pack 7x1mg with 14x2mg with 7x5mg

For peer review only

Angiotensin II receptor antagonists

Product code	GEM code	Description
5117	86113020	Amias 16mg tablets (Takeda UK Ltd)
4155	79053020	Amias 2mg tablets (Takeda UK Ltd)
31072	88790020	Amias 32mg tablets (Takeda UK Ltd)
4685	79054020	Amias 4mg tablets (Takeda UK Ltd)
5013	79055020	Amias 8mg tablets (Takeda UK Ltd)
35189	92767020	Amlodipine 10mg / Valsartan 160mg tablets
35343	92765020	Amlodipine 5mg / Valsartan 160mg tablets
35329	92763020	Amlodipine 5mg / Valsartan 80mg tablets
9196	85710020	Aprovel 150mg tablets (Sanofi)
11348	85711020	Aprovel 300mg tablets (Sanofi)
7338	85709020	Aprovel 75mg tablets (Sanofi)
4741	81321020	Candesartan 16mg tablets
52208	41322020	Candesartan 16mg tablets (A A H Pharmaceuticals Ltd)
57977	41323020	Candesartan 16mg tablets (Alliance Healthcare (Distribution) Ltd)
54414	41324020	Candesartan 16mg tablets (Consilient Health Ltd)
53680	41320020	Candesartan 16mg tablets (Teva UK Ltd)
62035	47163020	Candesartan 16mg tablets (Waymade Healthcare Plc)
529	81323020	Candesartan 2mg tablets
57266	46326020	Candesartan 2mg tablets (Actavis UK Ltd)
59802	41901020	Candesartan 2mg tablets (Teva UK Ltd)
7043	88788020	Candesartan 32mg tablets
54326	41435020	Candesartan 32mg tablets (Teva UK Ltd)
531	81324020	Candesartan 4mg tablets
58646	41311020	Candesartan 4mg tablets (Actavis UK Ltd)
51647	1764020	Candesartan 4mg tablets (Mawdsley-Brooks & Company Ltd)
62140	25450021	Candesartan 4mg tablets (Sandoz Ltd)
53755	41310020	Candesartan 4mg tablets (Teva UK Ltd)
4818	81325020	Candesartan 8mg tablets
51519	41317020	Candesartan 8mg tablets (A A H Pharmaceuticals Ltd)
57273	41316020	Candesartan 8mg tablets (Actavis UK Ltd)
59690	41319020	Candesartan 8mg tablets (Consilient Health Ltd)
51117	1771020	Candesartan 8mg tablets (DE Pharmaceuticals)
50185	41315020	Candesartan 8mg tablets (Teva UK Ltd)
57026	47162020	Candesartan 8mg tablets (Waymade Healthcare Plc)
52559	42156020	Candesartan 8mg tablets (Zentiva)
10316	83981020	CoAprovel 150mg/12.5mg tablets (Sanofi)
11526	83982020	CoAprovel 300mg/12.5mg tablets (Sanofi)
35196	92367020	CoAprovel 300mg/25mg tablets (Sanofi)
6877	87998020	Co-Diovan 160mg/12.5mg tablets (Novartis Pharmaceuticals UK Ltd)
25382	88008020	Co-Diovan 160mg/25mg tablets (Novartis Pharmaceuticals UK Ltd)
764	88424020	Co-Diovan 80mg/12.5mg tablets (Novartis Pharmaceuticals UK Ltd)
52858	10088020	Co-Diovan 80mg/12.5mg tablets (Sigma Pharmaceuticals Plc)
14965	80730020	Cozaar 100mg tablets (Merck Sharp & Dohme Ltd)
52427	10518020	Cozaar 100mg tablets (Necessity Supplies Ltd)
40571	96965020	Cozaar 12.5mg tablets (Merck Sharp & Dohme Ltd)
41232	97245020	Cozaar 2.5mg/ml oral suspension (Merck Sharp & Dohme Ltd)
4226	76329020	Cozaar 25mg tablets (Merck Sharp & Dohme Ltd)
5723	76328020	Cozaar 50mg tablets (Merck Sharp & Dohme Ltd)
37747	94429020	Cozaar-Comp 100mg/12.5mg tablets (Merck Sharp & Dohme Ltd)
21423	89783020	Cozaar-Comp 100mg/25mg tablets (Merck Sharp & Dohme Ltd)
57796	1684020	Cozaar-Comp 50mg/12.5mg tablets (DE Pharmaceuticals)

For peer review only

4540	82246020	Cozaar-Comp 50mg/12.5mg tablets (Merck Sharp & Dohme Ltd)
55160	1673020	Cozaar-Comp 50mg/12.5mg tablets (Sigma Pharmaceuticals Plc)
6518	83507020	Diovan 160mg capsules (Novartis Pharmaceuticals UK Ltd)
45600	98993020	Diovan 160mg Tablet (Novartis Pharmaceuticals UK Ltd)
39199	94445020	Diovan 320mg tablets (Novartis Pharmaceuticals UK Ltd)
11251	83505020	Diovan 40mg capsules (Novartis Pharmaceuticals UK Ltd)
24359	88544020	Diovan 40mg tablets (Novartis Pharmaceuticals UK Ltd)
11252	83506020	Diovan 80mg capsules (Novartis Pharmaceuticals UK Ltd)
6939	84000020	Eprosartan 300mg tablets
13123	84001020	Eprosartan 400mg tablets
12836	84002020	Eprosartan 600mg tablets
35096	92773020	Exforge 10mg/160mg tablets (Novartis Pharmaceuticals UK Ltd)
35697	92771020	Exforge 5mg/160mg tablets (Novartis Pharmaceuticals UK Ltd)
35317	92769020	Exforge 5mg/80mg tablets (Novartis Pharmaceuticals UK Ltd)
60780	38011020	Generic Sevikar HCT 20mg/5mg/12.5mg tablets
60007	38012020	Generic Sevikar HCT 40mg/10mg/12.5mg tablets
38367	94427020	Hydrochlorothiazide with losartan 12.5mg with 100mg Tablet
14738	79035020	Hydrochlorothiazide with losartan 12.5mg with 50mg Tablet
24632	89781020	Hydrochlorothiazide with losartan 25mg with 100mg Tablet
24484	87996020	Hydrochlorothiazide with valsartan 12.5mg with 160mg Tablet
24268	88422020	Hydrochlorothiazide with valsartan 12.5mg with 80mg Tablet
23456	88006020	Hydrochlorothiazide with valsartan 25mg with 160mg Tablet
31160	!8502589	IRBESARTAN
11448	80124020	Irbesartan 150mg / Hydrochlorothiazide 12.5mg tablets
1293	85706020	Irbesartan 150mg tablets
58108	44383020	Irbesartan 150mg tablets (A A H Pharmaceuticals Ltd)
58201	44381020	Irbesartan 150mg tablets (Actavis UK Ltd)
60597	43819020	Irbesartan 150mg tablets (Teva UK Ltd)
11469	80125020	Irbesartan 300mg / Hydrochlorothiazide 12.5mg tablets
35481	92365020	Irbesartan 300mg / Hydrochlorothiazide 25mg tablets
2971	85707020	Irbesartan 300mg tablets
55017	45155020	Irbesartan 300mg tablets (Accord Healthcare Ltd)
59393	47159020	Irbesartan 300mg tablets (Sandoz Ltd)
52972	1748020	Irbesartan 300mg tablets (Sigma Pharmaceuticals Plc)
61781	43823020	Irbesartan 300mg tablets (Teva UK Ltd)
36939	94158020	Irbesartan 300mg/5ml oral suspension
828	85705020	Irbesartan 75mg tablets
37650	94425020	Losartan 100mg / Hydrochlorothiazide 12.5mg tablets
48039	78259020	Losartan 100mg / Hydrochlorothiazide 12.5mg tablets (Teva UK Ltd)
10323	89779020	Losartan 100mg / Hydrochlorothiazide 25mg tablets
52189	10152020	Losartan 100mg / Hydrochlorothiazide 25mg tablets (A A H Pharmaceuticals Ltd)
624	79160020	Losartan 100mg tablets
49588	10510020	Losartan 100mg tablets (A A H Pharmaceuticals Ltd)
61288	10519020	Losartan 100mg tablets (Accord Healthcare Ltd)
54404	77737020	Losartan 100mg tablets (Actavis UK Ltd)
61053	10513020	Losartan 100mg tablets (Alliance Healthcare (Distribution) Ltd)
55446	43882020	Losartan 100mg tablets (Bristol Laboratories Ltd)
60506	77323020	Losartan 100mg tablets (Dexcel-Pharma Ltd)
57028	10512020	Losartan 100mg tablets (Generics (UK) Ltd)
56970	10517020	Losartan 100mg tablets (Pfizer Ltd)
47006	77302020	Losartan 100mg tablets (Teva UK Ltd)
52658	36312020	Losartan 100mg/5ml oral suspension
39944	96963020	Losartan 12.5mg tablets

52886	35998020	Losartan 12.5mg tablets (A A H Pharmaceuticals Ltd)
58967	35999020	Losartan 12.5mg tablets (Alliance Healthcare (Distribution) Ltd)
59340	77317020	Losartan 12.5mg tablets (Dexcel-Pharma Ltd)
40711	97243020	Losartan 2.5mg/ml oral suspension sugar free
520	76333020	Losartan 25mg tablets
50971	1640020	Losartan 25mg tablets (A A H Pharmaceuticals Ltd)
58274	1646020	Losartan 25mg tablets (Accord Healthcare Ltd)
54740	77731020	Losartan 25mg tablets (Actavis UK Ltd)
61495	14777021	Losartan 25mg tablets (Aptil Pharma Ltd)
51186	1643020	Losartan 25mg tablets (Arrow Generics Ltd)
58649	43813020	Losartan 25mg tablets (Bristol Laboratories Ltd)
48398	77320020	Losartan 25mg tablets (Dexcel-Pharma Ltd)
49492	1641020	Losartan 25mg tablets (Generics (UK) Ltd)
55718	1644020	Losartan 25mg tablets (Phoenix Healthcare Distribution Ltd)
59271	40515020	Losartan 25mg tablets (Sandoz Ltd)
59086	41899020	Losartan 25mg tablets (Wockhardt UK Ltd)
61754	35460020	Losartan 25mg/5ml oral suspension
6437	79786020	Losartan 50mg / Hydrochlorothiazide 12.5mg tablets
56975	1677020	Losartan 50mg / Hydrochlorothiazide 12.5mg tablets (A A H Pharmaceuticals Ltd)
56204	77510020	Losartan 50mg / Hydrochlorothiazide 12.5mg tablets (Actavis UK Ltd)
1780	76332020	Losartan 50mg tablets
56104	1658020	Losartan 50mg tablets (A A H Pharmaceuticals Ltd)
54049	1669020	Losartan 50mg tablets (Accord Healthcare Ltd)
51601	77734020	Losartan 50mg tablets (Actavis UK Ltd)
54735	1661020	Losartan 50mg tablets (Alliance Healthcare (Distribution) Ltd)
59750	14778021	Losartan 50mg tablets (Aptil Pharma Ltd)
54843	77349020	Losartan 50mg tablets (Dexcel-Pharma Ltd)
55296	1660020	Losartan 50mg tablets (Generics (UK) Ltd)
59351	1666020	Losartan 50mg tablets (Pfizer Ltd)
54057	77299020	Losartan 50mg tablets (Teva UK Ltd)
52659	35425020	Losartan 50mg/5ml oral solution
59903	35427020	Losartan 50mg/5ml oral suspension
17686	81476020	Micardis 20mg tablets (Boehringer Ingelheim Ltd)
13821	81474020	Micardis 40mg tablets (Boehringer Ingelheim Ltd)
17545	81475020	Micardis 80mg tablets (Boehringer Ingelheim Ltd)
18202	81233020	MicardisPlus 40mg/12.5mg tablets (Boehringer Ingelheim Ltd)
17689	49280020	MicardisPlus 80mg/12.5mg tablets (Boehringer Ingelheim Ltd)
38889	95336020	MicardisPlus 80mg/25mg tablets (Boehringer Ingelheim Ltd)
6217	78179020	Olmesartan medoxomil 10mg tablets
39786	96351020	Olmesartan medoxomil 10mg/5ml oral suspension
40316	96923020	Olmesartan medoxomil 20mg / Amlodipine 5mg tablets
18200	90629020	Olmesartan medoxomil 20mg / Hydrochlorothiazide 12.5mg tablets
18903	90631020	Olmesartan medoxomil 20mg / Hydrochlorothiazide 25mg tablets
6285	78212020	Olmesartan medoxomil 20mg tablets
40668	96927020	Olmesartan medoxomil 40mg / Amlodipine 10mg tablets
40639	96925020	Olmesartan medoxomil 40mg / Amlodipine 5mg tablets
43322	98337020	Olmesartan medoxomil 40mg / Hydrochlorothiazide 12.5mg tablets
6351	78176020	Olmesartan medoxomil 40mg tablets
14983	78194020	Olmetec 10mg tablets (Daiichi Sankyo UK Ltd)
18910	78191020	Olmetec 20mg tablets (Daiichi Sankyo UK Ltd)
20117	57407020	Olmetec 40mg tablets (Daiichi Sankyo UK Ltd)
29634	90635020	Olmetec Plus 20mg/12.5mg tablets (Daiichi Sankyo UK Ltd)
27520	90637020	Olmetec Plus 20mg/25mg tablets (Daiichi Sankyo UK Ltd)

For peer review only

43915	98339020	Olmetec Plus 40mg/12.5mg tablets (Daiichi Sankyo UK Ltd)
39984	96929020	Sevikar 20mg/5mg tablets (Daiichi Sankyo UK Ltd)
41203	96933020	Sevikar 40mg/10mg tablets (Daiichi Sankyo UK Ltd)
41205	96931020	Sevikar 40mg/5mg tablets (Daiichi Sankyo UK Ltd)
46355	99779020	Sevikar HCT 20mg/5mg/12.5mg tablets (Daiichi Sankyo UK Ltd)
47616	99785020	Sevikar HCT 40mg/10mg/12.5mg tablets (Daiichi Sankyo UK Ltd)
53220	99791020	Sevikar HCT 40mg/10mg/25mg tablets (Daiichi Sankyo UK Ltd)
47573	99783020	Sevikar HCT 40mg/5mg/12.5mg tablets (Daiichi Sankyo UK Ltd)
47727	99789020	Sevikar HCT 40mg/5mg/25mg tablets (Daiichi Sankyo UK Ltd)
6243	74674020	Telmisartan 20mg tablets
61177	33020	Telmisartan 20mg tablets (Sigma Pharmaceuticals Plc)
14870	81237020	Telmisartan 40mg / Hydrochlorothiazide 12.5mg tablets
5988	74672020	Telmisartan 40mg tablets
16161	58831020	Telmisartan 80mg / Hydrochlorothiazide 12.5mg tablets
38459	95334020	Telmisartan 80mg / Hydrochlorothiazide 25mg tablets
12874	74673020	Telmisartan 80mg tablets
9745	84004020	Teveten 300mg tablets (Abbott Healthcare Products Ltd)
16285	84005020	Teveten 400mg tablets (Abbott Healthcare Products Ltd)
16371	84006020	Teveten 600mg tablets (Abbott Healthcare Products Ltd)
11864	87994020	Valsartan 160mg / Hydrochlorothiazide 12.5mg tablets
14283	88002020	Valsartan 160mg / Hydrochlorothiazide 25mg tablets
4645	83503020	Valsartan 160mg capsules
55187	39798020	Valsartan 160mg capsules (Arrow Generics Ltd)
53833	38960020	Valsartan 160mg capsules (Generics (UK) Ltd)
61442	1715020	Valsartan 160mg capsules (Teva UK Ltd)
55821	78684020	Valsartan 160mg capsules (Teva UK Ltd)
60076	47156020	Valsartan 160mg capsules (Waymade Healthcare Plc)
44778	91979020	Valsartan 160mg tablets
37573	94443020	Valsartan 320mg tablets
59029	99475020	Valsartan 3mg/1ml oral solution
575	83501020	Valsartan 40mg capsules
58669	1689020	Valsartan 40mg capsules (Teva UK Ltd)
54726	78678020	Valsartan 40mg capsules (Teva UK Ltd)
14943	88542020	Valsartan 40mg tablets
16060	88420020	Valsartan 80mg / Hydrochlorothiazide 12.5mg tablets
3222	83502020	Valsartan 80mg capsules
59448	38828020	Valsartan 80mg capsules (A A H Pharmaceuticals Ltd)
58910	1699020	Valsartan 80mg capsules (Sigma Pharmaceuticals Plc)
38395	91976020	Valsartan 80mg tablets

For peer review only

Beta blockers

Product code	GEM code	Description
26788	4486007	PROPRANOLOL 2.5 MG ELI
23604	!6000601	PROPRANOLOL S/R
29803	4484007	PROPRANOLOL 3 MG ELI
19003	4394007	SPIRONOLACTONE/PROPRANOLOL 50 MG TAB
4021	6228007	PROPRANOLOL 20 MG TAB
26105	5680007	PROPRANOLOL paed 4 MG TAB
46493	4483007	PROPRANOLOL 15 MG SYR
32470	4480007	PROPRANOLOL 1 MG LIQ
25818	5678007	PROPRANOLOL 30 MG SUS
22634	4485007	PROPRANOLOL 10 MG SUS
27036	5679007	PROPRANOLOL POWDERS 5 MG POW
44310	!3431105	INDERAL
25052	!3121601	HALF-INDERAL LA
61573	30311020	Atenolol 25mg/5ml oral solution
8765	5317007	ATENOLOL/CHLORTHALIDONE 50 MG TAB
24677	!0394102	ATENOLOL
25037	!0394103	ATENOLOL
26807	!7046101	TENORETIC
52611	31454020	Bisoprolol 10mg/5ml oral solution
18722	!8504177	CARVEDILOL
22796	!8504114	CARVEDILOL 3.125 MG
7491	6201007	LABETALOL TAB
24520	4879007	HYDROCHLOROTHIAZIDE /METOPROLOL TARTRATE 25 MG TAB
28493	3289007	METOPROLOL FUMARATE 95 MG TAB
22151	!4529101	METOPROLOL 100MG/CHLORTHALIDONE 12.5MG
41892	!4530101	METOPROLOL 100MG/HYDROCHLOROTHIAZ.12.5MG
17876	3667007	METOPROLOL FUMARATE 190 MG TAB
41897	!1385102	CO-BETALOC
24378	!0681102	BETALOC S.A. (CALENDAR PACK)
20813	!0681101	BETALOC S.A.
16669	2319007	LOPRESOR SR 200 MG TAB
27086	6704007	NADOLOL 80MG/BENDROFLUAZIDE 5MG MG TAB
8113	59925020	Acebutolol 200mg capsules
8023	52703020	Sectral 400mg tablets (Sanofi)
12296	52701020	Sectral 100mg capsules (Sanofi)
8172	59924020	Acebutolol 100mg capsules
8555	52702020	Sectral 200mg capsules (Sanofi)
7620	59926020	Acebutolol 400mg tablets
45309	71703020	Acebutolol 400mg tablets (A A H Pharmaceuticals Ltd)
14126	59935020	Acebutolol 200mg / Hydrochlorothiazide 12.5mg tablets
8189	51499020	Secadrex 200mg/12.5mg tablets (Sanofi)
7429	51862020	Tenormin 5mg/10ml solution for injection ampoules (AstraZeneca UK Ltd)
197	60300020	Atenolol 5mg/10ml solution for injection ampoules
34754	51439020	Atenolol 100mg tablets (Sandoz Ltd)
53826	1006020	Atenolol 25mg tablets (Boston Healthcare Ltd)
4983	68844020	Atenolol with amiloride and hydrochlorothiazide capsules
44858	56835020	Atenolol 25mg tablets (Actavis UK Ltd)
34265	51438020	Atenolol 50mg tablets (Sandoz Ltd)
51998	1011020	Atenolol 25mg tablets (Co-Pharma Ltd)
34976	59302020	Atenolol 25mg tablets (Tillomed Laboratories Ltd)

For peer review only

53204	973020	Atenolol 50mg tablets (Alliance Healthcare (Distribution) Ltd)
33850	56836020	Atenolol 50mg tablets (Actavis UK Ltd)
19172	57790020	Atenolol 25mg tablets (IVAX Pharmaceuticals UK Ltd)
13394	51861020	Tenormin 25mg/5ml syrup (AstraZeneca UK Ltd)
21133	86681020	Atenamin 50mg Tablet (OPD Pharm)
30636	72687020	Vasaten 50mg Tablet (Shire Pharmaceuticals Ltd)
46908	56095020	Atenolol 100mg tablets (Kent Pharmaceuticals Ltd)
33085	51023020	Atenolol 100mg tablets (A A H Pharmaceuticals Ltd)
59982	1012020	Atenolol 25mg tablets (Accord Healthcare Ltd)
34882	52909020	Atenolol 50mg Tablet (Berk Pharmaceuticals Ltd)
34365	53708020	Atenolol 50mg tablets (Teva UK Ltd)
31536	56093020	Atenolol 25mg tablets (Kent Pharmaceuticals Ltd)
59695	980020	Atenolol 50mg tablets (Boston Healthcare Ltd)
53414	985020	Atenolol 50mg tablets (Accord Healthcare Ltd)
52500	70253020	Atenolol 50mg tablets (Almus Pharmaceuticals Ltd)
34585	51440020	Atenolol 25mg tablets (Sandoz Ltd)
20502	72697020	Atenix 100 tablets (Ashbourne Pharmaceuticals Ltd)
29398	86682020	Atenamin 100mg Tablet (OPD Pharm)
2587	51860020	Tenormin 100mg tablets (AstraZeneca UK Ltd)
5	60153020	Atenolol 50mg tablets
20728	86680020	Atenamin 25mg Tablet (OPD Pharm)
54542	1008020	Atenolol 25mg tablets (Zanza Laboratories Ltd)
33092	51022020	Atenolol 50mg tablets (A A H Pharmaceuticals Ltd)
19191	53707020	Atenolol 100mg tablets (Teva UK Ltd)
53802	1007020	Atenolol 25mg tablets (Sigma Pharmaceuticals Plc)
33184	52914020	Atenolol 100mg tablets (Wockhardt UK Ltd)
29368	53709020	Atenolol 25mg tablets (Teva UK Ltd)
57817	74385020	Atenolol 50mg tablets (Zentiva)
19182	55394020	Atenolol 50mg tablets (IVAX Pharmaceuticals UK Ltd)
17322	72698020	Atenix 25 tablets (Ashbourne Pharmaceuticals Ltd)
3526	69296020	Amiloride with atenolol with hydrochlorothiazide capsules
55778	983020	Atenolol 50mg tablets (Phoenix Healthcare Distribution Ltd)
54752	984020	Atenolol 50mg tablets (Co-Pharma Ltd)
34695	56094020	Atenolol 50mg tablets (Kent Pharmaceuticals Ltd)
10191	72696020	Atenix 50 tablets (Ashbourne Pharmaceuticals Ltd)
28177	68841020	Hydrochlorothiazide with atenolol and amiloride Capsule
26211	68815020	Antipressan 25mg tablets (Teva UK Ltd)
24	60152020	Atenolol 100mg tablets
47870	70250020	Atenolol 25mg tablets (Almus Pharmaceuticals Ltd)
24195	68814020	Antipressan 100mg tablets (Teva UK Ltd)
49953	1009020	Atenolol 25mg tablets (Bristol Laboratories Ltd)
24191	68813020	Antipressan 50mg tablets (Teva UK Ltd)
52310	39793020	Atenolol 25mg tablets (Crescent Pharma Ltd)
34492	60231020	Atenolol 25mg tablets (Generics (UK) Ltd)
2590	72526020	Tenormin 25mg tablets (AstraZeneca UK Ltd)
26	67920020	Atenolol 25mg tablets
36261	59303020	Atenolol 50mg tablets (Tillomed Laboratories Ltd)
6066	67919020	Atenolol 25mg/5ml oral solution sugar free
34575	52915020	Atenolol 25mg tablets (Wockhardt UK Ltd)
18950	72693020	Totamol 25mg Tablet (C P Pharmaceuticals Ltd)
33657	51024020	Atenolol 25mg tablets (A A H Pharmaceuticals Ltd)
34443	52913020	Atenolol 50mg tablets (Wockhardt UK Ltd)
31934	55395020	Atenolol 100mg tablets (IVAX Pharmaceuticals UK Ltd)
33650	60232020	Atenolol 50mg tablets (Generics (UK) Ltd)

For peer review only

50702	998020	Atenolol 25mg tablets (Alliance Healthcare (Distribution) Ltd)
15176	72691020	Totamol 50mg Tablet (C P Pharmaceuticals Ltd)
33079	60233020	Atenolol 100mg tablets (Generics (UK) Ltd)
2432	54818020	Tenormin LS 50mg tablets (AstraZeneca UK Ltd)
15730	72692020	Totamol 100mg Tablet (C P Pharmaceuticals Ltd)
51643	1013020	Atenolol 25mg/5ml oral solution sugar free (Alliance Healthcare (Distribution) Ltd)
46931	56837020	Atenolol 100mg tablets (Actavis UK Ltd)
56445	77037020	Atenolol 25mg/5ml oral solution sugar free (A A H Pharmaceuticals Ltd)
53215	982020	Atenolol 50mg tablets (Bristol Laboratories Ltd)
7543	56162020	Kalten capsules (M & A Pharmachem Ltd)
9178	83986020	Atenolol 25mg / Bendroflumethiazide 1.25mg capsules
18743	83989020	Tenben 25mg/1.25mg capsules (Galen Ltd)
1288	51857020	Tenoret 50mg/12.5mg tablets (AstraZeneca UK Ltd)
5721	73883020	Co-tenidone 100mg/25mg tablets
34449	60352020	Co-tenidone 50mg/12.5mg tablets (Generics (UK) Ltd)
46952	56911020	Co-tenidone 100mg/25mg tablets (Actavis UK Ltd)
31470	57164020	Tenchlor 50mg/12.5mg tablets (Teva UK Ltd)
24280	80150020	Totaretic 100mg+25mg Tablet (C P Pharmaceuticals Ltd)
31708	56910020	Co-tenidone 50mg/12.5mg tablets (Actavis UK Ltd)
1124	54815020	Tenoretic 100mg/25mg tablets (AstraZeneca UK Ltd)
32094	53521020	Co-tenidone 50mg/12.5mg tablets (A A H Pharmaceuticals Ltd)
26741	80149020	Totaretic 50mg+12.5mg Tablet (C P Pharmaceuticals Ltd)
26248	57165020	Tenchlor 100mg/25mg tablets (Teva UK Ltd)
34034	55247020	Co-tenidone 50mg/12.5mg tablets (IVAX Pharmaceuticals UK Ltd)
34825	54653020	Co-tenidone 50mg/12.5mg tablets (Teva UK Ltd)
41572	54654020	Co-tenidone 100mg/25mg tablets (Teva UK Ltd)
34899	53522020	Co-tenidone 100mg/25mg tablets (A A H Pharmaceuticals Ltd)
13526	63763020	Atenix Co 100 tablets (Ashbourne Pharmaceuticals Ltd)
37725	60353020	Co-tenidone 100mg/25mg tablets (Generics (UK) Ltd)
34012	55246020	Co-tenidone 100mg/25mg tablets (IVAX Pharmaceuticals UK Ltd)
9783	73882020	Co-tenidone 50mg/12.5mg tablets
21873	63762020	Atenix Co 50 tablets (Ashbourne Pharmaceuticals Ltd)
4542	68847020	Atenolol 50mg / Nifedipine 20mg modified-release capsules
52728	1077020	Beta-Adalat modified-release capsules (Lexon (UK) Ltd)
61719	1072020	Beta-Adalat modified-release capsules (Waymade Healthcare Plc)
8642	68652020	Tenif 50mg/20mg modified-release capsules (AstraZeneca UK Ltd)
1684	68629020	Beta-Adalat modified-release capsules (Bayer Plc)
43251	75323020	Bisoprolol 1.25mg tablets (Generics (UK) Ltd)
58763	47116020	Bisoprolol 2.5mg tablets (Waymade Healthcare Plc)
33839	62689020	Bisoprolol 10mg tablets (Actavis UK Ltd)
17615	83190020	Cardicor 5mg tablets (Merck Serono Ltd)
33909	90617020	Congescor 1.25mg tablets (Tillomed Laboratories Ltd)
19178	62996020	Bisoprolol 10mg tablets (Ranbaxy (UK) Ltd)
56240	40506020	Bisoprolol 3.75mg tablets (Sandoz Ltd)
472	68438020	Bisoprolol 5mg tablets
55791	40507020	Bisoprolol 3.75mg tablets (Actavis UK Ltd)
58498	15612021	Bisoprolol 2.5mg tablets (Medreich Plc)
52686	31458020	Bisoprolol 2.5mg/5ml oral solution
822	91125020	Bisoprolol 1.5mg/5ml oral suspension
52548	78457020	Bisoprolol 1.25mg tablets (Almus Pharmaceuticals Ltd)
3588	68353020	Monocor 5mg tablets (Wyeth Pharmaceuticals)
594	83178020	Bisoprolol 2.5mg tablets

For peer review only - http://bmjopen.bmj.com/site/about/guidelines.xhtml

53664	40505020	Bisoprolol 2.5mg tablets (Sandoz Ltd)
61651	1149020	Bisoprolol 7.5mg tablets (Almus Pharmaceuticals Ltd)
10892	68369020	Emcor 10mg tablets (Merck Serono Ltd)
38991	75624020	Bisoprolol 7.5mg tablets (A A H Pharmaceuticals Ltd)
24083	62681020	Bisoprolol 5mg tablets (Teva UK Ltd)
50514	1122020	Bisoprolol 2.5mg tablets (Chanelle Medical UK Ltd)
34821	62677020	Bisoprolol 10mg tablets (Generics (UK) Ltd)
47041	75328020	Bisoprolol 2.5mg tablets (Generics (UK) Ltd)
18185	83191020	Cardicor 7.5mg tablets (Merck Serono Ltd)
53885	74139020	Bisoprolol 1.25mg tablets (A A H Pharmaceuticals Ltd)
59148	41897020	Bisoprolol 2.5mg tablets (Zentiva)
58974	1116020	Bisoprolol 2.5mg tablets (Alliance Healthcare (Distribution) Ltd)
19200	63135020	Bisoprolol 5mg tablets (IVAX Pharmaceuticals UK Ltd)
61340	38485020	Bisoprolol 5mg tablets (DE Pharmaceuticals)
55929	45144020	Bisoprolol 5mg tablets (Accord Healthcare Ltd)
59495	43811020	Bisoprolol 1.25mg tablets (Teva UK Ltd)
61564	47117020	Bisoprolol 3.75mg tablets (Waymade Healthcare Plc)
37118	73449020	Bisoprolol 2.5mg tablets (A A H Pharmaceuticals Ltd)
7553	90711020	Bisoprolol 5mg/5ml oral suspension
39846	84559020	Vivacor 5mg tablets (Lexon (UK) Ltd)
50224	41307020	Congescor 2.5mg tablets (Teva UK Ltd)
55298	1097020	Bisoprolol 10mg tablets (Sigma Pharmaceuticals Plc)
57176	45145020	Bisoprolol 10mg tablets (Accord Healthcare Ltd)
57934	67815020	Bisoprolol 5mg tablets (Sandoz Ltd)
32552	90619020	Congescor 2.5mg tablets (Tillomed Laboratories Ltd)
599	68440020	Bisoprolol 1.25mg tablets
57626	19127020	Bisoprolol 1.25mg/5ml oral solution
1290	68439020	Bisoprolol 10mg tablets
59969	1090020	Bisoprolol 5mg tablets (Almus Pharmaceuticals Ltd)
59037	62942020	Bisoprolol 5mg tablets (A A H Pharmaceuticals Ltd)
43564	62726020	Bisoprolol 5mg Tablet (PLIVA Pharma Ltd)
4771	68375020	Emcor LS 5mg tablets (Merck Serono Ltd)
54479	1103020	Bisoprolol 1.25mg tablets (Alliance Healthcare (Distribution) Ltd)
21905	86502020	Bipranix 10mg tablets (Ashbourne Pharmaceuticals Ltd)
14058	83182020	Cardicor 1.25mg tablets (Merck Serono Ltd)
58982	38487020	Bisoprolol 10mg tablets (Medreich Plc)
58109	19125020	Bisoprolol 1.25mg/5ml oral suspension
7091	83179020	Bisoprolol 3.75mg tablets
52635	1082020	Bisoprolol 5mg tablets (Alliance Healthcare (Distribution) Ltd)
39646	96562020	Bisoprolol 0.625mg/5ml oral solution
41591	62682020	Bisoprolol 10mg tablets (Teva UK Ltd)
44000	98730020	Bisoprolol 2.5mg/5ml oral suspension
14030	83183020	Cardicor 2.5mg tablets (Merck Serono Ltd)
19858	83192020	Cardicor 10mg tablets (Merck Serono Ltd)
50403	73571020	Bisoprolol 1.25mg Tablet (Teva UK Ltd)
5713	83180020	Bisoprolol 7.5mg tablets
60761	15611021	Bisoprolol 1.25mg tablets (Medreich Plc)
60896	38486020	Bisoprolol 5mg tablets (Medreich Plc)
61115	19119020	Bisoprolol 5mg/5ml oral solution
19853	83184020	Cardicor 3.75mg tablets (Merck Serono Ltd)
57023	1125020	Bisoprolol 2.5mg tablets (Almus Pharmaceuticals Ltd)
50300	41306020	Congescor 1.25mg tablets (Teva UK Ltd)
51528	40504020	Bisoprolol 1.25mg tablets (Actavis UK Ltd)
53334	62943020	Bisoprolol 10mg tablets (A A H Pharmaceuticals Ltd)

For peer review only

56486	1094020	Monocor 10mg tablets (Dowelhurst Ltd)
21966	86500020	Bipranix 5mg tablets (Ashbourne Pharmaceuticals Ltd)
58973	63965020	Bisoprolol 10mg tablets (Niche Generics Ltd)
37837	73574020	Bisoprolol 2.5mg Tablet (Teva UK Ltd)
53916	78463020	Bisoprolol 2.5mg tablets (Almus Pharmaceuticals Ltd)
32630	77372020	Vivacor 10mg tablets (Lexon (UK) Ltd)
34963	62688020	Bisoprolol 5mg tablets (Actavis UK Ltd)
57578	1123020	Cardicor 2.5mg tablets (Necessity Supplies Ltd)
60502	38490020	Bisoprolol 3.75mg tablets (DE Pharmaceuticals)
56459	45146020	Bisoprolol 2.5mg tablets (Accord Healthcare Ltd)
32114	62676020	Bisoprolol 5mg tablets (Generics (UK) Ltd)
56768	77972020	Bisoprolol 2.5mg tablets (Niche Generics Ltd)
5968	68354020	Monocor 10mg tablets (Wyeth Pharmaceuticals)
58455	40508020	Bisoprolol 7.5mg tablets (Sandoz Ltd)
58511	40503020	Bisoprolol 1.25mg tablets (Sandoz Ltd)
17462	75023020	Bisoprolol 10mg / Hydrochlorothiazide 6.25mg tablets
17149	75020020	Monozide 10 tablets (Wyeth Pharmaceuticals)
34741	67913020	Carvedilol 3.125mg tablets (IVAX Pharmaceuticals UK Ltd)
47107	567021	Carvedilol 5mg/5ml oral suspension
34501	67708020	Carvedilol 12.5mg tablets (Actavis UK Ltd)
49142	31922020	Carvedilol 3.125mg/5ml oral suspension
18414	72241020	Eucardic 12.5mg tablets (Roche Products Ltd)
46936	67784020	Carvedilol 3.125mg tablets (A A H Pharmaceuticals Ltd)
46935	67701020	Carvedilol 3.125mg tablets (Actavis UK Ltd)
4410	83758020	Carvedilol 6.25mg tablets
54106	35298020	Carvedilol 1.5mg/5ml oral suspension
14146	83760020	Eucardic 6.25mg tablets (Roche Products Ltd)
14117	72243020	Eucardic 3.125mg tablets (Roche Products Ltd)
19202	67720020	Carvedilol 6.25mg tablets (Teva UK Ltd)
19437	72242020	Eucardic 25mg tablets (Roche Products Ltd)
34740	67705020	Carvedilol 6.25mg tablets (Actavis UK Ltd)
59549	19333020	Carvedilol 5mg/5ml oral suspension
61663	67717020	Carvedilol 3.125mg tablets (Teva UK Ltd)
33374	69739020	Carvedilol 12.5mg tablets (Genus Pharmaceuticals Ltd)
7049	72237020	Carvedilol 25mg tablets
817	72238020	Carvedilol 3.125mg tablets
2629	72236020	Carvedilol 12.5mg tablets
8262	68837020	Celiprolol 200mg tablets
16776	68966020	Celectol 400mg Tablet (Pantheon Healthcare Ltd)
35940	91237020	Celectol 400mg tablets (Zentiva)
56485	1155020	Celectol 200mg tablets (Waymade Healthcare Plc)
41740	63783020	Celiprolol 200mg tablets (Teva UK Ltd)
57573	1153020	Celectol 200mg tablets (Dowelhurst Ltd)
4265	68965020	Celectol 200mg Tablet (Pantheon Healthcare Ltd)
7974	68838020	Celiprolol 400mg tablets
42795	61054020	Celiprolol 200mg tablets (Generics (UK) Ltd)
35054	91235020	Celectol 200mg tablets (Zentiva)
30541	57093020	Esmolol HCl 250mg/ml concentrate solution for infusion
26922	56705020	Brevibloc Premixed 100mg/10ml solution for injection vials (Baxter Healthcare Ltd)
32135	56706020	Brevibloc Concentrate 2.5g/10ml solution for infusion ampoules (Baxter Healthcare Ltd)
39819	89270020	Esmolol 2.5g/250ml infusion bags
7066	68976020	Metoprolol 100mg / Hydrochlorothiazide 12.5mg tablets

For peer review only

10627	48722020	Co-Betaloc tablets (Pfizer Ltd)
59222	43810020	Labetalol 100mg/20ml solution for injection ampoules (Focus Pharmaceuticals Ltd)
19068	63903020	Labetalol 50mg/10ml solution for injection pre-filled syringes
38370	90779020	Labetalol 100mg/20ml solution for injection ampoules
19998	51987020	Trandate 100mg/20ml solution for injection ampoules (Focus Pharmaceuticals Ltd)
35778	58483020	Labrocol 100mg Tablet (Lagap)
40240	49924020	Labetalol 400mg tablets (A A H Pharmaceuticals Ltd)
34177	49922020	Labetalol 100mg tablets (A A H Pharmaceuticals Ltd)
1295	68333020	Labetalol 400mg tablets
22793	58484020	Labrocol 200mg Tablet (Lagap)
1597	63899020	Labetalol 100mg tablets
41827	60515020	Labetalol 100mg tablets (Generics (UK) Ltd)
47674	49918020	Labetalol 200mg Tablet (C P Pharmaceuticals Ltd)
4725	68332020	Labetalol 50mg tablets
45250	66745020	Labetalol 400mg tablets (Sandoz Ltd)
44083	57013020	Labetalol 200mg tablets (Actavis UK Ltd)
47673	54575020	Labetalol 400mg Tablet (Approved Prescription Services Ltd)
8707	54881020	Trandate 200mg tablets (Focus Pharmaceuticals Ltd)
2775	63900020	Labetalol 200mg tablets
34171	49917020	Labetalol 100mg Tablet (C P Pharmaceuticals Ltd)
8807	52545020	Trandate 400mg tablets (Focus Pharmaceuticals Ltd)
30770	49923020	Labetalol 200mg tablets (A A H Pharmaceuticals Ltd)
9016	54880020	Trandate 100mg tablets (Focus Pharmaceuticals Ltd)
34188	51424020	Labetalol 200mg Tablet (Celltech Pharma Europe Ltd)
16645	58485020	Labrocol 400mg Tablet (Lagap)
9273	54879020	Trandate 50mg tablets (Focus Pharmaceuticals Ltd)
29998	69479020	Metoros 190mg Tablet (Novartis Pharmaceuticals UK Ltd)
27719	69482020	Metoros ls 95mg Tablet (Geigy Pharmaceuticals)
24461	53655020	Betaloc I.V. 5mg/5ml solution for injection ampoules (AstraZeneca UK Ltd)
14502	52381020	Metoprolol 5mg/5ml solution for injection ampoules
33659	68979020	Hydrochlorothiazide with metoprolol tartrate 25mg with 200mg Modified-release tablet
40167	54126020	Metoprolol 100mg tablets (IVAX Pharmaceuticals UK Ltd)
34509	60551020	Metoprolol 100mg tablets (Generics (UK) Ltd)
34094	50072020	Metoprolol 50mg tablets (A A H Pharmaceuticals Ltd)
3474	53660020	Betaloc-SA 200mg tablets (AstraZeneca UK Ltd)
20082	50243020	Lopresor SR 200mg tablets (Recordati Pharmaceuticals Ltd)
8068	64670020	Metoprolol 200mg modified-release tablets
29762	63850020	Mepranix 50mg Tablet (Ashbourne Pharmaceuticals Ltd)
57240	20539020	Metoprolol 50mg/5ml oral suspension (Drug Tariff Special Order)
11793	67160020	Metoprolol 50mg/5ml oral suspension
10429	50246020	Lopresor 50mg Tablet (Novartis Pharmaceuticals UK Ltd)
8071	48396020	Betaloc 50mg tablets (AstraZeneca UK Ltd)
34925	65979020	Metoprolol 50mg tablets (Sandoz Ltd)
32836	60550020	Metoprolol 50mg tablets (Generics (UK) Ltd)
739	64666020	Metoprolol 50mg tablets
3344	48397020	Betaloc 100mg tablets (AstraZeneca UK Ltd)
34430	57037020	Metoprolol 50mg tablets (Actavis UK Ltd)
47536	770021	Metoprolol tartrate 12.5mg/5ml Oral suspension
55979	20535020	Metoprolol 25mg/5ml oral suspension
46614	195021	Lopresor 50mg tablets (Recordati Pharmaceuticals Ltd)

For peer review only

34584	54125020	Metoprolol 50mg tablets (IVAX Pharmaceuticals UK Ltd)
45289	91796020	Metoprolol tartrate Oral solution
51447	20527020	Metoprolol 12.5mg/5ml oral suspension
29427	68980020	Hydrochlorothiazide with metoprolol tartrate 12.5mg with 100mg tablet
8147	50252020	Lopresoretic Tablet (Novartis Pharmaceuticals UK Ltd)
34854	57038020	Metoprolol 100mg tablets (Actavis UK Ltd)
753	64667020	Metoprolol 100mg tablets
13499	50247020	Lopresor 100mg Tablet (Novartis Pharmaceuticals UK Ltd)
34125	50073020	Metoprolol 100mg tablets (A A H Pharmaceuticals Ltd)
30400	63851020	Mepranix 100mg Tablet (Ashbourne Pharmaceuticals Ltd)
15488	68972020	Metoprolol tartrate with chlortalidone Tablet
34407	56767020	Metoprolol 50mg tablets (Teva UK Ltd)
34890	53461020	Metoprolol 50mg Tablet (Berk Pharmaceuticals Ltd)
34092	56768020	Metoprolol 100mg tablets (Teva UK Ltd)
46740	197021	Lopresor 100mg tablets (Recordati Pharmaceuticals Ltd)
18287	53671020	Co-Betaloc SA tablets (Pfizer Ltd)
20093	68975020	Metoprolol 200mg modified-release / Hydrochlorothiazide 25mg tablets
10716	54539020	Corgard 80mg tablets (Sanofi)
13415	54538020	Corgard 40mg tablets (Sanofi-Synthelabo Ltd)
11338	67627020	Bendroflumethiazide 5mg with Nadolol 40mg tablets
8935	64895020	Nadolol 40mg tablets
2499	64896020	Nadolol 80mg tablets
23134	64899020	Nadolol 40mg / Bendroflumethiazide 5mg tablets
14438	54543020	Corgaretic 80mg tablets (Sanofi-Synthelabo Ltd)
27946	64900020	Nadolol 80mg / Bendroflumethiazide 5mg tablets
5330	48767020	Corgaretic 40mg tablets (Sanofi-Synthelabo Ltd)
59961	21461021	Nebivolol 10mg tablets
7528	80460020	Nebilet 5mg tablets (A. Menarini Farmaceutica Internazionale SRL)
47300	76819020	Nebivolol 2.5mg tablets (Glenmark Generics (Europe) Ltd)
40761	97367020	Nebivolol 2.5mg tablets
751	80458020	Nebivolol 5mg tablets
44808	77127020	Nebivolol 2.5mg tablets (A A H Pharmaceuticals Ltd)
54487	44459020	Nebivolol 2.5mg tablets (Sigma Pharmaceuticals Plc)
52145	61691020	Cyclopenthiazide 0.25mg with oxprenolol 160mg modified-release tablets
2361	54886020	Trasicor 80mg Tablet (Novartis Pharmaceuticals UK Ltd)
3748	59365020	Oxprenolol 160mg Tablet
7474	54884020	Trasicor 20mg Tablet (Novartis Pharmaceuticals UK Ltd)
27357	49156020	Oxprenolol 40mg Tablet (Actavis UK Ltd)
29180	90895020	Trasicor 80mg tablets (Amdipharm Plc)
8673	73918020	Oxprenolol with cyclopenthiazide 160mg+0.25mg Modified-release tablet
35062	90891020	Trasicor 20mg tablets (Amdipharm Plc)
29230	59496020	Slow-pren 160mg Tablet (IVAX Pharmaceuticals UK Ltd)
10777	54889020	Trasicor 160mg Tablet (Novartis Pharmaceuticals UK Ltd)
3516	59360020	Oxprenolol 20mg tablets
21885	74896020	Oxyprenix SR 160mg tablets
4025	51584020	Slow-Trasicor 160mg tablets (AMCo)
24094	90893020	Trasicor 40mg tablets (Amdipharm Plc)
1334	65274020	Oxprenolol 160mg modified-release tablets
2780	59362020	Oxprenolol 80mg tablets
25644	57667020	Apsolox 80mg Tablet (Approved Prescription Services Ltd)

For peer review only

33569	49177020	Oxprenolol sr 160mg Modified-release tablet (Hillcross Pharmaceuticals Ltd)
8290	54885020	Trasicor 40mg Tablet (Novartis Pharmaceuticals UK Ltd)
1333	59361020	Oxprenolol 40mg tablets
13871	73921020	Co-prenozide 160mg/0.25mg modified-release tablets
4429	51994020	Trasidrex modified-release tablets (Mercury Pharma Group Ltd)
4588	52291020	Visken 5mg Tablet (Sovereign Medical Ltd)
25462	61503020	Clopamide 5mg with Pindolol 10mg tablets
20012	52292020	Visken 15mg Tablet (Sovereign Medical Ltd)
5284	65802020	Pindolol 5mg tablets
55853	60484020	Pindolol 15mg Tablet (Hillcross Pharmaceuticals Ltd)
14673	65803020	Pindolol 15mg tablets
35695	78143020	Visken 5mg tablets (AMCo)
32787	78135020	Visken 15mg tablets (AMCo)
9143	52285020	Viskaldix tablets (AMCo)
14057	65806020	Pindolol 10mg / Clopamide 5mg tablets
53177	92021020	Propranolol oral solution
27486	66311020	Propranolol 1mg/1ml solution for injection ampoules
10294	54252020	Inderal 1mg/1ml solution for injection ampoules (AstraZeneca UK Ltd)
5478	59376020	Propranolol 10mg/5ml oral solution sugar free
58491	924020	Propranolol 40mg tablets (Alliance Healthcare (Distribution) Ltd)
34378	50435020	Propranolol 10mg tablets (A A H Pharmaceuticals Ltd)
55416	71580020	Propranolol 40mg tablets (Almus Pharmaceuticals Ltd)
55949	20600020	Propranolol 40mg/5ml oral solution
58297	918020	Propranolol 10mg tablets (Kent Pharmaceuticals Ltd)
31776	63160020	Propranolol 40mg tablets (Generics (UK) Ltd)
45877	99275020	Beta-Prograne 160mg modified-release capsules (Teva UK Ltd)
1048	54495020	Inderal 80mg tablets (AstraZeneca UK Ltd)
34783	50422020	Propranolol 10mg tablets (Actavis UK Ltd)
48682	183020	Propranolol 50mg/5ml oral solution sugar free
3167	59374020	Propranolol 160mg tablets
35938	64558020	Propranolol 80mg modified-release capsules (A A H Pharmaceuticals Ltd)
14808	55786020	Bedranol SR 80mg capsules (Sandoz Ltd)
9185	75255020	Propranolol 80mg/5ml oral solution
34208	51777020	Propranolol SR 160mg Modified-release capsule (C P Pharmaceuticals Ltd)
29763	63927020	Propanix 160mg Tablet (Ashbourne Pharmaceuticals Ltd)
36603	50847020	Propranolol SR 160mg Modified-release capsule (Hillcross Pharmaceuticals Ltd)
45297	64022020	Propranolol 40mg tablets (IVAX Pharmaceuticals UK Ltd)
23587	66314020	Sloprolol 160mg Capsule (C P Pharmaceuticals Ltd)
34214	50424020	Propranolol 160mg tablets (Actavis UK Ltd)
31833	59468020	Angilol 80mg Tablet (DDSA Pharmaceuticals Ltd)
33602	85990020	Slo-Pro 160mg capsules (Generics (UK) Ltd)
41555	50436020	Propranolol 40mg tablets (A A H Pharmaceuticals Ltd)
25359	79243020	Rapranol SR 160mg capsules (Ranbaxy (UK) Ltd)
23131	67621020	Bendroflumethiazide 5mg with Propanolol 160mg modified-release capsules
297	59368020	Propranolol 10mg tablets
40241	56193020	Propranolol LA 160mg Capsule (Approved Prescription Services Ltd)
33644	50437020	Propranolol 80mg tablets (A A H Pharmaceuticals Ltd)

33836	57662020	Apsolol 160mg Tablet (Approved Prescription Services Ltd)
56173	955020	Half Beta-Prograne 80mg modified-release capsules (Actavis UK Ltd)
28128	54244020	Propranolol 80mg Modified-release capsule (Actavis UK Ltd)
1050	54494020	Inderal 40mg tablets (AstraZeneca UK Ltd)
33376	78494020	Probeta LA 160mg Capsule (Trinity Pharmaceuticals Ltd)
23326	71607020	Betadur cr 160mg Modified-release capsule (Monmouth Pharmaceuticals Ltd)
59597	75862020	Propranolol 160mg modified-release capsules (A A H Pharmaceuticals Ltd)
3005	49929020	Inderal LA 160mg capsules (AstraZeneca UK Ltd)
28996	87099020	Bedranol SR 160mg capsules (Sandoz Ltd)
1448	66308020	Propranolol 160mg modified-release capsules
3827	64050020	Propanix 40mg Tablet (Ashbourne Pharmaceuticals Ltd)
57063	957020	Bedranol SR 80mg capsules (Almus Pharmaceuticals Ltd)
45765	96602020	Syprol 40mg/5ml oral solution (Rosemont Pharmaceuticals Ltd)
21839	54823020	Berkolol 80mg Tablet (Berk Pharmaceuticals Ltd)
31214	63161020	Propranolol 80mg tablets (Generics (UK) Ltd)
8978	63928020	Propanix 160mg Modified-release capsule (Ashbourne Pharmaceuticals Ltd)
54623	99279020	Beta-Prograne 160mg modified-release capsules (Actavis UK Ltd)
27700	50423020	Propranolol 40mg tablets (Actavis UK Ltd)
45494	71577020	Propranolol 10mg tablets (Almus Pharmaceuticals Ltd)
32162	59465020	Propranolol 80mg Modified-release capsule (Lagap)
45343	51778020	Propranolol SR 80mg Modified-release capsule (C P Pharmaceuticals Ltd)
3087	75253020	Propranolol 40mg/5ml oral solution sugar free
54297	34198020	Propranolol 50mg/5ml oral solution
769	66307020	Propranolol 80mg modified-release capsules
707	59369020	Propranolol 40mg tablets
47543	627021	Half Beta-Prograne 80mg modified-release capsules (Actavis UK Ltd)
55849	63164020	Propranolol 160mg tablets (Generics (UK) Ltd)
17783	57624020	Spiroprop Tablet (Pharmacia Ltd)
21838	64051020	Propanix 80mg Tablet (Ashbourne Pharmaceuticals Ltd)
34949	54245020	Propranolol 160mg Modified-release capsule (Actavis UK Ltd)
59415	20140021	Propranolol 40mg tablets (Accord Healthcare Ltd)
17082	79593020	Syprol 5mg/5ml oral solution (Rosemont Pharmaceuticals Ltd)
26895	79594020	Syprol 10mg/5ml oral solution (Rosemont Pharmaceuticals Ltd)
15619	57243020	Half-betadur cr 80mg Capsule (Monmouth Pharmaceuticals Ltd)
25367	84837020	Rapranol SR 80mg capsules (Ranbaxy (UK) Ltd)
42152	79595020	Syprol 50mg/5ml oral solution (Rosemont Pharmaceuticals Ltd)
46363	99271020	Half Beta-Prograne 80mg modified-release capsules (Teva UK Ltd)
58407	53353020	Propranolol 80mg tablets (Teva UK Ltd)
39233	68806020	Propranolol 80mg modified-release capsules (Teva UK Ltd)
38433	55581020	Propranolol 50mg/5ml Oral solution (Rosemont Pharmaceuticals Ltd)
22208	84849020	Half propanix la 80mg Modified-release capsule (Ashbourne Pharmaceuticals Ltd)
60565	70592020	Propranolol 40mg tablets (Ranbaxy (UK) Ltd)
34884	66703020	Propranolol 160mg Modified-release capsule (Sandoz Ltd)
47907	538021	Bedranol SR 160mg capsules (Almus Pharmaceuticals Ltd)
36576	63159020	Propranolol 10mg tablets (Generics (UK) Ltd)
20468	72042020	Half Beta-Prograne 80mg modified-release capsules (Tillomed

For peer review only

		Laboratories Ltd)
21866	54822020	Berkolol 40mg Tablet (Berk Pharmaceuticals Ltd)
8331	54498020	Inderal 160mg Tablet (AstraZeneca UK Ltd)
34867	56034020	Propranolol 80mg Capsule (IVAX Pharmaceuticals UK Ltd)
57567	34143020	Propranolol 10mg/5ml oral suspension
34804	53351020	Propranolol 10mg tablets (Teva UK Ltd)
2414	54493020	Inderal 10mg tablets (AstraZeneca UK Ltd)
26229	56515020	Beta-Prograne 160mg modified-release capsules (Tillomed Laboratories Ltd)
220	59375020	Propranolol 5mg/5ml oral solution
940	59370020	Propranolol 80mg tablets
14552	64049020	Propanix 10mg Tablet (Ashbourne Pharmaceuticals Ltd)
27964	57658020	Apsolol 40mg Tablet (Approved Prescription Services Ltd)
26228	85670020	Propanix LA 160mg Modified-release capsule (Ashbourne Pharmaceuticals Ltd)
28048	59466020	Angilol 10mg Tablet (DDSA Pharmaceuticals Ltd)
11711	75254020	Propranolol 50mg/5ml oral solution
61727	20139021	Propranolol 10mg tablets (Accord Healthcare Ltd)
22912	67620020	Bendroflumethiazide 2.5mg with Propanolol 80mg capsules
52777	925020	Propranolol 40mg tablets (Kent Pharmaceuticals Ltd)
26255	78284020	Lopranol la 160mg Capsule (Opus Pharmaceuticals Ltd)
60934	948020	Propranolol 80mg modified-release capsules (Kent Pharmaceuticals Ltd)
34868	53352020	Propranolol 40mg tablets (Teva UK Ltd)
47833	536021	Bedranol SR 80mg capsules (Almus Pharmaceuticals Ltd)
56764	47096020	Propranolol 40mg tablets (Waymade Healthcare Plc)
57342	928020	Propranolol 40mg tablets (Phoenix Healthcare Distribution Ltd)
49863	182020	Propranolol 5mg/5ml oral solution sugar free
34945	59464020	Propranolol 160mg Modified-release capsule (Lagap)
52136	69495020	Bedranol sr 160mg Capsule (Lagap)
52609	942020	Inderal LA 160mg capsules (Sigma Pharmaceuticals Plc)
55228	927020	Propranolol 40mg tablets (Boston Healthcare Ltd)
34185	56194020	Propranolol LA 80mg Modified-release capsule (Approved Prescription Services Ltd)
1006	53253020	Half Inderal LA 80mg capsules (AstraZeneca UK Ltd)
24218	54826020	Berkolol 160mg Tablet (Berk Pharmaceuticals Ltd)
12495	54821020	Berkolol 10mg Tablet (Berk Pharmaceuticals Ltd)
43525	64019020	Propranolol 10mg tablets (IVAX Pharmaceuticals UK Ltd)
28788	85953020	Half propatard la 80mg Modified-release capsule (Galen Ltd)
12054	69745020	Propranolol 80mg / Bendroflumethiazide 2.5mg capsules
4796	54256020	Inderetic 80mg/2.5mg capsules (AstraZeneca UK Ltd)
8369	54259020	Inderex 160mg/5mg modified-release capsules (AstraZeneca UK Ltd)
8987	69746020	Propranolol 160mg modified-release / Bendroflumethiazide 5mg capsules
27727	66690020	Sotalol 2mg/ml injection
33578	79217020	Sotacor 40mg/4ml solution for injection ampoules (Bristol-Myers Squibb Pharmaceuticals Ltd)
38498	95260020	Sotalol 40mg/4ml solution for injection ampoules
17679	66689020	Sotalol 10mg/ml injection
24635	66701020	Sotacor 10mg/ml Injection (Bristol-Myers Squibb Pharmaceuticals Ltd)
1572	66682020	Sotalol 80mg tablets
3691	66694020	Sotalol 160mg with hydrochlorothiazide 25mg tablet

4004	51641020	Sotacor 80mg tablets (Bristol-Myers Squibb Pharmaceuticals Ltd)
51492	20827020	Sotalol 25mg/5ml oral solution
39423	58956020	Sotalol 80mg tablets (A A H Pharmaceuticals Ltd)
35710	91253020	Sotalol 25mg/5ml oral suspension
34640	59307020	Sotalol 40mg Tablet (Tillomed Laboratories Ltd)
34690	61147020	Sotalol 80mg tablets (Sandoz Ltd)
5858	54829020	Beta-Cardone 40mg tablets (Focus Pharmaceuticals Ltd)
34600	65314020	Sotalol 40mg tablets (Teva UK Ltd)
13487	54831020	Beta-Cardone 200mg tablets (Focus Pharmaceuticals Ltd)
6751	54830020	Beta-Cardone 80mg tablets (Focus Pharmaceuticals Ltd)
8061	66693020	Sotalol 80mg with hydrochlorothiazide 12.5mg tablet
15042	66704020	Tolerzide Tablet (Bristol-Myers Squibb Pharmaceuticals Ltd)
34371	63653020	Sotalol 40mg tablets (A A H Pharmaceuticals Ltd)
786	66681020	Sotalol 40mg tablets
9292	66683020	Sotalol 160mg tablets
13051	66686020	Sotalol 200mg tablets
43549	65838020	Sotalol 40mg tablets (IVAX Pharmaceuticals UK Ltd)
12456	51646020	Sotazide Tablet (Bristol-Myers Squibb Pharmaceuticals Ltd)
11380	51642020	Sotacor 160mg tablets (Bristol-Myers Squibb Pharmaceuticals Ltd)
34520	56537020	Sotalol 80mg tablets (Generics (UK) Ltd)
21182	69280020	Hydrochlorothiazide with timolol and amiloride 25mg with 10mg with 2.5mg Tablet
30519	69277020	Amiloride with timolol with hydrochlorothiazide tablets
25730	69274020	Timolol maleate with amiloride and hydrochlorothiazide Tablet
7852	54409020	Blocadren 10mg Tablet (Merck Sharp & Dohme Ltd)
7853	69193020	Timolol 10mg tablets
12517	67082020	Timolol maleate with bendroflumethiazide 20mg + 5mg Tablet
21025	67090020	Prestim forte Tablet (LEO Pharma)
12037	48400020	Betim 10mg Tablet (ICN Pharmaceuticals France S.A.)
19142	67631020	Bendroflumethiazide 2.5mg with Timolol maleate 10mg tablets
29610	88268020	Betim 10mg tablets (Meda Pharmaceuticals Ltd)
12651	67081020	Timolol 10mg / Bendroflumethiazide 2.5mg tablets
8623	52834020	Prestim Tablet (ICN Pharmaceuticals France S.A.)
25363	88272020	Prestim tablets (Meda Pharmaceuticals Ltd)
4605	54433020	Moducren tablets (Merck Sharp & Dohme Ltd)
12497	5741007	OXPRENOLOL 10 MG TAB
13309	4319007	TRASICOR 2 MG INJ
25764	!5661101	PINDOLOL 10MG/CLOPAMIDE 5MG
3041	3534007	SOTALOL HCl 40 MG INJ
12119	4397007	SOTALOL HCl S/R 80 MG TAB
23598	!6704106	SOTALOL HYDROCHLORIDE S/R
26290	3229007	SOTACOR 40 MG INJ
48745	37067020	Timolol 10mg / Amiloride 2.5mg / Hydrochlorothiazide 25mg tablets
8788	3217007	TIMOLOL 10MG/BENDROFLUAZIDE 2.5MG TAB

Calcium channel blockers

Product code	GEM code	Description
60744	22980021	Perindopril erbumine 8mg / Amlodipine 5mg tablets
52440	41782020	Amlodipine 10mg/5ml oral solution
9094	3901007	DILTIAZEM HCl SR 300 MG CAP
30491	!1893701	DILTIAZEM HYDROCHLORIDE
15659	5075007	DILTIAZEM HCL S/R 180 CAP
8024	3142007	DILTIAZEM HCl XL 300 MG CAP
9211	3072007	ADIZEM-XL 180 MG CAP
19015	5949007	ADIZEM CONTINUS 120 MG TAB
25026	!4904103	NIFEDIPINE RETARD
25044	!4904104	NIFEDIPINE RETARD
7823	6750007	NIFEDIPINE TAB 5 mg
25055	!4904102	NIFEDIPINE
25027	!0062301	ADALAT RETARD 10
25054	!0061102	ADALAT
27910	!0062101	ADALAT 5
23458	!7642107	VERAPAMIL SR
23730	5613007	VERAPAMIL 100 MG TAB
18631	!8504107	VERAPAMIL HCL MR
10897	3117007	VERAPAMIL S/F 40 MG/5ML SOL
21665	!1483101	CORDILOX
18690	4427007	SECURON (CALENDAR PACK) 120 MG TAB
3061	57178020	Diltiazem 12hr 180mg modified-release capsules
1995	57177020	Diltiazem 12hr 120mg modified-release capsules
5326	75621020	Diltiazem 24hr 300mg modified-release capsules
9708	75520020	Diltiazem 24hr 120mg modified-release capsules
4923	75521020	Diltiazem 24hr 180mg modified-release capsules
8558	75162020	Adizem xl 120mg Capsule (Napp Pharmaceuticals Ltd)
5194	74598020	Dilzem sr 120mg Capsule (Elan Pharma)
42731	68577020	Diltiazem sr 120mg Capsule (Hillcross Pharmaceuticals Ltd)
42804	65086020	Diltiazem HCl 180mg Capsule (PLIVA Pharma Ltd)
36583	81080020	Zemret xl 180mg Capsule (Neo Laboratories Ltd)
9240	75163020	Adizem xl 180mg Capsule (Napp Pharmaceuticals Ltd)
7562	58764020	Cardene 30mg capsules (Astellas Pharma Ltd)
8201	65032020	Nicardipine 30mg capsules
2926	65031020	Nicardipine 20mg capsules
45292	64571020	Nicardipine 30mg capsules (A A H Pharmaceuticals Ltd)
11943	58763020	Cardene 20mg capsules (Astellas Pharma Ltd)
21872	70717020	Angiopine 5mg Capsule (Ashbourne Pharmaceuticals Ltd)
34522	49116020	Nifedipine 5mg capsules (A A H Pharmaceuticals Ltd)
662	52564020	Adalat 5mg capsules (Bayer Plc)
452	65052020	Nifedipine 10mg capsules
34975	54545020	Nifedipine 5mg capsules (Teva UK Ltd)
42912	54546020	Nifedipine 10mg capsules (Teva UK Ltd)
34607	54160020	Nifedipine 5mg capsules (IVAX Pharmaceuticals UK Ltd)
22142	70808020	Calcilat 10mg Capsule (Eastern Pharmaceuticals Ltd)
43511	49117020	Nifedipine 10mg capsules (A A H Pharmaceuticals Ltd)
269	65053020	Nifedipine 5mg capsules
40074	65054020	Nifedipine 20mg Capsule
55455	1943020	Nifedipine 10mg capsules (Co-Pharma Ltd)
15117	68850020	Nifedipine with atenolol 20mg + 50mg Capsule
20878	70715020	Angiopine 10 capsules (Ashbourne Pharmaceuticals Ltd)

For peer review only

34247	49104020	Nifedipine 10mg Capsule (Berk Pharmaceuticals Ltd)
26265	79357020	Calanif 5mg Capsule (Berk Pharmaceuticals Ltd)
43515	57071020	Nifedipine 10mg capsules (Actavis UK Ltd)
46445	54159020	Nifedipine 10mg capsules (IVAX Pharmaceuticals UK Ltd)
22019	79356020	Calanif 10mg Capsule (Berk Pharmaceuticals Ltd)
2521	52565020	Adalat 10mg capsules (Bayer Plc)
2663	72565020	Diltiazem 240mg modified-release capsules
18606	81472020	Diltiazem and hydrochlorothiazide 150mg+12.5mg modified-release capsules
2453	68603020	Diltiazem 60mg modified-release capsules
11567	81446020	Ramipril 5mg with felodipine 5mg modified-release tablet
11965	81445020	Ramipril 2.5mg with felodipine 2.5mg modified-release tablet
8759	56680020	Verapamil hcl 120mg modified release tablets
4542	68847020	Atenolol 50mg / Nifedipine 20mg modified-release capsules
52728	1077020	Beta-Adalat modified-release capsules (Lexon (UK) Ltd)
61719	1072020	Beta-Adalat modified-release capsules (Waymade Healthcare Plc)
8642	68652020	Tenif 50mg/20mg modified-release capsules (AstraZeneca UK Ltd)
1684	68629020	Beta-Adalat modified-release capsules (Bayer Plc)
28949	84959020	Bi-carzem sr 120mg Modified-release capsule (Tillomed Laboratories Ltd)
20642	84957020	Bi-carzem sr 60mg Modified-release capsule (Tillomed Laboratories Ltd)
5513	74596020	Dilzem sr 60mg Capsule (Elan Pharma)
3676	75265020	Dilzem xl mr 180mg Modified-release capsule (Elan Pharma)
27135	68574020	Diltiazem sr 90mg Capsule (Hillcross Pharmaceuticals Ltd)
2811	57067020	Adizem sr 180mg Modified-release capsule (Napp Pharmaceuticals Ltd)
47415	77105020	Diltiazem sr 60mg Capsule (Hillcross Pharmaceuticals Ltd)
34377	60316020	Diltiazem HCl 90mg Modified-release capsule (Hillcross Pharmaceuticals Ltd)
5296	62587020	Tildiem la 300mg Modified-release capsule (Sanofi)
26270	77217020	Optil xl 300mg Modified-release capsule (Opus Pharmaceuticals Ltd)
60620	21730021	Adizem-XL 240mg capsules (Waymade Healthcare Plc)
1130	84427020	Viazem XL 300mg capsules (Thornton & Ross Ltd)
30197	90599020	Diltiazem 120mg modified-release capsules
7398	84428020	Viazem XL 360mg capsules (Thornton & Ross Ltd)
47732	97275020	Zemret 180 XL capsules (Tillomed Laboratories Ltd)
26309	77216020	Optil xl 240mg Modified-release capsule (Opus Pharmaceuticals Ltd)
1686	57176020	Diltiazem 90mg modified-release capsules
15221	75963020	Dilcardia xl 180mg Modified-release capsule (Generics (UK) Ltd)
17666	84422020	Viazem XL 180mg capsules (Thornton & Ross Ltd)
45759	65091020	Diltiazem HCl 240mg Capsule (PLIVA Pharma Ltd)
23733	69392020	Optil sr 90mg Modified-release capsule (Opus Pharmaceuticals Ltd)
18403	60315020	Diltiazem HCl 180mg Modified-release capsule (Hillcross Pharmaceuticals Ltd)
793	75164020	Adizem xl 240mg Capsule (Napp Pharmaceuticals Ltd)
20890	86888020	Zemtard 240 XL capsules (Galen Ltd)
536	62588020	Tildiem la 200mg Modified-release capsule (Sanofi)
35696	88983020	Kenzem SR 120mg capsules (Kent Pharmaceuticals Ltd)
59863	1895020	Dilzem XL 240 capsules (Lexon (UK) Ltd)
13033	86668020	Angitil XL 240 capsules (Chiesi Ltd)
18852	86924020	Disogram SR 120mg capsules (Ranbaxy (UK) Ltd)
44192	97277020	Zemret 240 XL capsules (Tillomed Laboratories Ltd)
26759	80908020	Zildil SR 60mg capsules (Chanelle Medical UK Ltd)

For peer review only

25777	86145020	Dilcardia SR 120mg capsules (Generics (UK) Ltd)
2592	84421020	Viazem XL 120mg capsules (Thornton & Ross Ltd)
9410	79520020	Angitil SR 120 capsules (Chiesi Ltd)
18830	82004020	Disogram SR 90mg capsules (Ranbaxy (UK) Ltd)
4308	74597020	Dilzem sr 90mg Capsule (Elan Pharma)
13075	87834020	Dilzem XL 180 capsules (Teva UK Ltd)
3370	75264020	Dilzem xl mr 120mg Modified-release capsule (Elan Pharma)
60415	1891020	Dilzem XL 180 capsules (Sigma Pharmaceuticals Plc)
48282	1882020	Diltiazem 90mg modified-release capsules (A A H Pharmaceuticals Ltd)
38831	95539020	Adizem-SR 180mg capsules (Napp Pharmaceuticals Ltd)
27401	88981020	Kenzem SR 90mg capsules (Kent Pharmaceuticals Ltd)
62207	21725021	Adizem-SR 120mg capsules (Waymade Healthcare Plc)
11770	87826020	Dilzem SR 60 capsules (Teva UK Ltd)
13302	87828020	Dilzem SR 90 capsules (Teva UK Ltd)
13926	75622020	Diltiazem 360mg modified-release capsules
23233	84958020	Bi-carzem sr 90mg Modified-release capsule (Tillomed Laboratories Ltd)
26463	81081020	Zemret xl 240mg Capsule (Neo Laboratories Ltd)
47724	95577020	Bi-Carzem XL 240mg capsules (Tillomed Laboratories Ltd)
38865	95559020	Adizem-XL 120mg capsules (Napp Pharmaceuticals Ltd)
27685	65095020	Diltiazem HCl 300mg Capsule (PLIVA Pharma Ltd)
39171	95543020	Bi-Carzem SR 60mg capsules (Tillomed Laboratories Ltd)
19426	79164020	Disogram SR 240mg capsules (Ranbaxy (UK) Ltd)
37774	88979020	Kenzem SR 60mg capsules (Kent Pharmaceuticals Ltd)
26460	75964020	Dilcardia xl 240mg Modified-release capsule (Generics (UK) Ltd)
52276	39293020	Adizem-XL 180mg capsules (DE Pharmaceuticals)
61532	26725021	Diltiazem 120mg modified-release capsules (Sigma Pharmaceuticals Plc)
6309	57068020	Adizem xl 300mg Capsule (Napp Pharmaceuticals Ltd)
26269	69394020	Optil sr 180mg Modified-release capsule (Opus Pharmaceuticals Ltd)
49289	1884020	Diltiazem 120mg modified-release capsules (Alliance Healthcare (Distribution) Ltd)
17425	84733020	Zemtard 120 XL capsules (Galen Ltd)
17406	84734020	Zemtard 180 XL capsules (Galen Ltd)
4635	72177020	Diltiazem 200mg modified-release capsules
10267	81943020	Adizem-XL 200mg capsules (Napp Pharmaceuticals Ltd)
38882	95563020	Adizem-XL 240mg capsules (Napp Pharmaceuticals Ltd)
13240	87832020	Dilzem XL 120 capsules (Teva UK Ltd)
636	86603020	Diltiazem 60mg modified-release capsules
48288	1886020	Diltiazem 120mg modified-release capsules (A A H Pharmaceuticals Ltd)
48457	1881020	Diltiazem 90mg modified-release capsules (Alliance Healthcare (Distribution) Ltd)
4408	76295020	Slozem 240mg capsules (Merck Serono Ltd)
52701	1902020	Tildiem LA 200 capsules (Mawdsley-Brooks & Company Ltd)
44887	86212020	Bi-carzem xl 300mg Capsule (Tillomed Laboratories Ltd)
4808	75522020	Diltiazem 240mg modified-release capsules
16038	87830020	Dilzem SR 120 capsules (Teva UK Ltd)
2686	75266020	Dilzem xl mr 240mg Modified-release capsule (Elan Pharma)
54799	1878020	Tildiem LA 300 capsules (Mawdsley-Brooks & Company Ltd)
17586	80135020	Slozem 300mg capsules (Merck Serono Ltd)
18834	82002020	Disogram SR 60mg capsules (Ranbaxy (UK) Ltd)
30242	90601020	Diltiazem 180mg modified-release capsules

For peer review only

59585	47168020	Uard 120XL capsules (Ennogen Healthcare Ltd)
41489	95547020	Bi-Carzem SR 120mg capsules (Tillomed Laboratories Ltd)
13127	87836020	Dilzem XL 240 capsules (Teva UK Ltd)
36664	81082020	Zemret xl 300mg Capsule (Neo Laboratories Ltd)
18379	86144020	Dilcardia SR 90mg capsules (Generics (UK) Ltd)
31489	86211020	Bi-carzem xl 240mg Capsule (Tillomed Laboratories Ltd)
57208	47167020	Diltiazem 120mg modified-release capsules (Cubic Pharmaceuticals Ltd)
17492	84732020	Zemtard 300 XL capsules (Galen Ltd)
38855	95561020	Adizem-XL 180mg capsules (Napp Pharmaceuticals Ltd)
38634	95575020	Adizem-XL 300mg capsules (Napp Pharmaceuticals Ltd)
48272	1893020	Diltiazem 60mg modified-release capsules (Alliance Healthcare (Distribution) Ltd)
32089	49567020	Diltiazem HCl 120mg Modified-release capsule (Hillcross Pharmaceuticals Ltd)
18874	68687020	Disogram SR 180mg capsules (Ranbaxy (UK) Ltd)
48870	39292020	Adizem-SR 90mg capsules (DE Pharmaceuticals)
47608	97279020	Zemret 300 XL capsules (Tillomed Laboratories Ltd)
23505	81470020	Adizem xl plus 150mg+12.5mg Modified-release capsule (Napp Pharmaceuticals Ltd)
61245	26728021	Diltiazem 60mg modified-release capsules (Sigma Pharmaceuticals Plc)
38632	95541020	Adizem-SR 90mg capsules (Napp Pharmaceuticals Ltd)
11223	79519020	Angitil SR 90 capsules (Chiesi Ltd)
38545	95521020	Tildiem LA 200 capsules (Sanofi)
517	57066020	Adizem sr 120mg Modified-release capsule (Napp Pharmaceuticals Ltd)
15288	86669020	Angitil XL 300 capsules (Chiesi Ltd)
5348	72176020	Diltiazem 300mg modified-release capsules
39298	95545020	Bi-Carzem SR 90mg capsules (Tillomed Laboratories Ltd)
42819	69923020	Diltiazem xl 240mg Capsule (Hillcross Pharmaceuticals Ltd)
3118	72560020	Adizem sr 90mg Modified-release capsule (Napp Pharmaceuticals Ltd)
38876	95519020	Tildiem LA 300 capsules (Sanofi)
32658	75962020	Dilcardia xl 120mg Modified-release capsule (Generics (UK) Ltd)
59098	1889020	Dilzem XL 180 capsules (Lexon (UK) Ltd)
13027	84423020	Viazem XL 240mg capsules (Thornton & Ross Ltd)
56758	47166020	Diltiazem 90mg modified-release capsules (Cubic Pharmaceuticals Ltd)
18404	49568020	Diltiazem 60mg modified-release capsules (A A H Pharmaceuticals Ltd)
31737	80910020	Zildil SR 120mg capsules (Chanelle Medical UK Ltd)
5054	79521020	Angitil SR 180 capsules (Chiesi Ltd)
38818	95537020	Adizem-SR 120mg capsules (Napp Pharmaceuticals Ltd)
47530	76823020	Horizem SR 60mg capsules (Horizon lifecare)
19440	77223020	Disogram SR 300mg capsules (Ranbaxy (UK) Ltd)
5234	76294020	Slozem 180mg capsules (Merck Serono Ltd)
26267	69393020	Optil sr 120mg Modified-release capsule (Opus Pharmaceuticals Ltd)
21145	86146020	Dilcardia SR 60mg capsules (Generics (UK) Ltd)
2528	76293020	Slozem 120mg capsules (Merck Serono Ltd)
3302	54862020	Cardene SR 30mg capsules (Astellas Pharma Ltd)
9386	52064020	Nicardipine 45mg modified-release capsules
5477	52063020	Nicardipine 30mg modified-release capsules
12875	54863020	Cardene SR 45mg capsules (Astellas Pharma Ltd)
737	73827020	Nifedipine 20mg modified-release capsules

For peer review only

4856	73758020	Coracten SR 20mg capsules (UCB Pharma Ltd)
2605	73828020	Nifedipine 10mg modified-release capsules
5162	79993020	Nifedipine 30mg modified-release capsules
3712	79074020	Coracten XL 30mg capsules (UCB Pharma Ltd)
4939	79075020	Coracten XL 60mg capsules (UCB Pharma Ltd)
9750	79994020	Nifedipine 60mg modified-release capsules
2746	73759020	Coracten SR 10mg capsules (UCB Pharma Ltd)
8975	68473020	Verapamil 180mg modified-release capsules
12392	68478020	Univer 180mg modified-release capsules (Teva UK Ltd)
8945	68479020	Univer 240mg modified-release capsules (Teva UK Ltd)
6510	68477020	Univer 120mg modified-release capsules (Teva UK Ltd)
18223	85883020	Trandolapril with verapamil 2mg + 180mg Modified-release capsule
1574	68472020	Verapamil 120mg modified-release capsules
3943	68474020	Verapamil 240mg modified-release capsules
19690	85865020	Verapamil 180mg modified-release / Trandolapril 2mg capsules
20579	85903020	Tarka modified-release capsules (Abbott Laboratories Ltd)
56467	1909020	Tildiem 60mg modified-release tablets (DE Pharmaceuticals)
38964	95553020	Adizem-SR 120mg tablets (Napp Pharmaceuticals Ltd)
34581	68132020	Diltiazem HCl 60mg Modified-release tablet (Kent Pharmaceuticals Ltd)
22619	68606020	Britiazim 60mg Modified-release tablet (Thames Laboratories Ltd)
4732	72564020	Diltiazem 90mg modified-release tablets
21918	85943020	Optil 60mg modified-release tablets (Opus Pharmaceuticals Ltd)
21763	49566020	Diltiazem 60mg modified-release tablets (A A H Pharmaceuticals Ltd)
34475	53802020	Diltiazem HCl 90mg Modified-release tablet (IVAX Pharmaceuticals UK Ltd)
9374	72559020	Adizem 60mg Modified-release tablet (Napp Pharmaceuticals Ltd)
32262	49558020	Diltiazem HCl 60mg Tablet (C P Pharmaceuticals Ltd)
11973	79934020	Calcicard CR 120mg tablets (Teva UK Ltd)
57859	47164020	Diltiazem 90mg modified-release tablets (Cubic Pharmaceuticals Ltd)
49001	11003020	Diltiazem 120mg modified-release tablets (Alliance Healthcare (Distribution) Ltd)
27136	60317020	Diltiazem 90mg modified-release tablets (A A H Pharmaceuticals Ltd)
34824	53803020	Diltiazem HCl 120mg Modified-release tablet (IVAX Pharmaceuticals UK Ltd)
29676	80745020	Calazem 60mg Modified-release tablet (Berk Pharmaceuticals Ltd)
21795	50256020	Retalzem 60 modified-release tablets (Kent Pharmaceuticals Ltd)
13410	70830020	Angiozem 60mg modified-release tablets (Ashbourne Pharmaceuticals Ltd)
41635	53801020	Diltiazem 60mg modified-release tablets (IVAX Pharmaceuticals UK Ltd)
62065	23040021	Diltiazem 90mg modified-release tablets (Colorama Pharmaceuticals Ltd)
21778	54633020	Diltiazem 60mg modified-release tablets (Teva UK Ltd)
1836	71948020	Diltiazem 60mg modified-release tablets
18975	68163020	Calcicard 60mg Tablet (3M Health Care Ltd)
46937	56930020	Diltiazem 60mg modified-release tablets (Actavis UK Ltd)
16850	83067020	Angiozem CR 120mg tablets (Ashbourne Pharmaceuticals Ltd)
49390	1871020	Diltiazem 90mg modified-release tablets (Alliance Healthcare (Distribution) Ltd)
57594	1906020	Tildiem 60mg modified-release tablets (Waymade Healthcare Plc)
21773	60454020	Diltiazem HCl 60mg Tablet (Generics (UK) Ltd)
939	60296020	Tildiem Retard 90mg tablets (Sanofi)
32870	60682020	Diltiazem 60mg modified-release tablets (Sterwin Medicines)

For peer review only

61010	20298021	Diltiazem 120mg modified-release tablets (Cubic Pharmaceuticals Ltd)
219	72563020	Diltiazem 120mg modified-release tablets
43430	61274020	Diltiazem 120mg modified-release tablets (A A H Pharmaceuticals Ltd)
62064	25559021	Diltiazem 120mg modified-release tablets (Mawdsley-Brooks & Company Ltd)
9723	79935020	Calcicard CR 90mg tablets (Teva UK Ltd)
2888	62586020	Tildiem 60mg modified-release tablets (Sanofi)
12639	56931020	Diltiazem HCl 90mg Modified-release tablet (Actavis UK Ltd)
12705	83066020	Angiozem CR 90mg tablets (Ashbourne Pharmaceuticals Ltd)
38066	59378020	Diltiazem HCl 60mg Modified-release tablet (Lagap)
51261	11007020	Tildiem Retard 120mg tablets (Mawdsley-Brooks & Company Ltd)
31676	56932020	Diltiazem HCl 120mg Modified-release tablet (Actavis UK Ltd)
1289	60297020	Tildiem Retard 120mg tablets (Sanofi)
4852	72558020	Adizem sr 120mg Modified-release tablet (Napp Pharmaceuticals Ltd)
26337	76787020	Cabren 10mg modified-release tablets (Teva UK Ltd)
29044	88260020	Neofel XL 10mg tablets (Kent Pharmaceuticals Ltd)
17566	87067020	Felotens XL 10mg tablets (Thornton & Ross Ltd)
38434	93025020	Keloc SR 10mg tablets (Teva UK Ltd)
20459	87085020	Felendil xl 10mg Modified-release tablet (Ratiopharm UK Ltd)
7280	65383020	Plendil 10mg modified-release tablets (AstraZeneca UK Ltd)
29145	91113020	Felendil xl 2.5mg Modified-release tablet (Ratiopharm UK Ltd)
9334	65384020	Plendil 2.5mg modified-release tablets (AstraZeneca UK Ltd)
58339	2044020	Neofel XL 2.5mg tablets (Almus Pharmaceuticals Ltd)
48009	68173020	Felodipine 5mg Modified-release tablet (Sandoz Ltd)
33932	88800020	Parmid XL 5mg tablets (Sandoz Ltd)
491	52181020	Felodipine 2.5mg modified-release tablets
9437	65382020	Plendil 5mg modified-release tablets (AstraZeneca UK Ltd)
25572	86937020	Felogen XL 5mg tablets (Generics (UK) Ltd)
501	52179020	Felodipine 5mg modified-release tablets
43790	92017020	Vascalpha 10mg modified-release tablets (Almus Pharmaceuticals Ltd)
14305	87544020	Vascalpha 10mg modified-release tablets (Actavis UK Ltd)
39357	93501020	Neofel XL 2.5mg tablets (Kent Pharmaceuticals Ltd)
35592	92751020	Cardioplen XL 2.5mg tablets (Chiesi Ltd)
44859	66904020	Felodipine sr 5mg Tablet (Approved Prescription Services Ltd)
33091	66476020	Felodipine 10mg modified-release tablets (A A H Pharmaceuticals Ltd)
55740	2041020	Neofel XL 2.5mg tablets (Actavis UK Ltd)
43394	89315020	Pinefeld XL 10mg tablets (Tillomed Laboratories Ltd)
40633	92015020	Vascalpha 5mg modified-release tablets (Almus Pharmaceuticals Ltd)
37897	94403020	Felotens XL 2.5mg tablets (Thornton & Ross Ltd)
568	52180020	Felodipine 10mg modified-release tablets
36620	88802020	Parmid XL 10mg tablets (Sandoz Ltd)
55306	99312020	Folpik XL 5mg tablets (Teva UK Ltd)
30557	87103020	Felogen XL 10mg tablets (Generics (UK) Ltd)
32922	68176020	Felodipine 10mg Modified-release tablet (Sandoz Ltd)
60652	23052021	Parmid XL 2.5mg tablets (Sandoz Ltd)
24365	87956020	Cardioplen XL 5mg tablets (Chiesi Ltd)
30991	76784020	Cabren 5mg modified-release tablets (Teva UK Ltd)
30915	78440020	Cabren 2.5mg modified-release tablets (Teva UK Ltd)
60884	21738021	Felodipine 2.5mg modified-release tablets (Phoenix Healthcare Distribution Ltd)
10153	87083020	Felendil xl 5mg Modified-release tablet (Ratiopharm UK Ltd)
17557	87065020	Felotens XL 5mg tablets (Thornton & Ross Ltd)
24366	87958020	Cardioplen XL 10mg tablets (Chiesi Ltd)

For peer review only

43512	66473020	Felodipine 5mg modified-release tablets (A A H Pharmaceuticals Ltd)
60569	20172021	Felodipine 2.5mg modified-release tablets (Waymade Healthcare Plc)
28721	88258020	Neofel XL 5mg tablets (Kent Pharmaceuticals Ltd)
35084	87542020	Vascalpha 5mg modified-release tablets (Actavis UK Ltd)
28438	85815020	Triapin 2.5mg/2.5mg modified-release tablets (Sanofi)
17474	78452020	Felodipine 5mg modified-release / Ramipril 5mg tablets
17006	85824020	Triapin 5mg/5mg modified-release tablets (Sanofi)
21162	78451020	Felodipine 2.5mg modified-release / Ramipril 2.5mg tablets
24228	83201020	Nimodrel 20mg modified-release tablet (Opus Pharmaceuticals Ltd)
52017	1952020	Adalat LA 30 tablets (Mawdsley-Brooks & Company Ltd)
5181	71013020	Angiopine MR 20mg tablets (Ashbourne Pharmaceuticals Ltd)
47027	63403020	Nifedipine 10mg Modified-release tablet (Kent Pharmaceuticals Ltd)
45685	99249020	Adanif XL 30mg tablets (Focus Pharmaceuticals Ltd)
13699	83276020	Angiopine la 40mg Tablet (Ashbourne Pharmaceuticals Ltd)
33025	90565020	Nimodrel XL 30mg tablets (Zurich Pharmaceuticals)
22217	83200020	Nimodrel 10mg modified-release tablet (Opus Pharmaceuticals Ltd)
20257	75490020	Cardilate MR 20mg tablets (IVAX Pharmaceuticals UK Ltd)
1262	65057020	Nifedipine 12 20mg Modified-release tablet
49762	1947020	Nifedipine 10mg modified-release tablets (Alliance Healthcare (Distribution) Ltd)
60856	23048021	Nifedipine 10mg modified-release tablets (Sigma Pharmaceuticals Plc)
11512	84876020	Nifedipress MR 10 tablets (Dexcel-Pharma Ltd)
34146	61467020	Nifedipine mr 10mg Modified-release tablet (IVAX Pharmaceuticals UK Ltd)
53500	1950020	Adalat LA 30 tablets (DE Pharmaceuticals)
20311	57957020	Nifedipress mr 20mg Modified-release tablet (Generics (UK) Ltd)
47285	73272020	Nifedipine xl 60mg Tablet (Hillcross Pharmaceuticals Ltd)
25132	90751020	Nifopress MR 20mg tablets (Teva UK Ltd)
59163	47174020	Nifedipine 20mg modified-release tablets (Cubic Pharmaceuticals Ltd)
53278	1953020	Adalat LA 30 tablets (Necessity Supplies Ltd)
9485	50475020	Hypolar Retard 20 tablets (Sandoz Ltd)
1300	54944020	Nifensar xl 20mg Modified-release tablet (Rhone-Poulenc Rorer Ltd)
11769	77698020	Calchan MR 20 tablets (Ranbaxy (UK) Ltd)
17448	84211020	Nifedipress mr 10mg Modified-release tablet (Sterwin Medicines)
8213	75515020	Nifedipine 24 20mg Modified-release tablet
21886	85847020	Nifedipress MR 20 tablets (Actavis UK Ltd)
58557	1975020	Adalat LA 20 tablets (Necessity Supplies Ltd)
41979	85923020	Adipine la 30mg Modified-release tablet (Chiesi Ltd)
14861	77697020	Calchan MR 10 tablets (Ranbaxy (UK) Ltd)
43818	98791020	Adalat LA 60 tablets (Bayer Plc)
43753	98789020	Adalat LA 30 tablets (Bayer Plc)
35646	91231020	Neozipine XL 60mg tablets (Kent Pharmaceuticals Ltd)
9573	54677020	Slofedipine XL 30mg tablets (Zentiva)
34101	61468020	Nifedipine mr 20mg Modified-release tablet (IVAX Pharmaceuticals UK Ltd)
10136	84877020	Nifedipress MR 20 tablets (Dexcel-Pharma Ltd)
13139	88895020	Adipine XL 30mg tablets (Chiesi Ltd)
43410	75517020	Nifedipine extra 60mg Modified-release tablet
21216	87081020	Hypolar Retard 10mg tablets (Sandoz Ltd)
12613	81213020	Unipine xl 30mg Modified-release tablet (Genus Pharmaceuticals Ltd)
19170	82932020	Tensipine MR 10 tablets (Thornton & Ross Ltd)
34187	59923020	Nifedipine 10mg Modified-release tablet (Generics (UK) Ltd)
28688	60425020	Nifedipine 10mg modified-release tablets (A A H Pharmaceuticals Ltd)
541	84301020	Adalat LA 20 tablets (Bayer Plc)

For peer review only

17325	75491020	Cardilate MR 10mg tablets (Teva UK Ltd)
16073	86867020	Nifedipress MR 10 tablets (Teva UK Ltd)
51917	1957020	Adalat LA 60 tablets (Sigma Pharmaceuticals Plc)
5806	82933020	Tensipine MR 20 tablets (Thornton & Ross Ltd)
3930	80874020	Nifedipine 60mg modified-release tablets
12606	76104020	Nifelease 20mg Modified-release tablet (Eastern Pharmaceuticals Ltd)
25919	49118020	Nifedipine 20mg modified-release tablets (A A H Pharmaceuticals Ltd)
57653	1970020	Adalat LA 20 tablets (Sigma Pharmaceuticals Plc)
4239	80337020	Adipine MR 10 tablets (Chiesi Ltd)
30199	68509020	Nifedipine 30mg modified-release tablets
25646	86340020	Nivaten retard 20mg Modified-release tablet (Actavis UK Ltd)
37530	91229020	Neozipine XL 30mg tablets (Kent Pharmaceuticals Ltd)
47217	85924020	Adipine la 60mg Modified-release tablet (Chiesi Ltd)
47614	60424020	Nifedipine 30mg modified-release tablets (A A H Pharmaceuticals Ltd)
5277	83624020	Fortipine LA 40 tablets (AMCo)
10246	88897020	Adipine XL 60mg tablets (Chiesi Ltd)
410	68508020	Nifedipine 10mg modified-release tablets
55824	59219020	Nifedipine 20mg Modified-release tablet (Berk Pharmaceuticals Ltd)
22696	84383020	Slofedipine 20mg tablets (Sterwin Medicines)
34115	68510020	Nifedipine 60mg Modified-release tablet
3711	79719020	Adipine MR 20 tablets (Chiesi Ltd)
13672	71014020	Angiopine MR 10mg tablets (Ashbourne Pharmaceuticals Ltd)
10135	86770020	Nifedipress mr 10mg Modified-release tablet (Sandoz Ltd)
9269	80875020	Nifedipine 40mg modified-release tablets
47887	90567020	Nimodrel XL 60mg tablets (Zurich Pharmaceuticals)
30473	86711020	Coroday MR 20mg tablets (Generics (UK) Ltd)
4227	64631020	Adalat la 60mg Tablet (Bayer Plc)
20591	86868020	Nifedipress MR 20 tablets (Teva UK Ltd)
7541	57458020	Nifopress Retard 20mg tablets (AMCo)
23736	86128020	Hypolar XL 30 tablets (Sandoz Ltd)
15715	85589020	Genalat retard 20mg Modified-release tablet (Wyeth Pharmaceuticals)
58990	47173020	Nifedipine 10mg modified-release tablets (Cubic Pharmaceuticals Ltd)
1449	80873020	Nifedipine 24 30mg Modified-release tablet
39800	94186020	Valni XL 60mg tablets (Zentiva)
56469	1958020	Adalat LA 60 tablets (Necessity Supplies Ltd)
53629	1972020	Adalat retard 20mg tablets (Lexon (UK) Ltd)
37025	90709020	Nifedipine 20mg modified-release tablets
21245	85846020	Nifedipress mr 10mg Modified-release tablet (Actavis UK Ltd)
9553	54678020	Slofedipine XL 60 tablets (Zentiva)
57531	1954020	Adalat LA 60 tablets (Waymade Healthcare Plc)
2280	52574020	Adalat retard 10mg tablets (Bayer Plc)
49338	1966020	Nifedipine 20mg modified-release tablets (Alliance Healthcare (Distribution) Ltd)
17338	84579020	Nifedotard 20 mr 20mg Modified-release tablet (Galen Ltd)
46887	99251020	Adanif XL 60mg tablets (Focus Pharmaceuticals Ltd)
17342	86339020	Nivaten retard 10mg Modified-release tablet (Actavis UK Ltd)
43222	97381020	Valni 20 Retard tablets (Tillomed Laboratories Ltd)
2343	52575020	Adalat retard 20mg tablets (Bayer Plc)
37184	94184020	Valni XL 30mg tablets (Zentiva)
17599	88811020	Verapress MR 240mg tablets (Sandoz Ltd)
13856	84570020	Verapress MR 240mg tablets (Actavis UK Ltd)
19457	84468020	Ranvera MR 240mg tablets (Ranbaxy (UK) Ltd)
13251	78368020	Vera-Til SR 240mg tablets (Tillomed Laboratories Ltd)

For peer review only

47222	75703020	Verapamil 120mg modified-release tablets (A A H Pharmaceuticals Ltd)
700	84862020	Vera-Til SR 120mg tablets (Tillomed Laboratories Ltd)
16328	78409020	Verapress MR 240mg tablets (Dexcel-Pharma Ltd)
59264	872020	Securon SR 240mg tablets (DE Pharmaceuticals)
45308	71935020	Verapamil 240mg modified-release tablets (Generics (UK) Ltd)
9569	65404020	Verapamil 120mg modified-release tablets
1298	65403020	Verapamil 240mg modified-release tablets
11972	86724020	Vertab SR 240 tablets (Chiesi Ltd)
19459	51963020	Verapamil 240mg modified-release tablets (A A H Pharmaceuticals Ltd)
47230	59413020	Verapamil 240mg modified-release tablets (Teva UK Ltd)
46884	65991020	Verapamil hc 240mg Modified-release tablet (Sandoz Ltd)
13965	71435020	Cordilox MR 240mg tablets (Teva UK Ltd)
3343	56683020	Half Securon SR 120mg tablets (Abbott Laboratories Ltd)
30462	85740020	Ethimil MR 240mg tablets (Genus Pharmaceuticals Ltd)
3342	58870020	Securon SR 240mg tablets (Abbott Laboratories Ltd)
43879	97884020	Vera-Til SR 240mg tablets (Actavis UK Ltd)
42625	97882020	Vera-Til SR 120mg tablets (Actavis UK Ltd)
45051	56381020	Verapamil hc 240mg Modified-release tablet (Actavis UK Ltd)
51461	865020	Securon SR 240mg tablets (Waymade Healthcare Plc)
29637	89660020	Verapress MR 240mg tablets (Teva UK Ltd)
47529	98903020	Nifedipine 20mg/ml oral drops
46233	91269020	Amlodipine Oral solution
46724	415021	Amlodipine 5mg/5ml oral solution
55257	19777020	Diltiazem 60mg/5ml oral solution
47707	93331020	Nifedipine Oral solution
31490	81761020	Zolvera 40mg/5ml oral solution (Rosemont Pharmaceuticals Ltd)
11777	75258020	Verapamil 40mg/5ml oral solution sugar free
54983	18978020	Amlodipine 2.5mg/5ml oral suspension
61374	29717020	Amlodipine 4mg/5ml oral suspension
45070	99159020	Amlodipine 10mg/5ml oral suspension
16162	91263020	Amlodipine 5mg/5ml oral suspension
47002	413021	Amlodipine 10mg/5ml sugar free Oral suspension
11922	90541020	Diltiazem 60mg/5ml oral suspension
26774	91013020	Nifedipine 10mg/5ml Oral suspension
53357	20553020	Nifedipine 10mg/5ml oral suspension
37726	92999020	Nifedipine 100mg/5ml oral suspension
53990	20557020	Nifedipine 5mg/5ml oral suspension
27295	70927020	Securon IV 5mg/2ml solution for injection ampoules (Abbott Laboratories Ltd)
19325	48764020	Cordilox 2.5mg/ml Injection (IVAX Pharmaceuticals UK Ltd)
26674	68346020	Verapamil 5mg/2ml solution for injection ampoules
31761	88254020	Amlostin 10mg tablets (Discovery Pharmaceuticals Ltd)
17640	88252020	Amlostin 5mg tablets (Discovery Pharmaceuticals Ltd)
61422	2002020	Amlodipine 5mg tablets (Accord Healthcare Ltd)
59001	68889020	Amlodipine 10mg tablets (Generics (UK) Ltd)
54696	68498020	Amlodipine 10mg tablets (Sandoz Ltd)
39914	67735020	Amlodipine 5mg tablets (Teva UK Ltd)
749	88863020	Amlodipine 5mg tablets
56147	2023020	Amlodipine 10mg tablets (Accord Healthcare Ltd)
43880	75222020	Amlodipine 5mg tablets (Almus Pharmaceuticals Ltd)
6856	88865020	Amlodipine 10mg tablets
32917	67688020	Amlodipine 5mg tablets (IVAX Pharmaceuticals UK Ltd)

For peer review only

54654	75178020	Amlodipine 10mg tablets (Almus Pharmaceuticals Ltd)
42210	68111020	Amlodipine 10mg tablets (Zentiva)
59762	67739020	Amlodipine 10mg tablets (Teva UK Ltd)
39804	70039020	Amlodipine 5mg tablets (Dr Reddy's Laboratories (UK) Ltd)
58580	38962020	Amlodipine 10mg tablets (APC Pharmaceuticals & Chemicals (Europe) Ltd)
46792	99771020	Olmesartan medoxomil with amlodipine and hydrochlorothiazide 40mg + 5mg + 12.5mg Tablet
49636	38506020	Amlodipine 10mg tablets (DE Pharmaceuticals)
3917	72822020	Istin 5mg tablets (Pfizer Ltd)
55358	99777020	Olmesartan medoxomil with amlodipine and hydrochlorothiazide 40mg + 10mg + 25mg Tablet
45279	68495020	Amlodipine 5mg tablets (Sandoz Ltd)
46687	99769020	Olmesartan medoxomil with amlodipine and hydrochlorothiazide 20mg + 5mg + 12.5mg Tablet
32595	67634020	Amlodipine 5mg tablets (A A H Pharmaceuticals Ltd)
46715	99773020	Olmesartan medoxomil with amlodipine and hydrochlorothiazide 40mg + 10mg + 12.5mg Tablet
34093	67637020	Amlodipine 10mg tablets (A A H Pharmaceuticals Ltd)
60244	2021020	Amlodipine 10mg tablets (Phoenix Healthcare Distribution Ltd)
56334	2018020	Amlodipine 10mg tablets (Bristol Laboratories Ltd)
36202	68410020	Amlodipine 10mg tablets (Actavis UK Ltd)
47467	99775020	Olmesartan medoxomil with amlodipine and hydrochlorothiazide 40mg + 5mg + 25mg Tablet
53868	68406020	Amlodipine 5mg tablets (Actavis UK Ltd)
54515	2008020	Amlodipine 10mg tablets (Alliance Healthcare (Distribution) Ltd)
54633	1996020	Amlodipine 5mg tablets (Bristol Laboratories Ltd)
5914	72823020	Istin 10mg tablets (Pfizer Ltd)
43470	75838020	Amlodipine 5mg tablets (Wockhardt UK Ltd)
40316	96923020	Olmesartan medoxomil 20mg / Amlodipine 5mg tablets
39984	96929020	Sevikar 20mg/5mg tablets (Daiichi Sankyo UK Ltd)
35096	92773020	Exforge 10mg/160mg tablets (Novartis Pharmaceuticals UK Ltd)
47573	99783020	Sevikar HCT 40mg/5mg/12.5mg tablets (Daiichi Sankyo UK Ltd)
53220	99791020	Sevikar HCT 40mg/10mg/25mg tablets (Daiichi Sankyo UK Ltd)
8310	69563020	Isradipine 2.5mg tablets
8257	69560020	Prescal 2.5mg tablets (Novartis Pharmaceuticals UK Ltd)
11966	74740020	Motens 2mg tablets (GlaxoSmithKline UK Ltd)
5158	74744020	Lacidipine 2mg tablets
3221	74745020	Lacidipine 4mg tablets
56994	77966020	Lacidipine 4mg tablets (Teva UK Ltd)
57680	78002020	Lacidipine 4mg tablets (A A H Pharmaceuticals Ltd)
9670	74741020	Motens 4mg tablets (GlaxoSmithKline UK Ltd)
60699	2051020	Lacidipine 2mg tablets (Sigma Pharmaceuticals Plc)
61611	23689021	Lercanidipine 10mg tablets (DE Pharmaceuticals)
5570	85619020	Zanidip 10mg tablets (Recordati Pharmaceuticals Ltd)
56767	77347020	Lercanidipine 20mg tablets (Generics (UK) Ltd)
14300	90707020	Zanidip 20mg tablets (Recordati Pharmaceuticals Ltd)
5593	79553020	Lercanidipine 10mg tablets
57444	14783021	Lercanidipine 10mg tablets (Aptil Pharma Ltd)
59233	77287020	Lercanidipine 20mg tablets (Actavis UK Ltd)
47331	77345020	Lercanidipine 10mg tablets (Generics (UK) Ltd)
13243	90705020	Lercanidipine 20mg tablets
1854	64630020	Adalat la 30mg Tablet (Bayer Plc)
38107	73256020	Nifedipine sr 30mg Tablet (Hillcross Pharmaceuticals Ltd)

For peer review only

10595	68599020	Nimotop 30mg tablets (Bayer Plc)
11547	74190020	Nimodipine 30mg tablets
40668	96927020	Olmesartan medoxomil 40mg / Amlodipine 10mg tablets
40639	96925020	Olmesartan medoxomil 40mg / Amlodipine 5mg tablets
41203	96933020	Sevikar 40mg/10mg tablets (Daiichi Sankyo UK Ltd)
41205	96931020	Sevikar 40mg/5mg tablets (Daiichi Sankyo UK Ltd)
46355	99779020	Sevikar HCT 20mg/5mg/12.5mg tablets (Daiichi Sankyo UK Ltd)
60067	22976021	Perindopril erbumine 4mg / Amlodipine 5mg tablets
60684	22974021	Perindopril erbumine 4mg / Amlodipine 10mg tablets
35317	92769020	Exforge 5mg/80mg tablets (Novartis Pharmaceuticals UK Ltd)
26252	53578020	Berkatens 160mg Tablet (Berk Pharmaceuticals Ltd)
32590	60639020	Verapamil 40mg tablets (Generics (UK) Ltd)
46009	63423020	Verapamil 120mg tablets (Kent Pharmaceuticals Ltd)
35729	54551020	Verapamil 80mg tablets (Teva UK Ltd)
28843	50788020	Verapamil hc 80mg Tablet (Celltech Pharma Europe Ltd)
33471	56374020	Verapamil 40mg tablets (Actavis UK Ltd)
8524	58375020	Securon 40mg Tablet (Abbott Laboratories Ltd)
12104	53065020	Cordilox 160mg tablets (IVAX Pharmaceuticals UK Ltd)
3057	58377020	Securon 120mg tablets (Abbott Laboratories Ltd)
41693	60641020	Verapamil 120mg tablets (Generics (UK) Ltd)
1118	65395020	Verapamil 40mg tablets
28844	53573020	Berkatens 120mg Tablet (Berk Pharmaceuticals Ltd)
34959	50799020	Verapamil 40mg tablets (A A H Pharmaceuticals Ltd)
16677	48759020	Cordilox 80mg tablets (IVAX Pharmaceuticals UK Ltd)
39009	54550020	Verapamil 40mg tablets (Teva UK Ltd)
10688	69592020	Verapamil 160mg tablets
1120	65396020	Verapamil 80mg tablets
1748	53062020	Cordilox 120mg tablets (IVAX Pharmaceuticals UK Ltd)
41586	56375020	Verapamil 80mg tablets (Actavis UK Ltd)
41679	56209020	Verapamil 80mg tablets (IVAX Pharmaceuticals UK Ltd)
8884	48758020	Cordilox 40mg tablets (IVAX Pharmaceuticals UK Ltd)
1747	65397020	Verapamil 120mg tablets
40405	54552020	Verapamil 120mg tablets (Teva UK Ltd)
46955	60640020	Verapamil 80mg tablets (Generics (UK) Ltd)
22826	56814020	Securon 160mg Tablet (Abbott Laboratories Ltd)
19175	56208020	Verapamil 40mg tablets (IVAX Pharmaceuticals UK Ltd)
23872	53571020	Berkatens 40mg Tablet (Berk Pharmaceuticals Ltd)
25059	53572020	Berkatens 80mg Tablet (Berk Pharmaceuticals Ltd)
10832	58376020	Securon 80mg Tablet (Abbott Laboratories Ltd)
31711	50800020	Verapamil 80mg tablets (A A H Pharmaceuticals Ltd)
29	72826020	Amlodipine besilate 5mg tablets
729	87704020	Amlodipine maleate 5mg tablets
6477	87706020	Amlodipine maleate 10mg tablets
71	72827020	Amlodipine besilate 10mg tablets
35304	92957020	Valsartan 160mg with amlodipine 10mg tablets
35173	92953020	Valsartan 160mg with amlodipine 5mg tablets
35174	92951020	Valsartan 80mg with amlodipine 5mg tablets
1538	68602020	Diltiazem 60mg tablets
47616	99785020	Sevikar HCT 40mg/10mg/12.5mg tablets (Daiichi Sankyo UK Ltd)
47727	99789020	Sevikar HCT 40mg/5mg/25mg tablets (Daiichi Sankyo UK Ltd)
35343	92765020	Amlodipine 5mg / Valsartan 160mg tablets
35189	92767020	Amlodipine 10mg / Valsartan 160mg tablets
35697	92771020	Exforge 5mg/160mg tablets (Novartis Pharmaceuticals UK Ltd)
35329	92763020	Amlodipine 5mg / Valsartan 80mg tablets

For peer review only

For peer review only

Thiazides and thiazide-like diuretics

Product code	GEM code	Description
23505	81470020	Adizem xl plus 150mg+12.5mg Modified-release capsule (Napp Pharmaceuticals Ltd)
3701	69292020	Amiloride 2.5mg / hydrochlorothiazide 25mg tablets
2002	69291020	Amiloride 5mg / hydrochlorothiazide 50mg tablets
4034	69293020	Amiloride 5mg / hydrochlorothiazide 50mg/5ml solution
3526	69296020	Amiloride with atenolol with hydrochlorothiazide capsules
30519	69277020	Amiloride with timolol with hydrochlorothiazide tablets
8526	48201020	Aprinox 2.5mg tablets (AMCo)
7698	48202020	Aprinox 5mg tablets (Amdipharm Plc)
13526	63763020	Atenix Co 100 tablets (Ashbourne Pharmaceuticals Ltd)
21873	63762020	Atenix Co 50 tablets (Ashbourne Pharmaceuticals Ltd)
1788	67598020	Atenolol 100mg with Chlortalidone 25mg tablets
581	67597020	Atenolol 50mg with Chlortalidone 12.5mg tablets
4983	68844020	Atenolol with amiloride and hydrochlorothiazide capsules
15457	48303020	Baycaron 25mg Tablet (Bayer Plc)
1211	67326020	Bendroflumethiazide 2.5mg / Potassium chloride 630mg (potassium 8.4mmol) modified-release tablets
34803	62827020	Bendroflumethiazide 2.5mg Tablet (Regent Laboratories Ltd)
2	58976020	Bendroflumethiazide 2.5mg tablets
33651	48846020	Bendroflumethiazide 2.5mg tablets (A A H Pharmaceuticals Ltd)
34059	55391020	Bendroflumethiazide 2.5mg tablets (Actavis UK Ltd)
40886	68065020	Bendroflumethiazide 2.5mg tablets (Almus Pharmaceuticals Ltd)
33415	63225020	Bendroflumethiazide 2.5mg tablets (Generics (UK) Ltd)
27689	57793020	Bendroflumethiazide 2.5mg tablets (IVAX Pharmaceuticals UK Ltd)
47844	66033020	Bendroflumethiazide 2.5mg tablets (Kent Pharmaceuticals Ltd)
34602	66500020	Bendroflumethiazide 2.5mg tablets (Sovereign Medical Ltd)
31670	54691020	Bendroflumethiazide 2.5mg tablets (Teva UK Ltd)
27256	48838020	Bendroflumethiazide 2.5mg tablets (Wockhardt UK Ltd)
22912	67620020	Bendroflumethiazide 2.5mg with Propanolol 80mg capsules
19142	67631020	Bendroflumethiazide 2.5mg with Timolol maleate 10mg tablets
7351	90559020	Bendroflumethiazide 2.5mg/5ml oral suspension
58	58977020	Bendroflumethiazide 5mg tablets
23427	48847020	Bendroflumethiazide 5mg tablets (A A H Pharmaceuticals Ltd)
34124	55390020	Bendroflumethiazide 5mg tablets (Actavis UK Ltd)
40149	57796020	Bendroflumethiazide 5mg tablets (IVAX Pharmaceuticals UK Ltd)
41517	54690020	Bendroflumethiazide 5mg tablets (Teva UK Ltd)
31820	48839020	Bendroflumethiazide 5mg tablets (Wockhardt UK Ltd)
11338	67627020	Bendroflumethiazide 5mg with Nadolol 40mg tablets
23131	67621020	Bendroflumethiazide 5mg with Propanolol 160mg modified-release capsules
53812	92033020	Bendroflumethiazide oral solution
30272	64126020	Benthiazide with Triamterene capsules
21803	48386020	Berkozide 2.5mg Tablet (Berk Pharmaceuticals Ltd)
21867	48387020	Berkozide 5mg Tablet (Berk Pharmaceuticals Ltd)
3203	68134020	Capozide LS Tablet (E R Squibb and Sons Ltd)
32166	80831020	Capto-co 25mg+50mg Tablet (IVAX Pharmaceuticals UK Ltd)
11641	68130020	Captopril 25mg with Hydrochlorothiazide 12.5mg tablets
10902	68129020	Captopril 50mg with Hydrochlorothiazide 25mg tablets
17655	72036020	Carace 10 Tablet (Bristol-Myers Squibb Pharmaceuticals Ltd)
39147	95901020	Carace 20 Plus tablets (Merck Sharp & Dohme Ltd)
9764	72035020	Carace 20 Tablet (Bristol-Myers Squibb Pharmaceuticals Ltd)

For peer review only

62066	23031021	Cardide SR 1.5mg tablets (Teva UK Ltd)
18973	52358020	Centyl 2.5mg Tablet (Edwin Burgess Ltd)
29991	52359020	Centyl 5mg Tablet (Edwin Burgess Ltd)
13246	90731020	Chlorothiazide 150mg/5ml oral suspension
54679	98525020	Chlorothiazide 250mg tablets
59834	32028020	Chlorothiazide 250mg/5ml oral solution
6816	86950020	Chlorothiazide 250mg/5ml oral suspension
56804	19362020	Chlorothiazide 25mg/5ml oral suspension
8836	61214020	Chlorothiazide 500mg tablets
60603	19370020	Chlorothiazide 50mg/5ml oral suspension
54341	30321020	Chlorothiazide 5mg/5ml oral suspension
55889	91770020	Chlorothiazide oral solution
3548	61268020	Chlortalidone 100mg tablets
19055	61275020	Chlortalidone 12.5mg with Atenolol 50mg tablets
16786	61276020	Chlortalidone 25mg with Atenolol 100mg tablets
605	61267020	Chlortalidone 50mg tablets
18733	70712020	Co-amilozide 5mg with 50mg/ml oral solution
10316	83981020	CoAprovel 150mg/12.5mg tablets (Sanofi)
11526	83982020	CoAprovel 300mg/12.5mg tablets (Sanofi)
35196	92367020	CoAprovel 300mg/25mg tablets (Sanofi)
5721	73883020	Co-tenidone 100mg/25mg tablets
34899	53522020	Co-tenidone 100mg/25mg tablets (A A H Pharmaceuticals Ltd)
46952	56911020	Co-tenidone 100mg/25mg tablets (Actavis UK Ltd)
37725	60353020	Co-tenidone 100mg/25mg tablets (Generics (UK) Ltd)
34012	55246020	Co-tenidone 100mg/25mg tablets (IVAX Pharmaceuticals UK Ltd)
41572	54654020	Co-tenidone 100mg/25mg tablets (Teva UK Ltd)
9783	73882020	Co-tenidone 50mg/12.5mg tablets
32094	53521020	Co-tenidone 50mg/12.5mg tablets (A A H Pharmaceuticals Ltd)
31708	56910020	Co-tenidone 50mg/12.5mg tablets (Actavis UK Ltd)
34449	60352020	Co-tenidone 50mg/12.5mg tablets (Generics (UK) Ltd)
34034	55247020	Co-tenidone 50mg/12.5mg tablets (IVAX Pharmaceuticals UK Ltd)
34825	54653020	Co-tenidone 50mg/12.5mg tablets (Teva UK Ltd)
51258	34961020	Coversyl Arginine Plus 5mg/1.25mg tablets (DE Pharmaceuticals)
37908	94709020	Coversyl Arginine Plus 5mg/1.25mg tablets (Servier Laboratories Ltd)
52145	61691020	Cyclopenthiazide 0.25mg with oxprenolol 160mg modified-release tablets
1170	61684020	Cyclopenthiazide 500microgram tablets
2833	61687020	CYCLOPENTHIAZIDE -K tablets
26220	68015020	Delvas Tablet (Berk Pharmaceuticals Ltd)
18606	81472020	Diltiazem and hydrochlorothiazide 150mg+12.5mg modified-release capsules
4044	49062020	Diurexan 20mg tablets (Meda Pharmaceuticals Ltd)
33724	86952020	Diuril 250mg/5ml oral suspension (Imported (United States))
5189	51068020	Enalapril 20mg / Hydrochlorothiazide 12.5mg tablets
18267	64533020	Enduron 5mg Tablet (Abbott Laboratories Ltd)
17252	49321020	Esidrex 25mg Tablet (Novartis Pharmaceuticals UK Ltd)
13363	49322020	Esidrex 50mg Tablet (Novartis Pharmaceuticals UK Ltd)
41885	95348020	Ethibide XL 1.5mg tablets (Genus Pharmaceuticals Ltd)
13525	58231020	Hydrenox 50mg Tablet (Knoll Ltd)
542	63248020	Hydrochlorothiazide 25mg tablets
3517	63249020	Hydrochlorothiazide 50mg tablets
48132	94685020	Hydrochlorothiazide Capsule
57488	92809020	Hydrochlorothiazide Oral solution

For peer review only

19890	63252020	Hydrochlorothiazide with amiloride 25mgwith2.5mg Tablet
22923	63254020	Hydrochlorothiazide with amiloride 50mg with 5mg Tablet
28177	68841020	Hydrochlorothiazide with atenolol and amiloride Capsule
15135	69269020	Hydrochlorothiazide with captopril 12.5mg with 25mg Tablet
11133	69268020	Hydrochlorothiazide with captopril 25mg with 50mg Tablet
38367	94427020	Hydrochlorothiazide with losartan 12.5mg with 100mg Tablet
14738	79035020	Hydrochlorothiazide with losartan 12.5mg with 50mg Tablet
24632	89781020	Hydrochlorothiazide with losartan 25mg with 100mg Tablet
29427	68980020	Hydrochlorothiazide with metoprolol tartrate 12.5mg with 100mg tablet
33659	68979020	Hydrochlorothiazide with metoprolol tartrate 25mg with 200mg Modified-release tablet
35380	90735020	Hydrochlorothiazide with olmesartan medoxomil 12.5mg with 20mg tablet
39021	90733020	Hydrochlorothiazide with olmesartan medoxomil 25mg with 20mg tablet
21182	69280020	Hydrochlorothiazide with timolol and amiloride 25mg with 10mg with 2.5mg Tablet
15127	63257020	Hydrochlorothiazide with triamterene 25mgwith50mg Tablet
24484	87996020	Hydrochlorothiazide with valsartan 12.5mg with 160mg Tablet
24268	88422020	Hydrochlorothiazide with valsartan 12.5mg with 80mg Tablet
23456	88006020	Hydrochlorothiazide with valsartan 25mg with 160mg Tablet
12110	63279020	Hydroflumethiazide 50mg Tablet
29529	63282020	Hydroflumethiazide with spironolactone 25mg+25mg Tablet
45916	63283020	Hydroflumethiazide with spironolactone 50mg+50mg Tablet
3054	49873020	Hygroton 100mg Tablet (Alliance Pharmaceuticals Ltd)
3997	49872020	Hygroton 50mg tablets (Alliance Pharmaceuticals Ltd)
8891	49876020	Hygroton -k Tablet (Novartis Pharmaceuticals UK Ltd)
25500	68145020	Hypertane 50 Tablet (Schwarz Pharma Ltd)
5112	63541020	Indapamide 1.5mg modified-release tablets
46675	75757020	Indapamide 1.5mg modified-release tablets (A A H Pharmaceuticals Ltd)
60020	14463021	Indapamide 1.5mg modified-release tablets (Waymade Healthcare Plc)
2612	63540020	Indapamide 2.5mg tablets
48099	60381020	Indapamide 2.5mg tablets (A A H Pharmaceuticals Ltd)
43516	57007020	Indapamide 2.5mg tablets (Actavis UK Ltd)
54316	683020	Indapamide 2.5mg tablets (Alliance Healthcare (Distribution) Ltd)
56296	685020	Indapamide 2.5mg tablets (Boston Healthcare Ltd)
56760	690020	Indapamide 2.5mg tablets (Co-Pharma Ltd)
34551	60492020	Indapamide 2.5mg tablets (Generics (UK) Ltd)
40907	68581020	Indapamide 2.5mg tablets (Genus Pharmaceuticals Ltd)
55259	682020	Indapamide 2.5mg tablets (Kent Pharmaceuticals Ltd)
42906	63983020	Indapamide 2.5mg tablets (Niche Generics Ltd)
49529	689020	Indapamide 2.5mg tablets (Phoenix Healthcare Distribution Ltd)
33083	56755020	Indapamide 2.5mg tablets (Teva UK Ltd)
48079	59993020	Indapamide 2.5mg tablets (Zentiva)
44168	97012020	Indipam XL 1.5mg tablets (Actavis UK Ltd)
1021	51047020	Innozide 20mg/12.5mg tablets (Merck Sharp & Dohme Ltd)
11448	80124020	Irbesartan 150mg / Hydrochlorothiazide 12.5mg tablets
11469	80125020	Irbesartan 300mg / Hydrochlorothiazide 12.5mg tablets
35481	92365020	Irbesartan 300mg / Hydrochlorothiazide 25mg tablets
28157	53155020	Kalspare ls Tablet (Dominion Pharma)
12546	53154020	Kalspare Tablet (Dominion Pharma)

For peer review only

6786	50823020	Lisinopril 10mg / Hydrochlorothiazide 12.5mg tablets
37710	67694020	Lisinopril 10mg / Hydrochlorothiazide 12.5mg tablets (Teva UK Ltd)
6468	50822020	Lisinopril 20mg / Hydrochlorothiazide 12.5mg tablets
55399	67847020	Lisinopril 20mg / Hydrochlorothiazide 12.5mg tablets (A A H Pharmaceuticals Ltd)
54201	75924020	Lisinopril 20mg / Hydrochlorothiazide 12.5mg tablets (Almus Pharmaceuticals Ltd)
33353	67697020	Lisinopril 20mg / Hydrochlorothiazide 12.5mg tablets (Teva UK Ltd)
56244	1491020	Lisinopril 20mg / Hydrochlorothiazide 12.5mg tablets (Tillomed Laboratories Ltd)
8147	50252020	Lopresoretic Tablet (Novartis Pharmaceuticals UK Ltd)
43184	97115020	Mapemid XL 1.5mg tablets (Teva UK Ltd)
17143	64368020	Mefruside 25mg Tablet
8464	64409020	Meprobamate with bendroflumethiazide Tablet
8602	50380020	Metenix 5mg tablets (Sanofi)
20057	64530020	Methyclothiazide 5mg Tablet
53674	45436020	Metolazone 2.5mg tablets
49752	33486020	Metolazone 2.5mg/5ml oral solution
4334	63211020	Metolazone 500microgram low dose Tablet
4332	64663020	Metolazone 5mg tablets
55777	33494020	Metolazone 5mg/5ml oral solution
54329	33496020	Metolazone 5mg/5ml oral suspension
54643	94928020	Metolazone Oral solution
15488	68972020	Metoprolol tartrate with chlortalidone Tablet
3293	50482020	Moduretic Oral solution (Bristol-Myers Squibb Pharmaceuticals Ltd)
348	50481020	Moduretic Tablet (Bristol-Myers Squibb Pharmaceuticals Ltd)
27957	78343020	Natramid 2.5mg Tablet (Trinity Pharmaceuticals Ltd)
7641	58678020	Natrilix 2.5mg tablets (Servier Laboratories Ltd)
3056	84103020	Natrilix SR 1.5mg tablets (Servier Laboratories Ltd)
2046	50633020	Navidrex 500microgram tablets (AMCo)
1125	50636020	Navidrex -k Tablet (Novartis Pharmaceuticals UK Ltd)
24189	75589020	Neo-bendromax 2.5mg Tablet (Ashbourne Pharmaceuticals Ltd)
24190	75590020	Neo-bendromax 5mg Tablet (Ashbourne Pharmaceuticals Ltd)
46302	99566020	Neo-Naclex 2.5mg tablets (AMCo)
1209	50651020	Neo-Naclex 5mg tablets (Mercury Pharma Group Ltd)
1213	50654020	Neo-Naclex-K modified-release tablets (Mercury Pharma Group Ltd)
12360	50657020	Nephril 1mg Tablet (Pfizer Ltd)
26275	75814020	Nindaxa 2.5 tablets (Ashbourne Pharmaceuticals Ltd)
8058	63267020	Normetic Tablet (Abbott Laboratories Ltd)
18200	90629020	Olmesartan medoxomil 20mg / Hydrochlorothiazide 12.5mg tablets
18903	90631020	Olmesartan medoxomil 20mg / Hydrochlorothiazide 25mg tablets
43322	98337020	Olmesartan medoxomil 40mg / Hydrochlorothiazide 12.5mg tablets
46687	99769020	Olmesartan medoxomil with amlodipine and hydrochlorothiazide 20mg + 5mg + 12.5mg Tablet
46715	99773020	Olmesartan medoxomil with amlodipine and hydrochlorothiazide 40mg + 10mg + 12.5mg Tablet
55358	99777020	Olmesartan medoxomil with amlodipine and hydrochlorothiazide 40mg + 10mg + 25mg Tablet
46792	99771020	Olmesartan medoxomil with amlodipine and hydrochlorothiazide 40mg + 5mg + 12.5mg Tablet
47467	99775020	Olmesartan medoxomil with amlodipine and hydrochlorothiazide 40mg + 5mg + 25mg Tablet
29634	90635020	Olmetec Plus 20mg/12.5mg tablets (Daiichi Sankyo UK Ltd)
27520	90637020	Olmetec Plus 20mg/25mg tablets (Daiichi Sankyo UK Ltd)

For peer review only

43915	98339020	Olmetec Plus 40mg/12.5mg tablets (Daiichi Sankyo UK Ltd)
26256	78942020	Opumide 2.5mg Tablet (Opus Pharmaceuticals Ltd)
8673	73918020	Oxprenolol with cyclopenthiazide 160mg+0.25mg Modified-release tablet
50607	92909020	Perindopril arginine 2mg with Indapamide 625 micrograms tablet
48098	99501020	Perindopril arginine 4mg with Indapamide 1.25mg tablet
37978	94277020	Perindopril arginine 5mg / Indapamide 1.25mg tablets
6794	83957020	Perindopril erbumine 4mg / Indapamide 1.25mg tablets
56157	47939020	Perindopril tosilate 5mg / Indapamide 1.25mg tablets
21025	67090020	Prestim forte Tablet (LEO Pharma)
8623	52834020	Prestim Tablet (ICN Pharmaceuticals France S.A.)
59616	678020	Rawel XL 1.5mg tablets (Consilient Health Ltd)
17720	51466020	Saluric 500mg Tablet (Merck Sharp & Dohme Ltd)
31131	75349020	Spiro-co 25mg+25mg Tablet (IVAX Pharmaceuticals UK Ltd)
25505	75350020	Spiro-co 50mg+50mg Tablet (IVAX Pharmaceuticals UK Ltd)
8521	66716020	Spironolactone 25mg with hydroflumethiazide 25mg tablet
7961	66717020	Spironolactone 50mg with hydroflumethiazide 50mg tablet
8303	51854020	Tenavoid Tablet (Edwin Burgess Ltd)
26248	57165020	Tenchlor 100mg/25mg tablets (Teva UK Ltd)
31470	57164020	Tenchlor 50mg/12.5mg tablets (Teva UK Ltd)
1288	51857020	Tenoret 50mg/12.5mg tablets (AstraZeneca UK Ltd)
1124	54815020	Tenoretic 100mg/25mg tablets (AstraZeneca UK Ltd)
41861	95960020	Tensaid XL 1.5mg tablets (Generics (UK) Ltd)
25730	69274020	Timolol maleate with amiloride and hydrochlorothiazide Tablet
12517	67082020	Timolol maleate with bendroflumethiazide 20mg + 5mg Tablet
24280	80150020	Totaretic 100mg+25mg Tablet (C P Pharmaceuticals Ltd)
26741	80149020	Totaretic 50mg+12.5mg Tablet (C P Pharmaceuticals Ltd)
37294	67220020	Triamterene with chlortalidone 50mg + 25mg Tablet
9223	67204020	Triamterene with hydrochlorothiazide 50mg + 25mg Tablet
39447	95950020	Varbim XL 1.5mg tablets (Teva UK Ltd)
24008	72539020	Vasetic Tablet (Shire Pharmaceuticals Ltd)
7618	65442020	Xipamide 20mg tablets
19352	71715020	Xuret 0.5mg Tablet (Galen Ltd)
61846	45437020	Zaroxolyn 2.5mg tablets (IDIS)
6359	51463020	Zestoretic 10- 10mg+12.5mg Tablet (AstraZeneca UK Ltd)
39137	95875020	Zestoretic 10 tablets (AstraZeneca UK Ltd)
57539	1503020	Zestoretic 10 tablets (Sigma Pharmaceuticals Plc)
2982	51462020	Zestoretic 20- 20mg+12.5mg Tablet (AstraZeneca UK Ltd)
38995	95877020	Zestoretic 20 tablets (AstraZeneca UK Ltd)
26219	79843020	Zida-co 5mg+50mg Tablet (Opus Pharmaceuticals Ltd)

For peer review only

Centrally acting antihypertensives

Product code	GEM code	Description
60136	30024020	Clonidine 100micrograms/5ml oral solution
54467	28325020	Clonidine 300micrograms/24hours transdermal patches
27894	!1375104	CLONIDINE HYDROCHLORIDE
58090	30048020	Clonidine 75micrograms/5ml oral solution
28790	!1152103	CATAPRES
25088	!1152102	CATAPRES
27883	6602007	CATAPRES 15 MCG/ML INJ
20808	!1153101	CATAPRES PERLONGETS
2649	2258007	METHYLDOPA 250 MG CAP
25836	4723007	METHYLDOPA 200 MG TAB
49684	28322020	Clonidine 100micrograms/24hours transdermal patches
58529	18103020	Clonidine 200micrograms/24hours transdermal patches
6694	61497020	Clonidine 300microgram tablets
5289	72345020	Clonidine 250microgram modified-release capsules
16248	61500020	Clonidine 150micrograms/1ml solution for injection ampoules
2878	61496020	Clonidine 100microgram tablets
23380	48571020	Catapres 300microgram tablets (Boehringer Ingelheim Ltd)
4215	48570020	Catapres 100microgram tablets (Boehringer Ingelheim Ltd)
30293	58536020	Catapres 150micrograms/1ml solution for injection ampoules (Boehringer Ingelheim Ltd)
52555	19534020	Clonidine 50micrograms/5ml oral solution
53142	19536020	Clonidine 50micrograms/5ml oral suspension
8296	54917020	Catapres PL Perlongets 250microgram capsules (Boehringer Ingelheim Ltd)
24196	54853020	Dopamet 250mg Tablet (Berk Pharmaceuticals Ltd)
1707	59031020	Methyldopa 250mg tablets
3070	59032020	Methyldopa 500mg tablets
32913	50045020	Methyldopa 250mg tablets (Actavis UK Ltd)
28738	64556020	Methyldopa with hydrochlorothiazide Tablet
29570	54852020	Dopamet 125mg Tablet (Berk Pharmaceuticals Ltd)
7642	48071020	Aldomet 500mg Tablet (Merck Sharp & Dohme Ltd)
8033	48069020	Aldomet 125mg Tablet (Merck Sharp & Dohme Ltd)
7626	48070020	Aldomet 250mg Tablet (Merck Sharp & Dohme Ltd)
25275	51709020	Metalpha 500mg Tablet (Ashbourne Pharmaceuticals Ltd)
9225	77535020	Methyldopa 250mg Capsule
41661	50040020	Methyldopa 250mg Tablet (C P Pharmaceuticals Ltd)
43988	98722020	Aldomet 250mg tablets (Aspen Pharma Trading Ltd)
21346	64564020	Hydromet Tablet (MSD Thomas Morson Pharmaceuticals)
43989	98724020	Aldomet 500mg tablets (Aspen Pharma Trading Ltd)
14390	54403020	Aldomet 250mg/5ml Liquid (Merck Sharp & Dohme Ltd)
25289	54854020	Dopamet 500mg Tablet (Berk Pharmaceuticals Ltd)
23761	64552020	Methyldopa 250mg/5ml oral suspension
3049	59030020	Methyldopa 125mg tablets
18252	51708020	Metalpha 250mg Tablet (Ashbourne Pharmaceuticals Ltd)
26919	64553020	Methyldopa 50mg/ml Injection
7416	54406020	Aldomet 50mg/ml Injection (Merck Sharp & Dohme Ltd)
43531	68547020	Moxonidine 400microgram tablets (Sandoz Ltd)
9876	81117020	Physiotens 300microgram tablets (Abbott Healthcare Products Ltd)
60898	69282020	Moxonidine 200microgram tablets (Generics (UK) Ltd)
11177	81115020	Physiotens 200microgram tablets (Abbott Healthcare Products Ltd)
7174	81109020	Moxonidine 400microgram tablets

For peer review only

10253	81110020	Moxonidine 300microgram tablets
9749	81116020	Physiotens 400microgram tablets (Abbott Healthcare Products Ltd)
33322	68542020	Moxonidine 200microgram tablets (Sandoz Ltd)
40310	69037020	Moxonidine 200microgram tablets (Teva UK Ltd)
61036	45215020	Physiotens 300microgram tablets (Actavis UK Ltd)
4993	81108020	Moxonidine 200microgram tablets

For peer review only

Renin inhibitors

Product code	GEM code	Description
36878	93930020	Rasilez 150mg tablets (Novartis Pharmaceuticals UK Ltd)
36879	93932020	Rasilez 300mg tablets (Novartis Pharmaceuticals UK Ltd)
36909	93928020	Aliskiren 300mg tablets
36629	93926020	Aliskiren 150mg tablets

Vasodilators

Product code	GEM code	Description
25506	3361007	DIAZOXIDE 15 MG INJ
21749	5813007	HYDRALAZINE HCl 100 MG TAB
214	5817007	HYDRALAZINE 1 MG SYR
31971	4887007	HYDRALAZINE 6.25 MG SYR
4507	4885007	HYDRALAZINE HCl 12.5 MG TAB
23746	6300007	HYDRALAZINE HCl 10 MG TAB
22772	!8504292	SILDENAFIL
22819	!8504294	SILDENAFIL
22786	!8504293	SILDENAFIL
40527	95326020	Ambrisentan 10mg tablets
40528	95322020	Ambrisentan 5mg tablets
29561	78290020	Bosentan 125mg tablets
29560	78287020	Bosentan 62.5mg tablets
58632	78296020	Tracleer 125mg tablets (Actelion Pharmaceuticals UK Ltd)
47654	78293020	Tracleer 62.5mg tablets (Actelion Pharmaceuticals UK Ltd)
32267	62432020	Diazoxide 300mg/20ml solution for injection ampoules
13317	48198020	Apresoline 20mg powder for solution for injection ampoules (AMCo)
2362	48196020	Apresoline 25mg tablets (AMCo)
2680	48197020	Apresoline 50mg Tablet (Sovereign Medical Ltd)
18861	89188020	Hydralazine 10mg/5ml oral suspension
504	63242020	Hydralazine 20mg powder for solution for injection ampoules
573	58989020	Hydralazine 25mg tablets
31220	49792020	Hydralazine 25mg tablets (A A H Pharmaceuticals Ltd)
43500	49788020	Hydralazine 25mg tablets (Actavis UK Ltd)
1296	58990020	Hydralazine 50mg tablets
41639	49789020	Hydralazine 50mg tablets (Actavis UK Ltd)
59512	20076020	Hydralazine 50mg/5ml oral solution
61116	20078020	Hydralazine 50mg/5ml oral suspension
30341	87552020	Iloprost 20micrograms/2ml nebuliser liquid ampoules
29256	87554020	Ventavis 10micrograms/ml nebuliser solution 2ml ampoules (Schering Health Care Ltd)
53896	2181020	Iloprost 100micrograms/1ml solution for infusion ampoules
36840	93019020	Iloprost 10micrograms/1ml nebuliser liquid ampoules
51328	14602020	Iloprost 50micrograms/0.5ml solution for infusion ampoules
38519	93021020	Ventavis 10micrograms/ml nebuliser solution 1ml ampoules (Bayer Plc)
14495	64774020	Loniten 10mg tablets (Pfizer Ltd)
9697	64772020	Loniten 2.5mg tablets (Pfizer Ltd)
9463	64773020	Loniten 5mg tablets (Pfizer Ltd)
2968	64769020	Minoxidil 10mg tablets

For peer review only

2970	64767020	Minoxidil 2.5mg tablets
2967	64768020	Minoxidil 5mg tablets
46249	99911020	Revatio 10mg/12.5ml solution for injection vials (Pfizer Ltd)
30967	90581020	Revatio 20mg tablets (Pfizer Ltd)
46795	99909020	Sildenafil 10mg/12.5ml solution for injection vials
27137	90579020	Sildenafil 20mg tablets
45641	99463020	Sildenafil 40mg/50ml solution for injection vials
36612	66660020	Sodium nitroprusside 50mg powder for solution for infusion vials
47264	99180020	Adcirca 20mg tablets (Eli Lilly and Company Ltd)
6207	84307020	Tadalafil 20mg tablets

For peer review only

eTable 3. Frequency of different types of lifestyle advice given during follow-up

Advice type	Number of patients	%
Any lifestyle advice	34,397	31.6%
Advice about smoking	22,456	20.6%
Advice about diet	4,343	4.0%
Advice about exercise	3,778	3.5%
Advice about alcohol consumption	2,039	1.9%
Advice about weight	1,309	1.2%
Other advice	10,200	9.4%

Note: Some patients received more than one different type of advice

For peer review only

ISAC protocol 16_008R

Approved: 14th March 2016

PROTOCOL INFORMATION

In order to help ensure that protocols submitted for review contain adequate information for protocol evaluation, ISAC have produced guidance on the content of protocols for research using CPRD data. This guidance is available on the CPRD website (www.cprd.com/ISAC). All protocols using CPRD data which are submitted for review by ISAC must contain information on all the areas detailed below. If a specific area required by ISAC is not applicable to your protocol, please provide the justification underneath the relevant heading.

The protocol section (next page) has pre-defined headings and the protocol must be written using these headings. Additional headings are not acceptable; however, supplementary information may be placed in one or more of the appendices providing this information is essential and an appropriate reference to it is made within the protocol. Unless very short, codes lists should be placed in an Appendix. Applications will be regarded as invalid and returned to the applicant if any of the headings below are missing or if additional sections are included.

Please note that ISAC will not consider any application where the protocol exceeds 12 pages (excluding sections A-F of the application form and annexes). Annexes should be kept to a minimum and contain only vital information that could not be provided in the main protocol section. A font-size of at least 12 should be used. Protocols not exceeding 15 pages would be acceptable if ISAC has required a resubmission where additional information is requested.

Please note, your protocol will not be reviewed by ISAC if it falls short of the above requirements. You are advised to speak to the Secretariat if you have any queries.

Voluntary registration of ISAC approved studies:
Epidemiological studies are increasingly being included in registries of research around the world, including those primarily set up for clinical trials. To increase awareness amongst researchers of ongoing research, ISAC encourages voluntary registration of epidemiological research conducted using MHRA databases. This will not replace information on ISAC approved protocols that may be published on the CPRD website. It is for the applicant to determine the most appropriate registry for their study. Please inform the ISAC secretariat that you have registered a protocol and provide the location.

For peer review only - http://bmjopen.bmj.com/site/about/guidelines.xhtml

Protocol Section

A. Lay Summary (Max. 200 words)

Little is known about whether patients with moderately high blood pressure (mild hypertension) and a low risk of heart attack/stroke should be given medication to lower blood pressure. A definitive trial is unlikely to happen because it would be too expensive and require too many patients. Yet many guidelines encourage pharmacological treatment, and many patients receive it despite little evidence of benefit.

This study will examine the extent to which patients are prescribed pharmacological treatment for low risk mild hypertension, and explore the safety and effectiveness of this treatment using data from a large database of Primary Care records. Death from all causes will be compared in patients prescribed blood pressure lowering medication and those not prescribed medication, matched by characteristics such as age, sex, smoking status, prescription of other medications and history of disease. This approach will allow the effectiveness of pharmacological treatment to be examined in a large number of patients, followed-up for many years. This study will inform hypertension management guidelines, potentially leading to improved patient care.

B. Technical Summary (Max. 200 words)

Evidence to guide the pharmacological treatment of patients with uncomplicated (low cardiovascular disease risk), mild hypertension (blood pressure 140/90-159/99mmHg) is lacking. A definitive trial is unlikely due to the costs and numbers of patients required because of low event rates in this population. Yet many guidelines encourage treatment, and many patients are thought to receive it, despite little evidence of benefit.

This study will examine the extent to which patients are prescribed treatment for uncomplicated, mild hypertension in routine practice and the safety and efficacy of this treatment for using data from a large database of Primary Care records. The primary outcome of the study will be the rate of all-cause mortality in patients with uncomplicated mild hypertension prescribed antihypertensive treatment vs. those not prescribed therapy, matched using propensity scores estimating the likelihood of receiving treatment. Secondary outcomes will include the rate of cardiovascular morbidity/mortality, side effects to medication and rate of cancer in treated vs. non treated patients. These outcomes will be assessed using Cox proportional hazards modelling. The proportion of patients given lifestyle advice and/or pharmacological treatment for uncomplicated mild hypertension will also be estimated by year, and predictors of lifestyle advice/pharmacological treatment will be examined using logistic regression.

C. Objectives, Specific Aims and Rationale

Objective

Examine the extent to which patients are prescribed pharmacological treatment for uncomplicated, mild hypertension in routine practice (descriptive objective) and whether such treatment is safe and effective and reducing the risk of death and cardiovascular disease (hypothesis testing).

For peer review only - http://bmjopen.bmj.com/site/about/guidelines.xhtml

Specific aims

Descriptive objective

1. Establish the proportion of patients given lifestyle advice and/or pharmacological treatment for uncomplicated mild hypertension.
2. Examine the impact of the introduction of the Quality and Outcomes Framework (QOF) and NHS health check programme on the proportion of patients given lifestyle advice and/or pharmacological treatment for uncomplicated mild hypertension.
3. Examine factors which predict the likelihood of being given lifestyle advice/pharmacological treatment for uncomplicated mild hypertension in routine practice.

Hypothesis testing

1. **(Primary analysis)** Examine the impact of antihypertensive prescription on all-cause mortality in patients with uncomplicated (i.e. low cardiovascular disease risk mild hypertension (i.e. sustained blood pressure between 140/90-159/99mmHg).
2. Examine the impact of antihypertensive prescription on cardiovascular disease morbidity and mortality (myocardial infarction [MI], non-MI acute coronary syndrome, stroke, heart failure) in patients with uncomplicated mild hypertension.
3. Examine the impact of antihypertensive prescription on cancer morbitdity and mortality in patients with uncomplicated mild hypertension.
4. Examine the impact of antihypertensive prescription on hospital admissions related to side effects to medication (hypotension, syncope, bradycardia, electrolyte abnormalities, injurious falls and acute kidney injury/renal failure) in patients with uncomplicated mild hypertension.

Rationale

As discussed above and below, many patients with mild hypertension receive treatment, despite little evidence of benefit. The present proposal describes two distinct analyses using data from the Clinical Practice Research Datalink (CPRD). The first will examine the extent to which guidelines for the management of uncomplicated mild hypertension are followed and how this has changed over time. Factors which predict the likelihood of receiving antihypertensive treatment will be examined and this information will be used in a propensity score model to explore the safety and efficacy of antihypertensive treatment in these patients. These analyses will establish how many patients exist with uncomplicated mild hypertension, how it is currently managed in routine practice, and provide useful non-randomised data on treatment efficacy which will help guide future management decisions or inform whether future trial evidence is required.

D. Background

High blood pressure (hypertension) is a key risk factor for the development of cardiovascular disease[1] and a major cause of morbidity and mortality worldwide.[2] Classifications of hypertension are by definition arbitrary, but many guidelines recommend pharmacological treatment of blood pressure when it is sustained above 140/90mmHg, regardless of the underlying risk, end organ damage, diabetes or history of cardiovascular disease (table 1).[3-8] These recommendations have long been considered controversial,[9-13] particularly with regard to the treatment of uncomplicated (*i.e.* low cardiovascular disease risk) people with 'mild' hypertension (*i.e.* sustained blood pressure between 140/90-159/99mmHg).

There is little evidence to support initiation of pharmacological treatment in patients with uncomplicated mild hypertension and that which exists remains inconclusive. The recent SPRINT trial[14] did enrol a large

For peer review only - http://bmjopen.bmj.com/site/about/guidelines.xhtml

number of patients with mild hypertension (6,174 patients had a baseline systolic blood pressure <145mmHg) and compared a strategy of intensive blood pressure lowering against usual care. They observed significant reductions in death and cardiovascular mortality with intensive treatment, but all enrolled patients had at least one cardiovascular risk factor (in addition to high blood pressure) and thus would not be considered 'uncomplicated'. A recent Cochrane review by Diao *et al.,*[15] examined 8,912 patients from four clinical trials investigating the benefits of treating uncomplicated mild hypertension compared to placebo. They found no significant reduction in mortality with treatment (RR 0.85, 95% CI 0.63-1.15), nor was any reduction in coronary artery disease, stroke or total cardiovascular events observed in a subgroup of 7,080 patients. However, both the authors of that review and subsequent commentators[10 15] point to a lack of power in previous trials and meta-analyses to show significant results.

More recently, Sundström and colleagues[16] attempted to resolve this issue using individual patient data from the Blood Pressure Lowering Treatment Triallists Collaboration and aggregate data from trials identified in a systematic review. Whilst they found significant associations between blood pressure reductions with treatment and stroke or total death, the population studied still included a significant proportion of patients with diabetes, and thus would be considered high risk (not 'uncomplicated') under current clinical guidelines.[17] There are simply too few randomised data in low risk patients to make adequately powered treatment comparisons. Indeed, based on data from the Cochrane Review, a trial of treatment vs. placebo for mild hypertension would need to recruit over 100,000 patients, followed up for 5 years, to achieve statistical significance. The cost of funding such a trial mean it is unlikely to ever be conducted.

As a result, clinical guidelines make important recommendations based on 'expert opinion' alone. These guidelines are predictably contradictory, with those in the UK promoting lifestyle modification in low risk patients[17] whilst guidelines from Europe[6] and America[4 7 8] encourage prescription of drug therapy. The impact of these recommendations is not trivial. A recent study from the UK[18] suggested the costs of treating patients with uncomplicated mild hypertension could reach £229 million in the first year alone, although these estimates are based on regional data extrapolated to national population estimates. Against this background, a number of policy changes have occurred in the UK over the past 10 years which are likely to have affected general practitioner's treatment decisions. In particular, the introduction of the NHS health check in 2009[19] which will likely have increased the number of patients being diagnosed with uncomplicated mild hypertension, and QOF indicators briefly introduced in 2013 promoting treatment in this population.[20]

The proposed study will establish the national prevalence of uncomplicated mild hypertension in Primary Care, how it is currently managed in routine practice, and provide useful non-randomised data on treatment efficacy which will help guide future management decisions or inform whether future trial evidence is required and feasible.

E. Study Type

Hypothesis testing.

F. Study Design

Longitudinal cohort study.

For peer review only - http://bmjopen.bmj.com/site/about/guidelines.xhtml

G. Sample Size

Using estimates of treatment effect size from the recent Cochrane Review by Diao *et al.,* (risk ratio of 0.85 for all-cause mortality with treatment), and assuming a survival rate of 97.9% in the control group over 5 years, approximately 102,776 patients (51,388 in each group) and 1,600 events will be required to accurately define the relationship between antihypertensive treatment and all-cause mortality with 90% power and up to 20% withdrawal/crossover. The present analysis will utilise data from all available patients in the CPRD (with data linkage to ONS and HES) who fulfil the study eligibility criteria. We have examined the number of potentially eligible patients in a random sample of 20 general practices (413,279 patients) contributing to the CPRD database. Approximately 56,785 patients were identified with a diagnosis of hypertension, of which 8,791 (2.1%) fulfilled the study eligibility criteria (including 2,796 patients prescribed medication). The median length of follow-up in this population was 5.6 years. Extrapolating these data to the total number of patients within the CPRD database with linked data (10,022,053 patients) suggests a sample of 213,169 patients (including 67,803 patients on treatment) would be available.

H. Data Linkage Required (if applicable)

Data linkage to the ONS and Basic HES are required to define primary and secondary outcomes in the study. The ONS mortality register will be used to define the primary outcome of the study, all-cause mortality (and also censor follow-up at death). Specific linkages required will include data and ICD-10 coded cause of death (see appendix 2). Linkages to Basic Inpatient HES will be combined with data from the ONS to define secondary outcomes in the study, specifically death or admission to hospital with cardiovascular disease (defined previously), cancer or side effects to medication (hypotension, syncope, bradycardia, electrolyte abnormalities, injurious falls and acute kidney injury/renal failure) (see appendix 2). HES data will also be used to define patients eligibility for the study (e.g. previous stroke), and define the study population (e.g. ethnicity where unavailable in Primary Care records) – but only where it is recorded before the index date so as not to bias the completeness of data by those suffering an event during the study period. Data required from Basic Inpatient HES will include Primary diagnosis, secondary diagnosis, patient characteristics (e.g. sex, ethnicity), date of admission and date of discharge. All deaths and hospital admissions occurring after a patient's index date will be included. A linkage to the Index of Multiple Deprivation is required to acquire practice and patient level deciles of multiple deprivation. However, patients with missing IMD deprivation data will not be excluded, since these data will not be used to define any study outcomes.

Linkage to the MINAP dataset has not been requested due to limitations in the resources available for the study. However, this is not expected to a significant impact on the findings of the study, since:

- A recent study of national database recording of non-fatal MI found that CPRD and HES alone capture 92% of all events.[21]
- The MINAP dataset is unlikely to contribute any additional mortality data used for defining the primary outcome of this study which is not already captured in the ONS mortality register, CPRD or HES.
- Even if the databases used do not capture all outcomes of interest, we do not see any reason to expect the likelihood of an outcome being recorded in MINAP alone to be confounded by treatment status.

For peer review only - http://bmjopen.bmj.com/site/about/guidelines.xhtml

I. Study Population

Individual patient data will be extracted from the medical records of all patients registered at general practices contributing to the CPRD in the UK. Patients will be entered into the study on the 'index date', and exit at the first of the following time points to occur:

- date at which the most recent linked data are available from the CPRD (March 2015),
- Date of the most recent data upload from the practice to which a given patient is registered
- Date at which a given patient transfers out of a registered CPRD practice
- Date of death

Definition of the index date

The index will be defined as 12 months after the date of the third consecutive blood pressure reading between 140/90-159/99mmHg (readings must be taken within 12 months of each other) occurring after the study start date 01/01/1998. This date has been selected because it represents the date from which all relevant data linkages were first available (see below for details). Eligible patients will be those fulfilling the following criteria:

- Linked general practice, Hospital Episodes Statistics (HES) and Office for National Statistics (ONS) mortality records.
- Aged between 18-74 years.
- No Read code for stroke, myocardial infarction (MI), angina, coronary heart disease, peripheral vascular disease, heart failure, left ventricular hypertrophy, atrial fibrillation, diabetes or chronic kidney disease or family history of premature heart disease
- Three consecutive blood pressure readings between 140/90-159/99mmHg (within 12 months of each other).
- No record of any blood pressure lowering medication prescription in the 12 months prior to the third consecutive blood pressure reading between 140/90-159/99 mmHg.
- Classified as a CPRD 'acceptable patient'
- Registered to a CPRD practice classified as 'up-to-standard'

Exclusions will be defined using read coded diagnoses listed in the appendix (see appendix 2 and 3).

J. Selection of comparison group(s) or controls

Intervention group

The intervention in the present study will be sustained prescription of blood pressure lowering therapy to low risk patients with stage 1 hypertension. Patients considered in the treatment group will be those fulfilling the criteria above, who are initiated on blood pressure lowering treatment in the 12 months between the date of the third consecutive BP readings and the index date. The analysis will employ an intention-to-treat approach, so after initiation of treatment, patients will remain in the study/allocation until the last day of follow-up or until they are censored due to mortality, or cardiovascular morbidity.

Control group

Patients in the control group will be those fulfilling the eligibility criteria who were not initiated on blood pressure lowering treatment in the 12 months between the date of the third consecutive BP readings and the index date.Those prescribed therapy more than 12 months after the original index date will be included in the control group.

Patients eligible for the control and treatment groups will be matched using the index date and propensity scores, which reduce the bias introduced in observational studies because patients are not randomised to

For peer review only - http://bmjopen.bmj.com/site/about/guidelines.xhtml

their treatment allocation. Propensity scores indicate the likelihood a patient will be prescribed treatment on the basis of their known (pre-treatment) characteristics and other known information which might influence the decision to treat. This method is advantageous because it allows patients to be matched on multiple characteristics using a single variable (the propensity score), although this means associations between specific covariates and outcomes cannot be assessed.

K. Exposures, Outcomes and Covariates

Exposures

The exposure variable (blood pressure lowering treatment) in the primary analysis in this study will be defined as a prescription of an angiotensin-converting-enzyme inhibitor, angiotensin-II-receptor blocker, calcium channel blocker, thiazide or thiazide-like diuretic, beta-blocker, alpha-blocker or any other antihypertensive listed in the British National Formulary (see appendix 1). Reads codes for being offered lifestyle advice will also be extracted (see appendix 1).

Outcomes

The main aim of this study is to examine the safety and efficacy of treatment in uncomplicated, mild hypertensives. All-cause mortality has been chosen as the primary outcome for this analysis because it permits assessment of the possible benefits of treatment (reduction in death from cardiovascular disease) and the safety of treatment (in terms of risk of non-cardiovascular death) combined in a single outcome. Secondary outcomes will include:

Descriptive secondary outcomes

- The proportion of patients given lifestyle advice and/or pharmacological treatment for uncomplicated mild hypertension.
- The likelihood of patients being given lifestyle advice and/or pharmacological treatment for uncomplicated mild hypertension before and after the introduction of QOF and the NHS health check programme.
- Factors which predict the likelihood of being given lifestyle advice/ pharmacological treatment

Hypothesis testing secondary outcomes

- death or hospitalisation from major cardiovascular events (MI, non-MI acute coronary syndrome, stroke, heart failure or death from cardiovascular disease) using the same definition as that used in the SPRINT trial[14]
- death or hospitalisation from stroke
- death or hospitalisation from MI or non-MI acute coronary syndrome
- death or hospitalisation from cancer
- hospitalisation with suspected side effects to medication (hypotension, syncope, bradycardia, electrolyte abnormalities, injurious falls and acute kidney injury/renal failure)

Outcomes will be captured from hospital admissions (including date of admission and discharge and primary diagnosis), read coded diagnoses in the CPRD and death certificates occurring after the index date using linked data from the Basic Inpatient HES and ONS mortality register (for codes see appendix 2).

Covariates

Data relating to baseline patient characteristics (age, sex, ethnicity [where available], deprivation), lifestyle factors (smoking status [never, current, ex-smoker], excessive alcohol consumption [more than 6/8 units a day or 35/50 units a week for men/women], and BMI [where available]), pre-treatment blood pressure

For peer review only - http://bmjopen.bmj.com/site/about/guidelines.xhtml

readings (in the preceeding 12 months), non-cardiovascular related co-morbidities (rheumatoid arthritis, high total cholesterol [>5mmol/l]) and all statin/antiplatelet medications prescribed will be extracted from the medical records of eligible patients. For subgroup analyses taking into account a patient's apparent adherence to treatment, 'daily equivalent dose' data will be extracted from CPRD and the medication possession ratio (percentage of days when medication was available [MPR]) will be estimated from the sum of tablets prescribed, divided by the number of days of follow-up.

L. Data/ Statistical analysis

The primary analysis will use a propensity score-matched longitudinal cohort study design to examine the safety and efficacy of antihypertensive treatment. Secondary analyses will include an interrupted time-series analysis of prescriptions and lifestyle advice given to patients diagnosed with uncomplicated mild hypertension, comparing before and after the introduction of the NHS health check programme.

Descriptive objectives

Aim 1 – Descriptive analyses

Descriptive statistics will be used to estimate the proportion of patients being offered lifestyle advice or therapy following a diagnosis of uncomplicated mild hypertension (aim 1). Estimates will be adjusted for age and sex and results will be stratified by year of diagnosis.

Aim 2 – Interrupted time-series analysis

The impact of the introduction of the NHS health check programme (April 2009) and changes to QOF indicators (April 2013) and on prescription rates will be examined using an interrupted time-series analysis. Rates of pharmacological treatment and or lifestyle advice will be estimated before and after these specific policy changes and the overall level and slope of the rates in the pre and post policy change periods will be compared using segmented regression.

Aim 3 – Propensity score model

Predictors of lifestyle advice and antihypertensive medication prescription following a diagnosis of uncomplicated mild hypertension (defined as three consecutive blood pressure readings between 140/90-159/99mmHg within a 12 month period) will be explored in a logistic regression model examining the association between the outcome of interest and patients and practice characteristics: age, sex, ethnicity, blood pressure, deprivation (practice level), smoking status, BMI, number of existing antihypertensive medication prescriptions, presence of statin or antiplatelet prescriptions, the presence of rheumatoid arthritis or hypercholesterolemia, calendar year of the index date and the general practice to which the patient is registered. The model examining predictors of pharmacological treatment will be used to generate a propensity score matched cohort for analysis of the primary outcome. The success of propensity score matching will be examined by comparing the characteristics patients in each group pre- and post-matching using chi-squared tests and t-tests. To assess the reduction in bias achieved by propensity score matching, the proportional reduction in the difference between each patient characteristic in the treatment and control groups will estimated and compared pre- and post-matching.

Hypothesis testing

Aim 4 - Primary analysis

Using an intention-to-treat approach, the efficacy of antihypertensive treatment will be examined with Cox proportional hazards modelling, comparing all-cause mortality in those prescribed (any) antihypertensive treatment vs. those not prescribed treatment. Where hazards are not proportional, non-parametric

For peer review only - http://bmjopen.bmj.com/site/about/guidelines.xhtml

methods such as inverse probability weights will be considered. Kaplan-Meier plots will be produced to display the cumulative incidence of all-cause mortality in the treatment group compared to the control group. Hazard ratios will be generated and adjusted by any other factors, unbalanced at baseline, not already included in the initial propensity score matching.

Aims 5-7 – Secondary analyses

Where appropriate (and sufficiently powered to detect an effect), hazard ratios will be calculated using Cox proportional hazards modelling, examining the association between treatment status and secondary endpoints in the study: major cardiovascular disease event, hospitalisation and/or death from stroke, or MI or non-MI acute coronary syndrome and pre-specified side effects to medication.

Subgroup analyses

Subgroup analyses will be conducted to examine the impact of patient adherence to medication on the effectiveness of treatment. We will stratify our analysis by quantiles of medication possession ratio and the treatment effects will be compared between groups. Other pre-specified subgroup analyses will include comparison of the treatment effect stratified by age (defined according to 10 year age bands, where possible), antihypertensive drug class (if sufficiently powered) and in males vs. females.

M. Plan for addressing confounding

The validity of propensity score matching will be examined using ‘negative controls’: the impact of antihypertensive treatment on an outcome not known to be affected by such treatment. Here, the impact of antihypertensive therapy on hospitalisation and/or death from cancer will be studied as the negative control (aim 6). If treatment with antihypertensives has a significant impact on this outcome, we can conclude there is something missing in the propensity score (i.e. an unmeasurable factor of propensity for treatment such as being generally unwell or an unhealthy lifestyle) causing an imbalance between the treatment and control groups, rather than a true treatment effect.

N. Plan for addressing missing data

There is potential for missing data in this study, particularly with variables such as ethnicity which are recorded with varying degrees of accuracy in routine practice. Because this analysis is focused on treatment of uncomplicated mild hypertension, accurate selection of the sample population is important and therefore any patients with insufficient data available to define their blood pressure status at baseline (index date) will be excluded. Where there is no record of blood pressure lowering, statin or antiplatelet treatment, it will be assumed the patients were not exposed to blood pressure lowering, statin or antiplatelet treatment.

Patient eligibility includes only using ‘acceptable patients’ in the analysis, and therefore there is no need to impute age and sex. Where there is no record of smoking history or alcohol consumption, patients will be assumed to be non-smokers and non-drinkers. Likewise, those with no record of rheumatoid arthritis or hypercholesterolemia will be assumed to have no history of these conditions. All other covariates used in the propensity score model will be imputed using multiple imputation. Ethnicity and IMD will be treated as categorical variable in the imputation model. BMI will be treated as a continuous variable.

The primary analysis examining the efficacy of treatment will be conducted by intention-to-treat, and patients moving practice after the index date (and therefore being lost to follow-up) will be censored at the point at which they are no longer active in the database.

For peer review only - http://bmjopen.bmj.com/site/about/guidelines.xhtml

O. Limitations of the study design, data sources and analytical methods

This study will use an observational cohort design, and as such, there is an inherent selection bias of patients in both intervention and control groups. The impact of this bias will be limited by propensity score matching but such an approach assumes that all factors influencing the propensity for medication prescription have been measured and accounted for within the score. In the present study, we will examine the validity of this assumption by studying the impact of treatment on a negative control: hospitalisation and/or death from cancer. We will conclude the assumption has been met if antihypertensive treatment is shown to have no impact on cancer outcomes, as would be expected from previous trial evidence.

Previous randomised studies examining the efficacy of treatment for uncomplicated stage 1 hypertension have been underpowered to demonstrate a true treatment effect. This study will use data from the largest linked primary care database in the world with a potential sample population of approximately 210,000 patients, sufficient to power the study for the primary and subgroup analyses. In pre-specified subgroup analyses, we will examine the impact of potential confounding factors such as patient adherence to medication.

Strategy for dealing with potential errors resulting from multiple testing

This study includes one descriptive objective to examine the extent to which patients are prescribed pharmacological treatment for uncomplicated, mild hypertension and one hypothesis testing objective to examine whether such treatment is safe and effective at reducing the risk of death and cardiovascular disease. To avoid potential errors arising from multiple testing, a primary outcome has been clearly defined as the impact of antihypertensive treatment on all-cause mortality. Secondary outcomes will examine the impact of antihypertensive treatment on major cardiovascular events (composite endpoint), death or hospitalisation from stroke, death or hospitalisation from MI or non-MI acute coronary syndrome, death or hospitalisation from cancer or hospitalisation with suspected side effects to medication (hypotension, syncope, bradycardia, electrolyte abnormalities, injurious falls and acute kidney injury/renal failure). The primary outcome will be given priority in the final analysis write-up and any related reports and presentations.

Primary and secondary outcomes will be defined using ONS, inpatient HES and read-coded primary care data. The accuracy of such outcome data has been examined previously in patients with acute myocardial infarction[21] and whilst recording of risk factor and co-morbid information was consistent across primary care, hospital admissions and disease registry records, the crude incidence of acute myocardial infarction was underestimated by up to 50% if only one data source was used, compared with using all three sources. The use of linked CPRD data in the present study is therefore crucial to ensure outcome data are ascertained accurately.

P. Patient or user group involvement (if applicable)

We have not carried out specific PPI for this particular project, although PPI work we have recently conducted for similar studies is relevant. In particular, we have conducted focus groups to examine patient views on blood pressure treatment and primary prevention of cardiovascular disease. Specific issues highlighted included that healthy older individuals, like many others, sometimes take their state of good health for granted and few considered that they might be at risk of strokes or heart attacks. The haphazard nature of blood pressure/cholesterol checks by their GP seemed confusing and many opted for an attitude of fatalism over future health. They were often informed by health experiences of friends and popular media opinion and were wary of the potential side-effects of medication (hence preferred not to take it).

For peer review only - http://bmjopen.bmj.com/site/about/guidelines.xhtml

These issues are pertinent in the population of interest, and evidence provided by the present analysis will support shared decision making between patients and doctors as well as informing future cardiovascular disease prevention programmes and policy.

Q. Plans for disseminating and communicating study results, including the presence or absence of any restrictions on the extent and timing of publication

We expect at least two publications arising from this research: one describing the prevalence of pharmacological treatment and lifestyle advice for uncomplicated mild hypertension, and how this has changed over time. The second will explore the safety and efficacy of treatment for mild hypertension. A third publication may be possible exploring the efficacy of treatment in predefined sub-groups in detail. Findings will be presented at scientific meetings and published in journals at the earliest possible convenience. We will endeavour to publish our study findings in lay format on our University website and through lay and social media where appropriate.

For peer review only

For peer review only - http://bmjopen.bmj.com/site/about/guidelines.xhtml

References

1. Lewington S, Clarke R, Qizilbash N, et al. Age-specific relevance of usual blood pressure to vascular mortality: a meta-analysis of individual data for one million adults in 61 prospective studies. *Lancet* 2002;360(9349):1903-13. [published Online First: 2002/12/21]

2. Lozano R, Naghavi M, Foreman K, et al. Global and regional mortality from 235 causes of death for 20 age groups in 1990 and 2010: a systematic analysis for the Global Burden of Disease Study 2010. *Lancet* 2012;380(9859):2095-128. doi: 10.1016/s0140-6736(12)61728-0 [published Online First: 2012/12/19]

3. Whitworth JA. 2003 World Health Organization (WHO)/International Society of Hypertension (ISH) statement on management of hypertension. *Journal of hypertension* 2003;21(11):1983-92. doi: 10.1097/01.hjh.0000084751.37215.d2 [published Online First: 2003/11/05]

4. Sanchez RA, Ayala M, Baglivo H, et al. Latin American guidelines on hypertension. Latin American Expert Group. *Journal of hypertension* 2009;27(5):905-22. doi: 10.1097/HJH.0b013e32832aa6d2 [published Online First: 2009/04/08]

5. Ogihara T, Kikuchi K, Matsuoka H, et al. The Japanese Society of Hypertension Guidelines for the Management of Hypertension (JSH 2009). *Hypertension research : official journal of the Japanese Society of Hypertension* 2009;32(1):3-107. [published Online First: 2009/03/21]

6. Mancia G, Fagard R, Narkiewicz K, et al. 2013 ESH/ESC Guidelines for the management of arterial hypertension: the Task Force for the management of arterial hypertension of the European Society of Hypertension (ESH) and of the European Society of Cardiology (ESC). *Journal of hypertension* 2013;31(7):1281-357. doi: 10.1097/01.hjh.0000431740.32696.cc [published Online First: 2013/07/03]

7. James PA, Oparil S, Carter BL, et al. 2014 evidence-based guideline for the management of high blood pressure in adults: report from the panel members appointed to the Eighth Joint National Committee (JNC 8). *JAMA : the journal of the American Medical Association* 2014;311(5):507-20. doi: 10.1001/jama.2013.284427 [published Online First: 2013/12/20]

8. Hackam DG, Quinn RR, Ravani P, et al. The 2013 Canadian Hypertension Education Program recommendations for blood pressure measurement, diagnosis, assessment of risk, prevention, and treatment of hypertension. *The Canadian journal of cardiology* 2013;29(5):528-42. doi: 10.1016/j.cjca.2013.01.005 [published Online First: 2013/04/02]

9. Toth PJ, Horwitz RI. Conflicting clinical trials and the uncertainty of treating mild hypertension. *The American journal of medicine* 1983;75(3):482-8. [published Online First: 1983/09/01]

10. Tavares A. Pharmacotherapy for mild hypertension. *Sao Paulo medical journal = Revista paulista de medicina* 2012;130(6):417-8. [published Online First: 2013/01/23]

11. Ramsay LE, ul Haq I, Yeo WW, et al. Interpretation of prospective trials in hypertension: do treatment guidelines accurately reflect current evidence? *Journal of hypertension Supplement : official journal of the International Society of Hypertension* 1996;14(5):S187-94. [published Online First: 1996/12/01]

12. Heath I. Waste and harm in the treatment of mild hypertension. *JAMA internal medicine* 2013;173(11):956-7. doi: 10.1001/jamainternmed.2013.970 [published Online First: 2013/05/24]

13. Hart JT. Historical footnote on the treatment of mild hypertension. *BMJ (Clinical research ed)* 2012;345:e6297. doi: 10.1136/bmj.e6297 [published Online First: 2012/09/26]

For peer review only - http://bmjopen.bmj.com/site/about/guidelines.xhtml

14. Wright JT, Jr., Williamson JD, Whelton PK, et al. A Randomized Trial of Intensive versus Standard Blood-Pressure Control. *The New England journal of medicine* 2015;373(22):2103-16. doi: 10.1056/NEJMoa1511939 [published Online First: 2015/11/10]

15. Diao D, Wright JM, Cundiff DK, et al. Pharmacotherapy for mild hypertension. *The Cochrane database of systematic reviews* 2012;8:Cd006742. doi: 10.1002/14651858.CD006742.pub2 [published Online First: 2012/08/17]

16. Sundstrom J, Arima H, Jackson R, et al. Effects of blood pressure reduction in mild hypertension: a systematic review and meta-analysis. *Annals of internal medicine* 2015;162(3):184-91. doi: 10.7326/m14-0773 [published Online First: 2014/12/23]

17. National Clinical Guideline Centre. Hypertension: clinical management of primary hypertension in adults; Clinical guideline 127. London: Royal College of Physicians (UK), 2011.

18. Sheppard JP, Fletcher K, McManus RJ, et al. Prevalence and costs of treating uncomplicated stage 1 hypertension in primary care: a cross-sectional analysis. *The British journal of general practice : the journal of the Royal College of General Practitioners* 2014;64(627):e641-8. doi: 10.3399/bjgp14X681817 [published Online First: 2014/10/01]

19. Public Health England. NHS Health Check: our approach to the evidence. https://www.gov.uk/government/uploads/system/uploads/attachment_data/file/224537/NHS_Health_Check_our_approach_to_the_evidence_v2.pdf: NHS England, 2013.

20. General practitioners committee Ne. 2013/14 general medical services (GMS) contract quality and outcomes framework (QOF): Guidence for GMS contract 2013/14: NHS Employers, 2013.

21. Herrett E, Shah AD, Boggon R, et al. Completeness and diagnostic validity of recording acute myocardial infarction events in primary care, hospital care, disease registry, and national mortality records: cohort study. *BMJ (Clinical research ed)* 2013;346:f2350. doi: 10.1136/bmj.f2350 [published Online First: 2013/05/23]

For peer review only

The RECORD statement – checklist of items, extended from the STROBE statement, that should be reported in observational studies using routinely collected health data.

	Item No.	STROBE items	Location in manuscript where items are reported	RECORD items	Location in manuscript where items are reported
**Title and abstract**					
	1	(a) Indicate the study's design with a commonly used term in the title or the abstract (b) Provide in the abstract an informative and balanced summary of what was done and what was found		RECORD 1.1: The type of data used should be specified in the title or abstract. When possible, the name of the databases used should be included.<br><br>RECORD 1.2: If applicable, the geographic region and timeframe within which the study took place should be reported in the title or abstract.<br><br>RECORD 1.3: If linkage between databases was conducted for the study, this should be clearly stated in the title or abstract.	p1<br><br>p2<br><br>p2
**Introduction**					
Background rationale	2	Explain the scientific background and rationale for the investigation being reported			P4
Objectives	3	State specific objectives, including any prespecified hypotheses			P4
**Methods**					
Study Design	4	Present key elements of study design early in the paper			P4
Setting	5	Describe the setting, locations, and relevant dates, including periods of recruitment, exposure, follow-up, and data collection			P4-5
Participants	6	*(a) Cohort study* - Give the eligibility criteria, and the		RECORD 6.1: The methods of study population selection (such as codes or	P4-5

For peer review only

		sources and methods of selection of participants. Describe methods of follow-up<br>*Case-control study* - Give the eligibility criteria, and the sources and methods of case ascertainment and control selection. Give the rationale for the choice of cases and controls<br>*Cross-sectional study* - Give the eligibility criteria, and the sources and methods of selection of participants<br><br>*(b) Cohort study* - For matched studies, give matching criteria and number of exposed and unexposed<br>*Case-control study* - For matched studies, give matching criteria and the number of controls per case		algorithms used to identify subjects) should be listed in detail. If this is not possible, an explanation should be provided.<br><br>RECORD 6.2: Any validation studies of the codes or algorithms used to select the population should be referenced. If validation was conducted for this study and not published elsewhere, detailed methods and results should be provided.<br><br>RECORD 6.3: If the study involved linkage of databases, consider use of a flow diagram or other graphical display to demonstrate the data linkage process, including the number of individuals with linked data at each stage.	 <br><br><br>n/a<br><br><br><br><br><br><br>Figure 1
Variables	7	Clearly define all outcomes, exposures, predictors, potential confounders, and effect modifiers. Give diagnostic criteria, if applicable.		RECORD 7.1: A complete list of codes and algorithms used to classify exposures, outcomes, confounders, and effect modifiers should be provided. If these cannot be reported, an explanation should be provided.	P5
Data sources/ measurement	8	For each variable of interest, give sources of data and details of methods of assessment (measurement).<br>Describe comparability of assessment methods if there is more than one group			P5
Bias	9	Describe any efforts to address potential sources of bias			P6
Study size	10	Explain how the study size was			P5

For peer review only

		arrived at			
Quantitative variables	11	Explain how quantitative variables were handled in the analyses. If applicable, describe which groupings were chosen, and why			P5
Statistical methods	12	(a) Describe all statistical methods, including those used to control for confounding<br>(b) Describe any methods used to examine subgroups and interactions<br>(c) Explain how missing data were addressed<br>(d) *Cohort study* - If applicable, explain how loss to follow-up was addressed<br>*Case-control study* - If applicable, explain how matching of cases and controls was addressed<br>*Cross-sectional study* - If applicable, describe analytical methods taking account of sampling strategy<br>(e) Describe any sensitivity analyses			p6
Data access and cleaning methods		..		RECORD 12.1: Authors should describe the extent to which the investigators had access to the database population used to create the study population.<br><br>RECORD 12.2: Authors should provide information on the data cleaning methods used in the study.	P6<br><br>P6
Linkage		..		RECORD 12.3: State whether the study included person-level, institutional-	P7

For peer review only

				level, or other data linkage across two or more databases. The methods of linkage and methods of linkage quality evaluation should be provided.	
**Results**					
Participants	13	(a) Report the numbers of individuals at each stage of the study (*e.g.*, numbers potentially eligible, examined for eligibility, confirmed eligible, included in the study, completing follow-up, and analysed)<br>(b) Give reasons for non-participation at each stage.<br>(c) Consider use of a flow diagram		RECORD 13.1: Describe in detail the selection of the persons included in the study (*i.e.,* study population selection) including filtering based on data quality, data availability and linkage. The selection of included persons can be described in the text and/or by means of the study flow diagram.	p7 and figure 1
Descriptive data	14	(a) Give characteristics of study participants (*e.g.*, demographic, clinical, social) and information on exposures and potential confounders<br>(b) Indicate the number of participants with missing data for each variable of interest<br>(c) *Cohort study* - summarise follow-up time (*e.g.*, average and total amount)			p7, table 3
Outcome data	15	*Cohort study* - Report numbers of outcome events or summary measures over time<br>*Case-control study* - Report numbers in each exposure category, or summary measures of exposure<br>*Cross-sectional study* - Report numbers of outcome events or summary measures			p7-8
Main results	16	(a) Give unadjusted estimates			P7-8

For peer review only

		and, if applicable, confounder-adjusted estimates and their precision (e.g., 95% confidence interval). Make clear which confounders were adjusted for and why they were included<br>(b) Report category boundaries when continuous variables were categorized<br>(c) If relevant, consider translating estimates of relative risk into absolute risk for a meaningful time period			
Other analyses	17	Report other analyses done—e.g., analyses of subgroups and interactions, and sensitivity analyses			p8
**Discussion**					
Key results	18	Summarise key results with reference to study objectives			P8
Limitations	19	Discuss limitations of the study, taking into account sources of potential bias or imprecision. Discuss both direction and magnitude of any potential bias		RECORD 19.1: Discuss the implications of using data that were not created or collected to answer the specific research question(s). Include discussion of misclassification bias, unmeasured confounding, missing data, and changing eligibility over time, as they pertain to the study being reported.	P8-9
Interpretation	20	Give a cautious overall interpretation of results considering objectives, limitations, multiplicity of analyses, results from similar studies, and other relevant evidence			P9-10
Generalisability	21	Discuss the generalisability (external validity) of the study results			P9-10

For peer review only

Other Information					
Funding	22	Give the source of funding and the role of the funders for the present study and, if applicable, for the original study on which the present article is based			p11
Accessibility of protocol, raw data, and programming code		..		RECORD 22.1: Authors should provide information on how to access any supplemental information such as the study protocol, raw data, or programming code.	Online appendix

*Reference: Benchimol EI, Smeeth L, Guttmann A, Harron K, Moher D, Petersen I, Sørensen HT, von Elm E, Langan SM, the RECORD Working Committee. The REporting of studies Conducted using Observational Routinely-collected health Data (RECORD) Statement. *PLoS Medicine* 2015; in press.

*Checklist is protected under Creative Commons Attribution (CC BY) license.

For peer review only
